# Genome resolved analysis of a premature infant gut microbial community reveals a *Varibaculum cambriense* genome and a shift towards fermentation-based metabolism during the third week of life

**DOI:** 10.1186/2049-2618-1-30

**Published:** 2013-12-17

**Authors:** Christopher T Brown, Itai Sharon, Brian C Thomas, Cindy J Castelle, Michael J Morowitz, Jillian F Banfield

**Affiliations:** 1 Department of Plant & Microbial Biology, University of California, Berkeley, USA; 2 Department of Earth & Planetary Science, University of California, Berkeley, USA; 3 Department of Surgery, University of Pittsburgh School of Medicine, M240 Scaife Hall, 3550 Terrace Street, Pittsburgh, PA 15261, USA; 4 Department of Environmental Science, Policy, and Management, University of California, Berkeley, USA

**Keywords:** Binning, Community genomics, Genome reconstruction, Human gut, Metagenomics, Microbial dynamics, Premature infant, *Varibaculum cambriense*, Time series binning

## Abstract

**Background:**

The premature infant gut has low individual but high inter-individual microbial diversity compared with adults. Based on prior 16S rRNA gene surveys, many species from this environment are expected to be similar to those previously detected in the human microbiota. However, the level of genomic novelty and metabolic variation of strains found in the infant gut remains relatively unexplored.

**Results:**

To study the stability and function of early microbial colonizers of the premature infant gut, nine stool samples were taken during the third week of life of a premature male infant delivered via Caesarean section. Metagenomic sequences were assembled and binned into near-complete and partial genomes, enabling strain-level genomic analysis of the microbial community.

We reconstructed eleven near-complete and six partial bacterial genomes representative of the key members of the microbial community. Twelve of these genomes share >90% putative ortholog amino acid identity with reference genomes. Manual curation of the assembly of one particularly novel genome resulted in the first essentially complete genome sequence (in three pieces, the order of which could not be determined due to a repeat) for *Varibaculum cambriense* (strain Dora), a medically relevant species that has been implicated in abscess formation.

During the period studied, the microbial community undergoes a compositional shift, in which obligate anaerobes (fermenters) overtake *Escherichia coli* as the most abundant species. Other species remain stable, probably due to their ability to either respire anaerobically or grow by fermentation, and their capacity to tolerate fluctuating levels of oxygen. Metabolic predictions for *V. cambriense* suggest that, like other members of the microbial community, this organism is able to process various sugar substrates and make use of multiple different electron acceptors during anaerobic respiration. Genome comparisons within the family *Actinomycetaceae* reveal important differences related to respiratory metabolism and motility.

**Conclusions:**

Genome-based analysis provided direct insight into strain-specific potential for anaerobic respiration and yielded the first genome for the genus *Varibaculum*. Importantly, comparison of these *de novo* assembled genomes with closely related isolate genomes supported the accuracy of the metagenomic methodology. Over a one-week period, the early gut microbial community transitioned to a community with a higher representation of obligate anaerobes, emphasizing both taxonomic and metabolic instability during colonization.

## Background

The human adult microbiota consists of 10-fold more cells than the human body (the majority reside in the gut) and 100-fold more genes than the human genome [1-3]. The gut microbiota are involved in host nutrient acquisition [4], regulation and development of the host immune system [5,6], and the modulation of host gene expression [7]. All of these influences have the potential to seriously affect human health. Aberrations in gut microbiota membership and community structure, termed microbial dysbiosis, have been associated with obesity [8] and diseases such as inflammatory bowel disease [9], both type 1 and type 2 diabetes [10,11], and necrotizing enterocolitis in premature infants [12-14]. Although previous studies have focused on gut colonization [15,16], few have shown the process in a high-resolution manner [17,18]. Thus, much is still not known about the diversity, metabolic potential, or roles of early gut colonizers.

Although the gut microbiota of infants is characterized by high levels of inter-individual diversity (beta diversity), community composition begins to look like that of adults within the first year of life [15]. In comparison with both adults and infants, premature infants have especially low individual diversity (alpha diversity), making them ideal subjects for high-resolution (species or strain-level) community genomics approaches [17,18]. Continued study of microbial colonization in the gut of premature infants may yield further insights into the details of this process and the implications of disease-associated microbial dysbiosis.

Community genomics, the use of genomes sequenced from natural microbial communities to understand the structure and metabolism of the community, has been successful in environments with varying levels of diversity [17-24]. Recently, this approach has been applied to the human microbiome, where the genomes of abundant bacterial species were assembled from a premature infant [17] and, most recently, where increased sequencing depth allowed for genomes to be assembled for both high-abundance and low-abundance members of the microbial community found in the gut of another premature infant (including genomes for members that make up less than 0.05% of the microbial community) [18]. Both of these studies involved analysis of strain-level variation within the human gut microbiome. In human adults, a draft genome of Shiga-Toxigenic *Escherichia coli* O104:H4 was assembled from metagenome data taken from individuals involved in an outbreak, providing strain-level resolution of this pathogen [25]. Strain-level analysis of microbial communities contrasts strongly with 16S rRNA-based fingerprinting methods that characterize communities with phylum to genus-level resolution. This is primarily due to the added benefit of being able to directly determine the metabolic potential of strains in a particular community (which need not have been previously studied), and to identify metabolic variation between strains that may have highly similar or even identical 16S rRNA gene sequences [26]. In general, the study of infants enables development of an understanding of microbial colonization in humans, and can provide genomes for biologically and medically relevant, and oftentimes novel, species directly from their source environments (without the bias of isolation or cell sorting, or single cell manipulation and genome amplification steps).

Here, we investigate gut colonization in a relatively healthy premature infant during the third week of life with the objectives of comparing genomic novelty between the natural consortia and isolate strains, recovery and analysis of genomes from previously uncharacterized community members, and metabolic analysis of the microbial community. This period of early gut colonization was targeted for intensive sample collection because it is believed that aberrant colonization near this time can contribute to the pathogenesis of necrotizing enterocolitis (which was not observed in this infant). Our approach involves reconstructing complete and near-complete genomes from DNA extracted from fecal samples to enable prediction of the roles of specific species and strains in the community. Time series abundance analysis is a key component of the approach because shifts in community composition can be detected, and also because organism abundance patterns greatly increase the accuracy with which assembled fragments can be assigned to specific organisms (binning; [18]). We show that, even in the human gut, where many species can be represented by reference genomes, there are organisms with genomic potential not represented by reference sequences. Specifically, we report the first genome for the genus *Varibaculum*, a genus that has been implicated in human abscess formation, but that has not been associated with the human gut [27].

## Methods

### Patient, samples, and sequencing

We studied the colonization of the gut of a male (birth weight 1,205 g) born via Caesarean section during the 31st week of pregnancy to a mother with chronic hypertension and superimposed pre-eclampsia. The patient was born at Comer Children’s Hospital at the University of Chicago. He was administered total parenteral nutrition (TPN) soon after admission, but started bolus nasogastric feeding on his second day of life (DOL). The patient was weaned from TPN as he began increasing feeds with fortified breast milk. TPN was discontinued on DOL 6. The patient reached full feeds on DOL 8, continuing to be fed on fortified breast milk. The patient was never intubated but did briefly receive supplemental oxygen. He received antibiotics (ampicillin and gentamicin) only during the first 48 hours of life. Stool samples were collected on the following days of life: 14, 15, 18, 19, and 20. Samples were collected twice daily, except for DOL 14, and stored at −80°C. The patient was discharged in good health on DOL 53.

Microbial DNA was extracted from frozen fecal samples using the QIAamp DNA Stool mini-Kit (Qiagen) with modifications [28]. DNA was sequenced on an Illumina HiSeq2000 sequencer for 101 cycles from each DNA fragment end using the TruSeq SBS sequencing kit (version 2). Sequencing data were handled with pipeline 1.7 according to the manufacturer’s instructions (Illumina, San Diego, CA). The protocol for sample collection and processing was approved by the Institutional Review Board of The University of Chicago (IRB #15895A). All samples were collected with the consent of the infant’s mother.

### Metagenome assembly, binning, and annotation

Environmental shotgun DNA sequences for all samples were processed and assembled as previously described [18]. Sequences were quality trimmed and human DNA was filtered out prior to assembly. Sequences from all samples were co-assembled in a multistep, iterative approach, in which optimal parameters (coverage and k-mer length) for assembly of genomes from specific populations or groups of populations were selected. Velvet [29] was used to assemble the data and the resulting assembly was subjected to quality controls that detect mis-assemblies based on regions of zero insert coverage.

All scaffolds longer than 400 bp were annotated by first predicting open reading frames (ORFs) using the metagenome implementation of Prodigal [30] and then searching translated ORFs against the UniProt UniRef90 database [31] using USEARCH [32] with an *E*-value threshold of 0.001. The coverage of each scaffold was determined by mapping reads using BowTie [33] with the parameters -best and -e 200. Coverage was calculated as the total number of sequence bases mapped to a scaffold divided by the length of the scaffold.

Clustering of scaffolds into genome bins was conducted as previously described [18]. Specifically, the Databionic implementation of an emergent self-organizing map (ESOM; [34]) was used to cluster scaffolds longer than 400 base pairs (bp) based on their time series abundance patterns (after first breaking scaffolds into 1.5 kbp fragments) (Additional file 1). This allowed us to bin scaffolds into near-complete and partial genomes. Genome bins were assessed in part by coloring fragments clustered on the ESOM based on the best BLAST [35] hit of each scaffold against the NCBI NT database [36] (Additional file 2: Figure S1). Bins were manually extracted by contouring fields on the ESOM. Genome completeness was determined by comparing the length of each putative genome with the most closely related reference genome and by searching for 26 universal single copy marker genes (Additional file 3 and [37]).

### Manual curation of microbial genome bins

Each genome bin was evaluated based on its size, coverage, and the presence of single copy genes. Single copy genes were used to estimate genome completeness and to determine whether multiple genomes were being clustered into a single bin. Incomplete bins may be the result of several factors: (i) low coverage can prevent an entire genome from being assembled; (ii) sequence variation can cause genomic regions unique to strains to assemble separately, sometimes generating very small contigs that cannot be binned; (iii) strain-specific genomic regions will be binned separately from shared regions if the time series abundance patterns of the strains differ; and (iv) inherent noise in coverage calculations can result in scaffolds representing a genome being placed in separate bins. Bins with similar taxonomic affiliations were evaluated to determine whether their scaffolds were split into different bins owing to strain variation or noise in coverage calculations. Such bins were subsequently combined into a single genome bin if they did not contain redundant single copy genes. Other bins with similar taxonomic affiliations but with overall different time series abundance patterns were considered to be the result of strain variation.

The genome assembled and binned for *V. cambriense* was reassembled and manually curated. This involved analyzing the read mapping for the entire metagenome assembly and capturing the reads (along with their pairs) that mapped to scaffolds in the *V. cambriense* bin. These reads were then used in several Velvet assemblies, in which the parameters for k-mer length and expected coverage were altered. Genome size was estimated based on the size of the bin and used to evaluate the Velvet assemblies. The best assembly based on expected length and N50 was checked for mis-assemblies (scaffolds were split at regions with zero insert coverage) and then manually curated.

The genome for *V. cambriense* was manually curated using a suite of in-house scripts designed to extend scaffolds by recruiting paired-reads that extend from existing scaffolds. These paired-reads were assembled independently using Velvet. Their resulting contigs were compared with existing scaffolds, based on their sequence similarity determined by BLAST. Regions of high similarity between the newly assembled contigs and existing scaffolds oftentimes reveal scaffolds that could be combined with one another [18].

Open reading frames for the manually curated *V. cambriense* genome were annotated using an in-house pipeline that includes BLAST-based homology searches against the NCBI NR [36], KEGG [38], UniRef90, and COG [39] databases, in addition to HMM-based functional domain recognition searches using InterProScan [40]. Metabolic analysis of the functional predictions for the *V. cambriense* genome was completed using Pathway Tools [41] and KEGG [42] (Additional file 4 and Additional file 5).

### Plasmid and phage genomes

Plasmid genomes were identified by searching for potentially circular scaffolds by computing the Needleman-Wunsch [43] global alignment for the first and last 100 bp of the scaffold. Scaffolds with high overlap identity were further analyzed by searching those scaffolds for genes indicative of plasmids (such as plasmid replication and maintenance proteins). Putative phage fragments were identified by searching for phage-related genes (such as the capsid or tail fibers) on unbinned scaffolds and on scaffolds binned along with a genome. Phage scaffolds binned with bacterial genomes have significantly higher coverage than the bacterial-associated scaffolds.

Plasmids were associated with individual species in the community by comparing the abundance patterns of each plasmid with each species and by leveraging plasmid phylogenetic annotations. To narrow down the list of possible host organisms for each plasmid, Pearson correlation coefficients were calculated on the abundance pattern of each plasmid compared with each bacterial species. Putative phage fragments were associated with species based on initial ESOM binning, searching for integration sites in reconstructed genomes, and by comparing the relative abundance patterns of phage with bacterial species (assisted by the Pearson correlation coefficient).

Plasmid novelty and diversity were determined by building a phylogenetic tree of plasmid replication proteins. Sequences representative of closely related plasmid replication proteins were acquired by searching the NCBI NR database using BLAST. The amino acid sequences were aligned using MUSCLE [44] and a phylogenetic tree was reconstructed using FastTree2 [45] with the Jones-Taylor-Thornton model of amino acid evolution and by assuming a single rate of evolution for each site (known as the “CAT” approximation) [46]. Local support values were calculated with the Shimodaira-Hasegawa test [47] and the tree was formatted using FigTree [48].

### Coverage and abundance calculations

Coverage was calculated for each scaffold based on mapped reads. Absolute abundance, average coverage, and relative abundance were calculated for reconstructed genomes at each time point, in order to represent changes in microbial community structure (Additional file 2: Tables S2-5). Genome coverage was calculated as the number of bases mapped to the genome divided by the total length of the genome. Relative abundance was calculated for a genome by taking the average coverage of the genome and normalizing it by the sum of the average coverage values for all genomes. Thus, relative abundance is the abundance of a genome taken as a percent of the total abundance of all genomes. Absolute abundance was calculated for each genome by dividing the total number of sequence bases that mapped to the genome by the total number of bases associated with reads used in the assembly. Rank abundance was calculated from the relative abundance of each genome from the combined read mapping of the time points in each phase of community colonization (phases were defined after observing community abundance patterns across the time series). Plots of relative abundance were created using the R plot function [49].

### Microbial community composition based on EMIRGE 16S rRNA genes

EMIRGE [50] was used to reconstruct 16S rRNA gene sequences from the metagenomic data (Additional file 6). The closest relatives of each sequence were found by searching a TaxCollector [51] version of the Ribosomal Database Project (RDP) database [52] and the GreenGenes [53] database using BLAST. Reconstructed 16S rRNA gene sequences were connected with genomes based on several criteria. First, paired-end sequences were used to link scaffolds carrying fragments of 16S rRNA gene sequences to genome scaffolds. Then, coverage and taxonomic information, from both marker genes and for the genome overall, were used to refine associations when paired-read connections were inconclusive. More 16S rRNA gene sequences were reconstructed by EMIRGE than could be represented by genome bins; thus, a subset of low-abundance 16S rRNA gene sequences were assumed to be either incorrectly reconstructed or from very rare community members, and thus were disregarded during further analyses.

Reconstructed 16S rRNA gene sequences were aligned along with sequences from their closest relatives and additional species previously reported to be in the infant gut. Sequences were aligned with PyNAST [54] using the GreenGenes [53] alignment of operational taxonomic units (OTUs) classified at 97% sequence similarity as a template. FastTree2 was used to construct the phylogenetic tree using the generalized time-reversible model for nucleotide evolution [55] and the CAT approximation. Local support values were calculated with the Shimodaira-Hasegawa test. The tree was rooted with the 16S rRNA gene sequence for *Halobacterium salinarum* and formatted using FigTree.

### Comparison of genomes with reference genomes

Each complete, near-complete, and partial bacterial and plasmid genome was compared to the genome of its closest sequenced relative. Both complete and draft genomes from NCBI were used in the comparison. The most closely related bacterial genomes were determined using reconstructed 16S rRNA gene sequences, ribosomal protein L5, ribosomal protein S15, and hits to other protein sequences in UniRef90. Aligning reconstructed plasmid genomes to all available sequenced plasmid genomes identified their most closely related relatives. Once selected, each genome was compared to its reference genome. The shared amino acid identity between each reconstructed and reference genome was calculated as the average amino acid identity of reciprocal best USEARCH hits between the two genomes (putative orthologs).

### Evaluating community oxygen tolerance and respiration capability

Community oxygen tolerance and respiration capacity were evaluated based on the presence of specific genes in the metagenome. To assess the oxygen utilization capacity of the community, all predicted ORFs were searched for cytochrome *c* oxidase, cytochrome *bd* oxidase, and heme-copper cytochrome oxidase genes based on assignments from UniRef90. To evaluate the potential for the community to use various terminal electron acceptors in anaerobic respiration, UniRef90 annotations were searched for the presence of fumarate, trimethylamine *N*-oxide (TMAO), dimethyl sulfoxide (DMSO), nitrate, nitrite, and nitric oxide reductase genes. To confirm the annotations for these genes in the *V. cambriense* genome, a phylogenetic tree was reconstructed with the amino acid sequences of the putative catalytic subunits for the nitrate reductase and DMSO reductase genes. The tree was reconstructed using MEGA5 [56] to produce a maximum-likelihood phylogeny calculated with 100 bootstrap replicates based on the Jones-Taylor-Thornton model of amino acid evolution. All positions containing alignment gaps and missing data were eliminated based on pairwise sequence comparisons (pairwise deletion option).

### Analysis of Human Microbiome Project data

The Human Microbiome Project (HMP) [57] hosts QIIME [58] output files for the HMP 16S rRNA Clinical Production Phase I and the HMP 454 Clinical Production Pilot studies (NCBI SRA projects SRP002395 and SRP002012, respectively), which together consist of over 5,700 samples. The 16S rRNA gene variable region 3 to 5 (V35) was sequenced for all samples and the 16S variable region 1 to 3 (V13) was sequenced for a subset of 2,911 samples. The OTU abundance matrices were downloaded for each dataset (V35 and V13) and the abundance of each OTU was calculated as a percent of total reads for each sample. These tables were used to evaluate the relative abundance of *Varibaculum* across samples and body sites in the HMP data collected for healthy human adults.

### Comparative genomics

Although there are no previously sequenced genomes for any member of the genus *Varibaculum*, several complete and draft genomes are available for members of the family *Actinomycetaceae*. These genomes, along with the genomes reconstructed from the microbial community of this premature infant, were used in a comparative analysis. Each genome was annotated by finding reciprocal best USEARCH hits between each genome and a subset of the KEGG database containing only prokaryotic protein sequences with KOs (with a minimum bit score of 40 and maximum *E*-value of 0.01). Metabolic functional potential was compared across genomes by identifying gene sequences associated with specific metabolic functions in each genome (see Additional file 7 for a complete list of these proteins and their associations). These findings were visualized by normalizing the number of genes identified for each function and then using the R pheatmap library to produce a heatmap clustered using the complete linkage method on a Euclidean distance matrix.

## Results

### Metagenome sequencing, assembly, binning, and annotation

The nine samples collected on days of life 14 through 20 from the infant in our study resulted in 35 gigabase pairs (Gbp) of paired-end Illumina DNA sequences with a length of 101 nucleotides. Filtering out human DNA and quality trimming resulted in 27.8 Gbp of Illumina reads with an average length of 93 bp. The iterative metagenome assembly method used resulted in 89.29% of high quality reads being assembled into 12,184 scaffolds longer than 400 bp (40.8 megabase pairs (Mbp), N50: 13,265 bp, longest scaffold: 608,611 bp). From the scaffolds larger than 400 bp, 46,156 ORFs were predicted (average amino acid length: 254), 94.4% of which had a match to the UniRef90 database with an *E*-value less than or equal to 0.001.

**Figure 1:**
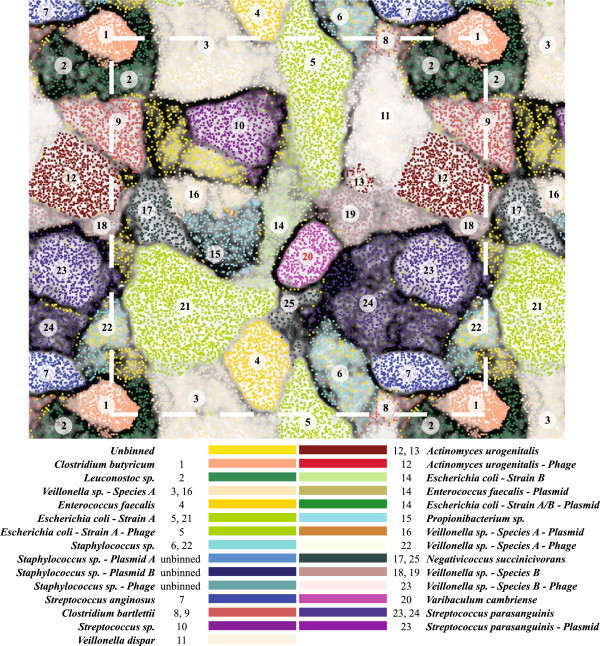
**Emergent self-organizing map (ESOM) binning of the metagenome assembly.** ESOM showing the clustering and binning of *de novo* assembled metagenomic data. Each point represents a fragment of an assembled scaffold. Clustering of data points is based on the time series abundance pattern of each assembled scaffold. Dark lines between clusters show definitive separation of genome bins. Colors designate the genome bin for each scaffold fragment.

Scaffolds were clustered based on their time series abundance patterns using an ESOM, resulting in 25 bins (Figure 1, Additional file 2: Table S1 and Additional file 1). These bins represent complete, near-complete, and partial bacterial, plasmid, and viral genomes (Table 1 and Additional file 2: Table S1) and 85.98% of high quality sequencing reads. Six complete (circular) plasmids were assembled along with five putative phage fragments (Table 1 and Additional file 2: Table S4). Plasmid and phage fragments account for only 0.27% and 0.23% of the total sequence data, respectively; however, they account for the majority of the community in terms of relative abundance (41.7% and 16.6%; Table 1 and Additional file 2: Table S4).

**Table 1:** Assessment of genomes reconstructed from the shotgun-sequenced microbial community: assembly, binning, phylogeny, and genome completeness

**Genome**	**Near-complete genome**	**Phylum**	**Bin**	**Genome size (bp)**	**Genome relative abundance (%)**	**N50 (bp)**	**Number of ORFs**	**Predicted% single copy genes**
*Escherichia coli - strain A/B - plasmid*	Yes	n/a	14	3,225	41.67	3,225	5	n/a
*Escherichia coli - strain A - phage*		n/a	5	3,920	16.57	3,920	4	n/a
*Enterococcus faecalis - plasmid*	Yes	n/a	14	5,231	5.98	5,231	5	n/a
*Streptococcus anginosus*	Yes	Firmicutes	7	2,108,491	5.14	537,826	2,252	100.0
*Escherichia coli - strain A*		Proteobacteria	5, 21	5,662,200	4.91	5,304	7,405	48.1
*Actinomyces urogenitalis - phage*		n/a	12	2,996	4.85	2,996	4	n/a
*Veillonella dispar*	Yes	Firmicutes	11	2,445,194	3.99	53,688	2,693	100.0
*Veillonella sp. - species A - phage*		n/a	22	20,453	3.49	20,453	32	n/a
*Veillonella sp. - species B - phage*		n/a	23	16,533	2.43	16,533	30	n/a
*Actinomyces urogenitalis*	Yes	Actinobacteria	12, 13	2,604,957	2.28	3,337	3,035	92.6
*Clostridium butyricum*	Yes	Firmicutes	1	4,350,784	2.17	103,127	4,094	100.0
*Veillonella sp. - species A - plasmid*	Yes	n/a	16	47,631	1.20	47,631	56	n/a
*Veillonella sp. - species A*		Firmicutes	3, 16	2,664,763	1.03	4,180	3,427	37.0
*Enterococcus faecalis*	Yes	Firmicutes	4	2,960,721	0.76	235,714	2,906	100.0
*Staphylococcus sp. - plasmid B*	Yes	n/a	Unbinned	2,539	0.65	2,539	2	n/a
*Staphylococcus sp. - plasmid A*	Yes	n/a	Unbinned	2,556	0.54	2,556	3	n/a
*Staphylococcus sp. - phage*		n/a	Unbinned	2,423	0.42	2,423	5	n/a
*Escherichia coli - strain B*		Proteobacteria	14	633,084	0.37	4,041	873	3.7
***Varibaculum cambriense***	Yes	**Actinobacteria**	**20**	**2,247,641**	**0.37**	**240,417**	**1,954**	**100.0**
*Streptococcus parasanguinis - plasmid*	Yes	n/a	23	8,975	0.31	8,975	7	n/a
*Veillonella sp. - species B*		Firmicutes	18, 19	639,180	0.27	2,044	879	29.6
*Clostridium bartlettii*	Yes	Firmicutes	8, 9	2,685,446	0.18	12,095	2,633	92.6
*Negativicoccus succinicivorans*	Yes	Negativicoccus	17, 25	1,508,898	0.10	15,236	1,686	100.0
*Staphylococcus sp.*		Firmicutes	6, 22	1,509,765	0.09	9,200	1,634	33.3
*Propionibacterium sp.*		Propionibacterium	15	336,576	0.07	1,117	565	18.5
*Streptococcus parasanguinis*	Yes	Firmicutes	23, 24	2,822,032	0.06	3,494	3,647	77.8
*Leuconostoc sp.*		Firmicutes	2	566,369	0.06	1,603	874	25.9
*Streptococcus sp.*		Firmicutes	10	1,915,777	0.05	11,008	2,114	55.6

n/a, not available.

### Microbial genome identification and curation

Well-defined genomes were binned for *Clostridium butyricum, Enterococcus faecalis, Streptococcus anginosus*, *Streptococcus sp.,* and *Varibaculum cambriense.* However, some bins were not clearly delineated owing to the low abundance of the associated species (at the limits of sequencing detection), similar abundance patterns between species, or coverage miscalculations due to strain variation. This was the case for the bins of *Actinomyces urogenitalis*, *Clostridium bartlettii*, two *Escherichia coli* strains, *Leuconostoc sp.*, *Negativicoccus succinicivorans, Propionibacterium sp., Staphylococcus sp., Streptococcus parasanguinis*, *Veillonella dispar*, and two additional *Veillonella* species. Manual curation of these bins resulted in near-complete and partial genome reconstructions for these species, and revealed strain-resolved genomic novelty within the *E. coli* population (Table 1 and [59]).

We evaluated the completeness of each genome using a list of 26 single copy marker genes (Additional file 3 and [37]), revealing that the genomes for *Actinomyces urogenitalis, Clostridium bartlettii, Clostridium butyricum, Enterococcus faecalis, Negativicoccus succinicivorans, Streptococcus anginosus, Streptococcus parasanguinis, Varibaculum cambriense,* and *Veillonella dispar* (9 out of the 17 total genomes) are near-complete (over 75% of marker genes could be identified) (Table 1).

The genome for *Varibaculum cambriense* was reassembled and manually curated. Before reassembly, the genome was represented by 35 scaffolds with a total length of 2.25 Mbp and an N50 of 240,417 bp. Following reassembly and manual curation, the genome was assembled into three scaffolds, each terminated by a repeat sequence corresponding to a transposase gene. The three scaffolds include completely assembled 16S rRNA and 23S rRNA genes, a total length of 2.28 Mbp, an N50 of 1,648,569 bp, and 105-fold coverage. The *V. cambriense* genome has a GC-content of 52.5%. Approximately 70% of ORFs could be assigned to a putative function. All three scaffolds are connected to each other but their order cannot be determined; thus, all connections are resolved and we consider this genome to be essentially complete. Furthermore, all of the single copy marker genes used to assess genome completeness could be identified along with all 20 aminoacyl tRNA synthetase genes in the *V. cambriense* genome (Additional file 3).

The assembled *E. coli* plasmid has the highest copy number of any assembled plasmid, phage, or bacterial member of the community (Table 1 and Additional file 2: Table S4). The plasmid contains a replication protein distinct from other *Enterobacteriaceae* plasmids, suggesting that it is novel (Additional file 2: Figure S3). The largest plasmid (47.63 kbp) is associated with *Veillonella sp. - species A*, and also contains a novel replication protein (Additional file 2: Figure S3), indicating that this plasmid has not been previously studied. Two plasmids are related to known *Staphylococcus* plasmids and are highly correlated with one another (Pearson coefficient = 0.89), although they are not closely related to one another (based on their replication proteins; Additional file 2: Figure S3). Based on the annotations and abundance patterns for these plasmids, their host is the *Staphylococcus sp.*, for which we reconstructed a near-complete genome (Pearson coefficients of 0.45 and 0.62). Additionally, a complete plasmid genome was assembled and associated with *S. parasanguinis* (based on protein annotations and time series abundance patterns; Pearson coefficient = 0.99).

### Phylogenetic placement of EMIRGE-based 16S rRNA genes

EMIRGE reconstructed 77 candidate 16S rRNA gene sequences, 14 of which could be associated with reconstructed genomes (Table 2 and Additional file 6). The discrepancy between the number of EMIRGE sequences and genomes suggests that EMIRGE is overestimating the number of OTUs. Genes constructed by EMIRGE that could not be assigned to genomes were related to *Shigella* (probably represented by binned *E. coli* genomes), *Okadaella, Buttiauxella, Brevibacterium,* and *Citrobacter*. Of these, low-abundance ORFs from the community could be assigned to the genus *Brevibacterium* at greater than 90% amino acid identity, and to *Citrobacter*, but at less than 90% amino acid identity, suggesting the possible presence of these genera in low abundance in the community. EMIRGE sequences that were not connected with bins were not analyzed further.

**Table 2:** Comparison of reconstructed genomes and 16S rRNA gene sequences with reference databases

**Genome**	**Genome size (bp)**	**Closest relative with sequenced genome**	**Closest relative genome size (bp)**	**% ORFs orthologous to reference**	**Average% amino acid ID of orthologs**	**EMIRGE 16S rRNA gene match**	**EMIRGE 16S% ID**
*Escherichia coli - strain A/B - plasmid*	3,225	*Salmonella enterica subsp. enterica serovar Newport str. SL254 plasmid*	684	20.0	93.9	n/a	n/a
*Escherichia coli - strain A - phage*	3,920	n/a	n/a	n/a	n/a	n/a	n/a
*Enterococcus faecalis - plasmid*	5,231	*Enterococcus faecalis 62 plasmid*	1,295	100.0	100.0	n/a	n/a
*Streptococcus anginosus*	2,108,491	*Streptococcus anginosus 1 2 62CV uid62163*	1,821,055	67.7	95.2	*S. anginosus*	99.8
*Escherichia coli - strain A*	5,662,200	*Escherichia coli S88 uid62979*	5,032,268	47.7	97.5	*E. coli O83:H1 str. NRG 85*	99.9
*Actinomyces urogenitalis - phage*	2,996	n/a	n/a	n/a	n/a	n/a	n/a
*Veillonella dispar*	2,445,194	*Veillonella dispar ATCC 17748*	2,118,767	60.0	91.6	*V. dispar*	99.3
*Veillonella sp. - species A - phage*	20,453	n/a	n/a	n/a	n/a	n/a	n/a
*Veillonella sp. - species B - phage*	16,533	n/a	n/a	n/a	n/a	n/a	n/a
*Actinomyces urogenitalis*	2,604,957	*Actinomyces urogenitalis DSM 15434*	2,702,812	67.1	98.7	*A. urogenitalis*	99.9
*Clostridium butyricum*	4,350,784	*Clostridium butyricum 5521*	4,540,699	73.5	96.5	*C. butyricum*	99.3
*Veillonella sp. - species A - plasmid*	47,631	*Caldicellulosiruptor kristjanssonii 177R1B plasmid*	3,674	3.6	38.8	n/a	n/a
*Veillonella sp. - species A*	2,664,763	*Veillonella dispar ATCC 17748*	2,118,767	47.1	95.6	*Veillonella sp. oral taxon 158*	91.2
*Enterococcus faecalis*	2,960,721	*Enterococcus faecalis OG1RF*	2,739,625	79.1	98.8	*E. faecalis OG1RF*	98.7
*Staphylococcus sp. - plasmid B*	2,539	*Staphylococcus haemolyticus JCSC1435 plasmid*	402	100.0	99.8	n/a	n/a
*Staphylococcus sp. - plasmid A*	2,556	*Macrococcus caseolyticus JCSC5402 plasmid*	997	33.3	80.3	n/a	n/a
*Staphylococcus sp. - phage*	2,423	n/a	n/a	n/a	n/a	n/a	n/a
*Escherichia coli - strain B*	633,084	*Escherichia coli S88 uid62979*	5,032,268	52.7	82.3	n/a	n/a
***Varibaculum cambriense***	**2,247,641**	***Mobiluncus mulieris ATCC 35239***	**2,533,633**	**56.0**	**53.9**	***V. cambriense***	**99.2**
*Streptococcus parasanguinis - plasmid*	8,975	*Enterococcus faecium Aus0004 plasmid*	1,192	14.3	32.4	n/a	n/a
*Veillonella sp. - species B*	639,180	*Veillonella dispar ATCC 17748*	2,118,767	62.5	86.7	*V. parvula DSM 2008*	90.3
*Clostridium bartlettii*	2,685,446	*Clostridium bartlettii DSM 16795*	2,972,256	81.7	98.1	*Clostridium sp. MDA2315*	99.4
*Negativicoccus succinicivorans*	1,508,898	*Bacillus coagulans 36D1*	3,552,226	45.0	43.4	*N. succinicivorans*	98.0
*Staphylococcus sp.*	1,509,765	*Staphylococcus epidermidis ATCC 12228*	2,499,279	78.3	98.4	*S. epidermidis ATCC 12228*	96.2
*Propionibacterium sp.*	336,576	*Propionibacterium 5 U 42AFAA*	2,532,807	46.4	82.4	*Propionibacterium sp. H456*	99.4
*Streptococcus parasanguinis*	2,822,032	*Streptococcus parasanguinis ATCC 15912*	2,153,652	45.0	94.3	*S. parasanguinis*	98.9
*Leuconostoc sp.*	566,369	*Leuconostoc citreum KM20*	1,796,284	66.7	96.0	n/a	n/a
*Streptococcus sp.*	1,915,777	*Streptococcus M334*	2,207,013	67.6	93.9	n/a	n/a

Reconstructed genomes were compared with the genomes of isolate strains. Reconstructed 16S rRNA genes were searched against sequences in the RDP and GreenGenes databases to aid classification. n/a, not available.

The 16S rRNA gene sequences reconstructed with EMIRGE were used to build a phylogenetic tree to classify organisms in the community (Additional file 2: Figure S2). The tree shows that many of the reconstructed genomes are very closely related to sequenced genomes, based on their 16S rRNA gene sequences. The tree highlights the lack of reference genomes for *Varibaculum*, although there are numerous 16S rRNA gene sequences from isolates and from clone libraries. Phylogenetic placement of the EMIRGE sequence for bin 20 confirms that this genome is from a member of the species *V. cambriense* (99.2% 16S rRNA gene sequence identity).

### Comparison of reconstructed genomes to reference genomes

The 25 ESOM bins represent the genomes of 17 unique organisms from the microbial community. Of the nine reconstructed near-complete genomes, seven share over 90% ortholog amino acid identity with reference strains, while five share over 95% ortholog amino acid identity (with at least 60% of ORFs being defined as orthologs) (Table 2). Despite this high-level of similarity, 14% of the ORFs predicted for near-complete reconstructed genomes do not have orthologs within their most closely related reference genomes. At the extreme, 57% of the predicted ORFs for *Negativicoccus succinicivorans* are not orthologous with genes found in the most closely related reference genome (Table 2). Although the level of genomic divergence observed here does not fall outside of the range previously observed [60], this finding underscores the importance of genome reconstructions, as opposed to 16S rRNA gene sequence analysis, for inferring microbial metabolic potential. Of particular note, the genome for *V. cambriense* has an average ortholog amino acid sequence identity of 54% with the genome of its closest sequenced relative, *Mobiluncus mulieris* (Table 2).

### Evidence for the importance of anaerobic metabolism and oxygen tolerance

**Figure 2:**
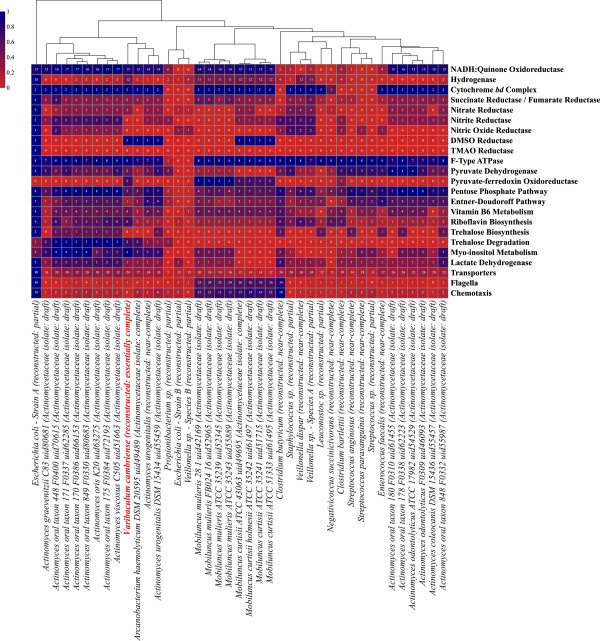
**Metabolic analysis of reconstructed community and isolate genomes.** Genomes reconstructed from the microbial community were compared with each other and with the genomes of cultured isolates previously sequenced for members of the family *Actinomycetaceae*. Each genome was annotated with KEGG and the genes that matched specific metabolic features were counted (Additional file 7). The number of genes identified for each group was normalized across genomes to facilitate coloring and clustering. The number of genes identified for each feature in each genome is presented on the heatmap.

Several genomes encode cytochrome *bd* oxidase (Table 3 and Figure 2), a high oxygen affinity enzyme indicative of an ability to grow in the presence of low levels of oxygen, either by providing protection from reactive oxygen species or by using oxygen as a terminal electron acceptor during respiration [61,62]. The presence of fumarate, TMAO, DMSO, nitrate, nitrite, and nitric oxide reductase genes supports the notion that members of the community are capable of using several terminal electron acceptors to respire anaerobically (Table 3, Figure 2, and Additional file 2: Figure S4). *E. coli* was the only organism found to encode heme-copper cytochrome oxidase genes, indicating its ability to use oxygen as an electron acceptor when present. To further assess the oxygen utilization capacity of the community, all binned and unbinned ORFs were searched for cytochrome *c* oxidase genes, which would indicate aerobic, or possibly aero-tolerant metabolism [62], but none were found. Taken together, we conclude that all organisms in the community are either obligate or facultative anaerobes (Table 3 and Figure 2).

**Table 3:** Metabolism of bacterial members of the microbial community

**Genome**	**Predicted oxygen requirement**	**NADH: quinone oxidoreductase**	**Cytochrome *bd *** **complex**	**Fumarate reductase**	**TMAO reductase**	**DMSO reductase**	**Nitrate reductase**	**Nitrite reductase**	**Nitric oxide reductase**
*Streptococcus anginosus*	Obligate anaerobe								
*Escherichia coli - strain A*	Facultative anaerobe	*	*	*	*	*	*	*	*
*Veillonella dispar*	Obligate anaerobe		*	*			*	*	*
*Actinomyces urogenitalis*	Facultative anaerobe	*	*	*		*	*	*	
*Clostridium butyricum*	Obligate anaerobe					*	*	*	
*Veillonella sp. - species A*	Obligate anaerobe		*	*			*	*	*
*Enterococcus faecalis*	Facultative anaerobe		*						
*Escherichia coli - strain B*	Facultative anaerobe						*		*
***Varibaculum cambriense***	**Facultative anaerobe**	*	*	*		*	*		
*Veillonella sp. - species B*	Obligate anaerobe						*		*
*Clostridium bartlettii*	Obligate anaerobe								
*Negativicoccus succinicivorans*	Obligate anaerobe						*		
*Staphylococcus sp.*	Facultative anaerobe		*				*	*	*
*Propionibacterium sp.*	Facultative anaerobe	*		*				*	
*Streptococcus parasanguinis*	Obligate anaerobe								*
*Leuconostoc sp.*	Facultative anaerobe		*						
*Streptococcus sp.*	Facultative anaerobe								

Presence (*) and absence of components required for anaerobic respiration and the predicted oxygen requirement of each member of the bacterial community.

### Microbial abundance and community shifts

**Figure 3:**
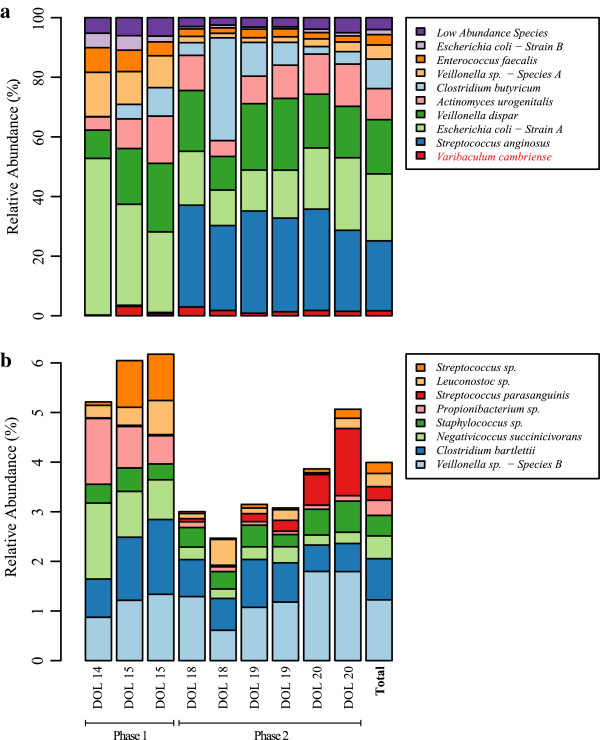
**Relative abundance of bacterial species over time.** Relative abundances were calculated for bacterial species at nine different time points during the third week of life of a premature male infant. **(a)** Shows dominant taxa and **(b)** shows low-abundance species across the time series. During this period, the colonization process is defined by two distinct phases based on the dominance of either facultative (phase 1) or obligate (phase 2) anaerobes.

**Figure 4:**
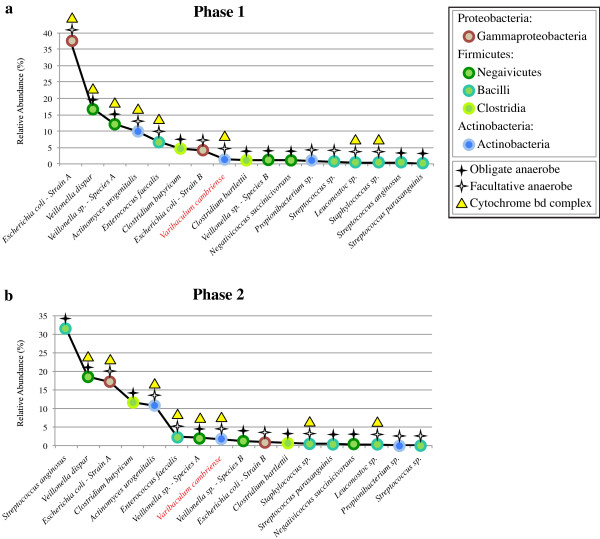
**Rank abundance of bacterial species during phases of colonization.** Rank abundance was determined from the relative abundance of each species during each phase of microbial colonization. Taxonomic identification and metabolic analysis was completed based on genome reconstructions from the shotgun-sequenced microbial community. The colonization process is broken into two distinct phases defined by the dominance of either **(a)** facultative anaerobes during phase one or **(b)** obligate anaerobes during phase two.

*Escherichia coli - strain A* accounts for the greatest percentage of high quality sequence data and *Propionibacterium sp.* accounts for the least amount (33.45% and 0.03%, respectively; Additional file 2: Table S2). Species abundance patterns over the third week of life defined two phases of microbial community composition (Figures 3 and 4). The first phase is observed during DOL 14 to DOL 15, whereas the second covers DOL 18 to DOL 20. The first phase is defined by a dominant *E. coli* strain (a facultative anaerobe), and the second phase is dominated by obligate anaerobes (*Streptococcus anginosus, Clostridium butyricum,* and *Veillonella dispar*). Early in the second phase (first time point on DOL 18) there is a significant increase in the relative abundance of *Streptococcus anginosus,* followed by a stable abundance afterwards. This is followed by a spike in the abundance of *Clostridium butyricum* observed on the second time point taken on DOL 18, after which the relative abundance immediately decreases. There is no apparent clinical variable (for example, change in diet or medication) that accounts for the shift between phase one and phase two. The distinct difference in community composition between theses phases corresponds with a shift towards fermentation-based metabolism in the successors of initially dominant *E. coli*.

*V. cambriense* is nearly undetectable during the first time point (0.15%) and remains at a low abundance throughout the time series (always ≤3%).

It is interesting to note that *Streptococcus, Escherichia, Veillonella, Actinomyces,* and *Enterococcus* dominate the microbial community and that, similar to observations in other premature infants, no *Bacteroides*, *Bifidobacterium*, or *Lactobacillus* were observed throughout the time series [17,18,63].

### Metabolism of *V. cambriens*e based on genomic analysis

*V. cambriense* (strain Dora) (Table 3) became the focus of further analysis for several reasons, including (i) the lack of reference genomes available for *Varibaculum* (Table 2 and Additional file 2: Figure S2), (ii) the availability of a new, essentially complete genome (Table 1), and (iii) the fact that members of this genus are medically relevant and drastically understudied in the human gut [27,64,65]. Further, *Varibaculum* have never been studied in the human gut at a species (or genome) level of resolution. Finally, there is some metabolic information in *Bergey’s Manual of Systematic Bacteriology* for cultured strains of *V. cambriense*[66].

### *V. cambriense* cell wall and motility

Genome analysis shows that genes involved in the lipopolysaccharide biosynthesis pathway are missing, confirming that *V. cambriense* does not have a Gram-negative cell envelope. The peptidoglycan biosynthesis pathway containing meso-diaminopimelate is complete. However, the β-lactam resistance pathway for peptidoglycan synthesis is incomplete, suggesting sensitivity to β-lactam antibiotics. No genes for flagella, pili, or chemotaxis were identified, indicating that this strain, like cultured members of this species, is not motile.

### *V. cambriense* transporters and resistance

Twenty different sugar transport ORFs were identified, indicating that *V. cambriense* has the ability to use many different types of sugars. A putative sialic acid transporter is present, along with genes required for metabolizing *N*-acetylneuraminate (discussed later), suggesting that this abundant component of both human and non-human cell-surface glycoproteins, human breast milk glycans, and intestinal mucins can be used as a nutrient. Additionally, a glycerol-3-phosphate transporter, a putative phosphotransferase IIA system, a sodium-galactoside symporter, and multiple sugar transport system permease genes were identified. No acetate transporter was found, although several pathways were identified for converting pyruvate into acetate for the bidirectional conversion between acetyl-CoA and acetate. Complete KEGG modules suggest that *V. cambriense* can transport ribose, phosphate, nickel, lipopolysaccharides, and fructose.

Although no complete antibiotic resistance pathways were identified, a drug resistance transporter (EmrB/QacA subfamily) and a methicillin resistance protein are coded for in the genome. *V. cambriense* has various resistance mechanisms, including an arsenate resistance pathway, the pathway for glycine betaine biosynthesis (a compound capable of protecting against osmotic stress; [67]), and a P-type ATPase for translocating copper and silver (suggesting Cu^2+^ tolerance).

The genome contains the enzyme trehalose synthase, which is necessary for trehalose synthesis from β-maltose. The enzymes for synthesizing trehalose from glycogen were also identified, but not the enzymes for degrading trehalose. Trehalose has several biological roles, including that of structural component [68] and stress protector [69]. The pathway for ppGpp is also encoded by the genome; this pathway is known for its role in regulating responses to nutrient or energy starvation and environmental stresses [70].

### *V. cambriense* nutrient sources

Based on the genome, *V. cambriense* is able to use acetoacetate, ammonia, arabinose, ethanol, fructose, glucose, glycerol, glycogen, lactose, mannose, melibiose, ribose, sialic acid, starch, sucrose, and xylose as nutrient sources. It is interesting to note that cultured representatives of *V. cambriense* have not been shown to use either starch or xylose [66]. Fructose, glucose, glycerol, glycogen, lactose, mannose, melibiose, starch, sucrose, and xylose can all be directed to glycolysis, for which the complete pathway was identified. Unlike other members of the community, including *A. urogenitalis* (the other Actinomycetaceae), *V. cambriense* does not have the enzymes for the Entner-Doudoroff pathway (Figure 2).

The genome encodes several neuraminidases (also known as sialidases), suggesting that *V. cambriense* is able to cleave various sialic acid species from host-derived substrates. Sialic acids are found terminally bound to cell-surface glycoproteins, human breast milk glycans, and mucins, but are only accessible by microbes once they have been cleaved from their substrate [71-73]. The genome also contains a sialic acid transporter and the enzymes necessary for converting the predominant form of sialic acid found in humans, *N-*acetylneuraminate, to d-fructose-6-phosphate, which can in turn be fed into glycolysis. Thus, unlike many bacterial species, *V. cambriense* is probably able both to liberate sialic acids and to make use of them as a nutrient source. However, the genes for the pathway that converts *N*-acetylneuraminate to CMP-*N*-acetylneuraminate are not present, making it unlikely that *V. cambriense* can coat its outer membrane with sialic acids as other species do to evade the host immune system [74].

The pathway for degrading *N*-acetylglucosamine, a derivative of glucose that is found in chitin, fungal, and prokaryote cell walls, was identified. A near-complete pathway was identified for converting myo-inositol to dihydroxyacetone phosphate, acetyl-CoA, and CO_2_ (while reducing two NAD^+^ to NADH). However, no pathways for butyrate or cellulose metabolism could be found, nor genes involved in mucin protein degradation, nor evidence for CO_2_ fixation (no evidence could be found for the presence of pyruvate formate-lyase).

### *V. cambriense* pentose phosphate pathway

The *V. cambriense* genome is missing both glucose-6-phosphate dehydrogenase and 6-phosphogluconolactonase, primary components of the oxidative branch of the pentose phosphate pathway. However, as is expected for a facultative anaerobe, the non-oxidative pathway is complete. The presence of a gluconate transporter and ribose transport system suggests that the pentose phosphate pathway could use these precursors to produce D-glyceraldehyde-3-phosphate, which in turn could enter the methylerythritol phosphate pathway and create isopentenyl diphosphate and dimethylallyl diphosphate (fundamental units of isoprenoid biosynthesis), and geranyl diphosphate (a crucial precursor of menaquinone biosynthesis). Likewise, d-fructose-6-phosphate produced in the pentose phosphate pathway can be fed into glycolysis or can participate in the synthesis of UDP-N-acetyl-d-glucosamine, a necessary precursor of cell wall peptidoglycan. Another branch from d-erythrose-4-phosphate in the pentose phosphate pathway could lead to the biosynthesis of chorismate, an important biochemical intermediate. Overall, these pathways indicate the sources of several key metabolic precursors.

### *V. cambriense* fermentation and degradation reactions

The presence of lactate dehydrogenase suggests that pyruvate produced from glycolysis can be fermented to lactate; however, consistent with isolate metabolic data, no pathways were found to consume lactate [66]. We identified that the α, β, and γ subunits of pyruvate-ferredoxin oxidoreductase (EC: 1.2.7.1) are successively encoded on the *V. cambriense* genome (the δ subunit could not be identified). This enzyme uses an oxidized ferredoxin to ferment pyruvate and produce H^+^, CO_2_, and acetyl-CoA, which can subsequently be converted into either acetate or ethanol. Although the directionality of ethanol interconversion is difficult to infer from protein sequences alone, it is possible that the reverse of the ethanol fermentation reaction can occur without additional enzymes, resulting in the formation of acetyl-CoA from ethanol with a gain of two NADH. Additionally, the pathway for acetoacetate degradation through the intermediate acetoacetyl-CoA, which has a net yield of one molecule of acetyl-CoA, also exists. In *E. coli*, acetoacetate can function as a total source of carbon and energy through this pathway [75], and this may be the case for *V. cambriense*.

### *V. cambriense* tricarboxylic acid cycle

There is strong evidence for a tricarboxylic acid (TCA) cycle and respiratory capacity in the *V. cambriense* genome. Two of the three components of the pyruvate dehydrogenase complex are encoded by the genome (the E1 component could not be identified). If functional, *V. cambriense* could convert pyruvate into acetyl-CoA (which could be used in the TCA cycle) using either this enzyme or pyruvate-ferredoxin oxidoreductase. The genome encodes the enzymes for converting acetyl-CoA into succinate. The TCA cycle can then continue by converting succinate into fumarate using succinate dehydrogenase/fumarate reductase (EC: 1.3.99.1). Four subunits are required for this enzyme, but only three were identified (the iron-sulfur subunit, flavoprotein subunit, and cytochrome b556 subunit are co-localized on the genome). No evidence could be found for the membrane anchor subunit, which may be due to the presence of a small scaffolding gap in this region of the genome, or may indicate the existence of a divergent form of this enzyme. The presence of fumarate lyase provides a way for fumarate to be converted into malate, thus continuing the cycle. The form of malate dehydrogenase that converts malate into oxaloacetate by reducing NAD^+^ to NADH and H^+^ is present, but not the form of the enzyme that uses a quinone. Taken together, *V. cambriense* encodes a complete TCA cycle.

### *V. cambriense* anaerobic respiration

Several components of an anaerobic respiratory chain were identified. The large and small subunits of the hydrogenase enzyme (containing iron-sulfur clusters, EC: 1.12.99.6) and all 14 subunits of NADH dehydrogenase (EC: 1.6.5.3) were identified, indicating that both hydrogen acquired from the environment and NADH produced by glycolysis, the TCA cycle, and substrate degradation reactions (ethanol degradation, for example) can be used as electron donors during anaerobic respiration.

Identification of fumarate reductase (EC: 1.3.99.1), nitrate reductase (EC: 1.7.99.4), and dimethyl sulfoxide (DMSO) reductase (EC: 1.8.5.3), suggests that fumarate, nitrate, and DMSO can all be used as terminal electron acceptors. Phylogenetic analysis of the nitrate reductase and DMSO reductase catalytic subunits supports the functional roles of these genes (Additional file 2: Figure S4). Furthermore, owing to the presence of a TAT signal sequence in the *V. cambriense* nitrate reductase, the active site is located on the outside of the cytoplasmic membrane, as is common in Archaea [76]. Also, the five signature residues suggested as being involved in nitrite and nitrate binding are conserved [76]. We also identified genes encoding a nitrate-nitrite transporter, but no other reactions that produce or consume nitrate or nitrite (the organism does not fix nitrogen, for example). No reactions for forming DMSO, or transporters for DMSO could be identified. The nitrite resulting from nitrate reduction could be further reduced by several other community members, several of which have the capacity to further reduce nitric oxide to nitrous oxide (*Escherichia coli - strain A, Staphylococcus sp., Veillonella dispar,* and *Veillonella sp. - species A*), although no species is predicted to be able to reduce nitrous oxide (Table 3).

As is common in gram-positive bacteria, *V. cambriense* does not have the genes required for the formation of ubiquinones. However, a near-complete pathway is present for the biosynthesis of menaquinones, electron mediators essential during fumarate, DMSO, and nitrate reduction [77]. All subunits of the F-type H^+^-transporting ATPase were identified, indicating that *V. cambriense* is able to produce ATP from the generated proton gradient.

As noted, *V. cambriense* is not capable of aerobic respiration. However, both subunits of the cytochrome *bd* complex were identified. This cytochrome along with cysteine synthase and superoxide dismutase (also identified) can protect against oxidative stress and contribute to limited oxygen tolerance [61,78]. Consistent with cultured strains, no evidence was found for catalase production [27,66].

### Other metabolic pathways found in *V. cambriense*

The genome contains complete amino acid biosynthesis and degradation pathways and all 20 aminoacyl tRNA synthetase genes. Complete pathways were found for riboflavin (vitamin B2) and vitamin K2 biosynthesis. The pathway for the synthesis of folate (vitamin B6) is incomplete; however, some crucial and unique enzymes to the pathway were identified, suggesting that this organism may also be able to synthesize this vitamin (approximately 30% of ORFs do not have a predicted function and could be responsible for this and other pathways). The combination of these functions indicates that *V. cambriense* may exist symbiotically with its human host under certain conditions.

### Abundance of *Varibaculum* in the healthy adult human microbiota

We searched data from the HMP, surveying the V35 region of the 16S rRNA gene in order to assess the abundance and distribution of *Varibaculum* in the human microbiota (Additional file 2: Figure S5) [57]. Out of the 5,000 samples taken from 235 healthy human subjects, only 90 had hits for *Varibaculum* (0% oral, 0.31% stool, 1.42% nasal, 3.92% skin, and 10.56% vaginal samples encompassing 24.68% of subjects). In only 29 of these (from 25 different individuals) was the relative abundance of *Varibaculum* greater than 0.05%, while this genus never represented more than 2.5% of any sample. On average, the most abundant organism in communities studied by the HMP represent 14.42% of the total community (standard deviation of 14.11%), and in communities with *Varibaculum*, the most abundant organism represented 20.48% (standard deviation 10.96%). *Varibaculum* was most abundant in samples from the antecubital fossa (skin) and the vagina. Only one stool sample had hits for *Varibaculum*, where it represented only 0.02% relative abundance. Although *Varibaculum* is not uncommon, it is never a dominant community member in the large, healthy, adult population surveyed by the HMP.

### Comparative genomics of *V. cambriense*

Comparing the genome for *V. cambriense* with available genomes for members of the family *Actinomycetaceae* revealed few unique genes, most of which are phage-associated or are not annotated. Several of these unique genes corresponded with folate, butanoate, and benzoate metabolism (among other pathways), but no complete pathways could be established from this set. However, there is considerable metabolic variation within the *Actinomycetaceae* (Figure 2). Only members of the genus *Mobiluncus* are motile, encoding genes for both flagella and chemotaxis. Nitrate reductase is common in the family, but not encoded by *Actinomyces coleocanis, Actinomyces graevenitzii, Arcanobacterium haemolyticum,* some strains of *Mobiluncus curtisii,* nor *Mobiluncus mulieris,* while nitrite reductase and nitric oxide reductase are found only in the *Actinomyces*. DMSO reductase is found only in *Mobiluncus curtisii*, *Actinomyces urogenitalis, Arcanobacterium haemolyticum,* and *V. cambriense.* Taken together, the *Actinomycetaceae* rely on several different terminal electron acceptors for anaerobic respiration.

Although genes were identified for riboflavin biosynthesis in *V. cambriense*, these genes are not common in the *Actinomycetaceae*. Several species of *Actinomyces* and members of the microbial community of the premature infant in our study (but not the *A. urogenitalis* genome reconstructed from the community) encode the genes for trehalose biosynthesis, suggesting a possible interrelationship between these species and *V. cambriense*, which only encodes the genes for trehalose degradation (Figure 2). In the *Actinomycetaceae*, pyruvate-ferredoxin oxidoreductase, mentioned previously for its importance in converting pyruvate into acetyl-CoA, is found only in *V. cambriense* and members of *Mobiluncus*. Neuraminidases, which are required for cleaving sialic acids from glycoproteins, human breast milk glycans, and intestinal mucins, are distributed throughout the *Actinomycetaceae*. However, most members of the family are missing transporters for sialic acids, although the presence of the enzymes for their degradation suggests that a currently uncharacterized transporter exists for these species (Additional file 5). Members of the *Actinomycetaceae* and the microbial community in the gut of this infant engage in diverse metabolisms. Clustering based on select metabolic characters shows that *V. cambriense* is more metabolically similar to other *Actinomycetaceae* than to other members identified in the gut of this infant, despite significant metabolic overlap among community members (Figure 2).

## Discussion

Genome reconstructions facilitated prediction of the metabolic roles of individual bacterial members in the context of their community. Applied to the gut microbiome of a premature male infant, the time series abundance information also provided by this method revealed strain-specific dynamics during the third week of life. Comparison of reconstructed genomes to the genomes of isolate strains revealed genomic novelty, even among members of this relatively simple microbial community. However, overall similarities between most reconstructed and reference genomes validated the *de novo* genome binning strategy.

Metabolic analysis revealed a community consisting of facultative anaerobes and obligate (fermentative) anaerobes. The facultative anaerobe *Escherichia coli* was initially dominant in the time series, but was replaced by obligate anaerobes (*Streptococcus anginosus* and *Clostridium butyricum*) during a switch to a community dominated by fermentation-based metabolism. This shift emphasizes the instability of the infant gut in terms of both membership and metabolism, and could be the result of several factors previously observed in the human gut. For example, dominance of species from the family *Enterobacteriaceae* (including *E. coli*) has been associated with either high oxygen levels or the availability of nitrate (a natural byproduct of the host immune response) [79]. Thus, this shift could be the result of decreased availability of either nitrate or oxygen, either of which could be depleted by *E. coli* during respiration. Decreased inflammation could also decrease available nitrate and decrease the competitive advantage of *E. coli* over obligate fermenters [80]. In terms of the gut environment, succession during early life is driven by the presence of oxygen [81], and replacement of *E. coli* with obligate fermenters is suggestive of a decrease in oxygen; however, we cannot rule out the hypothesis that this shift in relative abundance is stochastic. Regardless of the mechanism, this represents a dramatic shift in community composition with the potential to affect host metabolism.

Although the abundance of *S. anginosus* and *E. coli* appeared to equilibrate by the end of the time series, the drop in abundance of *C. butyricum* after its initial spike suggests a potential competition between the two obligate anaerobes. Interestingly, *S. anginosus* and *C. butyricum* have different clinical presentations. *S. anginosus* is commonly observed in association with abscess formation [82], while *C. butyricum* is usually considered a beneficial, butyrate-producing commensal [83]. In this case, the microbe more likely to be beneficial in the gut environment is outcompeted.

To explore the potential role of a species not commonly observed or previously characterized from the human gut, we manually curated and metabolically analyzed the genome of *Varibaculum cambriense* (strain Dora), resulting in the first genomic sampling of a member of the genus *Varibaculum*. Strains of *V. cambriense* isolated from human cerebral and skin abscesses, intrauterine contraceptive devices, and the human vagina have been used to show that it is an anaerobic, catalase-negative, gram-positive, diphtheroid-shaped bacterium [27,66]. However, genome analysis revealed additional insight into the metabolic potential of this organism and informed which substrates *V. cambriense* may use for anaerobic respiration. *V. cambriense* is metabolically similar to common gut inhabitants, and is predicted to use various carbon sources, respire anaerobically (using fumarate, nitrate, and DMSO), and produce lactate during fermentation.

Several community members (including *V. cambriense*) are predicted to use myo-inositol as a nutrient source. This is interesting because myo-inositol plays a role in eukaryotic cell messaging and is found in breast milk and infant formula, although it is generally not found in solutions used for intravenous feeding [84]. Inositol has been shown to benefit premature infants with respiratory ailments [85], suggesting that microbial degradation of this compound would decrease its health benefits. In contrast, the potential for *V. cambriense* to produce essential vitamins suggests a beneficial contribution by this organism to its human host.

Although this infant was fed fortified breast milk (an abundant source of glycan-bound sialic acids) during the time period studied, only *V. cambriense*, *Streptococcus sp.*, and *Streptococcus parasanguinis*, all low-abundance members of the gut community, have neuraminidases (enzymes that cleave sialic acids) and enzymes for sialic acid degradation. Although the *E. coli* genome does not encode a neuraminidase, it has a sialic acid transporter and degradation machinery. Thus, low-abundance community members may be making sialic acids available to *E. coli*. It has been shown that some pathogenic species incapable of accessing bound sialic acids are able to make use of sialic acids cleaved by commensal organisms to promote their own growth [86].

The ability of *V. cambriense* to degrade, but not produce, trehalose suggests a possible dependency on other members of the microbial community able to produce this disaccharide. Furthering community interrelationships, nitrite produced by *V. cambriense* during anaerobic respiration can by further utilized by community members capable of nitrite and nitric oxide reduction.

## Conclusions

This study underlines the higher resolution insight that can be obtained using genome-centric metagenomic approaches. Strain-resolved community dynamics revealed two phases in colonization during the third week of life of a premature infant. The phases were distinguishable based on the dominance of either respiratory or fermentation-based metabolism. Comparison of *V. cambriense* with other members of the microbial community revealed similarities with traditional gut inhabitants, while comparisons with other members of the family *Actinomycetaceae* illustrate how the metabolic diversity of this family could mislead species-level functional analysis based on 16S rRNA gene sequencing. Analysis of reconstructed genomes enabled strain-specific metabolic potential to be determined for the microbial community, and suggested potential community interdependencies.

### Availability of supporting data

All data have been made publically available and can be accessed through SRA [SRA: SRS470507], DDBJ/EMBL/GenBank [GenBank: AZMA00000000, AZMB00000000, AZMC00000000, AZMD00000000, AZME00000000, AZMF00000000, AZMG00000000, AZMH00000000, AZMI00000000, AZMJ00000000, AZMK00000000, AZML00000000, AZMM00000000], and ggKbase [59]. DDBJ/EMBL/GenBank versions [GenBank: AZMA01000000, AZMB01000000, AZMC01000000, AZMD01000000, AZME01000000, AZMF01000000, AZMG01000000, AZMH01000000, AZMI01000000, AZMJ01000000, AZMK01000000, AZML01000000, AZMM01000000] are described here.

## Abbreviations

BLAST: Basic local alignment search tool; DMSO: Dimethyl sulfoxide; DOL: Day of life; ESOM: Emergent self-organizing map; HMP: Human Microbiome Project; ORF: Open reading frame; OTU: Operational taxonomic unit; RDP: Ribosomal database project; TCA: Tricarboxylic acid; TMAO: Trimethylamine *N*-oxide; TPN: Total parenteral nutrition.

## Competing interests

The authors declare that they have no competing interests.

## Authors’ contributions

CTB carried out the binning and genome curation, functional, time series abundance and phylogenetic analysis, and drafted the manuscript. IS assembled the sequence data and contributed to the bioinformatics analysis. BCT performed the functional annotation. CJC assisted with biochemical analysis. MJM oversaw sample collection and handled medical aspects of the research. MJM and JFB designed and oversaw the study and data analysis. All authors contributed to manuscript preparation. All authors read and approved the final manuscript.

## Supplementary Material

Additional file 1 ESOM data. Click here for file

Additional file 2 Supplemental figures and tables. Click here for file

Additional file 3 Single copy genes in reconstructed genomes. Click here for file

Additional file 4 ***V. cambriense *** **pathway tools data.** Click here for file

Additional file 5 KEGG annotations. Click here for file

Additional file 6 EMIRGE reconstructed 16S rRNA gene sequences. Click here for file

Additional file 7 Metabolic features for comparative genomics. Click here for file
