# Supplementary material for: Genome resolved analysis of a premature infant gut microbial community reveals a Varibaculum cambriense genome and a shift towards fermentation-based metabolism during the third week of life
Source: Microbiome. 2013 Dec 17;1:30. doi: 10.1186/2049-2618-1-30 (PMC4177395; doi:10.1186/2049-2618-1-30)
Supplement: Additional file 2 — Supplemental figures and tables. [file 2049-2618-1-30-S2.pdf]

Supplemental Figures

Supplemental Figure 1 - ESOM Showing Best Blast Hit of Scaffolds

ESOM showing the clustering of *de novo* assembled metagenomic data. Each point represents a fragment of an assembled scaffold. Clustering of data points is based on the time series abundance pattern of each assembled scaffold. Dark lines between clusters show definitive separation of genome bins. Data points are colored based on the best BLAST hit of each scaffold compared against the NCBI NT database (coloring is independent of the assembly and binning).

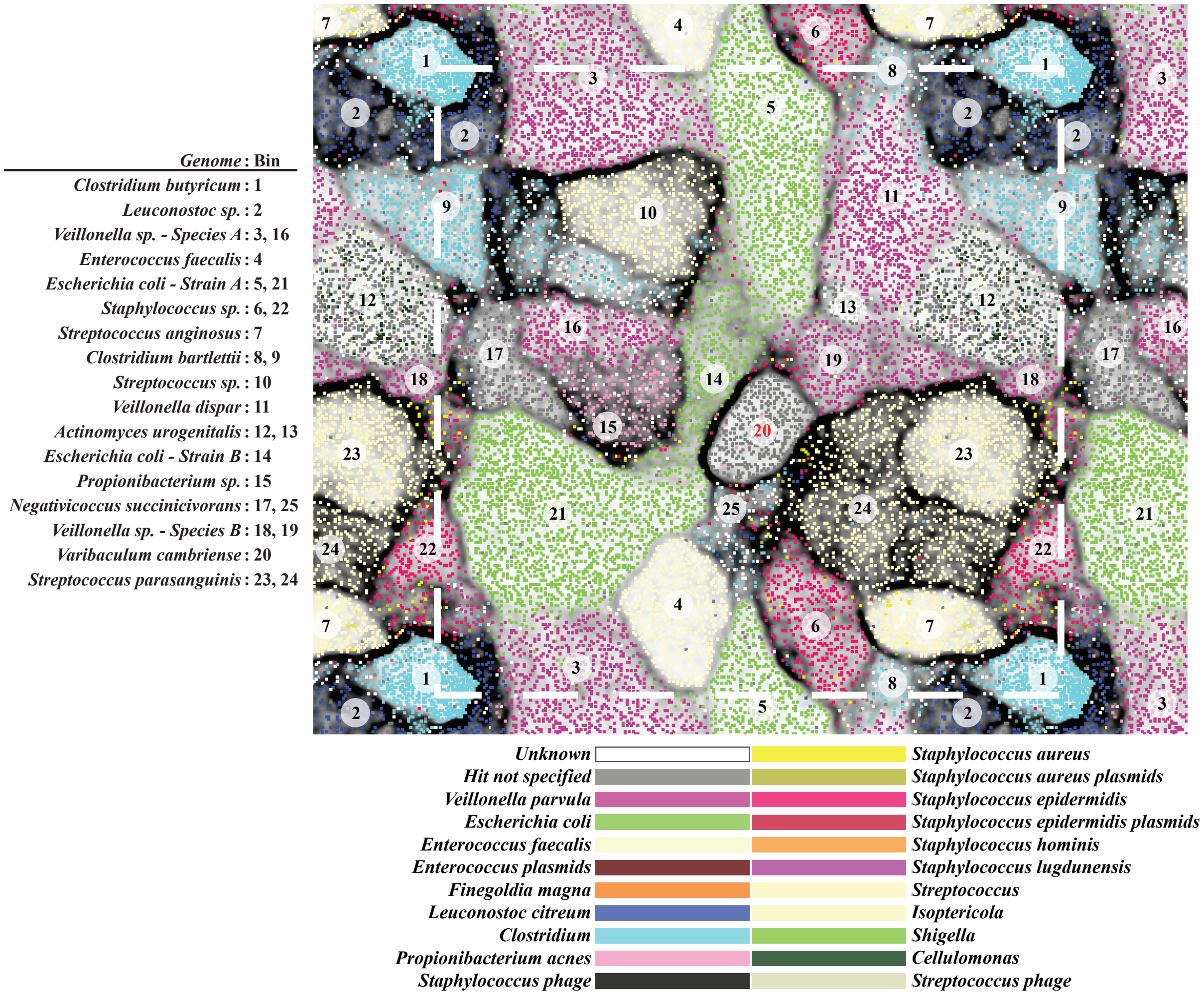

Supplemental Figure 2 - Phylogeny of EMIRGE 16S rRNA Genes

Reconstructed 16S rRNA gene sequences were aligned along with sequences from their closest relatives in addition to species previously identified in the infant gut. Sequences were aligned with PyNAST using the GreenGenes {DeSantis:2006ii} alignment of OTUs classified at 97% sequence similarity as a template. The phylogenetic tree was constructed with FastTree2 using the generalized time-reversible model for nucleotide evolution and the CAT approximation. Local support values were calculated with the Shimodaira-Hasegawa test. The tree was rooted with the 16S sequence for *Halobacterium salinarum* and formatted using FigTree. Sequences reconstructed with EMIRGE from the microbial community are shown in blue, and reference sequences with an associated sequenced genome are shown in red.

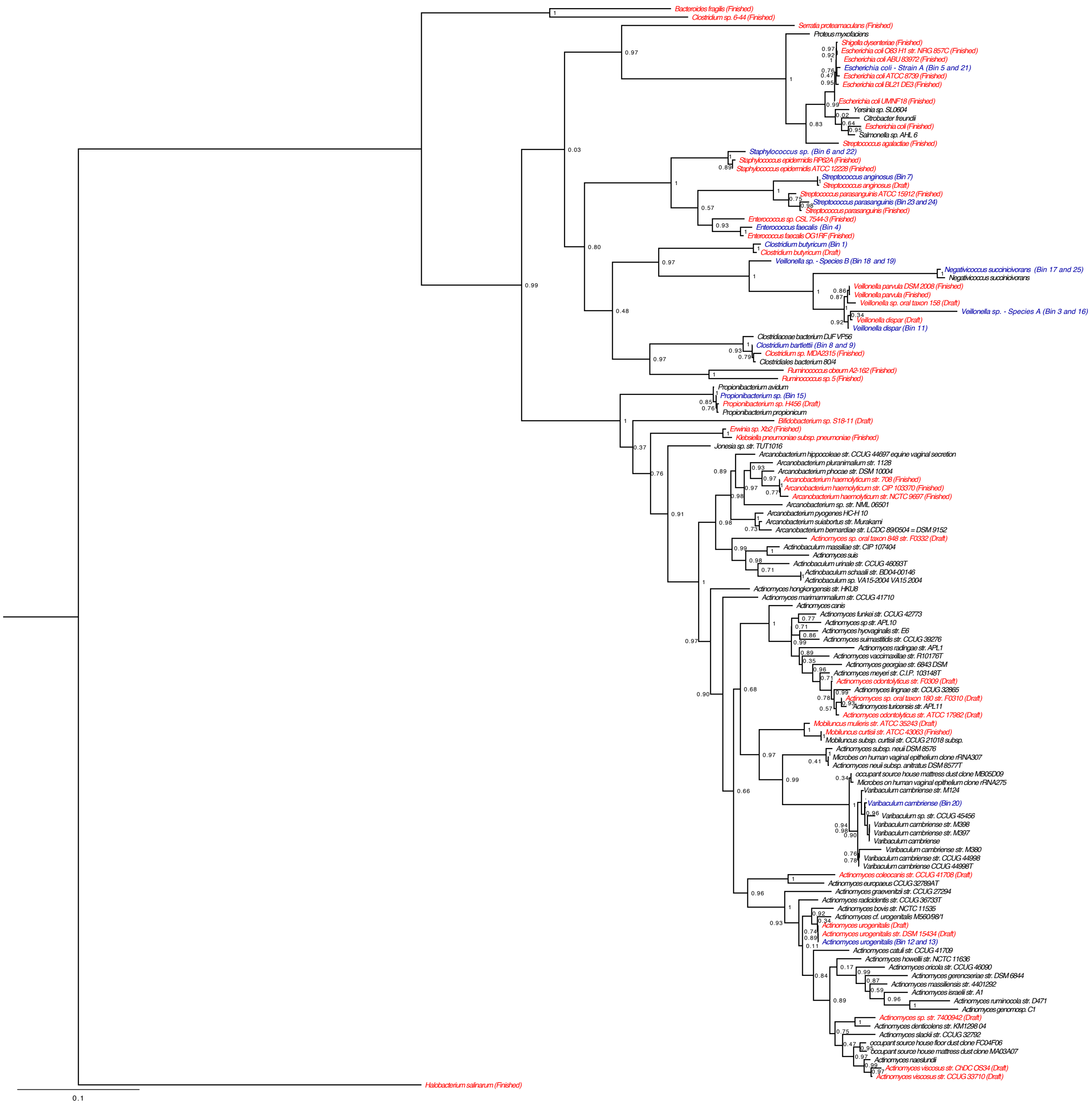

Supplemental Figure 3 - Phylogeny of Plasmid Replication Protein Genes

Plasmid novelty and diversity represented by plasmid replication proteins. Protein sequences were aligned using MUSCLE and the phylogenetic tree was constructed using FastTree2 with the Jones-Taylor-Thornton model of amino acid evolution and the CAT approximation. Local support values were calculated with the Shimodaira-Hasegawa test. Sequences from plasmids reconstructed from the microbial community are shown in red.

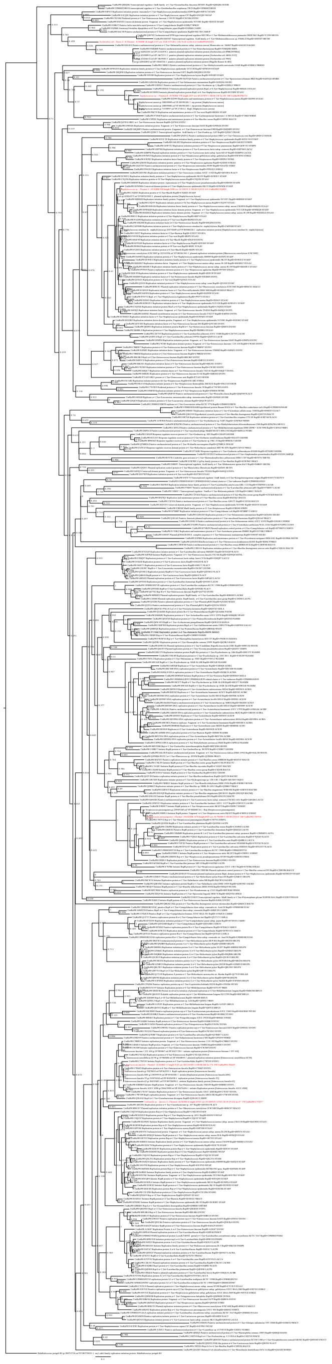

Supplemental Figure 4 - Phylogenetic Analysis of the Catalytic Subunits of the Nitrate Reductase and Dimethyl Dulfoxide (DMSO) Reductase Superfamilies

Genes newly assigned to the nitrate reductase and DMSO reductase superfamilies (this study) are shown in red.

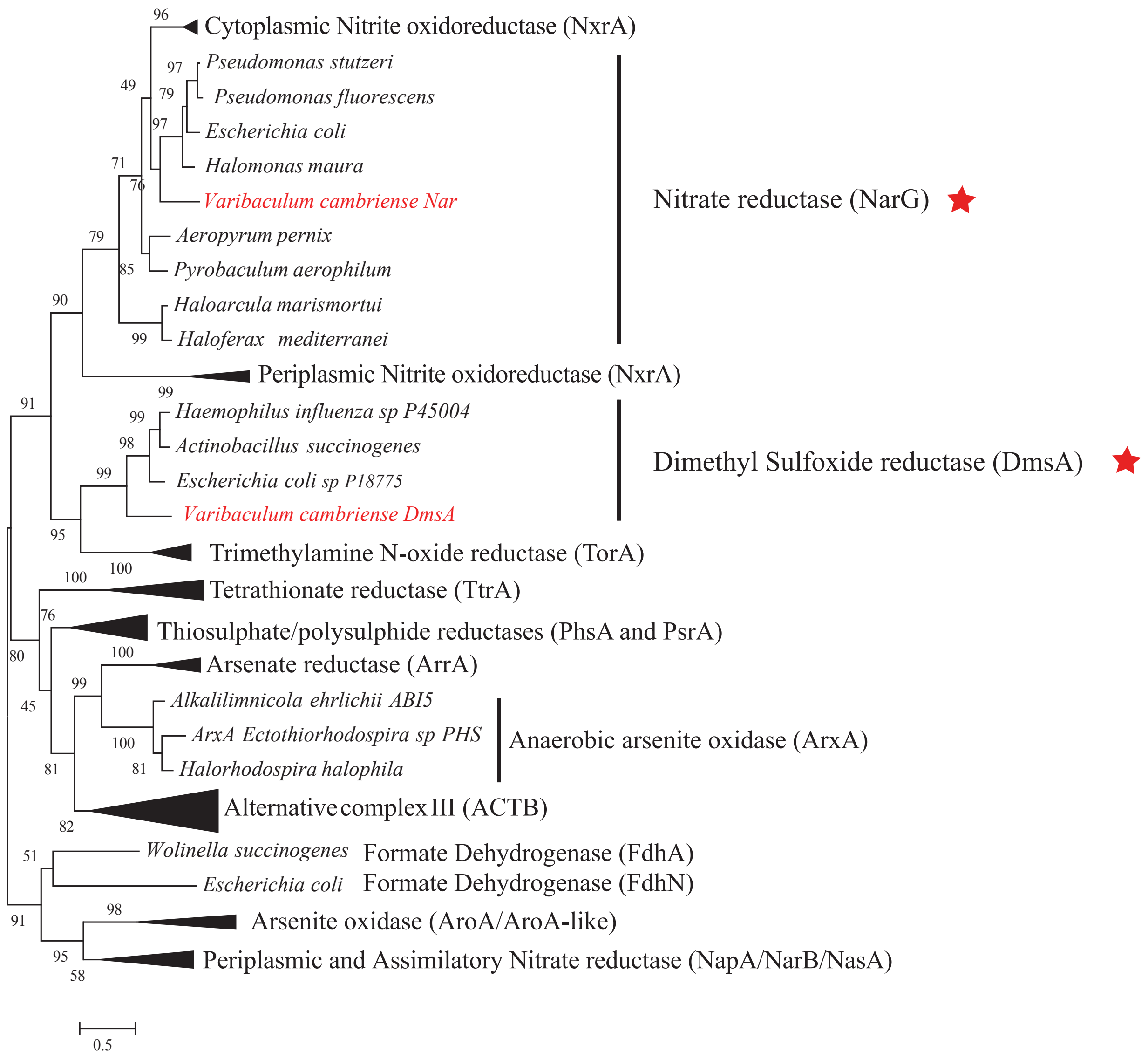

Supplemental Figure 5 - Relative Abundance of *Varibaculum* in Human Microbiome Project Data

The relative abundance of *Varibaculum* was determined for each sample analyzed by the Human Microbiome Project. These samples were characterized by sequencing the V13 (a) and V35 (b) regions of the 16S rRNA gene.

a)

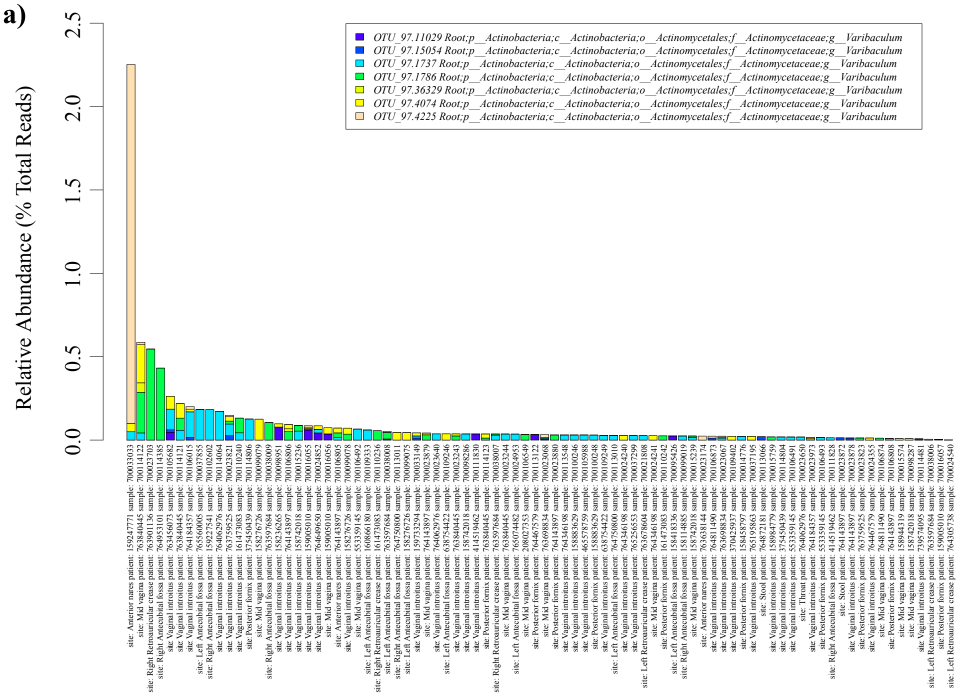

b)

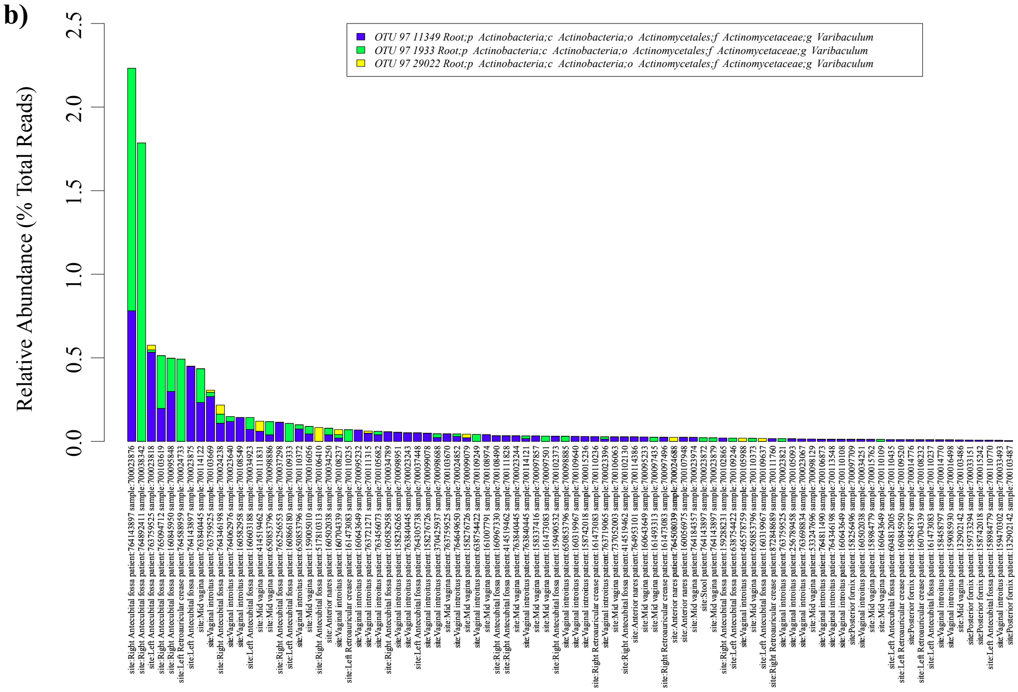

Supplemental Tables

Supplemental Table 1 - ESOM bins.

Assembly and classification of scaffolds clustered into bins using an ESOM. Each bin was compared to a sequenced microbial genome by identifying orthologs. Orthologs were identified by finding reciprocral best hits from pair-wise protein sequence searches between each bin and the genome of a sequenced relative.

Genome	Bin	Phylum	Bin Size (bp)	Bin Coverage	N50	Number of ORFs	Predicted % Single Copy Genes	Closest Relative with Sequenced Genome	Closest Relative Genome Size (bp)	% ORFs Orthologous to Reference	Average % AA ID of Orthologs
Actinomyces urogenitalis	13	Actinobacteria	116,128.00	1,982.48	2,487	156	3.70	*Actinomyces urogenitalis DSM 15434 uid55459*	2,702,812	51.92	94.34
Escherichia coli - Strain A	5	Proteobacteria	1,704,074.00	1,829.54	3,509	2,503	11.11	*Escherichia coli S88 uid62979*	5,032,268	55.21	95.00
Streptococcus anginosus	7	Firmicutes	2,108,491.00	1,714.41	537,826	2,252	100.00	*Streptococcus anginosus 1 2 62CV uid62163*	1,821,055	67.67	95.22
Escherichia coli - Strain A	21	Proteobacteria	3,962,046.00	1,563.00	6,305	4,906	37.04	*Escherichia coli S88 uid62979*	5,032,268	60.44	97.38
Veillonella dispar	11	Firmicutes	2,445,194.00	1,331.29	53,688	2,693	100.00	*Veillonella dispar ATCC 17748 uid55331*	2,118,767	60.04	91.57
Clostridium butyricum	1	Firmicutes	4,350,784.00	724.59	103,127	4,094	100.00	*Clostridium butyricum 5521 uid54843*	4,540,699	73.47	96.52
Actinomyces urogenitalis	12	Actinobacteria	2,491,825.00	704.73	3,400	2,883	92.59	*Actinomyces urogenitalis DSM 15434 uid55459*	2,702,812	69.48	98.64
Veillonella sp. - Species A	3	Firmicutes	2,173,194.00	373.08	4,666	2,727	37.04	*Veillonella oral taxon 158 F0412 uid61047*	2,176,752	56.33	92.59
Enterococcus faecalis	4	Firmicutes	2,960,721.00	255.03	235,714	2,906	100.00	*Enterococcus faecalis OG1RF uid54927*	2,739,625	79.11	98.82
Veillonella sp. - Species A	16	Firmicutes	539,200.00	230.21	3,983	756	7.41	*Veillonella dispar ATCC 17748 uid55331*	2,118,767	64.29	92.45
Escherichia coli - Strain B	14	Proteobacteria	641,540.00	207.93	4,041	883	3.70	*Escherichia coli S88 uid62979*	5,032,268	52.66	82.25
Veillonella sp. - Species B	18	Firmicutes	109,076.00	158.19	1,316	174	22.22	*Veillonella dispar ATCC 17748 uid55331*	2,118,767	79.31	91.53
Varibaculum cambriense	**20**	**Actinobacteria**	**2,247,641.00**	**122.51**	**240,417**	**1,954**	**100.00**	***Mobiluncus mulieris ATCC 35239 uid52345***	**2,533,633**	**58.19**	**53.79**
Staphylococcus sp.	22	Firmicutes	490,354.00	75.43	7,348	564	18.52	*Staphylococcus epidermidis ATCC 12228 uid57861*	2,499,279	74.82	94.85
Veillonella sp. - Species B	19	Firmicutes	530,104.00	75.38	2,283	705	7.41	*Veillonella dispar ATCC 17748 uid55331*	2,118,767	63.12	85.29
Clostridium bartlettii	9	Firmicutes	2,444,327.00	60.98	12,184	2,342	92.59	*Clostridium bartlettii DSM 16795 uid54809*	2,972,256	89.03	98.53
Clostridium bartlettii	8	Firmicutes	241,119.00	56.99	17,901	291	0.00	*Clostridium bartlettii DSM 16795 uid54809*	2,972,256	51.20	64.62
Negativicoccus succinicivorans	17	Negativicoccus	1,341,981.00	33.59	17,543	1,448	100.00	*Bacillus coagulans 36D1 uid54335*	3,552,226	50.28	43.78
Negativicoccus succinicivorans	25	Negativicoccus	166,917.00	32.97	1,766	238	3.70	*Bacillus coagulans 36D1 uid54335*	3,552,226	23.11	36.62
Streptococcus parasanguinis	23	Firmicutes	2,017,810.00	32.00	5,231	2,366	77.78	*Streptococcus parasanguinis ATCC 15912 uid49313*	2,153,652	68.43	94.96
Staphylococcus sp.	6	Firmicutes	1,039,864.00	31.23	10,033	1,102	18.52	*Staphylococcus epidermidis ATCC 12228 uid57861*	2,499,279	84.94	98.43
Propionibacterium sp.	15	Propionibacterium	336,576.00	22.16	1,117	565	18.52	*Propionibacterium 5 U 42AFAA uid75117*	2,532,807	46.37	82.40
Leuconostoc sp.	2	Firmicutes	566,369.00	19.44	1,603	874	25.93	*Leuconostoc citreum KM20 uid58481*	1,796,284	66.70	96.03
Streptococcus sp.	10	Firmicutes	1,915,777.00	16.17	11,008	2,114	55.56	*Streptococcus M334 uid62529*	2,207,013	67.64	93.88
Streptococcus parasanguinis	24	Firmicutes	829,730.00	8.19	1,343	1,318	14.81	*Streptococcus parasanguinis ATCC 15912 uid49313*	2,153,652	59.03	70.58

Supplemental Table 2 - Absolute abundance of reconstructed genomes.

Percent of total reads used in the metagenome assembly that could be mapped to reconstructed genomes at each time point and as a total.

Genome	DOL 14	DOL 15	DOL 15	DOL 18	DOL 18	DOL 19	DOL 19	DOL 20	DOL 20	Total
Escherichia coli - Strain A	58.860	44.566	36.751	29.765	17.912	22.821	26.512	33.088	36.597	33.447
Unassembled and Unbinned	18.582	16.636	16.092	13.071	10.374	12.357	12.489	13.682	14.552	14.012
Streptococcus anginosus	0.044	0.197	0.206	20.935	15.970	21.174	19.276	20.431	15.224	13.016
Veillonella dispar	4.596	10.642	13.491	14.499	7.331	15.952	17.137	12.572	11.219	11.722
Clostridium butyricum	0.092	4.917	9.954	5.376	39.871	14.426	9.686	3.120	4.781	11.352
Actinomyces urogenitalis	2.283	6.018	9.913	8.936	3.681	7.049	8.455	9.991	9.811	7.135
Veillonella sp. - Species A	7.836	6.820	6.841	1.621	1.076	1.247	1.444	1.949	2.306	3.298
Enterococcus faecalis	4.844	4.937	3.321	2.175	1.443	2.491	2.318	1.799	1.609	2.719
Varibaculum cambriense	0.071	1.638	0.387	1.942	1.067	0.579	0.887	1.143	0.898	0.992
Clostridium bartlettii	0.409	0.794	0.972	0.582	0.458	0.760	0.618	0.406	0.405	0.586
Escherichia coli - Strain B	0.608	0.717	0.295	0.143	0.159	0.131	0.121	0.204	0.178	0.281
Veillonella sp. - Species B	0.111	0.181	0.205	0.240	0.104	0.201	0.220	0.328	0.305	0.206
Streptococcus parasanguinis	0.006	0.016	0.016	0.053	0.025	0.133	0.180	0.497	1.014	0.205
Negativicoccus succinicivorans	0.457	0.324	0.290	0.111	0.077	0.113	0.143	0.087	0.091	0.182
Staphylococcus sp.	0.114	0.166	0.116	0.173	0.140	0.193	0.108	0.223	0.252	0.164
Escherichia coli - Strain A / B - Plasmid	0.352	0.304	0.159	0.114	0.075	0.118	0.107	0.117	0.133	0.162
Streptococcus sp.	0.026	0.419	0.429	0.022	0.012	0.041	0.019	0.043	0.093	0.112
Veillonella sp. - Species A - Phage	0.102	0.096	0.102	0.054	0.034	0.042	0.066	0.101	0.204	0.086
Escherichia coli - Strain A - Phage	0.139	0.107	0.084	0.069	0.048	0.057	0.064	0.068	0.077	0.078
Veillonella sp. - Species A - Plasmid	0.242	0.226	0.139	0.009	0.005	0.008	0.006	0.006	0.007	0.069
Veillonella sp. - Species B - Phage	0.038	0.036	0.035	0.035	0.021	0.027	0.048	0.068	0.140	0.048
Leuconostoc sp.	0.028	0.048	0.093	0.017	0.078	0.019	0.035	0.006	0.031	0.040
Enterococcus faecalis - Plasmid	0.060	0.106	0.037	0.025	0.016	0.029	0.025	0.025	0.019	0.038
Propionibacterium sp.	0.088	0.065	0.046	0.011	0.009	0.007	0.007	0.008	0.010	0.027
Actinomyces urogenitalis - Phage	0.006	0.018	0.023	0.019	0.010	0.017	0.022	0.023	0.022	0.017
Streptococcus parasanguinis - Plasmid	0.000	0.000	0.000	0.000	0.000	0.002	0.003	0.010	0.016	0.003
Staphylococcus sp. - Plasmid B	0.002	0.003	0.001	0.001	0.001	0.002	0.001	0.003	0.004	0.002
Staphylococcus sp. - Plasmid A	0.002	0.003	0.001	0.001	0.001	0.001	0.001	0.002	0.003	0.002
Staphylococcus sp. - Phage	0.002	0.001	0.001	0.002	0.002	0.001	0.001	0.001	0.001	0.001

Supplemental Table 3 - Coverage of reconstructed genomes.

Coverage of reconstructed genomes at each time point and as a total.

Genome	DOL 14	DOL 15	DOL 15	DOL 18	DOL 18	DOL 19	DOL 19	DOL 20	DOL 20	Total
Escherichia coli - Strain A / B - Plasmid	3,310.53	3,006.34	1,139.91	1,148.89	958.28	1,041.67	1,026.07	1,127.07	1,150.90	13,909.65
Escherichia coli - Strain A - Phage	1,074.53	866.54	496.74	574.85	506.76	413.92	504.95	541.55	550.77	5,530.63
Enterococcus faecalis - Plasmid	347.35	645.56	165.90	157.22	126.40	158.52	147.64	147.28	101.04	1,996.92
Streptococcus anginosus	0.63	2.97	2.26	323.78	312.81	285.34	282.81	301.64	202.17	1,714.41
Escherichia coli - Strain A	315.09	250.71	150.33	171.42	130.65	114.52	144.85	181.91	180.98	1,640.47
Actinomyces urogenitalis - Phage	57.29	186.51	177.61	210.95	142.71	164.47	232.03	237.30	208.64	1,617.51
Veillonella dispar	56.97	138.64	127.79	193.36	123.83	185.37	216.81	160.05	128.47	1,331.29
Veillonella sp. - Species A - Phage	151.54	149.00	115.57	86.39	68.37	58.96	100.50	154.19	279.74	1,164.27
Veillonella sp. - Species B - Phage	69.35	69.17	48.50	69.94	52.17	46.84	88.93	128.20	237.07	810.17
Actinomyces urogenitalis	26.56	73.59	88.14	111.86	58.35	76.88	100.41	119.40	105.45	760.65
Clostridium butyricum	0.64	36.00	52.99	40.30	378.49	94.21	68.87	22.32	30.77	724.59
Veillonella sp. - Species A - Plasmid	154.11	151.29	67.74	5.89	4.57	4.57	3.76	4.02	3.95	399.91
Veillonella sp. - Species A	89.13	81.53	59.46	19.83	16.67	13.30	16.77	22.77	24.24	343.69
Enterococcus faecalis	49.59	53.11	25.98	23.96	20.13	23.91	24.22	18.92	15.22	255.03
Staphylococcus sp. - Plasmid B	27.47	40.68	12.85	12.42	19.30	20.04	14.18	31.35	39.36	217.65
Staphylococcus sp. - Plasmid A	28.46	31.18	7.79	11.22	12.01	15.54	17.04	23.88	33.56	180.68
Staphylococcus sp. - Phage	19.83	17.89	10.31	21.14	27.86	9.33	8.39	10.60	14.48	139.83
Escherichia coli - Strain B	29.11	36.07	10.78	7.36	10.39	5.87	5.91	10.01	7.85	123.35
Varibaculum cambriense	0.96	23.22	3.99	28.18	19.62	7.32	12.21	15.83	11.18	122.51
Streptococcus parasanguinis - Plasmid	0.01	0.04	0.09	1.70	1.16	7.64	10.92	33.18	50.03	104.78
Veillonella sp. - Species B	5.25	9.01	7.44	12.24	6.72	8.94	10.63	15.95	13.35	89.51
Clostridium bartlettii	4.62	9.42	8.38	7.07	7.04	8.04	7.12	4.71	4.22	60.62
Negativicoccus succinicivorans	9.18	6.84	4.45	2.41	2.10	2.12	2.93	1.79	1.69	33.52
Staphylococcus sp.	2.28	3.50	1.77	3.73	3.83	3.64	2.21	4.61	4.67	30.23
Propionibacterium sp.	7.95	6.18	3.16	1.09	1.05	0.58	0.60	0.73	0.82	22.16
Streptococcus parasanguinis	0.07	0.18	0.13	0.61	0.36	1.34	1.97	5.48	10.07	20.21
Leuconostoc sp.	1.52	2.69	3.82	0.96	5.72	0.95	1.93	0.31	1.54	19.44
Streptococcus sp.	0.42	6.97	5.18	0.38	0.26	0.61	0.31	0.70	1.35	16.17

Supplemental Table 4 - Relative abunance of reconstructed genomes.

Relative abundance of reconstructed genomes at each time point and as a total.

Genome	DOL 14	DOL 15	DOL 15	DOL 18	DOL 18	DOL 19	DOL 19	DOL 20	DOL 20	Total
Escherichia coli - Strain A / B - Plasmid	56.68	50.91	40.72	35.36	31.76	37.54	33.59	33.89	33.72	41.67
Escherichia coli - Strain A - Phage	18.40	14.68	17.75	17.69	16.79	14.92	16.53	16.28	16.13	16.57
Enterococcus faecalis - Plasmid	5.95	10.93	5.93	4.84	4.19	5.71	4.83	4.43	2.96	5.98
Streptococcus anginosus	0.01	0.05	0.08	9.97	10.37	10.28	9.26	9.07	5.92	5.14
Escherichia coli - Strain A	5.39	4.25	5.37	5.28	4.33	4.13	4.74	5.47	5.30	4.91
Actinomyces urogenitalis - Phage	0.98	3.16	6.35	6.49	4.73	5.93	7.60	7.14	6.11	4.85
Veillonella dispar	0.98	2.35	4.57	5.95	4.10	6.68	7.10	4.81	3.76	3.99
Veillonella sp. - Species A - Phage	2.59	2.52	4.13	2.66	2.27	2.13	3.29	4.64	8.19	3.49
Veillonella sp. - Species B - Phage	1.19	1.17	1.73	2.15	1.73	1.69	2.91	3.85	6.94	2.43
Actinomyces urogenitalis	0.45	1.25	3.15	3.44	1.93	2.77	3.29	3.59	3.09	2.28
Clostridium butyricum	0.01	0.61	1.89	1.24	12.54	3.40	2.25	0.67	0.90	2.17
Veillonella sp. - Species A - Plasmid	2.64	2.56	2.42	0.18	0.15	0.16	0.12	0.12	0.12	1.20
Veillonella sp. - Species A	1.53	1.38	2.12	0.61	0.55	0.48	0.55	0.68	0.71	1.03
Enterococcus faecalis	0.85	0.90	0.93	0.74	0.67	0.86	0.79	0.57	0.45	0.76
Staphylococcus sp. - Plasmid B	0.47	0.69	0.46	0.38	0.64	0.72	0.46	0.94	1.15	0.65
Staphylococcus sp. - Plasmid A	0.49	0.53	0.28	0.35	0.40	0.56	0.56	0.72	0.98	0.54
Staphylococcus sp. - Phage	0.34	0.30	0.37	0.65	0.92	0.34	0.27	0.32	0.42	0.42
Escherichia coli - Strain B	0.50	0.61	0.39	0.23	0.34	0.21	0.19	0.30	0.23	0.37
Varibaculum cambriense	0.02	0.39	0.14	0.87	0.65	0.26	0.40	0.48	0.33	0.37
Streptococcus parasanguinis - Plasmid	0.00	0.00	0.00	0.05	0.04	0.28	0.36	1.00	1.47	0.31
Veillonella sp. - Species B	0.09	0.15	0.27	0.38	0.22	0.32	0.35	0.48	0.39	0.27
Clostridium bartlettii	0.08	0.16	0.30	0.22	0.23	0.29	0.23	0.14	0.12	0.18
Negativicoccus succinicivorans	0.16	0.12	0.16	0.07	0.07	0.08	0.10	0.05	0.05	0.10
Staphylococcus sp.	0.04	0.06	0.06	0.11	0.13	0.13	0.07	0.14	0.14	0.09
Propionibacterium sp.	0.14	0.10	0.11	0.03	0.03	0.02	0.02	0.02	0.02	0.07
Streptococcus parasanguinis	0.00	0.00	0.00	0.02	0.01	0.05	0.06	0.16	0.29	0.06
Leuconostoc sp.	0.03	0.05	0.14	0.03	0.19	0.03	0.06	0.01	0.05	0.06
Streptococcus sp.	0.01	0.12	0.19	0.01	0.01	0.02	0.01	0.02	0.04	0.05

Supplemental Table 5 - Relative abundance of bacterial genomes.

Relative abundance of reconstructed bacterial genomes at each time point and as a total.

Genome	DOL 14	DOL 15	DOL 15	DOL 18	DOL 18	DOL 19	DOL 19	DOL 20	DOL 20	Total
Streptococcus anginosus	0.10	0.40	0.41	34.13	28.49	34.26	31.40	34.00	27.17	23.46
Escherichia coli - Strain A	52.52	33.85	27.04	18.07	11.90	13.75	16.08	20.51	24.32	22.45
Veillonella dispar	9.50	18.72	22.98	20.39	11.28	22.25	24.08	18.04	17.27	18.22
Actinomyces urogenitalis	4.43	9.94	15.85	11.79	5.31	9.23	11.15	13.46	14.17	10.41
Clostridium butyricum	0.11	4.86	9.53	4.25	34.47	11.31	7.65	2.52	4.14	9.92
Veillonella sp. - Species A	14.86	11.01	10.69	2.09	1.52	1.60	1.86	2.57	3.26	4.70
Enterococcus faecalis	8.27	7.17	4.67	2.53	1.83	2.87	2.69	2.13	2.05	3.49
Escherichia coli - Strain B	4.85	4.87	1.94	0.78	0.95	0.70	0.66	1.13	1.06	1.69
Varibaculum cambriense	0.16	3.14	0.72	2.97	1.79	0.88	1.36	1.78	1.50	1.68
Veillonella sp. - Species B	0.87	1.22	1.34	1.29	0.61	1.07	1.18	1.80	1.79	1.22
Clostridium bartlettii	0.77	1.27	1.51	0.75	0.64	0.97	0.79	0.53	0.57	0.83
Negativicoccus succinicivorans	1.53	0.92	0.80	0.25	0.19	0.26	0.33	0.20	0.23	0.46
Staphylococcus sp.	0.38	0.47	0.32	0.39	0.35	0.44	0.25	0.52	0.63	0.41
Propionibacterium sp.	1.33	0.83	0.57	0.11	0.10	0.07	0.07	0.08	0.11	0.30
Streptococcus parasanguinis	0.01	0.02	0.02	0.06	0.03	0.16	0.22	0.62	1.35	0.28
Leuconostoc sp.	0.25	0.36	0.69	0.10	0.52	0.11	0.21	0.04	0.21	0.27
Streptococcus sp.	0.07	0.94	0.93	0.04	0.02	0.07	0.03	0.08	0.18	0.22
