# Supplementary material for: Genome resolved analysis of a premature infant gut microbial community reveals a Varibaculum cambriense genome and a shift towards fermentation-based metabolism during the third week of life
Source: Microbiome. 2013 Dec 17;1:30. doi: 10.1186/2049-2618-1-30 (PMC4177395; doi:10.1186/2049-2618-1-30)
Supplement: Additional file 5 — KEGG annotations. [file 2049-2618-1-30-S5.zip › af5_V_cambriense_pathway_tools/1.0/reports/DORA_filled-holes.html]

Filled pathway holes in <i>Varibaculum cambriense</i> dora.

# Filled pathway holes in *Varibaculum cambriense* dora.

16:38:02 of Thursday, 1/17/2013 (GMT-8)

52 of 518 pathway holes were filled (10.0%).

|  |  |  |  |  |  |
| --- | --- | --- | --- | --- | --- |
| **Hole-filler** | **Hole EC#** | **P(has-function)** | **All functions of hole-filler** | **Pathway(s) requiring this reaction** | **Date filled** |
| mcu:HMPREF0573\_10379 | 2.5.1.76 | 0.975 | mcu:HMPREF0573\_10379 thrC; threonine synthase (EC:4.2.3.1); K01733 threonine synthase [EC:4.2.3.1] (db=KEGG) (RBH) B | coenzyme M biosynthesis II | 17-Jan-2013 |
| cfi:Celf\_2759 | 3.2.1.33 | 0.951 | cfi:Celf\_2759 glycogen debranching enzyme GlgX; K02438 glycogen operon protein GlgX [EC:3.2.1.-] (db=KEGG) (RBH) B | glycogen degradation I | 17-Jan-2013 |
| cfi:Celf\_2759 | 3.2.1.33 | 0.951 | cfi:Celf\_2759 glycogen debranching enzyme GlgX; K02438 glycogen operon protein GlgX [EC:3.2.1.-] (db=KEGG) (RBH) B | glycogen degradation I | 17-Jan-2013 |
| mcu:HMPREF0573\_11568 | 1.1.1.290 | 0.939 | mcu:HMPREF0573\_11568 serA2; phosphoglycerate dehydrogenase (EC:1.1.1.95); K00058 D-3-phosphoglycerate dehydrogenase [EC:1.1.1.95] (db=KEGG) (RBH) B | pyridoxal 5'-phosphate biosynthesis I | 17-Jan-2013 |
| mcu:HMPREF0573\_11568 | 1.1.1.28 | 0.982 | mcu:HMPREF0573\_11568 serA2; phosphoglycerate dehydrogenase (EC:1.1.1.95); K00058 D-3-phosphoglycerate dehydrogenase [EC:1.1.1.95] (db=KEGG) (RBH) B | mixed acid fermentation | 17-Jan-2013 |
| mcu:HMPREF0573\_11568 | 1.1.1.28 | 0.982 | mcu:HMPREF0573\_11568 serA2; phosphoglycerate dehydrogenase (EC:1.1.1.95); K00058 D-3-phosphoglycerate dehydrogenase [EC:1.1.1.95] (db=KEGG) (RBH) B | mixed acid fermentation | 17-Jan-2013 |
| mcu:HMPREF0573\_11568 | 1.1.1.290 | 0.939 | mcu:HMPREF0573\_11568 serA2; phosphoglycerate dehydrogenase (EC:1.1.1.95); K00058 D-3-phosphoglycerate dehydrogenase [EC:1.1.1.95] (db=KEGG) (RBH) B | pyridoxal 5'-phosphate biosynthesis I | 17-Jan-2013 |
| ROK | 2.7.1.60 | 0.991 | ROK family protein; K00845 glucokinase [EC:2.7.1.2] (db=KEGG evalue=0.0 bit\_score=262.0 identity=41.5 coverage=95.87301587301587) (BLAST) C | N-acetylneuraminate and N-acetylmannosamine degradation | 17-Jan-2013 |
| ROK | 2.7.1.59 | 0.978 | ROK family protein; K00845 glucokinase [EC:2.7.1.2] (db=KEGG evalue=0.0 bit\_score=262.0 identity=41.5 coverage=95.87301587301587) (BLAST) C | chitin derivatives degradation, N-acetylglucosamine degradation II | 17-Jan-2013 |
| gvh:HMPREF9231\_0059 | 4.1.3.3 | 0.982 | gvh:HMPREF9231\_0059 dihydrodipicolinate synthase (EC:4.2.1.52); K01714 dihydrodipicolinate synthase [EC:4.2.1.52] (db=KEGG) (RBH) B | N-acetylneuraminate and N-acetylmannosamine degradation | 17-Jan-2013 |
| cga:Celgi\_0340 | 2.1.1.- | 0.941 | cga:Celgi\_0340 FAD linked oxidase domain protein; K06911 (db=KEGG) (RBH) B | mycolate biosynthesis | 17-Jan-2013 |
| cga:Celgi\_0340 | 2.1.1.- | 0.941 | cga:Celgi\_0340 FAD linked oxidase domain protein; K06911 (db=KEGG) (RBH) B | mycolate biosynthesis | 17-Jan-2013 |
| cga:Celgi\_0340 | 2.1.1.- | 0.941 | cga:Celgi\_0340 FAD linked oxidase domain protein; K06911 (db=KEGG) (RBH) B | mycolate biosynthesis | 17-Jan-2013 |
| cga:Celgi\_0340 | 2.1.1.- | 0.941 | cga:Celgi\_0340 FAD linked oxidase domain protein; K06911 (db=KEGG) (RBH) B | mycolate biosynthesis | 17-Jan-2013 |
| cga:Celgi\_1503 | 3.2.1.68 | 0.998 | cga:Celgi\_1503 glycogen debranching enzyme GlgX; K02438 glycogen operon protein GlgX [EC:3.2.1.-] (db=KEGG) (RBH) B | trehalose biosynthesis V | 17-Jan-2013 |
| cga:Celgi\_1503 | 3.2.1.68 | 0.998 | cga:Celgi\_1503 glycogen debranching enzyme GlgX; K02438 glycogen operon protein GlgX [EC:3.2.1.-] (db=KEGG) (RBH) B | trehalose biosynthesis V | 17-Jan-2013 |
| mcu:HMPREF0573\_10425 | 2.7.9.2 | 0.907 | mcu:HMPREF0573\_10425 ppdK; pyruvate phosphate dikinase (EC:2.7.9.1); K01006 pyruvate,orthophosphate dikinase [EC:2.7.9.1] (db=KEGG) (RBH) B | glycolysis I, gluconeogenesis I | 17-Jan-2013 |
| iva:Isova\_2663 | 2.5.1.- | 1.000 | iva:Isova\_2663 O-acetylhomoserine/O-acetylserine sulfhydrylase (EC:2.5.1.47); K01740 O-acetylhomoserine (thiol)-lyase [EC:2.5.1.49] (db=KEGG) (RBH) B | methionine biosynthesis II | 17-Jan-2013 |
| iva:Isova\_2663 | 2.5.1.- | 1.000 | iva:Isova\_2663 O-acetylhomoserine/O-acetylserine sulfhydrylase (EC:2.5.1.47); K01740 O-acetylhomoserine (thiol)-lyase [EC:2.5.1.49] (db=KEGG) (RBH) B | seleno-amino acid biosynthesis | 17-Jan-2013 |
| iva:Isova\_2663 | 2.5.1.48 | 1.000 | iva:Isova\_2663 O-acetylhomoserine/O-acetylserine sulfhydrylase (EC:2.5.1.47); K01740 O-acetylhomoserine (thiol)-lyase [EC:2.5.1.49] (db=KEGG) (RBH) B | methionine biosynthesis I | 17-Jan-2013 |
| iva:Isova\_2663 | 4.4.1.8 | 0.999 | iva:Isova\_2663 O-acetylhomoserine/O-acetylserine sulfhydrylase (EC:2.5.1.47); K01740 O-acetylhomoserine (thiol)-lyase [EC:2.5.1.49] (db=KEGG) (RBH) B | methionine biosynthesis II, methionine biosynthesis I | 17-Jan-2013 |
| cga:Celgi\_2334 | none | 0.988 | cga:Celgi\_2334 2-oxoglutarate dehydrogenase E1; K00164 2-oxoglutarate dehydrogenase E1 component [EC:1.2.4.2] (db=KEGG) (RBH) B | TCA cycle II (eukaryotic), TCA cycle I (prokaryotic) | 17-Jan-2013 |
| bcv:Bcav\_1999 | 3.1.7.2 | 1.000 | bcv:Bcav\_1999 (p)ppGpp synthetase I SpoT/RelA (EC:2.7.6.5); K00951 GTP pyrophosphokinase [EC:2.7.6.5] (db=KEGG) (RBH) B | ppGpp biosynthesis | 17-Jan-2013 |
| bcv:Bcav\_1999 | 3.1.7.2 | 1.000 | bcv:Bcav\_1999 (p)ppGpp synthetase I SpoT/RelA (EC:2.7.6.5); K00951 GTP pyrophosphokinase [EC:2.7.6.5] (db=KEGG) (RBH) B | ppGpp biosynthesis | 17-Jan-2013 |
| ahe:Arch\_1100 | none | 0.911 | ahe:Arch\_1100 glutamyl-tRNA synthetase; K01885 glutamyl-tRNA synthetase [EC:6.1.1.17] (db=KEGG) (RBH) B | L-glutamine biosynthesis II (tRNA-dependent) | 17-Jan-2013 |
| UniRef90\_E8JFA8 | 4.1.1.48 | 0.993 | UniRef90\_E8JFA8 Indole-3-glycerol phosphate synthase n=1 Tax=Actinomyces sp. oral taxon 178 str. F0338 RepID=E8JFA8\_9ACTO (db=UNIREF) (RBH) B | tryptophan biosynthesis | 17-Jan-2013 |
| UniRef90\_E8JFA8 | 4.1.1.48 | 0.993 | UniRef90\_E8JFA8 Indole-3-glycerol phosphate synthase n=1 Tax=Actinomyces sp. oral taxon 178 str. F0338 RepID=E8JFA8\_9ACTO (db=UNIREF) (RBH) B | tryptophan biosynthesis | 17-Jan-2013 |
| bcv:Bcav\_2235 | 2.4.2.- | 0.963 | bcv:Bcav\_2235 Imidazole glycerol phosphate synthase cyclase subunit; K02500 cyclase [EC:4.1.3.-] (db=KEGG) (RBH) B | histidine biosynthesis | 17-Jan-2013 |
| bcv:Bcav\_2235 | 2.4.2.- | 0.963 | bcv:Bcav\_2235 Imidazole glycerol phosphate synthase cyclase subunit; K02500 cyclase [EC:4.1.3.-] (db=KEGG) (RBH) B | histidine biosynthesis | 17-Jan-2013 |
| ahe:Arch\_0909 | 3.6.1.- | 0.975 | ahe:Arch\_0909 undecaprenol kinase; K06153 undecaprenyl-diphosphatase [EC:3.6.1.27] (db=KEGG) (RBH) B | mono-trans, poly-cis decaprenyl phosphate biosynthesis | 17-Jan-2013 |
| ahe:Arch\_0927 | 2.4.1.21 | 0.984 | ahe:Arch\_0927 glycogen synthase (db=KEGG) (RBH) B | starch biosynthesis | 17-Jan-2013 |
| ahe:Arch\_0927 | 2.4.1.21 | 0.984 | ahe:Arch\_0927 glycogen synthase (db=KEGG) (RBH) B | starch biosynthesis | 17-Jan-2013 |
| UniRef90\_E8JEQ8 | 2.7.1.23 | 0.998 | UniRef90\_E8JEQ8 Probable inorganic polyphosphate/ATP-NAD kinase n=1 Tax=Actinomyces sp. oral taxon 178 str. F0338 RepID=E8JEQ8\_9ACTO (db=UNIREF) (RBH) B | NAD phosphorylation and dephosphorylation | 17-Jan-2013 |
| UniRef90\_E8JEQ8 | 2.7.1.23 | 0.998 | UniRef90\_E8JEQ8 Probable inorganic polyphosphate/ATP-NAD kinase n=1 Tax=Actinomyces sp. oral taxon 178 str. F0338 RepID=E8JEQ8\_9ACTO (db=UNIREF) (RBH) B | NAD phosphorylation and dephosphorylation | 17-Jan-2013 |
| mcu:HMPREF0573\_10751 | none | 0.950 | mcu:HMPREF0573\_10751 putative penicillin-binding protein (db=KEGG) (RBH) B | peptidoglycan biosynthesis III (mycobacteria) | 17-Jan-2013 |
| mcu:HMPREF0573\_10751 | none | 0.950 | mcu:HMPREF0573\_10751 putative penicillin-binding protein (db=KEGG) (RBH) B | peptidoglycan biosynthesis III (mycobacteria) | 17-Jan-2013 |
| mcu:HMPREF0573\_11145 | 4.4.1.6 | 0.928 | mcu:HMPREF0573\_11145 aspC; aspartate transaminase (EC:2.6.1.1) (db=KEGG) (RBH) B | glutathione-mediated detoxification II | 17-Jan-2013 |
| mcu:HMPREF0573\_10281 | 1.3.5.1 | 0.991 | mcu:HMPREF0573\_10281 sdhB; succinate dehydrogenase/fumarate reductase iron-sulfur subunit (EC:1.3.99.1); K00240 succinate dehydrogenase iron-sulfur subunit [EC:1.3.99.1] (db=KEGG) (RBH) B | TCA cycle II (eukaryotic), TCA cycle I (prokaryotic), aerobic respiration (cytochrome c) | 17-Jan-2013 |
| mcu:HMPREF0573\_10281 | 1.3.5.1 | 0.991 | mcu:HMPREF0573\_10281 sdhB; succinate dehydrogenase/fumarate reductase iron-sulfur subunit (EC:1.3.99.1); K00240 succinate dehydrogenase iron-sulfur subunit [EC:1.3.99.1] (db=KEGG) (RBH) B | TCA cycle II (eukaryotic), TCA cycle I (prokaryotic), aerobic respiration (cytochrome c) | 17-Jan-2013 |
| ahe:Arch\_1331 | 1.4.3.16 | 0.999 | ahe:Arch\_1331 succinate dehydrogenase or fumarate reductase, flavoprotein subunit (EC:1.3.99.1); K00239 succinate dehydrogenase flavoprotein subunit [EC:1.3.99.1] (db=KEGG) (RBH) B | NAD biosynthesis I (from aspartate) | 17-Jan-2013 |
| ahe:Arch\_1331 | 1.3.5.4 | 0.980 | ahe:Arch\_1331 succinate dehydrogenase or fumarate reductase, flavoprotein subunit (EC:1.3.99.1); K00239 succinate dehydrogenase flavoprotein subunit [EC:1.3.99.1] (db=KEGG) (RBH) B | respiration (anaerobic), mixed acid fermentation | 17-Jan-2013 |
| ahe:Arch\_1331 | 1.3.5.4 | 0.980 | ahe:Arch\_1331 succinate dehydrogenase or fumarate reductase, flavoprotein subunit (EC:1.3.99.1); K00239 succinate dehydrogenase flavoprotein subunit [EC:1.3.99.1] (db=KEGG) (RBH) B | respiration (anaerobic), mixed acid fermentation | 17-Jan-2013 |
| ahe:Arch\_1331 | 1.4.3.16 | 0.999 | ahe:Arch\_1331 succinate dehydrogenase or fumarate reductase, flavoprotein subunit (EC:1.3.99.1); K00239 succinate dehydrogenase flavoprotein subunit [EC:1.3.99.1] (db=KEGG) (RBH) B | NAD biosynthesis I (from aspartate) | 17-Jan-2013 |
| cfi:Celf\_2170 | none | 0.947 | cfi:Celf\_2170 phospho-2-dehydro-3-deoxyheptonate aldolase (EC:2.5.1.54); K01626 3-deoxy-7-phosphoheptulonate synthase [EC:2.5.1.54] (db=KEGG) (RBH) B | 3-amino-5-hydroxybenzoate biosynthesis | 17-Jan-2013 |
| cga:Celgi\_1403 | 1.3.5.2 | 0.991 | cga:Celgi\_1403 dihydroorotate dehydrogenase; K00226 dihydroorotate dehydrogenase (fumarate) [EC:1.3.98.1] (db=KEGG) (RBH) B | uridine-5'-phosphate biosynthesis | 17-Jan-2013 |
| rha:RHA1\_ro01876 | 2.3.1.16 | 0.996 | rha:RHA1\_ro01876 acetyl-CoA C-acetyltransferase (EC:2.3.1.9); K00626 acetyl-CoA C-acetyltransferase [EC:2.3.1.9] (db=KEGG) (RBH) B | fatty acid beta-oxidation I | 17-Jan-2013 |
| rha:RHA1\_ro01876 | 2.3.1.16 | 0.935 | rha:RHA1\_ro01876 acetyl-CoA C-acetyltransferase (EC:2.3.1.9); K00626 acetyl-CoA C-acetyltransferase [EC:2.3.1.9] (db=KEGG) (RBH) B | sitosterol degradation to androstenedione, cholesterol degradation to androstenedione II (cholesterol dehydrogenase), cholesterol degradation to androstenedione I (cholesterol oxidase) | 17-Jan-2013 |
| rha:RHA1\_ro01876 | 2.3.1.176 | 0.935 | rha:RHA1\_ro01876 acetyl-CoA C-acetyltransferase (EC:2.3.1.9); K00626 acetyl-CoA C-acetyltransferase [EC:2.3.1.9] (db=KEGG) (RBH) B | sitosterol degradation to androstenedione, cholesterol degradation to androstenedione II (cholesterol dehydrogenase), cholesterol degradation to androstenedione I (cholesterol oxidase) | 17-Jan-2013 |
| mta:Moth\_1259 | 2.8.3.- | 0.991 | mta:Moth\_1259 propionate CoA-transferase (EC:2.8.3.1); K01026 propionate CoA-transferase [EC:2.8.3.1] (db=KEGG) (RBH) B | acetoacetate degradation (to acetyl CoA) | 17-Jan-2013 |
| fabG; | 1.1.1.35 | 0.933 | fabG; 3-ketoacyl-ACP reductase (EC:1.1.1.100); K07535 2-hydroxycyclohexanecarboxyl-CoA dehydrogenase [EC:1.1.1.-] (db=KEGG evalue=0.0 bit\_score=249.0 identity=50.4 coverage=95.2755905511811) (BLAST) C | fatty acid beta-oxidation I | 17-Jan-2013 |
| fabG; | 1.1.1.36 | 0.950 | fabG; 3-ketoacyl-ACP reductase (EC:1.1.1.100); K07535 2-hydroxycyclohexanecarboxyl-CoA dehydrogenase [EC:1.1.1.-] (db=KEGG evalue=0.0 bit\_score=249.0 identity=50.4 coverage=95.2755905511811) (BLAST) C | acetyl-CoA fermentation to butyrate II | 17-Jan-2013 |
| ske:Sked\_09950 | 2.7.8.33 | 0.984 | ske:Sked\_09950 UDP-N-acetylmuramyl pentapeptide phosphotransferase/UDP-N-acetylglucosamine-1-phosphate transferase (db=KEGG) (RBH) B | teichoic acid (poly-glycerol) biosynthesis | 17-Jan-2013 |
| msm:MSMEG\_3094 | 1.1.1.- | 0.958 | msm:MSMEG\_3094 oxidoreductase, zinc-binding dehydrogenase (db=KEGG) (RBH) B | galactose degradation IV | 17-Jan-2013 |
| msm:MSMEG\_3094 | 1.1.1.- | 0.958 | msm:MSMEG\_3094 oxidoreductase, zinc-binding dehydrogenase (db=KEGG) (RBH) B | galactose degradation IV | 17-Jan-2013 |
| pfr:PFREUD\_02440 | 6.2.1.1 | 0.993 | pfr:PFREUD\_02440 caiC; crotonobetaine/carnitine-CoA ligase (EC:6.2.1.-); K02182 crotonobetaine/carnitine-CoA ligase [EC:6.2.1.-] (db=KEGG) (RBH) B | acetate conversion to acetyl-CoA, ethanol degradation II | 17-Jan-2013 |
| pfr:PFREUD\_02440 | 6.2.1.1 | 0.993 | pfr:PFREUD\_02440 caiC; crotonobetaine/carnitine-CoA ligase (EC:6.2.1.-); K02182 crotonobetaine/carnitine-CoA ligase [EC:6.2.1.-] (db=KEGG) (RBH) B | acetate conversion to acetyl-CoA, ethanol degradation II | 17-Jan-2013 |
| pfr:PFREUD\_02420 | 1.3.8.- | 0.933 | pfr:PFREUD\_02420 caiA; crotonobetainyl-CoA dehydrogenase (EC:1.3.99.-); K08297 crotonobetainyl-CoA dehydrogenase [EC:1.3.99.-] (db=KEGG) (RBH) B | 2-methylbutyrate biosynthesis, isoleucine degradation I | 17-Jan-2013 |
| pfr:PFREUD\_02420 | 1.3.8.1 | 0.935 | pfr:PFREUD\_02420 caiA; crotonobetainyl-CoA dehydrogenase (EC:1.3.99.-); K08297 crotonobetainyl-CoA dehydrogenase [EC:1.3.99.-] (db=KEGG) (RBH) B | pyruvate fermentation to butanol I, acetyl-CoA fermentation to butyrate II | 17-Jan-2013 |
| pfr:PFREUD\_02420 | 1.3.8.1 | 0.935 | pfr:PFREUD\_02420 caiA; crotonobetainyl-CoA dehydrogenase (EC:1.3.99.-); K08297 crotonobetainyl-CoA dehydrogenase [EC:1.3.99.-] (db=KEGG) (RBH) B | pyruvate fermentation to butanol I, acetyl-CoA fermentation to butyrate II | 17-Jan-2013 |
| pfr:PFREUD\_02420 | 1.3.8.- | 0.933 | pfr:PFREUD\_02420 caiA; crotonobetainyl-CoA dehydrogenase (EC:1.3.99.-); K08297 crotonobetainyl-CoA dehydrogenase [EC:1.3.99.-] (db=KEGG) (RBH) B | 2-methylbutyrate biosynthesis, isoleucine degradation I | 17-Jan-2013 |
| mcu:HMPREF0573\_10607 | 1.17.4.- | 0.988 | mcu:HMPREF0573\_10607 nrdA; ribonucleotide-diphosphate reductase subunit alpha (EC:1.17.4.1); K00525 ribonucleoside-diphosphate reductase alpha chain [EC:1.17.4.1] (db=KEGG) (RBH) B | adenosine nucleotides de novo biosynthesis | 17-Jan-2013 |
| mcu:HMPREF0573\_10607 | 1.17.4.- | 0.988 | mcu:HMPREF0573\_10607 nrdA; ribonucleotide-diphosphate reductase subunit alpha (EC:1.17.4.1); K00525 ribonucleoside-diphosphate reductase alpha chain [EC:1.17.4.1] (db=KEGG) (RBH) B | guanosine nucleotides de novo biosynthesis | 17-Jan-2013 |
| mcu:HMPREF0573\_10607 | 1.17.4.- | 0.999 | mcu:HMPREF0573\_10607 nrdA; ribonucleotide-diphosphate reductase subunit alpha (EC:1.17.4.1); K00525 ribonucleoside-diphosphate reductase alpha chain [EC:1.17.4.1] (db=KEGG) (RBH) B | pyrimidine deoxyribonucleotides de novo biosynthesis I | 17-Jan-2013 |
| mcu:HMPREF0573\_10607 | 1.17.4.- | 0.999 | mcu:HMPREF0573\_10607 nrdA; ribonucleotide-diphosphate reductase subunit alpha (EC:1.17.4.1); K00525 ribonucleoside-diphosphate reductase alpha chain [EC:1.17.4.1] (db=KEGG) (RBH) B | pyrimidine deoxyribonucleotides de novo biosynthesis I | 17-Jan-2013 |
| ahe:Arch\_0038 | 1.1.1.22 | 0.958 | ahe:Arch\_0038 alcohol dehydrogenase zinc-binding domain protein; K00121 S-(hydroxymethyl)glutathione dehydrogenase / alcohol dehydrogenase [EC:1.1.1.284 1.1.1.1] (db=KEGG) (RBH) B | galactose degradation III | 17-Jan-2013 |
| iva:Isova\_0655 | 2.7.1.25 | 0.990 | iva:Isova\_0655 translation elongation factor Tu (EC:2.7.7.4); K02358 elongation factor Tu (db=KEGG) (RBH) B | sulfate activation for sulfonation | 17-Jan-2013 |
| mcu:HMPREF0573\_10969 | 3.5.1.16 | 0.925 | mcu:HMPREF0573\_10969 rpoC; DNA-directed RNA polymerase (EC:2.7.7.6); K03046 DNA-directed RNA polymerase subunit beta' [EC:2.7.7.6] (db=KEGG) (RBH) B | ornithine biosynthesis | 17-Jan-2013 |
| paz:TIA2EST2\_02200 | 1.2.1.8 | 0.997 | paz:TIA2EST2\_02200 methylmalonate-semialdehyde dehydrogenase; K00140 malonate-semialdehyde dehydrogenase (acetylating) / methylmalonate-semialdehyde dehydrogenase [EC:1.2.1.18 1.2.1.27] (db=KEGG) (RBH) B | glycine betaine biosynthesis II (Gram-positive bacteria) | 17-Jan-2013 |
