# Supplementary material for: Genome resolved analysis of a premature infant gut microbial community reveals a Varibaculum cambriense genome and a shift towards fermentation-based metabolism during the third week of life
Source: Microbiome. 2013 Dec 17;1:30. doi: 10.1186/2049-2618-1-30 (PMC4177395; doi:10.1186/2049-2618-1-30)
Supplement: Additional file 5 — KEGG annotations. [file 2049-2618-1-30-S5.zip › af5_V_cambriense_pathway_tools/1.0/reports/DORAholes0-119.html]

 Identification of enzymes to fill pathway holes in  Varibaculum cambriense  dora 
 Identification of enzymes to fill pathway holes in  Varibaculum cambriense  dora 
Nodes used in Bayesian network:
 SAME-GENE DIRECTON ADJACENT SSCORE EVALS ALN RANK 
   
  

 

 
 Summary Statistics:    Number of pathways analyzed: 117   Number of pathway holes: 480   One or more candidates have been identified for 241 holes.
 

 

 
     List of Pathways 
  

 
 chorismate biosynthesis from 3-dehydroquinate 
 
 alanine biosynthesis I 
 
 asparagine biosynthesis III (tRNA-dependent) 
 
 &beta;-alanine biosynthesis I 
 
 glutamine biosynthesis III 
 
 lysine biosynthesis I 
 
 methionine biosynthesis II 
 
 methionine biosynthesis I 
 
  S -adenosyl-L-methionine cycle I 
 
 ornithine biosynthesis 
 
 proline biosynthesis II (from arginine) 
 
 serine biosynthesis 
 
 tryptophan biosynthesis 
 
 tyrosine biosynthesis I 
 
 glycogen degradation I 
 
 starch degradation II 
 
 starch degradation I 
 
 sucrose biosynthesis 
 
 gluconeogenesis I 
 
 galactose degradation III 
 
 starch biosynthesis 
 
 seleno-amino acid biosynthesis 
 
 2-methylbutyrate biosynthesis 
 
 autoinducer AI-2 biosynthesis II ( Vibrio ) 
 
 autoinducer AI-2 biosynthesis I 
 
 teichoic acid (poly-glycerol) biosynthesis 
 
 UDP- N -acetylmuramoyl-pentapeptide biosynthesis II (lysine-containing) 
 
 peptidoglycan biosynthesis III (mycobacteria) 
 
 peptidoglycan biosynthesis V (&beta;-lactam resistance) 
 
 coenzyme M biosynthesis II 
 
 molybdenum cofactor biosynthesis 
 
 NAD phosphorylation and dephosphorylation 
 
 NAD biosynthesis I (from aspartate) 
 
 NAD salvage pathway I 
 
 mono- trans , poly- cis  decaprenyl phosphate biosynthesis 
 
 methylerythritol phosphate pathway 
 
  trans, trans -farnesyl diphosphate biosynthesis 
 
 superpathway of heme biosynthesis from uroporphyrinogen-III 
 
 1,4-dihydroxy-2-naphthoate biosynthesis I 
 
 ubiquinol-6 biosynthesis (eukaryotic) 
 
 ubiquinol-8 biosynthesis (prokaryotic) 
 
 tetrapyrrole biosynthesis I 
 
 flavin biosynthesis I (bacteria and plants) 
 
 formylTHF biosynthesis I 
 
 folate transformations I 
 
 6-hydroxymethyl-dihydropterin diphosphate biosynthesis I 
 
 phosphopantothenate biosynthesis I 
 
 thiamin diphosphate biosynthesis I (E. coli) 
 
 thiamin diphosphate biosynthesis II (Bacillus) 
 
 thiamin salvage I 
 
 pyridoxal 5'-phosphate biosynthesis I 
 
 juvenile hormone III biosynthesis I 
 
 juvenile hormone III biosynthesis II 
 
 salidroside biosynthesis 
 
 phenylethanol biosynthesis 
 
 3-amino-5-hydroxybenzoate biosynthesis 
 
 streptomycin biosynthesis 
 
 D- myo -inositol (1,4,5)-trisphosphate degradation 
 
 mycolate biosynthesis 
 
 CDP-diacylglycerol biosynthesis II 
 
 CDP-diacylglycerol biosynthesis I 
 
 phosphatidylglycerol biosynthesis II (non-plastidic) 
 
 tRNA processing pathway I 
 
 queuosine biosynthesis 
 
 guanosine nucleotides  de novo  biosynthesis 
 
 inosine-5'-phosphate biosynthesis I 
 
 pyrimidine ribonucleotides interconversion 
 
 pyrimidine deoxyribonucleotides  de novo  biosynthesis I 
 
 glycine betaine biosynthesis II (Gram-positive bacteria) 
 
  myo -inositol degradation 
 
 glycerol degradation I 
 
 arginine degradation X (arginine monooxygenase pathway) 
 
 isoleucine degradation I 
 
 citrulline degradation 
 
 proline degradation 
 
 tyrosine degradation III 
 
 valine degradation II 
 
 CO 2  fixation into oxaloacetate (anapleurotic) 
 
 reductive monocarboxylic acid cycle 
 
 formaldehyde oxidation II (glutathione-dependent) 
 
 acrylate degradation 
 
 stachyose degradation 
 
 chitin degradation II 
 
 chitin derivatives degradation 
 
 glycogen degradation II 
 
 chondroitin sulfate degradation (metazoa) 
 
 dermatan sulfate degradation (metazoa) 
 
 starch degradation V 
 
 xyloglucan degradation II (exoglucanase) 
 
 xyloglucan degradation I (endoglucanase) 
 
 galactose degradation IV 
 
 galactose degradation I (Leloir pathway) 
 
 L-arabinose degradation I 
 
 sucrose degradation V (mammalian) 
 
 fatty acid &beta;-oxidation I 
 
 purine deoxyribonucleosides degradation 
 
 adenosine nucleotides degradation I 
 
 pyrimidine deoxyribonucleosides degradation 
 
 nitrate reduction VI (assimilatory) 
 
 ( R )-cysteate degradation 
 
 sulfolactate degradation III 
 
 androstenedione degradation 
 
 sitosterol degradation to androstenedione 
 
 cholesterol degradation to androstenedione I (cholesterol oxidase) 
 
 cholesterol degradation to androstenedione II (cholesterol dehydrogenase) 
 
 glutathione-mediated detoxification II 
 
 superoxide radicals degradation 
 
 pyruvate fermentation to butanol I 
 
 mixed acid fermentation 
 
 acetyl-CoA fermentation to butyrate II 
 
 pyruvate fermentation to acetate IV 
 
 glycolysis I 
 
 glycerol-3-phosphate shuttle 
 
 photosynthesis light reactions 
 
 respiration (anaerobic) 
 
 TCA cycle V (2-oxoglutarate:ferredoxin oxidoreductase) 
 
 TCA cycle I (prokaryotic) 
 
     

  chorismate biosynthesis from 3-dehydroquinate     Total # of reactions in pathway = 6   Present reactions: 5
  

 

 
 Reaction 
 

 
 Protein(s) 
 

 

 

 
CHORISMATE-SYNTHASE-RXN
 

 
(# )
 

 

 

 
2.5.1.19-RXN
 

 
(# )
 

 

 

 
SHIKIMATE-KINASE-RXN
 

 
(# 
 # )
 

 

 

 
SHIKIMATE-5-DEHYDROGENASE-RXN
 

 
(# )
 

 

 

 
3-DEHYDROQUINATE-DEHYDRATASE-RXN
 

 
(# )
 

 

 
   Missing reactions: 1
  

 

 
 RXN-7968 
 

 

 
        Evidence for quinate/shikimate dehydrogenase, EC# 1.1.1.282   RXN-7968 
  
no BLAST hits found
 
     

  alanine biosynthesis I     Total # of reactions in pathway = 3   Present reactions: 2
  

 

 
 Reaction 
 

 
 Protein(s) 
 

 

 

 
BRANCHED-CHAINAMINOTRANSFERVAL-RXN
 

 
(# )
 

 

 

 
ALARACECAT-RXN
 

 
(# )
 

 

 
   Missing reactions: 1
  

 

 
 VALINE-PYRUVATE-AMINOTRANSFER-RXN 
 

 

 
        Evidence for valine&mdash;pyruvate transaminase, EC# 2.6.1.66   VALINE-PYRUVATE-AMINOTRANSFER-RXN 
  

 

 
 Hit 
 

 
 Common 
 

 
 P 
 

 
 #Q 
 

 
 Best Qry 
 

 
 Best Eval 
 

 
 Avg Rank 
 

 
 Aln Len 
 

 
 Qry Len 
 

 
 IDOP? 
 

 
 adj? 
 

 

 

 
GKFG-364-MONOMER
 

 
mcu:HMPREF0573_11145 aspC
 

 
0.039642163
 

 
1.0
 

 
P09053
 

 
5.38966e-7
 

 
1.0
 

 
0.98081535
 

 
417
 

 
T
 

 
NIL
 

 

 

 
GKFG-510-MONOMER
 

 
bcv:Bcav_3969 histidinol-
 

 
6.873975e-5
 

 
1.0
 

 
P09053
 

 
0.0400613
 

 
2.0
 

 
0.2470024
 

 
417
 

 
NIL
 

 
NIL
 

 

 

 
GKFG-288-MONOMER
 

 
metallophosphoesterase (d
 

 
1.0926308e-6
 

 
1.0
 

 
P09053
 

 
0.182987
 

 
3.0
 

 
0.086330935
 

 
417
 

 
T
 

 
NIL
 

 

 
     

  asparagine biosynthesis III (tRNA-dependent)     Total # of reactions in pathway = 3   Present reactions: 2
  

 

 
 Reaction 
 

 
 Protein(s) 
 

 

 

 
6.3.5.6-RXN
 

 
(# 
 # 
 # 
 # )
 

 

 

 
RXN-12460
 

 
(# )
 

 

 
   Missing reactions: 1
  

 

 
 RXN490-3616 
 

 

 
        Evidence for Aspartate--tRNA ligase, EC# 6.1.1.-   RXN490-3616 
  

 

 
 Hit 
 

 
 Common 
 

 
 P 
 

 
 #Q 
 

 
 Best Qry 
 

 
 Best Eval 
 

 
 Avg Rank 
 

 
 Aln Len 
 

 
 Qry Len 
 

 
 IDOP? 
 

 
 adj? 
 

 

 

 
GKFG-1794-MONOMER
 

 
iva:Isova_1635 aspartyl-t
 

 
0.42053136
 

 
1.0
 

 
Q9RVH4
 

 
9.27824e-25
 

 
1.0
 

 
0.67356324
 

 
435
 

 
T
 

 
NIL
 

 

 

 
GKFG-1029-MONOMER
 

 
mcu:HMPREF0573_10505 lysS
 

 
0.15667598
 

 
1.0
 

 
Q9RVH4
 

 
5.97769e-14
 

 
2.0
 

 
0.78390807
 

 
435
 

 
T
 

 
NIL
 

 

 

 
GKFG-720-MONOMER
 

 
fabG; 3-ketoacyl-ACP redu
 

 
1.2082764e-6
 

 
1.0
 

 
Q9RVH4
 

 
0.442382
 

 
3.0
 

 
0.17471264
 

 
435
 

 
T
 

 
NIL
 

 

 
     

  &beta;-alanine biosynthesis I     Total # of reactions in pathway = 2   Present reactions: 1
  

 

 
 Reaction 
 

 
 Protein(s) 
 

 

 

 
RXN-6382
 

 
(# )
 

 

 
   Missing reactions: 1
  

 

 
 RXN-6381 
 

 

 
        Evidence for , EC# 1.4.3.22   RXN-6381      

  glutamine biosynthesis III     Total # of reactions in pathway = 9   Present reactions: 7
  

 

 
 Reaction 
 

 
 Protein(s) 
 

 

 

 
PYRUVATEORTHOPHOSPHATE-DIKINASE-RXN
 

 
(# )
 

 

 

 
CITSYN-RXN
 

 
(# )
 

 

 

 
ISOCITRATE-DEHYDROGENASE-NAD+-RXN
 

 
(# )
 

 

 

 
GLUTAMINESYN-RXN
 

 
(# 
 # )
 

 

 

 
ACONITATEDEHYDR-RXN
 

 
(# )
 

 

 

 
ACONITATEHYDR-RXN
 

 
(# )
 

 

 

 
ISOCITDEH-RXN
 

 
(# )
 

 

 
   Missing reactions: 2
  

 

 
 PEPCARBOX-RXN 
 

 

 

 
 GLUTAMATE-SYNTHASE-FERREDOXIN-RXN 
 

 

 
        Evidence for phospho enol pyruvate carboxylase, EC# 4.1.1.31   PEPCARBOX-RXN 
  

 

 
 Hit 
 

 
 Common 
 

 
 P 
 

 
 #Q 
 

 
 Best Qry 
 

 
 Best Eval 
 

 
 Avg Rank 
 

 
 Aln Len 
 

 
 Qry Len 
 

 
 IDOP? 
 

 
 adj? 
 

 

 

 
GKFG-289-MONOMER
 

 
mcu:HMPREF0573_10751 puta
 

 
1.5421621e-5
 

 
1.0
 

 
A8AW99
 

 
0.703679
 

 
1.0
 

 
0.21518987
 

 
948
 

 
T
 

 
NIL
 

 

 

 
GKFG-1465-MONOMER
 

 
iva:Isova_1268 UvrD/REP h
 

 
1.5421621e-5
 

 
1.0
 

 
A4VN44
 

 
0.215022
 

 
1.0
 

 
0.1592719
 

 
879
 

 
T
 

 
NIL
 

 

 

 
GKFG-1767-MONOMER
 

 
hypothetical protein (db=
 

 
1.5421621e-5
 

 
1.0
 

 
Q0STS8
 

 
0.046729
 

 
1.0
 

 
0.1359404
 

 
537
 

 
T
 

 
NIL
 

 

 

 
GKFG-594-MONOMER
 

 
hypothetical protein (db=
 

 
1.5421621e-5
 

 
1.0
 

 
B0R7F9
 

 
0.884405
 

 
1.0
 

 
0.14634146
 

 
492
 

 
T
 

 
NIL
 

 

 

 
GKFG-765-MONOMER
 

 
cfl:Cfla_2325 Preprotein 
 

 
1.5421621e-5
 

 
1.0
 

 
Q8TZL5
 

 
0.14614
 

 
1.0
 

 
0.17372881
 

 
472
 

 
T
 

 
NIL
 

 

 

 
GKFG-142-MONOMER
 

 
dsy:DSY3812 hypothetical 
 

 
1.3945618e-5
 

 
1.0
 

 
Q8PS70
 

 
0.667179
 

 
1.0
 

 
0.10076046
 

 
526
 

 
T
 

 
NIL
 

 

 

 
GKFG-1046-MONOMER
 

 
xce:Xcel_0723 ATP-depende
 

 
1.3945618e-5
 

 
1.0
 

 
O32483
 

 
0.139123
 

 
1.0
 

 
0.053418804
 

 
936
 

 
T
 

 
NIL
 

 

 

 
GKFG-1621-MONOMER
 

 
tcu:Tcur_3714 3-phosphosh
 

 
1.3945618e-5
 

 
1.0
 

 
O28786
 

 
0.102436
 

 
1.0
 

 
0.10403397
 

 
471
 

 
T
 

 
NIL
 

 

 

 
GKFG-739-MONOMER
 

 
bcv:Bcav_1600 DEAD/DEAH b
 

 
1.3945618e-5
 

 
1.0
 

 
A1R2V3
 

 
0.792382
 

 
1.0
 

 
0.04828326
 

 
932
 

 
T
 

 
NIL
 

 

 

 
GKFG-1304-MONOMER
 

 
UniRef90_UPI000051013A hy
 

 
1.3945618e-5
 

 
1.0
 

 
Q5SKL7
 

 
0.671567
 

 
1.0
 

 
0.09440559
 

 
858
 

 
T
 

 
NIL
 

 

 

 
GKFG-732-MONOMER
 

 
ahe:Arch_1078 LuxR family
 

 
1.3945618e-5
 

 
1.0
 

 
Q2SL23
 

 
0.350988
 

 
1.0
 

 
0.08399546
 

 
881
 

 
T
 

 
NIL
 

 

 

 
GKFG-1347-MONOMER
 

 
aur:HMPREF9243_0883 hypot
 

 
1.3945618e-5
 

 
1.0
 

 
A4JGV5
 

 
0.0294267
 

 
1.0
 

 
0.055165496
 

 
997
 

 
T
 

 
NIL
 

 

 

 
GKFG-1068-MONOMER
 

 
ckp:ckrop_1874 hypothetic
 

 
1.3945618e-5
 

 
1.0
 

 
Q93MH3
 

 
0.252621
 

 
1.0
 

 
0.054406963
 

 
919
 

 
T
 

 
NIL
 

 

 

 
GKFG-86-MONOMER
 

 
Putative uncharacterized 
 

 
2.5282066e-6
 

 
2.0
 

 
O32483
 

 
0.238824
 

 
1.5
 

 
0.09476648
 

 
936
 

 
T
 

 
NIL
 

 

 

 
GKFG-1383-MONOMER
 

 
jde:Jden_1695 DEAD/DEAH b
 

 
2.5282066e-6
 

 
2.0
 

 
A4JGV5
 

 
0.0496707
 

 
1.5
 

 
0.070196435
 

 
997
 

 
T
 

 
NIL
 

 

 

 
GKFG-844-MONOMER
 

 
ahe:Arch_0366 ATP synthas
 

 
2.5282066e-6
 

 
1.0
 

 
A8AW99
 

 
0.828791
 

 
2.0
 

 
0.07489452
 

 
948
 

 
T
 

 
NIL
 

 

 

 
GKFG-177-MONOMER
 

 
ubiE; methylase; K03183 u
 

 
1.0926308e-6
 

 
1.0
 

 
O32483
 

 
0.310147
 

 
3.0
 

 
0.086538464
 

 
936
 

 
T
 

 
NIL
 

 

 
     Evidence for glutamate synthase (ferredoxin), EC# 1.4.7.1   GLUTAMATE-SYNTHASE-FERREDOXIN-RXN 
  

 

 
 Hit 
 

 
 Common 
 

 
 P 
 

 
 #Q 
 

 
 Best Qry 
 

 
 Best Eval 
 

 
 Avg Rank 
 

 
 Aln Len 
 

 
 Qry Len 
 

 
 IDOP? 
 

 
 adj? 
 

 

 

 
GKFG-151-MONOMER
 

 
cga:Celgi_0340 FAD linked
 

 
1.3945618e-5
 

 
1.0
 

 
Q9ZNZ7
 

 
0.119068
 

 
1.0
 

 
0.06067961
 

 
1648
 

 
T
 

 
NIL
 

 

 
     

  lysine biosynthesis I     Total # of reactions in pathway = 9   Present reactions: 6
  

 

 
 Reaction 
 

 
 Protein(s) 
 

 

 

 
DIAMINOPIMDECARB-RXN
 

 
(# )
 

 

 

 
TETHYDPICSUCC-RXN
 

 
(# )
 

 

 

 
DIHYDROPICRED-RXN
 

 
(# )
 

 

 

 
DIHYDRODIPICSYN-RXN
 

 
(# )
 

 

 

 
ASPARTATE-SEMIALDEHYDE-DEHYDROGENASE-RXN
 

 
(# )
 

 

 

 
ASPARTATEKIN-RXN
 

 
(# )
 

 

 
   Missing reactions: 3
  

 

 
 DIAMINOPIMEPIM-RXN 
 

 

 

 
 SUCCDIAMINOPIMDESUCC-RXN 
 

 

 

 
 SUCCINYLDIAMINOPIMTRANS-RXN 
 

 

 
        Evidence for diaminopimelate epimerase, EC# 5.1.1.7   DIAMINOPIMEPIM-RXN 
  

 

 
 Hit 
 

 
 Common 
 

 
 P 
 

 
 #Q 
 

 
 Best Qry 
 

 
 Best Eval 
 

 
 Avg Rank 
 

 
 Aln Len 
 

 
 Qry Len 
 

 
 IDOP? 
 

 
 adj? 
 

 

 

 
GKFG-74-MONOMER
 

 
transmembrane_regions (db
 

 
0.004913946
 

 
1.0
 

 
O29511
 

 
0.144216
 

 
1.0
 

 
0.575
 

 
280
 

 
NIL
 

 
NIL
 

 

 

 
GKFG-153-MONOMER
 

 
mcu:HMPREF0573_10425 ppdK
 

 
3.7905585e-4
 

 
2.0
 

 
A3M804
 

 
0.0222559
 

 
1.0
 

 
0.26690394
 

 
281
 

 
NIL
 

 
NIL
 

 

 

 
GKFG-342-MONOMER
 

 
transmembrane_regions (db
 

 
3.7905585e-4
 

 
1.0
 

 
P54897
 

 
0.123683
 

 
1.0
 

 
0.24912281
 

 
285
 

 
NIL
 

 
NIL
 

 

 

 
GKFG-63-MONOMER
 

 
154918..155439 - ( gc_con
 

 
3.7905585e-4
 

 
1.0
 

 
Q8FPE1
 

 
0.574695
 

 
1.0
 

 
0.24734983
 

 
283
 

 
NIL
 

 
NIL
 

 

 

 
GKFG-2024-MONOMER
 

 
pad:TIIST44_04395 glycero
 

 
3.182795e-4
 

 
1.0
 

 
Q2NBR6
 

 
0.91278
 

 
1.0
 

 
0.37174723
 

 
269
 

 
T
 

 
NIL
 

 

 

 
GKFG-708-MONOMER
 

 
120023..121090 - ( gc_con
 

 
3.182795e-4
 

 
1.0
 

 
Q2NYV5
 

 
0.415132
 

 
1.0
 

 
0.3943662
 

 
284
 

 
T
 

 
NIL
 

 

 

 
GKFG-1239-MONOMER
 

 
(db=HMMPfam db_id=PF05738
 

 
1.3958463e-4
 

 
1.0
 

 
B8CX90
 

 
0.821484
 

 
1.0
 

 
0.25
 

 
284
 

 
T
 

 
NIL
 

 

 

 
GKFG-1223-MONOMER
 

 
hypothetical protein (db=
 

 
1.3958463e-4
 

 
1.0
 

 
Q6G1L7
 

 
0.100518
 

 
1.0
 

 
0.24381626
 

 
283
 

 
T
 

 
NIL
 

 

 

 
GKFG-29-MONOMER
 

 
std:SPPN_11055 Zinc metal
 

 
4.188785e-5
 

 
1.0
 

 
Q9Z833
 

 
0.0897387
 

 
1.0
 

 
0.18677042
 

 
257
 

 
NIL
 

 
NIL
 

 

 

 
GKFG-316-MONOMER
 

 
putative secreted protein
 

 
4.188785e-5
 

 
1.0
 

 
Q2J390
 

 
0.185695
 

 
1.0
 

 
0.22525597
 

 
293
 

 
NIL
 

 
NIL
 

 

 

 
GKFG-726-MONOMER
 

 
mcu:HMPREF0573_11030 two-
 

 
2.5307965e-5
 

 
1.0
 

 
Q71Y02
 

 
0.170707
 

 
2.0
 

 
0.331307
 

 
329
 

 
T
 

 
NIL
 

 

 

 
GKFG-863-MONOMER
 

 
putative phage prohead pr
 

 
1.5421621e-5
 

 
2.0
 

 
C0ZYN8
 

 
0.00928361
 

 
1.0
 

 
0.18958806
 

 
292
 

 
T
 

 
NIL
 

 

 

 
GKFG-923-MONOMER
 

 
mcu:HMPREF0573_10607 nrdA
 

 
1.5421621e-5
 

 
1.0
 

 
Q71Y02
 

 
0.0594794
 

 
1.0
 

 
0.12158055
 

 
329
 

 
T
 

 
NIL
 

 

 

 
GKFG-1591-MONOMER
 

 
bcv:Bcav_0691 homoserine 
 

 
1.5421621e-5
 

 
1.0
 

 
B6IP82
 

 
0.419524
 

 
1.0
 

 
0.14089347
 

 
291
 

 
T
 

 
NIL
 

 

 

 
GKFG-1841-MONOMER
 

 
mml:MLC_0350 hypothetical
 

 
1.5421621e-5
 

 
1.0
 

 
B2UTR2
 

 
0.900521
 

 
1.0
 

 
0.16117217
 

 
273
 

 
T
 

 
NIL
 

 

 

 
GKFG-1207-MONOMER
 

 
no description (db=HMMSma
 

 
1.5421621e-5
 

 
1.0
 

 
B0RHB0
 

 
0.00777816
 

 
1.0
 

 
0.1780822
 

 
292
 

 
T
 

 
NIL
 

 

 

 
GKFG-1632-MONOMER
 

 
ahe:Arch_1316 acetate kin
 

 
1.5421621e-5
 

 
1.0
 

 
Q8DGM2
 

 
0.0816645
 

 
1.0
 

 
0.23426573
 

 
286
 

 
T
 

 
NIL
 

 

 

 
GKFG-2010-MONOMER
 

 
Rieske-type ferredoxin; K
 

 
1.5421621e-5
 

 
1.0
 

 
A0B6C1
 

 
0.214527
 

 
1.0
 

 
0.19354838
 

 
279
 

 
T
 

 
NIL
 

 

 

 
GKFG-1736-MONOMER
 

 
ribose 5-phosphate isomer
 

 
1.5421621e-5
 

 
1.0
 

 
B8FSD4
 

 
0.444248
 

 
1.0
 

 
0.1590106
 

 
283
 

 
T
 

 
NIL
 

 

 

 
GKFG-659-MONOMER
 

 
ribosome recycling factor
 

 
1.09376415e-5
 

 
1.0
 

 
Q71Y02
 

 
0.46336
 

 
3.0
 

 
0.30091184
 

 
329
 

 
T
 

 
NIL
 

 

 

 
GKFG-257-MONOMER
 

 
bcv:Bcav_0730 xylose isom
 

 
7.594039e-6
 

 
1.0
 

 
A3M804
 

 
0.644509
 

 
2.0
 

 
0.1886121
 

 
281
 

 
NIL
 

 
NIL
 

 

 

 
GKFG-1842-MONOMER
 

 
XRE family transcriptiona
 

 
2.795795e-6
 

 
1.0
 

 
C0ZYN8
 

 
0.889472
 

 
2.0
 

 
0.18150684
 

 
292
 

 
T
 

 
NIL
 

 

 

 
GKFG-1661-MONOMER
 

 
aau:AAur_3325 gdhA; gluta
 

 
2.5282066e-6
 

 
2.0
 

 
A6L7E5
 

 
0.209358
 

 
1.5
 

 
0.1140207
 

 
280
 

 
T
 

 
NIL
 

 

 

 
GKFG-844-MONOMER
 

 
ahe:Arch_0366 ATP synthas
 

 
1.6156636e-6
 

 
1.0
 

 
Q71Y02
 

 
0.582485
 

 
4.0
 

 
0.17021276
 

 
329
 

 
T
 

 
NIL
 

 

 
     Evidence for succinyl-diaminopimelate desuccinylase, EC# 3.5.1.18   SUCCDIAMINOPIMDESUCC-RXN 
  

 

 
 Hit 
 

 
 Common 
 

 
 P 
 

 
 #Q 
 

 
 Best Qry 
 

 
 Best Eval 
 

 
 Avg Rank 
 

 
 Aln Len 
 

 
 Qry Len 
 

 
 IDOP? 
 

 
 adj? 
 

 

 

 
GKFG-615-MONOMER
 

 
ahe:Arch_0744 peptidase M
 

 
0.27293468
 

 
36.0
 

 
Q1GMM6
 

 
1.27479e-11
 

 
1.0
 

 
0.2448985
 

 
395
 

 
T
 

 
NIL
 

 

 

 
GKFG-425-MONOMER
 

 
pfr:PFREUD_19070 hypothet
 

 
3.8675137e-4
 

 
1.0
 

 
A0L3N2
 

 
0.82683
 

 
3.0
 

 
0.49473685
 

 
380
 

 
NIL
 

 
NIL
 

 

 

 
GKFG-1800-MONOMER
 

 
bcv:Bcav_1999 (p)ppGpp sy
 

 
2.5307965e-5
 

 
2.0
 

 
Q2N7X8
 

 
0.12158
 

 
1.5
 

 
0.26400182
 

 
378
 

 
T
 

 
NIL
 

 

 

 
GKFG-1882-MONOMER
 

 
pcn:TIB1ST10_03215 oxidor
 

 
2.5307965e-5
 

 
1.0
 

 
A0L3N2
 

 
0.471576
 

 
2.0
 

 
0.24473684
 

 
380
 

 
T
 

 
NIL
 

 

 

 
GKFG-475-MONOMER
 

 
bfa:Bfae_25620 phosphate-
 

 
7.594039e-6
 

 
1.0
 

 
Q2RNM1
 

 
0.544254
 

 
2.0
 

 
0.16836734
 

 
392
 

 
NIL
 

 
NIL
 

 

 

 
GKFG-479-MONOMER
 

 
DNA replication and repai
 

 
7.594039e-6
 

 
1.0
 

 
Q28VJ1
 

 
0.171721
 

 
2.0
 

 
0.22938144
 

 
388
 

 
NIL
 

 
NIL
 

 

 

 
GKFG-1341-MONOMER
 

 
hypothetical protein (db=
 

 
2.795795e-6
 

 
1.0
 

 
Q99SN6
 

 
0.473594
 

 
2.0
 

 
0.15724815
 

 
407
 

 
T
 

 
NIL
 

 

 

 
GKFG-1567-MONOMER
 

 
hypothetical protein (db=
 

 
2.795795e-6
 

 
1.0
 

 
Q7VF72
 

 
0.196508
 

 
2.0
 

 
0.20408164
 

 
392
 

 
T
 

 
NIL
 

 

 

 
GKFG-888-MONOMER
 

 
UniRef90_C2KTG5 LacI fami
 

 
2.795795e-6
 

 
1.0
 

 
Q1QDC1
 

 
0.0263897
 

 
2.0
 

 
0.2063882
 

 
407
 

 
T
 

 
NIL
 

 

 

 
GKFG-1270-MONOMER
 

 
jde:Jden_1096 bifunctiona
 

 
2.5282066e-6
 

 
1.0
 

 
Q058B2
 

 
0.429807
 

 
2.0
 

 
0.07712766
 

 
376
 

 
T
 

 
NIL
 

 

 

 
GKFG-818-MONOMER
 

 
mcu:HMPREF0573_10271 pfkL
 

 
2.5282066e-6
 

 
1.0
 

 
Q1GMM6
 

 
0.0286153
 

 
2.0
 

 
0.048101265
 

 
395
 

 
T
 

 
NIL
 

 

 
     Evidence for succinyldiaminopimelate transaminase, EC# 2.6.1.17   SUCCINYLDIAMINOPIMTRANS-RXN 
  

 

 
 Hit 
 

 
 Common 
 

 
 P 
 

 
 #Q 
 

 
 Best Qry 
 

 
 Best Eval 
 

 
 Avg Rank 
 

 
 Aln Len 
 

 
 Qry Len 
 

 
 IDOP? 
 

 
 adj? 
 

 

 

 
GKFG-999-MONOMER
 

 
mcu:HMPREF0573_10302 hemL
 

 
0.8249092
 

 
2.0
 

 
P57600
 

 
2.13477e-16
 

 
1.0
 

 
0.94813097
 

 
408
 

 
T
 

 
NIL
 

 

 

 
GKFG-364-MONOMER
 

 
mcu:HMPREF0573_11145 aspC
 

 
0.7357018
 

 
1.0
 

 
Q9ZEX3
 

 
2.19626e-16
 

 
1.0
 

 
0.8186398
 

 
397
 

 
NIL
 

 
NIL
 

 

 

 
GKFG-510-MONOMER
 

 
bcv:Bcav_3969 histidinol-
 

 
6.873975e-5
 

 
1.0
 

 
Q9ZEX3
 

 
0.00795467
 

 
2.0
 

 
0.28463477
 

 
397
 

 
NIL
 

 
NIL
 

 

 

 
GKFG-1824-MONOMER
 

 
transmembrane_regions (db
 

 
1.2082764e-6
 

 
1.0
 

 
Q9ZEX3
 

 
0.142699
 

 
3.0
 

 
0.23173803
 

 
397
 

 
T
 

 
NIL
 

 

 
     

  methionine biosynthesis II     Total # of reactions in pathway = 6   Present reactions: 3
  

 

 
 Reaction 
 

 
 Protein(s) 
 

 

 

 
HOMOSERKIN-RXN
 

 
(# )
 

 

 

 
CYSTATHIONINE-BETA-LYASE-RXN
 

 
(# )
 

 

 

 
HOMOCYSMET-RXN
 

 
(# )
 

 

 
   Missing reactions: 3
  

 

 
 CYSPH-RXN 
 

 

 

 
 HOMOCYSTEINE-S-METHYLTRANSFERASE-RXN 
 

 

 

 
 MMUM-RXN 
 

 

 
        Evidence for , EC# 2.5.1.-   CYSPH-RXN 
  

 

 
 Hit 
 

 
 Common 
 

 
 P 
 

 
 #Q 
 

 
 Best Qry 
 

 
 Best Eval 
 

 
 Avg Rank 
 

 
 Aln Len 
 

 
 Qry Len 
 

 
 IDOP? 
 

 
 adj? 
 

 

 

 
GKFG-1592-MONOMER
 

 
iva:Isova_2663 O-acetylho
 

 
0.999776
 

 
1.0
 

 
P55217
 

 
1.12456e-55
 

 
1.0
 

 
0.74422735
 

 
563
 

 
T
 

 
T
 

 

 
     Evidence for S-adenosyl-L-methionine:L-homocysteine S-methyltransferase, EC# 2.1.1.10   HOMOCYSTEINE-S-METHYLTRANSFERASE-RXN 
  
no BLAST hits found
 
     Evidence for homocysteine  S -methyltransferase, EC# 2.1.1.10   MMUM-RXN 
  

 

 
 Hit 
 

 
 Common 
 

 
 P 
 

 
 #Q 
 

 
 Best Qry 
 

 
 Best Eval 
 

 
 Avg Rank 
 

 
 Aln Len 
 

 
 Qry Len 
 

 
 IDOP? 
 

 
 adj? 
 

 

 

 
GKFG-206-MONOMER
 

 
cef:CE1295 respiratory ni
 

 
3.787886e-5
 

 
1.0
 

 
O06745
 

 
0.0946223
 

 
1.0
 

 
0.10130719
 

 
612
 

 
NIL
 

 
NIL
 

 

 

 
GKFG-1991-MONOMER
 

 
ahe:Arch_0927 glycogen sy
 

 
2.5307965e-5
 

 
1.0
 

 
Q8LAX0
 

 
0.871278
 

 
2.0
 

 
0.3054755
 

 
347
 

 
T
 

 
NIL
 

 

 

 
GKFG-1386-MONOMER
 

 
gatA; gata; K02433 aspart
 

 
1.5421621e-5
 

 
1.0
 

 
Q8LAX0
 

 
0.366336
 

 
1.0
 

 
0.12103746
 

 
347
 

 
T
 

 
NIL
 

 

 

 
GKFG-1813-MONOMER
 

 
membrane protein (db=KEGG
 

 
2.5282066e-6
 

 
1.0
 

 
O06745
 

 
0.408125
 

 
2.0
 

 
0.0375817
 

 
612
 

 
T
 

 
NIL
 

 

 

 
GKFG-1819-MONOMER
 

 
ahe:Arch_0813 threonyl-tR
 

 
1.2082764e-6
 

 
1.0
 

 
Q8LAX0
 

 
0.932082
 

 
3.0
 

 
0.15561959
 

 
347
 

 
T
 

 
NIL
 

 

 
     

  methionine biosynthesis I     Total # of reactions in pathway = 5   Present reactions: 3
  

 

 
 Reaction 
 

 
 Protein(s) 
 

 

 

 
HOMOCYSMET-RXN
 

 
(# )
 

 

 

 
CYSTATHIONINE-BETA-LYASE-RXN
 

 
(# )
 

 

 

 
O-SUCCHOMOSERLYASE-RXN
 

 
(# )
 

 

 
   Missing reactions: 2
  

 

 
 HOMSUCTRAN-RXN 
 

 

 

 
 HOMOCYSMETB12-RXN 
 

 

 
        Evidence for homoserine  O -succinyltransferase, EC# 2.3.1.46   HOMSUCTRAN-RXN 
  

 

 
 Hit 
 

 
 Common 
 

 
 P 
 

 
 #Q 
 

 
 Best Qry 
 

 
 Best Eval 
 

 
 Avg Rank 
 

 
 Aln Len 
 

 
 Qry Len 
 

 
 IDOP? 
 

 
 adj? 
 

 

 

 
GKFG-1966-MONOMER
 

 
mcu:HMPREF0573_11649 hypo
 

 
0.0051016076
 

 
1.0
 

 
Q5N2Y8
 

 
0.00258355
 

 
1.0
 

 
0.6354515
 

 
299
 

 
T
 

 
NIL
 

 

 

 
GKFG-1543-MONOMER
 

 
UBA/ThiF-type NAD/FAD bin
 

 
1.3945618e-5
 

 
1.0
 

 
A5V3T8
 

 
0.830203
 

 
1.0
 

 
0.100946374
 

 
317
 

 
T
 

 
NIL
 

 

 

 
GKFG-1511-MONOMER
 

 
cga:Celgi_1503 glycogen d
 

 
1.3945618e-5
 

 
1.0
 

 
A3QG19
 

 
0.99092
 

 
1.0
 

 
0.115264796
 

 
321
 

 
T
 

 
NIL
 

 

 
     Evidence for methionine synthase, EC# 2.1.1.13   HOMOCYSMETB12-RXN 
  

 

 
 Hit 
 

 
 Common 
 

 
 P 
 

 
 #Q 
 

 
 Best Qry 
 

 
 Best Eval 
 

 
 Avg Rank 
 

 
 Aln Len 
 

 
 Qry Len 
 

 
 IDOP? 
 

 
 adj? 
 

 

 

 
GKFG-1062-MONOMER
 

 
mcu:HMPREF0573_10372 rho;
 

 
1.3945618e-5
 

 
1.0
 

 
Q49775
 

 
0.789321
 

 
1.0
 

 
0.04477612
 

 
1206
 

 
T
 

 
NIL
 

 

 

 
GKFG-1196-MONOMER
 

 
rha:RHA1_ro05116 sugar AB
 

 
1.3945618e-5
 

 
1.0
 

 
P13009
 

 
0.0662266
 

 
1.0
 

 
0.05378973
 

 
1227
 

 
T
 

 
NIL
 

 

 

 
GKFG-1944-MONOMER
 

 
bcv:Bcav_2184 excinucleas
 

 
2.5282066e-6
 

 
1.0
 

 
P13009
 

 
0.333957
 

 
2.0
 

 
0.051344745
 

 
1227
 

 
T
 

 
NIL
 

 

 

 
GKFG-1242-MONOMER
 

 
cfi:Celf_2761 alpha amyla
 

 
1.0926308e-6
 

 
1.0
 

 
P13009
 

 
0.624379
 

 
3.0
 

 
0.072534636
 

 
1227
 

 
T
 

 
NIL
 

 

 
     

   S -adenosyl-L-methionine cycle I     Total # of reactions in pathway = 5   Present reactions: 4
  

 

 
 Reaction 
 

 
 Protein(s) 
 

 

 

 
ADENOSYLHOMOCYSTEINE-NUCLEOSIDASE-RXN
 

 
(# )
 

 

 

 
RIBOSYLHOMOCYSTEINASE-RXN
 

 
(# )
 

 

 

 
SAM-PWY
 

 
NIL
 

 

 

 
HOMOCYSMET-RXN
 

 
(# )
 

 

 
   Missing reactions: 1
  

 

 
 RXN-7605 
 

 

 
        Evidence for , EC# 2.1.1.-   RXN-7605      

  ornithine biosynthesis     Total # of reactions in pathway = 5   Present reactions: 4
  

 

 
 Reaction 
 

 
 Protein(s) 
 

 

 

 
N-ACETYLTRANSFER-RXN
 

 
(# )
 

 

 

 
ACETYLGLUTKIN-RXN
 

 
(# )
 

 

 

 
N-ACETYLGLUTPREDUCT-RXN
 

 
(# )
 

 

 

 
ACETYLORNTRANSAM-RXN
 

 
(# )
 

 

 
   Missing reactions: 1
  

 

 
 ACETYLORNDEACET-RXN 
 

 

 
        Evidence for acetylornithine deacetylase, EC# 3.5.1.16   ACETYLORNDEACET-RXN 
  

 

 
 Hit 
 

 
 Common 
 

 
 P 
 

 
 #Q 
 

 
 Best Qry 
 

 
 Best Eval 
 

 
 Avg Rank 
 

 
 Aln Len 
 

 
 Qry Len 
 

 
 IDOP? 
 

 
 adj? 
 

 

 

 
GKFG-97-MONOMER
 

 
mcu:HMPREF0573_10969 rpoC
 

 
0.92492604
 

 
1.0
 

 
P13440
 

 
1.76629e-57
 

 
1.0
 

 
0.68231046
 

 
277
 

 
NIL
 

 
NIL
 

 

 

 
GKFG-1395-MONOMER
 

 
RNA-binding S4 domain-con
 

 
2.5307965e-5
 

 
1.0
 

 
P23908
 

 
0.0393783
 

 
2.0
 

 
0.26370758
 

 
383
 

 
T
 

 
NIL
 

 

 

 
GKFG-615-MONOMER
 

 
ahe:Arch_0744 peptidase M
 

 
1.5421621e-5
 

 
4.0
 

 
P23908
 

 
7.41004e-9
 

 
1.0
 

 
0.21875805
 

 
383
 

 
T
 

 
NIL
 

 

 
     

  proline biosynthesis II (from arginine)     Total # of reactions in pathway = 5   Present reactions: 3
  

 

 
 Reaction 
 

 
 Protein(s) 
 

 

 

 
ARGININE-DEIMINASE-RXN
 

 
(# )
 

 

 

 
ORNCARBAMTRANSFER-RXN
 

 
(# )
 

 

 

 
PYRROLINECARBREDUCT-RXN
 

 
(# )
 

 

 
   Missing reactions: 2
  

 

 
 ORNITHINE-GLU-AMINOTRANSFORASE-RXN 
 

 

 

 
 CITRULLINASE-RXN 
 

 

 
        Evidence for Ornithine--oxo-glutarate aminotransferase, EC# 2.6.1.13   ORNITHINE-GLU-AMINOTRANSFORASE-RXN 
  

 

 
 Hit 
 

 
 Common 
 

 
 P 
 

 
 #Q 
 

 
 Best Qry 
 

 
 Best Eval 
 

 
 Avg Rank 
 

 
 Aln Len 
 

 
 Qry Len 
 

 
 IDOP? 
 

 
 adj? 
 

 

 

 
GKFG-999-MONOMER
 

 
mcu:HMPREF0573_10302 hemL
 

 
0.5061234
 

 
1.0
 

 
P07991
 

 
3.8037e-17
 

 
1.0
 

 
0.8089623
 

 
424
 

 
T
 

 
NIL
 

 

 
     Evidence for citrullinase, EC# 3.5.1.20   CITRULLINASE-RXN      

  serine biosynthesis     Total # of reactions in pathway = 3   Present reactions: 2
  

 

 
 Reaction 
 

 
 Protein(s) 
 

 

 

 
PSERTRANSAM-RXN
 

 
(# )
 

 

 

 
PGLYCDEHYDROG-RXN
 

 
(# )
 

 

 
   Missing reactions: 1
  

 

 
 RXN0-5114 
 

 

 
        Evidence for , EC# 3.1.3.3   RXN0-5114 
  

 

 
 Hit 
 

 
 Common 
 

 
 P 
 

 
 #Q 
 

 
 Best Qry 
 

 
 Best Eval 
 

 
 Avg Rank 
 

 
 Aln Len 
 

 
 Qry Len 
 

 
 IDOP? 
 

 
 adj? 
 

 

 

 
GKFG-178-MONOMER
 

 
HAD superfamily hydrolase
 

 
3.7905585e-4
 

 
1.0
 

 
P0AGB0
 

 
1.08184e-4
 

 
1.0
 

 
0.35714287
 

 
322
 

 
NIL
 

 
NIL
 

 

 

 
GKFG-410-MONOMER
 

 
hydrolase of the HAD supe
 

 
7.594039e-6
 

 
1.0
 

 
P0AGB0
 

 
0.00472669
 

 
2.0
 

 
0.12111801
 

 
322
 

 
NIL
 

 
NIL
 

 

 

 
GKFG-281-MONOMER
 

 
bfa:Bfae_19800 copper/sil
 

 
3.2819723e-6
 

 
2.0
 

 
P0AGB0
 

 
0.378781
 

 
2.5
 

 
0.19654244
 

 
322
 

 
NIL
 

 
NIL
 

 

 

 
GKFG-101-MONOMER
 

 
hydrolase of the HAD supe
 

 
3.2819723e-6
 

 
1.0
 

 
P0AGB0
 

 
0.00472669
 

 
3.0
 

 
0.12111801
 

 
322
 

 
NIL
 

 
NIL
 

 

 

 
GKFG-1963-MONOMER
 

 
HAD-superfamily hydrolase
 

 
2.795795e-6
 

 
1.0
 

 
Q5M819
 

 
0.70472
 

 
2.0
 

 
0.15111111
 

 
225
 

 
T
 

 
NIL
 

 

 
     

  tryptophan biosynthesis     Total # of reactions in pathway = 6   Present reactions: 2
  

 

 
 Reaction 
 

 
 Protein(s) 
 

 

 

 
IGPSYN-RXN
 

 
(# )
 

 

 

 
PRAISOM-RXN
 

 
(# )
 

 

 
   Missing reactions: 4
  

 

 
 PRTRANS-RXN 
 

 

 

 
 ANTHRANSYN-RXN 
 

 

 

 
 RXN0-2382 
 

 

 

 
 RXN0-2381 
 

 

 
        Evidence for anthranilate phosphoribosyltransferase, EC# 2.4.2.18   PRTRANS-RXN 
  

 

 
 Hit 
 

 
 Common 
 

 
 P 
 

 
 #Q 
 

 
 Best Qry 
 

 
 Best Eval 
 

 
 Avg Rank 
 

 
 Aln Len 
 

 
 Qry Len 
 

 
 IDOP? 
 

 
 adj? 
 

 

 

 
GKFG-1952-MONOMER
 

 
UniRef90_E8JFA8 Indole-3-
 

 
0.33294967
 

 
1.0
 

 
O28668
 

 
6.49504e-14
 

 
1.0
 

 
0.4010989
 

 
546
 

 
T
 

 
NIL
 

 

 

 
GKFG-865-MONOMER
 

 
bfa:Bfae_24100 IMP cycloh
 

 
3.182795e-4
 

 
1.0
 

 
O66576
 

 
0.0197052
 

 
1.0
 

 
0.40882352
 

 
340
 

 
T
 

 
NIL
 

 

 

 
GKFG-1833-MONOMER
 

 
bcv:Bcav_2151 prephenate 
 

 
3.182795e-4
 

 
1.0
 

 
B3QUY6
 

 
0.551946
 

 
1.0
 

 
0.4098837
 

 
344
 

 
T
 

 
NIL
 

 

 

 
GKFG-885-MONOMER
 

 
srt:Srot_1167 terminase (
 

 
3.182795e-4
 

 
1.0
 

 
Q88WI3
 

 
0.624007
 

 
1.0
 

 
0.4100295
 

 
339
 

 
T
 

 
NIL
 

 

 

 
GKFG-838-MONOMER
 

 
bcv:Bcav_1314 N-acetylglu
 

 
3.182795e-4
 

 
1.0
 

 
Q5WGS4
 

 
0.742822
 

 
1.0
 

 
0.36070383
 

 
341
 

 
T
 

 
NIL
 

 

 

 
GKFG-1456-MONOMER
 

 
ahe:Arch_1237 peptidase M
 

 
1.3958463e-4
 

 
2.0
 

 
Q0BJW6
 

 
0.106925
 

 
1.0
 

 
0.27038175
 

 
337
 

 
T
 

 
NIL
 

 

 

 
GKFG-1620-MONOMER
 

 
bcv:Bcav_1262 GTPase EngC
 

 
1.3958463e-4
 

 
1.0
 

 
Q6L273
 

 
0.0759679
 

 
1.0
 

 
0.3148148
 

 
324
 

 
T
 

 
NIL
 

 

 

 
GKFG-1054-MONOMER
 

 
hypothetical protein (db=
 

 
1.3958463e-4
 

 
1.0
 

 
A1SLE8
 

 
0.0613233
 

 
1.0
 

 
0.24926686
 

 
341
 

 
T
 

 
NIL
 

 

 

 
GKFG-666-MONOMER
 

 
DNA protecting protein Dp
 

 
1.3958463e-4
 

 
1.0
 

 
Q24SK4
 

 
0.0682923
 

 
1.0
 

 
0.26190478
 

 
336
 

 
T
 

 
NIL
 

 

 

 
GKFG-1985-MONOMER
 

 
seg (db=Seg db_id=seg fro
 

 
1.3958463e-4
 

 
1.0
 

 
Q46WU8
 

 
0.623601
 

 
1.0
 

 
0.25203252
 

 
369
 

 
T
 

 
NIL
 

 

 

 
GKFG-1741-MONOMER
 

 
ABC transporter ATP-bindi
 

 
1.3958463e-4
 

 
1.0
 

 
A8FEK1
 

 
0.445843
 

 
1.0
 

 
0.28235295
 

 
340
 

 
T
 

 
NIL
 

 

 

 
GKFG-1883-MONOMER
 

 
transmembrane_regions (db
 

 
5.771539e-5
 

 
2.0
 

 
O68608
 

 
0.30369
 

 
1.5
 

 
0.36140743
 

 
354
 

 
T
 

 
NIL
 

 

 

 
GKFG-354-MONOMER
 

 
UniRef90_E6KU01 MIP famil
 

 
4.188785e-5
 

 
2.0
 

 
Q604F8
 

 
0.0415558
 

 
1.0
 

 
0.15151654
 

 
341
 

 
NIL
 

 
NIL
 

 

 

 
GKFG-136-MONOMER
 

 
hypothetical protein (db=
 

 
4.188785e-5
 

 
2.0
 

 
A5UT95
 

 
0.398955
 

 
1.0
 

 
0.18191916
 

 
341
 

 
NIL
 

 
NIL
 

 

 

 
GKFG-97-MONOMER
 

 
mcu:HMPREF0573_10969 rpoC
 

 
4.188785e-5
 

 
1.0
 

 
Q2NFE4
 

 
0.687433
 

 
1.0
 

 
0.16045845
 

 
349
 

 
NIL
 

 
NIL
 

 

 

 
GKFG-86-MONOMER
 

 
Putative uncharacterized 
 

 
4.188785e-5
 

 
1.0
 

 
Q5QX82
 

 
0.183148
 

 
1.0
 

 
0.15133531
 

 
337
 

 
NIL
 

 
NIL
 

 

 

 
GKFG-153-MONOMER
 

 
mcu:HMPREF0573_10425 ppdK
 

 
4.188785e-5
 

 
1.0
 

 
Q1CRX3
 

 
0.224922
 

 
1.0
 

 
0.2238806
 

 
335
 

 
NIL
 

 
NIL
 

 

 

 
GKFG-536-MONOMER
 

 
mcu:HMPREF0573_10685 gyrB
 

 
4.188785e-5
 

 
1.0
 

 
Q47YC2
 

 
0.0334908
 

 
1.0
 

 
0.1780822
 

 
365
 

 
NIL
 

 
NIL
 

 

 

 
GKFG-151-MONOMER
 

 
cga:Celgi_0340 FAD linked
 

 
4.188785e-5
 

 
1.0
 

 
Q9Y8T2
 

 
0.0216275
 

 
1.0
 

 
0.16811594
 

 
345
 

 
NIL
 

 
NIL
 

 

 

 
GKFG-442-MONOMER
 

 
mcu:HMPREF0573_10293 glmS
 

 
4.188785e-5
 

 
1.0
 

 
Q7NGU2
 

 
0.287361
 

 
1.0
 

 
0.1374269
 

 
342
 

 
NIL
 

 
NIL
 

 

 

 
GKFG-874-MONOMER
 

 
rer:RER_18960 phage porta
 

 
2.5307965e-5
 

 
1.0
 

 
Q7NGU2
 

 
0.789253
 

 
2.0
 

 
0.32163742
 

 
342
 

 
T
 

 
NIL
 

 

 

 
GKFG-791-MONOMER
 

 
iva:Isova_1606 carbamoyl-
 

 
1.5421621e-5
 

 
3.0
 

 
Q08654
 

 
1.71991e-10
 

 
1.0
 

 
0.2015652
 

 
589
 

 
T
 

 
NIL
 

 

 

 
GKFG-602-MONOMER
 

 
cfi:Celf_2170 phospho-2-d
 

 
1.5421621e-5
 

 
2.0
 

 
Q2RT49
 

 
0.348495
 

 
1.0
 

 
0.23938212
 

 
347
 

 
T
 

 
NIL
 

 

 

 
GKFG-809-MONOMER
 

 
ahe:Arch_0180 ATP-depende
 

 
1.5421621e-5
 

 
2.0
 

 
Q31J09
 

 
0.850856
 

 
1.0
 

 
0.202799
 

 
340
 

 
T
 

 
NIL
 

 

 

 
GKFG-1389-MONOMER
 

 
transmembrane_regions (db
 

 
1.5421621e-5
 

 
2.0
 

 
B4UHD1
 

 
0.0127508
 

 
1.0
 

 
0.1886405
 

 
337
 

 
T
 

 
NIL
 

 

 

 
GKFG-591-MONOMER
 

 
type I phosphodiesterase/
 

 
1.5421621e-5
 

 
1.0
 

 
B1I3Z8
 

 
0.487999
 

 
1.0
 

 
0.19653179
 

 
346
 

 
T
 

 
NIL
 

 

 

 
GKFG-632-MONOMER
 

 
rdn:HMPREF0733_11588 dinG
 

 
1.5421621e-5
 

 
1.0
 

 
Q12LE4
 

 
0.388437
 

 
1.0
 

 
0.1468144
 

 
361
 

 
T
 

 
NIL
 

 

 

 
GKFG-769-MONOMER
 

 
bcv:Bcav_0884 UspA domain
 

 
1.5421621e-5
 

 
1.0
 

 
B7IM73
 

 
0.197723
 

 
1.0
 

 
0.14662756
 

 
341
 

 
T
 

 
NIL
 

 

 

 
GKFG-781-MONOMER
 

 
mcu:HMPREF0573_10492 NCS2
 

 
1.5421621e-5
 

 
1.0
 

 
A1R6T5
 

 
0.590062
 

 
1.0
 

 
0.19602273
 

 
352
 

 
T
 

 
NIL
 

 

 

 
GKFG-867-MONOMER
 

 
ahe:Arch_1295 succinyl-Co
 

 
1.5421621e-5
 

 
1.0
 

 
Q1GYF5
 

 
0.319181
 

 
1.0
 

 
0.19061583
 

 
341
 

 
T
 

 
NIL
 

 

 

 
GKFG-1633-MONOMER
 

 
mcu:HMPREF0573_10221 pta;
 

 
1.5421621e-5
 

 
1.0
 

 
Q1CZH6
 

 
0.166062
 

 
1.0
 

 
0.147929
 

 
338
 

 
T
 

 
NIL
 

 

 

 
GKFG-1149-MONOMER
 

 
pac:PPA1589 hypothetical 
 

 
1.5421621e-5
 

 
1.0
 

 
B8DM45
 

 
0.0106187
 

 
1.0
 

 
0.21893491
 

 
338
 

 
T
 

 
NIL
 

 

 

 
GKFG-833-MONOMER
 

 
mlu:Mlut_15870 hypothetic
 

 
1.5421621e-5
 

 
1.0
 

 
A1KTN4
 

 
0.114344
 

 
1.0
 

 
0.21875
 

 
352
 

 
T
 

 
NIL
 

 

 

 
GKFG-732-MONOMER
 

 
ahe:Arch_1078 LuxR family
 

 
1.5421621e-5
 

 
1.0
 

 
Q8R9M6
 

 
0.453078
 

 
1.0
 

 
0.14749263
 

 
339
 

 
T
 

 
NIL
 

 

 

 
GKFG-1794-MONOMER
 

 
iva:Isova_1635 aspartyl-t
 

 
1.5421621e-5
 

 
1.0
 

 
Q8Y6Q3
 

 
0.225341
 

 
1.0
 

 
0.2359882
 

 
339
 

 
T
 

 
NIL
 

 

 

 
GKFG-1958-MONOMER
 

 
cga:Celgi_1839 hypothetic
 

 
1.3945618e-5
 

 
1.0
 

 
A1KKN4
 

 
0.858963
 

 
1.0
 

 
0.097297296
 

 
370
 

 
T
 

 
NIL
 

 

 

 
GKFG-1052-MONOMER
 

 
142523..142807 - ( gc_con
 

 
1.3945618e-5
 

 
1.0
 

 
Q28R66
 

 
0.19904
 

 
1.0
 

 
0.104347825
 

 
345
 

 
T
 

 
NIL
 

 

 

 
GKFG-1191-MONOMER
 

 
pfr:PFREUD_12970 glpC; an
 

 
1.3945618e-5
 

 
1.0
 

 
C3MV89
 

 
0.100737
 

 
1.0
 

 
0.08695652
 

 
345
 

 
T
 

 
NIL
 

 

 

 
GKFG-814-MONOMER
 

 
ahe:Arch_0168 chaperone p
 

 
1.3945618e-5
 

 
1.0
 

 
Q0RFI3
 

 
0.379389
 

 
1.0
 

 
0.07317073
 

 
369
 

 
T
 

 
NIL
 

 

 

 
GKFG-977-MONOMER
 

 
paz:TIA2EST2_02200 methyl
 

 
1.3945618e-5
 

 
1.0
 

 
C4ZI69
 

 
0.424764
 

 
1.0
 

 
0.088757396
 

 
338
 

 
T
 

 
NIL
 

 

 

 
GKFG-329-MONOMER
 

 
mcu:HMPREF0573_11679 opuE
 

 
7.594039e-6
 

 
1.0
 

 
B8DM45
 

 
0.465485
 

 
2.0
 

 
0.12426036
 

 
338
 

 
NIL
 

 
NIL
 

 

 

 
GKFG-515-MONOMER
 

 
DedA family protein; K039
 

 
4.3885325e-6
 

 
1.0
 

 
O28668
 

 
0.139429
 

 
4.0
 

 
0.13553114
 

 
546
 

 
NIL
 

 
NIL
 

 

 

 
GKFG-378-MONOMER
 

 
bbp:BBPR_0460 hypothetica
 

 
3.2819723e-6
 

 
1.0
 

 
O68608
 

 
0.374955
 

 
3.0
 

 
0.1779661
 

 
354
 

 
NIL
 

 
NIL
 

 

 

 
GKFG-1603-MONOMER
 

 
xce:Xcel_0763 hypothetica
 

 
2.795795e-6
 

 
2.0
 

 
A9F0C1
 

 
0.660281
 

 
1.5
 

 
0.15151958
 

 
348
 

 
T
 

 
NIL
 

 

 

 
GKFG-1017-MONOMER
 

 
lipoprotein LpqB (db=KEGG
 

 
2.795795e-6
 

 
1.0
 

 
Q28R66
 

 
0.410356
 

 
2.0
 

 
0.16521738
 

 
345
 

 
T
 

 
NIL
 

 

 

 
GKFG-1573-MONOMER
 

 
Putative uncharacterized 
 

 
2.795795e-6
 

 
1.0
 

 
Q5QX82
 

 
0.225965
 

 
2.0
 

 
0.15133531
 

 
337
 

 
T
 

 
NIL
 

 

 

 
GKFG-1402-MONOMER
 

 
riboflavin biosynthesis p
 

 
2.795795e-6
 

 
1.0
 

 
Q2JQ65
 

 
0.867618
 

 
2.0
 

 
0.14868805
 

 
343
 

 
T
 

 
NIL
 

 

 

 
GKFG-1018-MONOMER
 

 
mcu:HMPREF0573_10409 hist
 

 
2.795795e-6
 

 
1.0
 

 
O28668
 

 
0.0343625
 

 
2.0
 

 
0.21794872
 

 
546
 

 
T
 

 
NIL
 

 

 

 
GKFG-1308-MONOMER
 

 
hypothetical protein (db=
 

 
2.795795e-6
 

 
1.0
 

 
Q604F8
 

 
0.474091
 

 
2.0
 

 
0.17595308
 

 
341
 

 
T
 

 
NIL
 

 

 

 
GKFG-1551-MONOMER
 

 
threonine dehydratase (EC
 

 
2.5282066e-6
 

 
1.0
 

 
A1KTN4
 

 
0.407746
 

 
2.0
 

 
0.09090909
 

 
352
 

 
T
 

 
NIL
 

 

 

 
GKFG-681-MONOMER
 

 
archaeal fructose-1,6-bis
 

 
1.2082764e-6
 

 
1.0
 

 
Q28R66
 

 
0.557165
 

 
3.0
 

 
0.1536232
 

 
345
 

 
T
 

 
NIL
 

 

 

 
GKFG-1960-MONOMER
 

 
mcu:HMPREF0573_10477 adhC
 

 
1.2082764e-6
 

 
1.0
 

 
O28668
 

 
0.0859169
 

 
3.0
 

 
0.15384616
 

 
546
 

 
T
 

 
NIL
 

 

 
     Evidence for anthranilate synthase, EC# 4.1.3.27   ANTHRANSYN-RXN 
  

 

 
 Hit 
 

 
 Common 
 

 
 P 
 

 
 #Q 
 

 
 Best Qry 
 

 
 Best Eval 
 

 
 Avg Rank 
 

 
 Aln Len 
 

 
 Qry Len 
 

 
 IDOP? 
 

 
 adj? 
 

 

 

 
GKFG-791-MONOMER
 

 
iva:Isova_1606 carbamoyl-
 

 
0.57070076
 

 
23.0
 

 
P26922
 

 
2.80717e-13
 

 
1.173913
 

 
0.55761045
 

 
196
 

 
T
 

 
NIL
 

 

 

 
GKFG-1952-MONOMER
 

 
UniRef90_E8JFA8 Indole-3-
 

 
0.508736
 

 
5.0
 

 
P05328
 

 
1.03117e-44
 

 
1.0
 

 
0.3027454
 

 
770
 

 
T
 

 
NIL
 

 

 

 
GKFG-991-MONOMER
 

 
mcu:HMPREF0573_11504 hypo
 

 
0.0018147272
 

 
1.0
 

 
P00896
 

 
0.0771573
 

 
1.0
 

 
0.58666664
 

 
150
 

 
T
 

 
NIL
 

 

 

 
GKFG-387-MONOMER
 

 
cgt:cgR_2367 hypothetical
 

 
1.5675386e-4
 

 
1.0
 

 
P44339
 

 
0.472521
 

 
2.0
 

 
0.39378238
 

 
193
 

 
NIL
 

 
NIL
 

 

 

 
GKFG-1242-MONOMER
 

 
cfi:Celf_2761 alpha amyla
 

 
1.3958463e-4
 

 
1.0
 

 
Q9YGB3
 

 
0.0310971
 

 
1.0
 

 
0.28637412
 

 
433
 

 
T
 

 
NIL
 

 

 

 
GKFG-413-MONOMER
 

 
UniRef90_F3P5U3 Putative 
 

 
6.873975e-5
 

 
1.0
 

 
P09786
 

 
0.668365
 

 
2.0
 

 
0.3
 

 
200
 

 
NIL
 

 
NIL
 

 

 

 
GKFG-75-MONOMER
 

 
Bacterial adhesins (db=su
 

 
4.188785e-5
 

 
1.0
 

 
P21690
 

 
0.0529551
 

 
1.0
 

 
0.16597511
 

 
482
 

 
NIL
 

 
NIL
 

 

 

 
GKFG-1819-MONOMER
 

 
ahe:Arch_0813 threonyl-tR
 

 
1.5421621e-5
 

 
1.0
 

 
P09785
 

 
0.115279
 

 
1.0
 

 
0.13018867
 

 
530
 

 
T
 

 
NIL
 

 

 

 
GKFG-1859-MONOMER
 

 
mcu:HMPREF0573_10789 GTP-
 

 
1.5421621e-5
 

 
1.0
 

 
P96556
 

 
0.183856
 

 
1.0
 

 
0.16572505
 

 
531
 

 
T
 

 
NIL
 

 

 

 
GKFG-1962-MONOMER
 

 
hypothetical protein; K07
 

 
1.5421621e-5
 

 
1.0
 

 
Q9HS66
 

 
0.988837
 

 
1.0
 

 
0.18852459
 

 
488
 

 
T
 

 
NIL
 

 

 

 
GKFG-1852-MONOMER
 

 
seg (db=Seg db_id=seg fro
 

 
1.3945618e-5
 

 
1.0
 

 
P03963
 

 
0.614303
 

 
1.0
 

 
0.08932039
 

 
515
 

 
T
 

 
NIL
 

 

 

 
GKFG-724-MONOMER
 

 
serine/threonine protein 
 

 
2.795795e-6
 

 
1.0
 

 
P00896
 

 
0.401356
 

 
2.0
 

 
0.22
 

 
150
 

 
T
 

 
NIL
 

 

 

 
GKFG-1883-MONOMER
 

 
transmembrane_regions (db
 

 
2.795795e-6
 

 
1.0
 

 
Q7DAK6
 

 
0.854305
 

 
2.0
 

 
0.12931034
 

 
232
 

 
T
 

 
NIL
 

 

 

 
GKFG-1154-MONOMER
 

 
phage protein (db=KEGG ev
 

 
2.795795e-6
 

 
1.0
 

 
Q5V632
 

 
0.795383
 

 
2.0
 

 
0.23157895
 

 
190
 

 
T
 

 
NIL
 

 

 

 
GKFG-1391-MONOMER
 

 
peptide methionine sulfox
 

 
2.795795e-6
 

 
1.0
 

 
P21690
 

 
0.463338
 

 
2.0
 

 
0.1680498
 

 
482
 

 
T
 

 
NIL
 

 

 

 
GKFG-854-MONOMER
 

 
LPXTG_anchor: LPXTG-motif
 

 
2.5282066e-6
 

 
1.0
 

 
P15395
 

 
0.0198015
 

 
2.0
 

 
0.07133059
 

 
729
 

 
T
 

 
NIL
 

 

 

 
GKFG-1603-MONOMER
 

 
xce:Xcel_0763 hypothetica
 

 
2.5282066e-6
 

 
1.0
 

 
P00904
 

 
0.968607
 

 
2.0
 

 
0.09039548
 

 
531
 

 
T
 

 
NIL
 

 

 

 
GKFG-901-MONOMER
 

 
UniRef90_D0WNZ8 Electron 
 

 
1.6156636e-6
 

 
1.0
 

 
P15395
 

 
0.762665
 

 
4.0
 

 
0.14128944
 

 
729
 

 
T
 

 
NIL
 

 

 

 
GKFG-632-MONOMER
 

 
rdn:HMPREF0733_11588 dinG
 

 
1.0926308e-6
 

 
1.0
 

 
P20409
 

 
0.105754
 

 
3.0
 

 
0.044444446
 

 
765
 

 
T
 

 
NIL
 

 

 

 
GKFG-1429-MONOMER
 

 
mcu:HMPREF0573_10785 hydr
 

 
1.0926308e-6
 

 
1.0
 

 
P15395
 

 
0.374319
 

 
3.0
 

 
0.07133059
 

 
729
 

 
T
 

 
NIL
 

 

 

 
GKFG-1961-MONOMER
 

 
ske:Sked_20910 tRNA (aden
 

 
1.0926308e-6
 

 
1.0
 

 
P27710
 

 
0.591272
 

 
3.0
 

 
0.07446808
 

 
752
 

 
T
 

 
NIL
 

 

 

 
GKFG-746-MONOMER
 

 
two-component system hist
 

 
1.0926308e-6
 

 
1.0
 

 
Q92370
 

 
0.736275
 

 
3.0
 

 
0.11462451
 

 
759
 

 
T
 

 
NIL
 

 

 
     Evidence for tryptophan synthase (indole-salvaging), EC# 4.2.1.122   RXN0-2382 
  

 

 
 Hit 
 

 
 Common 
 

 
 P 
 

 
 #Q 
 

 
 Best Qry 
 

 
 Best Eval 
 

 
 Avg Rank 
 

 
 Aln Len 
 

 
 Qry Len 
 

 
 IDOP? 
 

 
 adj? 
 

 

 

 
GKFG-966-MONOMER
 

 
cfi:Celf_1658 pyridoxal-p
 

 
0.8538447
 

 
2.0
 

 
P14671
 

 
2.84673e-29
 

 
1.0
 

 
0.897591
 

 
470
 

 
T
 

 
NIL
 

 

 

 
GKFG-1551-MONOMER
 

 
threonine dehydratase (EC
 

 
2.795795e-6
 

 
1.0
 

 
P14671
 

 
1.5528e-4
 

 
2.0
 

 
0.20638297
 

 
470
 

 
T
 

 
NIL
 

 

 

 
GKFG-1065-MONOMER
 

 
mcu:HMPREF0573_10379 thrC
 

 
1.2082764e-6
 

 
1.0
 

 
P14671
 

 
0.949174
 

 
3.0
 

 
0.18297872
 

 
470
 

 
T
 

 
NIL
 

 

 
     Evidence for indole-3-glycerol-phosphate lyase, EC# 4.1.2.8   RXN0-2381 
  

 

 
 Hit 
 

 
 Common 
 

 
 P 
 

 
 #Q 
 

 
 Best Qry 
 

 
 Best Eval 
 

 
 Avg Rank 
 

 
 Aln Len 
 

 
 Qry Len 
 

 
 IDOP? 
 

 
 adj? 
 

 

 

 
GKFG-1952-MONOMER
 

 
UniRef90_E8JFA8 Indole-3-
 

 
0.0015685969
 

 
2.0
 

 
P42390
 

 
0.0179164
 

 
1.5
 

 
0.14933309
 

 
347
 

 
T
 

 
T
 

 

 

 
GKFG-186-MONOMER
 

 
mcu:HMPREF0573_11467 tatC
 

 
3.787886e-5
 

 
1.0
 

 
Q9FQ77
 

 
0.00495843
 

 
1.0
 

 
0.07964602
 

 
339
 

 
NIL
 

 
NIL
 

 

 

 
GKFG-1491-MONOMER
 

 
bbv:HMPREF9228_0173 thiaz
 

 
2.5307965e-5
 

 
4.0
 

 
Q9YGA9
 

 
0.11892
 

 
2.0
 

 
0.27809304
 

 
251
 

 
T
 

 
NIL
 

 

 

 
GKFG-1954-MONOMER
 

 
bcv:Bcav_2235 Imidazole g
 

 
1.09376415e-5
 

 
3.0
 

 
Q9FQ77
 

 
0.142057
 

 
2.6666667
 

 
0.26271868
 

 
339
 

 
T
 

 
NIL
 

 

 

 
GKFG-566-MONOMER
 

 
cfl:Cfla_1720 deoxyxylulo
 

 
2.5282066e-6
 

 
1.0
 

 
P0A877
 

 
0.582986
 

 
2.0
 

 
0.10074627
 

 
268
 

 
T
 

 
NIL
 

 

 
     

  tyrosine biosynthesis I     Total # of reactions in pathway = 3   Present reactions: 2
  

 

 
 Reaction 
 

 
 Protein(s) 
 

 

 

 
PREPHENATEDEHYDROG-RXN
 

 
(# )
 

 

 

 
CHORISMATEMUT-RXN
 

 
(# )
 

 

 
   Missing reactions: 1
  

 

 
 TYRAMINOTRANS-RXN 
 

 

 
        Evidence for Tyrosine transaminase, EC# 2.6.1.57   TYRAMINOTRANS-RXN 
  

 

 
 Hit 
 

 
 Common 
 

 
 P 
 

 
 #Q 
 

 
 Best Qry 
 

 
 Best Eval 
 

 
 Avg Rank 
 

 
 Aln Len 
 

 
 Qry Len 
 

 
 IDOP? 
 

 
 adj? 
 

 

 

 
GKFG-510-MONOMER
 

 
bcv:Bcav_3969 histidinol-
 

 
3.7905585e-4
 

 
1.0
 

 
P04693
 

 
0.54132
 

 
1.0
 

 
0.24685138
 

 
397
 

 
NIL
 

 
NIL
 

 

 
     

  glycogen degradation I     Total # of reactions in pathway = 7   Present reactions: 6
  

 

 
 Reaction 
 

 
 Protein(s) 
 

 

 

 
AMYLOMALT-RXN
 

 
(# )
 

 

 

 
GLYCOPHOSPHORYL-RXN
 

 
(# )
 

 

 

 
PHOSPHOGLUCMUT-RXN
 

 
(# )
 

 

 

 
RXN0-5182
 

 
(# )
 

 

 

 
RXN0-5183
 

 
(# )
 

 

 

 
GLUCOKIN-RXN
 

 
(# )
 

 

 
   Missing reactions: 1
  

 

 
 RXN0-5146 
 

 

 
        Evidence for , EC# 3.2.1.33   RXN0-5146 
  

 

 
 Hit 
 

 
 Common 
 

 
 P 
 

 
 #Q 
 

 
 Best Qry 
 

 
 Best Eval 
 

 
 Avg Rank 
 

 
 Aln Len 
 

 
 Qry Len 
 

 
 IDOP? 
 

 
 adj? 
 

 

 

 
GKFG-1248-MONOMER
 

 
cfi:Celf_2759 glycogen de
 

 
0.95115626
 

 
1.0
 

 
P15067
 

 
8.65997e-149
 

 
1.0
 

 
1.0
 

 
657
 

 
T
 

 
NIL
 

 

 

 
GKFG-1511-MONOMER
 

 
cga:Celgi_1503 glycogen d
 

 
0.4018846
 

 
1.0
 

 
P15067
 

 
7.0995e-124
 

 
2.0
 

 
0.7305936
 

 
657
 

 
T
 

 
NIL
 

 

 

 
GKFG-1290-MONOMER
 

 
vma:VAB18032_29276 alpha 
 

 
0.10432537
 

 
1.0
 

 
P15067
 

 
1.07439e-7
 

 
6.0
 

 
0.3652968
 

 
657
 

 
T
 

 
T
 

 

 

 
GKFG-1234-MONOMER
 

 
mcu:HMPREF0573_11119 glgB
 

 
0.021276189
 

 
1.0
 

 
P15067
 

 
3.15144e-13
 

 
4.0
 

 
0.5129376
 

 
657
 

 
T
 

 
NIL
 

 

 

 
GKFG-1241-MONOMER
 

 
ahe:Arch_0385 trehalose s
 

 
0.0014584069
 

 
1.0
 

 
P15067
 

 
4.1629e-9
 

 
5.0
 

 
0.651446
 

 
657
 

 
T
 

 
NIL
 

 

 

 
GKFG-1512-MONOMER
 

 
glycogen debranching prot
 

 
1.70975e-4
 

 
1.0
 

 
P15067
 

 
1.92813e-24
 

 
3.0
 

 
0.14611872
 

 
657
 

 
T
 

 
NIL
 

 

 

 
GKFG-1510-MONOMER
 

 
iva:Isova_2027 malto-olig
 

 
2.3426614e-5
 

 
1.0
 

 
P15067
 

 
0.983246
 

 
8.0
 

 
0.17656012
 

 
657
 

 
T
 

 
NIL
 

 

 

 
GKFG-1509-MONOMER
 

 
iva:Isova_2028 malto-olig
 

 
1.1396868e-5
 

 
1.0
 

 
P15067
 

 
2.2048e-5
 

 
7.0
 

 
0.20243531
 

 
657
 

 
T
 

 
NIL
 

 

 
     

  starch degradation II     Total # of reactions in pathway = 9   Present reactions: 1
  

 

 
 Reaction 
 

 
 Protein(s) 
 

 

 

 
RXN-12391
 

 
(# )
 

 

 
   Missing reactions: 8
  

 

 
 RXN-12203 
 

 

 

 
 RXN-12204 
 

 

 

 
 RXN-12276 
 

 

 

 
 RXN-12277 
 

 

 

 
 RXN-12278 
 

 

 

 
 RXN-12279 
 

 

 

 
 RXN-12280 
 

 

 

 
 RXN-12384 
 

 

 
        Evidence for , EC# 2.7.9.4   RXN-12203 
  

 

 
 Hit 
 

 
 Common 
 

 
 P 
 

 
 #Q 
 

 
 Best Qry 
 

 
 Best Eval 
 

 
 Avg Rank 
 

 
 Aln Len 
 

 
 Qry Len 
 

 
 IDOP? 
 

 
 adj? 
 

 

 

 
GKFG-1950-MONOMER
 

 
bcv:Bcav_2215 pyruvate ki
 

 
1.3945618e-5
 

 
1.0
 

 
Q9SAC6
 

 
0.382489
 

 
1.0
 

 
0.10150107
 

 
1399
 

 
T
 

 
NIL
 

 

 
     Evidence for , EC# 2.7.9.5   RXN-12204 
  
no BLAST hits found
 
     Evidence for , EC# 3.1.3.-   RXN-12276 
  

 

 
 Hit 
 

 
 Common 
 

 
 P 
 

 
 #Q 
 

 
 Best Qry 
 

 
 Best Eval 
 

 
 Avg Rank 
 

 
 Aln Len 
 

 
 Qry Len 
 

 
 IDOP? 
 

 
 adj? 
 

 

 

 
GKFG-724-MONOMER
 

 
serine/threonine protein 
 

 
5.771539e-5
 

 
1.0
 

 
Q9FEB5
 

 
0.470705
 

 
2.0
 

 
0.36147758
 

 
379
 

 
T
 

 
NIL
 

 

 

 
GKFG-1239-MONOMER
 

 
(db=HMMPfam db_id=PF05738
 

 
1.3945618e-5
 

 
1.0
 

 
Q9FEB5
 

 
0.265156
 

 
1.0
 

 
0.105540894
 

 
379
 

 
T
 

 
NIL
 

 

 
     Evidence for , EC# 3.1.3.-   RXN-12277 
  

 

 
 Hit 
 

 
 Common 
 

 
 P 
 

 
 #Q 
 

 
 Best Qry 
 

 
 Best Eval 
 

 
 Avg Rank 
 

 
 Aln Len 
 

 
 Qry Len 
 

 
 IDOP? 
 

 
 adj? 
 

 

 

 
GKFG-724-MONOMER
 

 
serine/threonine protein 
 

 
5.771539e-5
 

 
1.0
 

 
Q9FEB5
 

 
0.470705
 

 
2.0
 

 
0.36147758
 

 
379
 

 
T
 

 
NIL
 

 

 

 
GKFG-1239-MONOMER
 

 
(db=HMMPfam db_id=PF05738
 

 
1.3945618e-5
 

 
1.0
 

 
Q9FEB5
 

 
0.265156
 

 
1.0
 

 
0.105540894
 

 
379
 

 
T
 

 
NIL
 

 

 
     Evidence for , EC# 3.2.1.-   RXN-12278 
  
no BLAST hits found
 
     Evidence for , EC# 3.2.1.-   RXN-12279 
  
no BLAST hits found
 
     Evidence for , EC# 3.2.1.68   RXN-12280 
  

 

 
 Hit 
 

 
 Common 
 

 
 P 
 

 
 #Q 
 

 
 Best Qry 
 

 
 Best Eval 
 

 
 Avg Rank 
 

 
 Aln Len 
 

 
 Qry Len 
 

 
 IDOP? 
 

 
 adj? 
 

 

 

 
GKFG-1248-MONOMER
 

 
cfi:Celf_2759 glycogen de
 

 
0.43435922
 

 
1.0
 

 
Q9M0S5
 

 
5.45927e-100
 

 
2.0
 

 
0.8337696
 

 
764
 

 
T
 

 
NIL
 

 

 

 
GKFG-1511-MONOMER
 

 
cga:Celgi_1503 glycogen d
 

 
0.42872137
 

 
1.0
 

 
Q9M0S5
 

 
8.78766e-122
 

 
1.0
 

 
0.5929319
 

 
764
 

 
T
 

 
NIL
 

 

 

 
GKFG-1512-MONOMER
 

 
glycogen debranching prot
 

 
1.3788765e-4
 

 
1.0
 

 
Q9M0S5
 

 
1.28656e-13
 

 
3.0
 

 
0.14921466
 

 
764
 

 
T
 

 
NIL
 

 

 

 
GKFG-1290-MONOMER
 

 
vma:VAB18032_29276 alpha 
 

 
1.03159044e-4
 

 
1.0
 

 
Q9M0S5
 

 
5.12548e-6
 

 
7.0
 

 
0.30759162
 

 
764
 

 
T
 

 
NIL
 

 

 

 
GKFG-1241-MONOMER
 

 
ahe:Arch_0385 trehalose s
 

 
9.087932e-5
 

 
1.0
 

 
Q9M0S5
 

 
3.83935e-7
 

 
6.0
 

 
0.30890054
 

 
764
 

 
T
 

 
NIL
 

 

 

 
GKFG-957-MONOMER
 

 
mcu:HMPREF0573_10357 fusA
 

 
2.3426614e-5
 

 
1.0
 

 
Q9M0S5
 

 
0.0647198
 

 
8.0
 

 
0.16884817
 

 
764
 

 
T
 

 
NIL
 

 

 

 
GKFG-1234-MONOMER
 

 
mcu:HMPREF0573_11119 glgB
 

 
1.4625372e-5
 

 
1.0
 

 
Q9M0S5
 

 
4.58314e-10
 

 
4.0
 

 
0.2814136
 

 
764
 

 
T
 

 
NIL
 

 

 

 
GKFG-1509-MONOMER
 

 
iva:Isova_2028 malto-olig
 

 
4.3925725e-6
 

 
1.0
 

 
Q9M0S5
 

 
1.4403e-7
 

 
5.0
 

 
0.15837696
 

 
764
 

 
T
 

 
NIL
 

 

 
     Evidence for , EC# 3.2.1.-   RXN-12384 
  
no BLAST hits found
 
     

  starch degradation I     Total # of reactions in pathway = 9   Present reactions: 4
  

 

 
 Reaction 
 

 
 Protein(s) 
 

 

 

 
RXN-1826
 

 
(# )
 

 

 

 
RXN-1828
 

 
(# )
 

 

 

 
RXN-2141
 

 
(# )
 

 

 

 
PHOSPHOGLUCMUT-RXN
 

 
(# )
 

 

 
   Missing reactions: 5
  

 

 
 RXN-1823 
 

 

 

 
 RXN-1824 
 

 

 

 
 RXN-1825 
 

 

 

 
 RXN-1827 
 

 

 

 
 RXN-1685 
 

 

 
        Evidence for &alpha;-amylase, EC# 3.2.1.1   RXN-1823 
  

 

 
 Hit 
 

 
 Common 
 

 
 P 
 

 
 #Q 
 

 
 Best Qry 
 

 
 Best Eval 
 

 
 Avg Rank 
 

 
 Aln Len 
 

 
 Qry Len 
 

 
 IDOP? 
 

 
 adj? 
 

 

 

 
GKFG-1290-MONOMER
 

 
vma:VAB18032_29276 alpha 
 

 
0.74141914
 

 
27.0
 

 
P14899
 

 
7.03074e-48
 

 
2.4444444
 

 
0.30316174
 

 
499
 

 
T
 

 
NIL
 

 

 

 
GKFG-1241-MONOMER
 

 
ahe:Arch_0385 trehalose s
 

 
0.6580651
 

 
30.0
 

 
P14899
 

 
3.96984e-77
 

 
1.4333333
 

 
0.37225065
 

 
499
 

 
T
 

 
NIL
 

 

 

 
GKFG-1509-MONOMER
 

 
iva:Isova_2028 malto-olig
 

 
0.055705592
 

 
22.0
 

 
Q01117
 

 
2.09556e-13
 

 
3.8636363
 

 
0.24997778
 

 
624
 

 
T
 

 
NIL
 

 

 

 
GKFG-1510-MONOMER
 

 
iva:Isova_2027 malto-olig
 

 
0.0064745373
 

 
22.0
 

 
P14899
 

 
4.83965e-11
 

 
3.9545455
 

 
0.13168861
 

 
499
 

 
T
 

 
NIL
 

 

 

 
GKFG-1248-MONOMER
 

 
cfi:Celf_2759 glycogen de
 

 
3.5468966e-4
 

 
23.0
 

 
P14899
 

 
2.18598e-7
 

 
5.347826
 

 
0.1962569
 

 
499
 

 
T
 

 
NIL
 

 

 

 
GKFG-1242-MONOMER
 

 
cfi:Celf_2761 alpha amyla
 

 
2.1202605e-4
 

 
4.0
 

 
P14899
 

 
1.11288e-7
 

 
7.25
 

 
0.32150096
 

 
499
 

 
T
 

 
NIL
 

 

 

 
GKFG-1511-MONOMER
 

 
cga:Celgi_1503 glycogen d
 

 
1.552077e-4
 

 
28.0
 

 
P14899
 

 
8.4317e-9
 

 
4.821429
 

 
0.19860391
 

 
499
 

 
T
 

 
NIL
 

 

 

 
GKFG-498-MONOMER
 

 
mcu:HMPREF0573_10832 nrdD
 

 
5.75402e-5
 

 
1.0
 

 
P25718
 

 
0.173882
 

 
8.0
 

 
0.06656805
 

 
676
 

 
NIL
 

 
NIL
 

 

 

 
GKFG-1234-MONOMER
 

 
mcu:HMPREF0573_11119 glgB
 

 
5.7093494e-5
 

 
26.0
 

 
P21543
 

 
5.93599e-9
 

 
3.9615386
 

 
0.1982092
 

 
1196
 

 
T
 

 
NIL
 

 

 

 
GKFG-1306-MONOMER
 

 
putative dihydrofolate re
 

 
3.81314e-5
 

 
1.0
 

 
P25718
 

 
0.495526
 

 
9.0
 

 
0.10207101
 

 
676
 

 
T
 

 
NIL
 

 

 

 
GKFG-1172-MONOMER
 

 
LacI family transcription
 

 
2.3426614e-5
 

 
1.0
 

 
O14154
 

 
0.776064
 

 
8.0
 

 
0.13645224
 

 
513
 

 
T
 

 
NIL
 

 

 

 
GKFG-614-MONOMER
 

 
Putative glycerate kinase
 

 
2.118447e-5
 

 
1.0
 

 
P30269
 

 
0.64725
 

 
8.0
 

 
0.05532787
 

 
976
 

 
T
 

 
NIL
 

 

 

 
GKFG-973-MONOMER
 

 
pfr:PFREUD_23130 iolH; my
 

 
1.0306069e-5
 

 
1.0
 

 
Q23834
 

 
0.648995
 

 
7.0
 

 
0.11515152
 

 
495
 

 
T
 

 
NIL
 

 

 

 
GKFG-2008-MONOMER
 

 
mcu:HMPREF0573_11878 csd;
 

 
1.0306069e-5
 

 
1.0
 

 
Q9Y7S9
 

 
0.698033
 

 
7.0
 

 
0.04964539
 

 
564
 

 
T
 

 
NIL
 

 

 

 
GKFG-1134-MONOMER
 

 
seg (db=Seg db_id=seg fro
 

 
9.079168e-6
 

 
1.0
 

 
P30269
 

 
0.184033
 

 
6.0
 

 
0.08196721
 

 
976
 

 
T
 

 
NIL
 

 

 

 
GKFG-80-MONOMER
 

 
(db=HMMPfam db_id=PF00717
 

 
9.079168e-6
 

 
1.0
 

 
P29750
 

 
0.645312
 

 
6.0
 

 
0.0661157
 

 
605
 

 
T
 

 
NIL
 

 

 

 
GKFG-1963-MONOMER
 

 
HAD-superfamily hydrolase
 

 
3.972156e-6
 

 
1.0
 

 
P29957
 

 
0.729982
 

 
5.0
 

 
0.08370703
 

 
669
 

 
T
 

 
NIL
 

 

 

 
GKFG-81-MONOMER
 

 
transmembrane_regions (db
 

 
2.5282066e-6
 

 
1.0
 

 
P29957
 

 
0.0672689
 

 
2.0
 

 
0.11509716
 

 
669
 

 
T
 

 
NIL
 

 

 

 
GKFG-1893-MONOMER
 

 
seg (db=Seg db_id=seg fro
 

 
1.4610267e-6
 

 
1.0
 

 
P29957
 

 
0.523058
 

 
4.0
 

 
0.056801196
 

 
669
 

 
T
 

 
NIL
 

 

 
     Evidence for pullulanase, EC# 3.2.1.41   RXN-1824 
  

 

 
 Hit 
 

 
 Common 
 

 
 P 
 

 
 #Q 
 

 
 Best Qry 
 

 
 Best Eval 
 

 
 Avg Rank 
 

 
 Aln Len 
 

 
 Qry Len 
 

 
 IDOP? 
 

 
 adj? 
 

 

 

 
GKFG-1290-MONOMER
 

 
vma:VAB18032_29276 alpha 
 

 
0.15204865
 

 
1.0
 

 
P38536
 

 
4.05227e-18
 

 
2.0
 

 
0.22729716
 

 
1861
 

 
T
 

 
T
 

 

 

 
GKFG-1248-MONOMER
 

 
cfi:Celf_2759 glycogen de
 

 
0.07299803
 

 
4.0
 

 
O33840
 

 
5.13394e-39
 

 
2.5
 

 
0.5031942
 

 
843
 

 
T
 

 
NIL
 

 

 

 
GKFG-1511-MONOMER
 

 
cga:Celgi_1503 glycogen d
 

 
0.01360282
 

 
4.0
 

 
C0SPA0
 

 
1.64459e-31
 

 
3.0
 

 
0.41832668
 

 
718
 

 
T
 

 
NIL
 

 

 

 
GKFG-1234-MONOMER
 

 
mcu:HMPREF0573_11119 glgB
 

 
0.0016665078
 

 
4.0
 

 
C0SPA0
 

 
5.25894e-19
 

 
3.5
 

 
0.31988686
 

 
718
 

 
T
 

 
NIL
 

 

 

 
GKFG-1241-MONOMER
 

 
ahe:Arch_0385 trehalose s
 

 
1.3788765e-4
 

 
2.0
 

 
P38536
 

 
2.5062e-20
 

 
3.0
 

 
0.20107527
 

 
1861
 

 
T
 

 
NIL
 

 

 

 
GKFG-1510-MONOMER
 

 
iva:Isova_2027 malto-olig
 

 
1.2469188e-4
 

 
1.0
 

 
P38536
 

 
6.36014e-11
 

 
3.0
 

 
0.06716819
 

 
1861
 

 
T
 

 
NIL
 

 

 

 
GKFG-632-MONOMER
 

 
rdn:HMPREF0733_11588 dinG
 

 
3.972156e-6
 

 
1.0
 

 
P07811
 

 
0.166947
 

 
5.0
 

 
0.062043797
 

 
1096
 

 
T
 

 
NIL
 

 

 

 
GKFG-1509-MONOMER
 

 
iva:Isova_2028 malto-olig
 

 
1.6156636e-6
 

 
4.0
 

 
C0SPA0
 

 
4.11517e-8
 

 
4.0
 

 
0.21288724
 

 
718
 

 
T
 

 
NIL
 

 

 
     Evidence for &alpha;-amylase, EC# 3.2.1.1   RXN-1825 
  

 

 
 Hit 
 

 
 Common 
 

 
 P 
 

 
 #Q 
 

 
 Best Qry 
 

 
 Best Eval 
 

 
 Avg Rank 
 

 
 Aln Len 
 

 
 Qry Len 
 

 
 IDOP? 
 

 
 adj? 
 

 

 

 
GKFG-1290-MONOMER
 

 
vma:VAB18032_29276 alpha 
 

 
0.74141914
 

 
27.0
 

 
P14899
 

 
7.03074e-48
 

 
2.4444444
 

 
0.30316174
 

 
499
 

 
T
 

 
NIL
 

 

 

 
GKFG-1241-MONOMER
 

 
ahe:Arch_0385 trehalose s
 

 
0.6580651
 

 
30.0
 

 
P14899
 

 
3.96984e-77
 

 
1.4333333
 

 
0.37225065
 

 
499
 

 
T
 

 
NIL
 

 

 

 
GKFG-1509-MONOMER
 

 
iva:Isova_2028 malto-olig
 

 
0.055705592
 

 
22.0
 

 
Q01117
 

 
2.09556e-13
 

 
3.8636363
 

 
0.24997778
 

 
624
 

 
T
 

 
NIL
 

 

 

 
GKFG-1510-MONOMER
 

 
iva:Isova_2027 malto-olig
 

 
0.0064745373
 

 
22.0
 

 
P14899
 

 
4.83965e-11
 

 
3.9545455
 

 
0.13168861
 

 
499
 

 
T
 

 
NIL
 

 

 

 
GKFG-1248-MONOMER
 

 
cfi:Celf_2759 glycogen de
 

 
3.5468966e-4
 

 
23.0
 

 
P14899
 

 
2.18598e-7
 

 
5.347826
 

 
0.1962569
 

 
499
 

 
T
 

 
NIL
 

 

 

 
GKFG-1242-MONOMER
 

 
cfi:Celf_2761 alpha amyla
 

 
2.1202605e-4
 

 
4.0
 

 
P14899
 

 
1.11288e-7
 

 
7.25
 

 
0.32150096
 

 
499
 

 
T
 

 
NIL
 

 

 

 
GKFG-1511-MONOMER
 

 
cga:Celgi_1503 glycogen d
 

 
1.552077e-4
 

 
28.0
 

 
P14899
 

 
8.4317e-9
 

 
4.821429
 

 
0.19860391
 

 
499
 

 
T
 

 
NIL
 

 

 

 
GKFG-498-MONOMER
 

 
mcu:HMPREF0573_10832 nrdD
 

 
5.75402e-5
 

 
1.0
 

 
P25718
 

 
0.173882
 

 
8.0
 

 
0.06656805
 

 
676
 

 
NIL
 

 
NIL
 

 

 

 
GKFG-1234-MONOMER
 

 
mcu:HMPREF0573_11119 glgB
 

 
5.7093494e-5
 

 
26.0
 

 
P21543
 

 
5.93599e-9
 

 
3.9615386
 

 
0.1982092
 

 
1196
 

 
T
 

 
NIL
 

 

 

 
GKFG-1306-MONOMER
 

 
putative dihydrofolate re
 

 
3.81314e-5
 

 
1.0
 

 
P25718
 

 
0.495526
 

 
9.0
 

 
0.10207101
 

 
676
 

 
T
 

 
NIL
 

 

 

 
GKFG-1172-MONOMER
 

 
LacI family transcription
 

 
2.3426614e-5
 

 
1.0
 

 
O14154
 

 
0.776064
 

 
8.0
 

 
0.13645224
 

 
513
 

 
T
 

 
NIL
 

 

 

 
GKFG-614-MONOMER
 

 
Putative glycerate kinase
 

 
2.118447e-5
 

 
1.0
 

 
P30269
 

 
0.64725
 

 
8.0
 

 
0.05532787
 

 
976
 

 
T
 

 
NIL
 

 

 

 
GKFG-973-MONOMER
 

 
pfr:PFREUD_23130 iolH; my
 

 
1.0306069e-5
 

 
1.0
 

 
Q23834
 

 
0.648995
 

 
7.0
 

 
0.11515152
 

 
495
 

 
T
 

 
NIL
 

 

 

 
GKFG-2008-MONOMER
 

 
mcu:HMPREF0573_11878 csd;
 

 
1.0306069e-5
 

 
1.0
 

 
Q9Y7S9
 

 
0.698033
 

 
7.0
 

 
0.04964539
 

 
564
 

 
T
 

 
NIL
 

 

 

 
GKFG-1134-MONOMER
 

 
seg (db=Seg db_id=seg fro
 

 
9.079168e-6
 

 
1.0
 

 
P30269
 

 
0.184033
 

 
6.0
 

 
0.08196721
 

 
976
 

 
T
 

 
NIL
 

 

 

 
GKFG-80-MONOMER
 

 
(db=HMMPfam db_id=PF00717
 

 
9.079168e-6
 

 
1.0
 

 
P29750
 

 
0.645312
 

 
6.0
 

 
0.0661157
 

 
605
 

 
T
 

 
NIL
 

 

 

 
GKFG-1963-MONOMER
 

 
HAD-superfamily hydrolase
 

 
3.972156e-6
 

 
1.0
 

 
P29957
 

 
0.729982
 

 
5.0
 

 
0.08370703
 

 
669
 

 
T
 

 
NIL
 

 

 

 
GKFG-81-MONOMER
 

 
transmembrane_regions (db
 

 
2.5282066e-6
 

 
1.0
 

 
P29957
 

 
0.0672689
 

 
2.0
 

 
0.11509716
 

 
669
 

 
T
 

 
NIL
 

 

 

 
GKFG-1893-MONOMER
 

 
seg (db=Seg db_id=seg fro
 

 
1.4610267e-6
 

 
1.0
 

 
P29957
 

 
0.523058
 

 
4.0
 

 
0.056801196
 

 
669
 

 
T
 

 
NIL
 

 

 
     Evidence for &beta;-amylase, EC# 3.2.1.2   RXN-1827 
  

 

 
 Hit 
 

 
 Common 
 

 
 P 
 

 
 #Q 
 

 
 Best Qry 
 

 
 Best Eval 
 

 
 Avg Rank 
 

 
 Aln Len 
 

 
 Qry Len 
 

 
 IDOP? 
 

 
 adj? 
 

 

 

 
GKFG-1290-MONOMER
 

 
vma:VAB18032_29276 alpha 
 

 
0.15204865
 

 
1.0
 

 
P21543
 

 
2.53391e-11
 

 
2.0
 

 
0.21488294
 

 
1196
 

 
T
 

 
T
 

 

 

 
GKFG-1241-MONOMER
 

 
ahe:Arch_0385 trehalose s
 

 
0.0017570792
 

 
1.0
 

 
P21543
 

 
6.66203e-17
 

 
1.0
 

 
0.16220737
 

 
1196
 

 
T
 

 
NIL
 

 

 

 
GKFG-1944-MONOMER
 

 
bcv:Bcav_2184 excinucleas
 

 
1.3945618e-5
 

 
1.0
 

 
Q9FH80
 

 
0.520976
 

 
1.0
 

 
0.082728595
 

 
689
 

 
T
 

 
NIL
 

 

 

 
GKFG-1509-MONOMER
 

 
iva:Isova_2028 malto-olig
 

 
1.1396868e-5
 

 
1.0
 

 
P21543
 

 
5.23697e-6
 

 
7.0
 

 
0.13795987
 

 
1196
 

 
T
 

 
NIL
 

 

 

 
GKFG-1248-MONOMER
 

 
cfi:Celf_2759 glycogen de
 

 
1.0040111e-5
 

 
1.0
 

 
P21543
 

 
4.35757e-6
 

 
6.0
 

 
0.14464884
 

 
1196
 

 
T
 

 
NIL
 

 

 

 
GKFG-1510-MONOMER
 

 
iva:Isova_2027 malto-olig
 

 
3.972156e-6
 

 
1.0
 

 
P21543
 

 
3.04689e-6
 

 
5.0
 

 
0.06521739
 

 
1196
 

 
T
 

 
NIL
 

 

 

 
GKFG-1511-MONOMER
 

 
cga:Celgi_1503 glycogen d
 

 
1.6156636e-6
 

 
1.0
 

 
P21543
 

 
1.06325e-7
 

 
4.0
 

 
0.16555184
 

 
1196
 

 
T
 

 
NIL
 

 

 

 
GKFG-1234-MONOMER
 

 
mcu:HMPREF0573_11119 glgB
 

 
1.2082764e-6
 

 
1.0
 

 
P21543
 

 
5.93599e-9
 

 
3.0
 

 
0.12541807
 

 
1196
 

 
T
 

 
NIL
 

 

 
     Evidence for , EC# 2.7.1.1   RXN-1685 
  

 

 
 Hit 
 

 
 Common 
 

 
 P 
 

 
 #Q 
 

 
 Best Qry 
 

 
 Best Eval 
 

 
 Avg Rank 
 

 
 Aln Len 
 

 
 Qry Len 
 

 
 IDOP? 
 

 
 adj? 
 

 

 

 
GKFG-552-MONOMER
 

 
hypothetical protein (db=
 

 
3.787886e-5
 

 
1.0
 

 
Q42525
 

 
0.0802432
 

 
1.0
 

 
0.10483871
 

 
496
 

 
NIL
 

 
NIL
 

 

 
     

  sucrose biosynthesis     Total # of reactions in pathway = 9   Present reactions: 6
  

 

 
 Reaction 
 

 
 Protein(s) 
 

 

 

 
PHOSPHOGLUCMUT-RXN
 

 
(# )
 

 

 

 
GLUC1PURIDYLTRANS-RXN
 

 
(# )
 

 

 

 
PHOSGLYPHOS-RXN
 

 
(# )
 

 

 

 
GAPOXNPHOSPHN-RXN
 

 
(# )
 

 

 

 
F16ALDOLASE-RXN
 

 
(# )
 

 

 

 
PGLUCISOM-RXN
 

 
(# )
 

 

 
   Missing reactions: 3
  

 

 
 SUCROSE-PHOSPHATE-SYNTHASE-RXN 
 

 

 

 
 SUCROSE-PHOSPHATASE-RXN 
 

 

 

 
 F16BDEPHOS-RXN 
 

 

 
        Evidence for sucrose-phosphate synthase, EC# 2.4.1.14   SUCROSE-PHOSPHATE-SYNTHASE-RXN 
  

 

 
 Hit 
 

 
 Common 
 

 
 P 
 

 
 #Q 
 

 
 Best Qry 
 

 
 Best Eval 
 

 
 Avg Rank 
 

 
 Aln Len 
 

 
 Qry Len 
 

 
 IDOP? 
 

 
 adj? 
 

 

 

 
GKFG-1991-MONOMER
 

 
ahe:Arch_0927 glycogen sy
 

 
1.5421621e-5
 

 
2.0
 

 
Q43876
 

 
6.74198e-6
 

 
1.0
 

 
0.21832892
 

 
1059
 

 
T
 

 
NIL
 

 

 

 
GKFG-1814-MONOMER
 

 
cfl:Cfla_1787 group 1 gly
 

 
2.795795e-6
 

 
2.0
 

 
Q43876
 

 
0.0175366
 

 
2.0
 

 
0.20253651
 

 
1059
 

 
T
 

 
NIL
 

 

 
     Evidence for sucrose-phosphate phosphatase, EC# 3.1.3.24   SUCROSE-PHOSPHATASE-RXN 
  

 

 
 Hit 
 

 
 Common 
 

 
 P 
 

 
 #Q 
 

 
 Best Qry 
 

 
 Best Eval 
 

 
 Avg Rank 
 

 
 Aln Len 
 

 
 Qry Len 
 

 
 IDOP? 
 

 
 adj? 
 

 

 

 
GKFG-521-MONOMER
 

 
UniRef90_E6KT22 Stage 0 D
 

 
4.188785e-5
 

 
1.0
 

 
Q93WU4
 

 
0.063333
 

 
1.0
 

 
0.14823529
 

 
425
 

 
NIL
 

 
NIL
 

 

 

 
GKFG-1048-MONOMER
 

 
bfa:Bfae_17560 methionyl-
 

 
2.5307965e-5
 

 
1.0
 

 
Q93WU4
 

 
0.885997
 

 
2.0
 

 
0.33882353
 

 
425
 

 
T
 

 
NIL
 

 

 

 
GKFG-806-MONOMER
 

 
beta-lactamase (db=KEGG e
 

 
1.3945618e-5
 

 
1.0
 

 
Q84ZX8
 

 
0.785025
 

 
1.0
 

 
0.07322654
 

 
437
 

 
T
 

 
NIL
 

 

 
     Evidence for fructose-bisphosphatase, EC# 3.1.3.11   F16BDEPHOS-RXN 
  

 

 
 Hit 
 

 
 Common 
 

 
 P 
 

 
 #Q 
 

 
 Best Qry 
 

 
 Best Eval 
 

 
 Avg Rank 
 

 
 Aln Len 
 

 
 Qry Len 
 

 
 IDOP? 
 

 
 adj? 
 

 

 

 
GKFG-1229-MONOMER
 

 
hypothetical protein; K09
 

 
1.3958463e-4
 

 
1.0
 

 
C6BTU7
 

 
0.458626
 

 
1.0
 

 
0.295858
 

 
338
 

 
T
 

 
NIL
 

 

 

 
GKFG-1027-MONOMER
 

 
cfi:Celf_2525 mannose-6-p
 

 
1.3958463e-4
 

 
1.0
 

 
A1KHN9
 

 
0.810805
 

 
1.0
 

 
0.32320443
 

 
362
 

 
T
 

 
NIL
 

 

 

 
GKFG-933-MONOMER
 

 
HAD-superfamily hydrolase
 

 
3.3353957e-5
 

 
1.0
 

 
P21829
 

 
0.00268893
 

 
4.0
 

 
0.38235295
 

 
272
 

 
T
 

 
NIL
 

 

 

 
GKFG-1543-MONOMER
 

 
UBA/ThiF-type NAD/FAD bin
 

 
2.5307965e-5
 

 
1.0
 

 
P46726
 

 
0.899178
 

 
2.0
 

 
0.34317344
 

 
271
 

 
T
 

 
NIL
 

 

 

 
GKFG-681-MONOMER
 

 
archaeal fructose-1,6-bis
 

 
1.5421621e-5
 

 
3.0
 

 
P46726
 

 
0.0526868
 

 
1.0
 

 
0.23745935
 

 
271
 

 
T
 

 
NIL
 

 

 

 
GKFG-1563-MONOMER
 

 
hypothetical protein (db=
 

 
1.5421621e-5
 

 
1.0
 

 
O25936
 

 
0.252277
 

 
1.0
 

 
0.15172414
 

 
290
 

 
T
 

 
NIL
 

 

 

 
GKFG-304-MONOMER
 

 
hypothetical protein (db=
 

 
1.5421621e-5
 

 
1.0
 

 
Q5V3Z1
 

 
0.494724
 

 
1.0
 

 
0.23367697
 

 
291
 

 
T
 

 
NIL
 

 

 

 
GKFG-86-MONOMER
 

 
Putative uncharacterized 
 

 
1.5421621e-5
 

 
1.0
 

 
B0CAD9
 

 
0.0244708
 

 
1.0
 

 
0.19327731
 

 
357
 

 
T
 

 
NIL
 

 

 

 
GKFG-1275-MONOMER
 

 
metal ion ABC transporter
 

 
1.5421621e-5
 

 
1.0
 

 
Q2FM20
 

 
0.272969
 

 
1.0
 

 
0.15755627
 

 
311
 

 
T
 

 
NIL
 

 

 

 
GKFG-153-MONOMER
 

 
mcu:HMPREF0573_10425 ppdK
 

 
1.5421621e-5
 

 
1.0
 

 
A3D0P6
 

 
0.413008
 

 
1.0
 

 
0.20175439
 

 
342
 

 
T
 

 
NIL
 

 

 

 
GKFG-1632-MONOMER
 

 
ahe:Arch_1316 acetate kin
 

 
1.5421621e-5
 

 
1.0
 

 
B5EFV5
 

 
0.370326
 

 
1.0
 

 
0.17791411
 

 
326
 

 
T
 

 
NIL
 

 

 

 
GKFG-739-MONOMER
 

 
bcv:Bcav_1600 DEAD/DEAH b
 

 
1.5421621e-5
 

 
1.0
 

 
B2V6E2
 

 
0.348491
 

 
1.0
 

 
0.21671827
 

 
323
 

 
T
 

 
NIL
 

 

 

 
GKFG-935-MONOMER
 

 
ahe:Arch_0038 alcohol deh
 

 
1.5421621e-5
 

 
1.0
 

 
A5E987
 

 
0.14728
 

 
1.0
 

 
0.15680474
 

 
338
 

 
T
 

 
NIL
 

 

 

 
GKFG-410-MONOMER
 

 
hydrolase of the HAD supe
 

 
1.5421621e-5
 

 
1.0
 

 
P21829
 

 
7.54305e-6
 

 
1.0
 

 
0.23529412
 

 
272
 

 
T
 

 
NIL
 

 

 

 
GKFG-701-MONOMER
 

 
ach:Achl_1330 fibronectin
 

 
1.5421621e-5
 

 
1.0
 

 
P19112
 

 
0.0755288
 

 
1.0
 

 
0.21763085
 

 
363
 

 
T
 

 
NIL
 

 

 

 
GKFG-598-MONOMER
 

 
krh:KRH_14360 sigA; RNA p
 

 
1.3945618e-5
 

 
1.0
 

 
B0C7G7
 

 
0.366936
 

 
1.0
 

 
0.11782477
 

 
331
 

 
T
 

 
NIL
 

 

 

 
GKFG-66-MONOMER
 

 
kfl:Kfla_6989 hypothetica
 

 
1.3945618e-5
 

 
1.0
 

 
A9FZA0
 

 
0.460166
 

 
1.0
 

 
0.0873635
 

 
641
 

 
T
 

 
NIL
 

 

 

 
GKFG-1270-MONOMER
 

 
jde:Jden_1096 bifunctiona
 

 
1.3945618e-5
 

 
1.0
 

 
A7IL64
 

 
0.882531
 

 
1.0
 

 
0.10030395
 

 
329
 

 
T
 

 
NIL
 

 

 

 
GKFG-1882-MONOMER
 

 
pcn:TIB1ST10_03215 oxidor
 

 
1.3945618e-5
 

 
1.0
 

 
A7IGL7
 

 
0.937412
 

 
1.0
 

 
0.08625337
 

 
371
 

 
T
 

 
NIL
 

 

 

 
GKFG-176-MONOMER
 

 
bcv:Bcav_3230 geranylgera
 

 
1.3945618e-5
 

 
1.0
 

 
Q8D275
 

 
0.908721
 

 
1.0
 

 
0.10060976
 

 
328
 

 
T
 

 
NIL
 

 

 

 
GKFG-1699-MONOMER
 

 
mcu:HMPREF0573_11727 alaS
 

 
1.3945618e-5
 

 
1.0
 

 
A6X307
 

 
0.0588743
 

 
1.0
 

 
0.09117647
 

 
340
 

 
T
 

 
NIL
 

 

 

 
GKFG-1231-MONOMER
 

 
fma:FMG_0239 antibiotic A
 

 
1.3945618e-5
 

 
1.0
 

 
B1XVF7
 

 
0.0506714
 

 
1.0
 

 
0.06784661
 

 
339
 

 
T
 

 
NIL
 

 

 

 
GKFG-281-MONOMER
 

 
bfa:Bfae_19800 copper/sil
 

 
4.3925725e-6
 

 
1.0
 

 
P21829
 

 
0.55393
 

 
5.0
 

 
0.14338236
 

 
272
 

 
T
 

 
NIL
 

 

 

 
GKFG-1239-MONOMER
 

 
(db=HMMPfam db_id=PF05738
 

 
2.795795e-6
 

 
2.0
 

 
A6X307
 

 
0.50127
 

 
1.5
 

 
0.22415079
 

 
340
 

 
T
 

 
NIL
 

 

 

 
GKFG-101-MONOMER
 

 
hydrolase of the HAD supe
 

 
2.795795e-6
 

 
1.0
 

 
P21829
 

 
7.54305e-6
 

 
2.0
 

 
0.23529412
 

 
272
 

 
T
 

 
NIL
 

 

 

 
GKFG-75-MONOMER
 

 
Bacterial adhesins (db=su
 

 
2.5282066e-6
 

 
1.0
 

 
B5EFV5
 

 
0.521669
 

 
2.0
 

 
0.1196319
 

 
326
 

 
T
 

 
NIL
 

 

 

 
GKFG-1943-MONOMER
 

 
bcv:Bcav_1945 P-type HAD 
 

 
1.2082764e-6
 

 
1.0
 

 
P21829
 

 
0.00187333
 

 
3.0
 

 
0.1985294
 

 
272
 

 
T
 

 
NIL
 

 

 
     

  gluconeogenesis I     Total # of reactions in pathway = 13   Present reactions: 8
  

 

 
 Reaction 
 

 
 Protein(s) 
 

 

 

 
PGLUCISOM-RXN
 

 
(# )
 

 

 

 
F16ALDOLASE-RXN
 

 
(# )
 

 

 

 
GAPOXNPHOSPHN-RXN
 

 
(# )
 

 

 

 
PHOSGLYPHOS-RXN
 

 
(# )
 

 

 

 
3PGAREARR-RXN
 

 
(# 
 # 
 # 
 # )
 

 

 

 
2PGADEHYDRAT-RXN
 

 
(# )
 

 

 

 
PEPSYNTH-RXN
 

 
(# )
 

 

 

 
MALATE-DEH-RXN
 

 
(# )
 

 

 
   Missing reactions: 5
  

 

 
 F16BDEPHOS-RXN 
 

 

 

 
 PEPCARBOXYKIN-RXN 
 

 

 

 
 MALIC-NAD-RXN 
 

 

 

 
 MALIC-NADP-RXN 
 

 

 

 
 PEPCARBOX-RXN 
 

 

 
        Evidence for fructose-bisphosphatase, EC# 3.1.3.11   F16BDEPHOS-RXN 
  

 

 
 Hit 
 

 
 Common 
 

 
 P 
 

 
 #Q 
 

 
 Best Qry 
 

 
 Best Eval 
 

 
 Avg Rank 
 

 
 Aln Len 
 

 
 Qry Len 
 

 
 IDOP? 
 

 
 adj? 
 

 

 

 
GKFG-153-MONOMER
 

 
mcu:HMPREF0573_10425 ppdK
 

 
1.5421621e-5
 

 
1.0
 

 
A3D0P6
 

 
0.413008
 

 
1.0
 

 
0.20175439
 

 
342
 

 
T
 

 
NIL
 

 

 

 
GKFG-1229-MONOMER
 

 
hypothetical protein; K09
 

 
1.3958463e-4
 

 
1.0
 

 
C6BTU7
 

 
0.458626
 

 
1.0
 

 
0.295858
 

 
338
 

 
T
 

 
NIL
 

 

 

 
GKFG-1027-MONOMER
 

 
cfi:Celf_2525 mannose-6-p
 

 
1.3958463e-4
 

 
1.0
 

 
A1KHN9
 

 
0.810805
 

 
1.0
 

 
0.32320443
 

 
362
 

 
T
 

 
NIL
 

 

 

 
GKFG-933-MONOMER
 

 
HAD-superfamily hydrolase
 

 
3.3353957e-5
 

 
1.0
 

 
P21829
 

 
0.00268893
 

 
4.0
 

 
0.38235295
 

 
272
 

 
T
 

 
NIL
 

 

 

 
GKFG-1543-MONOMER
 

 
UBA/ThiF-type NAD/FAD bin
 

 
2.5307965e-5
 

 
1.0
 

 
P46726
 

 
0.899178
 

 
2.0
 

 
0.34317344
 

 
271
 

 
T
 

 
NIL
 

 

 

 
GKFG-681-MONOMER
 

 
archaeal fructose-1,6-bis
 

 
1.5421621e-5
 

 
3.0
 

 
P46726
 

 
0.0526868
 

 
1.0
 

 
0.23745935
 

 
271
 

 
T
 

 
NIL
 

 

 

 
GKFG-1563-MONOMER
 

 
hypothetical protein (db=
 

 
1.5421621e-5
 

 
1.0
 

 
O25936
 

 
0.252277
 

 
1.0
 

 
0.15172414
 

 
290
 

 
T
 

 
NIL
 

 

 

 
GKFG-304-MONOMER
 

 
hypothetical protein (db=
 

 
1.5421621e-5
 

 
1.0
 

 
Q5V3Z1
 

 
0.494724
 

 
1.0
 

 
0.23367697
 

 
291
 

 
T
 

 
NIL
 

 

 

 
GKFG-86-MONOMER
 

 
Putative uncharacterized 
 

 
1.5421621e-5
 

 
1.0
 

 
B0CAD9
 

 
0.0244708
 

 
1.0
 

 
0.19327731
 

 
357
 

 
T
 

 
NIL
 

 

 

 
GKFG-1275-MONOMER
 

 
metal ion ABC transporter
 

 
1.5421621e-5
 

 
1.0
 

 
Q2FM20
 

 
0.272969
 

 
1.0
 

 
0.15755627
 

 
311
 

 
T
 

 
NIL
 

 

 

 
GKFG-1632-MONOMER
 

 
ahe:Arch_1316 acetate kin
 

 
1.5421621e-5
 

 
1.0
 

 
B5EFV5
 

 
0.370326
 

 
1.0
 

 
0.17791411
 

 
326
 

 
T
 

 
NIL
 

 

 

 
GKFG-739-MONOMER
 

 
bcv:Bcav_1600 DEAD/DEAH b
 

 
1.5421621e-5
 

 
1.0
 

 
B2V6E2
 

 
0.348491
 

 
1.0
 

 
0.21671827
 

 
323
 

 
T
 

 
NIL
 

 

 

 
GKFG-935-MONOMER
 

 
ahe:Arch_0038 alcohol deh
 

 
1.5421621e-5
 

 
1.0
 

 
A5E987
 

 
0.14728
 

 
1.0
 

 
0.15680474
 

 
338
 

 
T
 

 
NIL
 

 

 

 
GKFG-410-MONOMER
 

 
hydrolase of the HAD supe
 

 
1.5421621e-5
 

 
1.0
 

 
P21829
 

 
7.54305e-6
 

 
1.0
 

 
0.23529412
 

 
272
 

 
T
 

 
NIL
 

 

 

 
GKFG-701-MONOMER
 

 
ach:Achl_1330 fibronectin
 

 
1.5421621e-5
 

 
1.0
 

 
P19112
 

 
0.0755288
 

 
1.0
 

 
0.21763085
 

 
363
 

 
T
 

 
NIL
 

 

 

 
GKFG-598-MONOMER
 

 
krh:KRH_14360 sigA; RNA p
 

 
1.3945618e-5
 

 
1.0
 

 
B0C7G7
 

 
0.366936
 

 
1.0
 

 
0.11782477
 

 
331
 

 
T
 

 
NIL
 

 

 

 
GKFG-66-MONOMER
 

 
kfl:Kfla_6989 hypothetica
 

 
1.3945618e-5
 

 
1.0
 

 
A9FZA0
 

 
0.460166
 

 
1.0
 

 
0.0873635
 

 
641
 

 
T
 

 
NIL
 

 

 

 
GKFG-1270-MONOMER
 

 
jde:Jden_1096 bifunctiona
 

 
1.3945618e-5
 

 
1.0
 

 
A7IL64
 

 
0.882531
 

 
1.0
 

 
0.10030395
 

 
329
 

 
T
 

 
NIL
 

 

 

 
GKFG-1882-MONOMER
 

 
pcn:TIB1ST10_03215 oxidor
 

 
1.3945618e-5
 

 
1.0
 

 
A7IGL7
 

 
0.937412
 

 
1.0
 

 
0.08625337
 

 
371
 

 
T
 

 
NIL
 

 

 

 
GKFG-176-MONOMER
 

 
bcv:Bcav_3230 geranylgera
 

 
1.3945618e-5
 

 
1.0
 

 
Q8D275
 

 
0.908721
 

 
1.0
 

 
0.10060976
 

 
328
 

 
T
 

 
NIL
 

 

 

 
GKFG-1699-MONOMER
 

 
mcu:HMPREF0573_11727 alaS
 

 
1.3945618e-5
 

 
1.0
 

 
A6X307
 

 
0.0588743
 

 
1.0
 

 
0.09117647
 

 
340
 

 
T
 

 
NIL
 

 

 

 
GKFG-1231-MONOMER
 

 
fma:FMG_0239 antibiotic A
 

 
1.3945618e-5
 

 
1.0
 

 
B1XVF7
 

 
0.0506714
 

 
1.0
 

 
0.06784661
 

 
339
 

 
T
 

 
NIL
 

 

 

 
GKFG-281-MONOMER
 

 
bfa:Bfae_19800 copper/sil
 

 
4.3925725e-6
 

 
1.0
 

 
P21829
 

 
0.55393
 

 
5.0
 

 
0.14338236
 

 
272
 

 
T
 

 
NIL
 

 

 

 
GKFG-1239-MONOMER
 

 
(db=HMMPfam db_id=PF05738
 

 
2.795795e-6
 

 
2.0
 

 
A6X307
 

 
0.50127
 

 
1.5
 

 
0.22415079
 

 
340
 

 
T
 

 
NIL
 

 

 

 
GKFG-101-MONOMER
 

 
hydrolase of the HAD supe
 

 
2.795795e-6
 

 
1.0
 

 
P21829
 

 
7.54305e-6
 

 
2.0
 

 
0.23529412
 

 
272
 

 
T
 

 
NIL
 

 

 

 
GKFG-75-MONOMER
 

 
Bacterial adhesins (db=su
 

 
2.5282066e-6
 

 
1.0
 

 
B5EFV5
 

 
0.521669
 

 
2.0
 

 
0.1196319
 

 
326
 

 
T
 

 
NIL
 

 

 

 
GKFG-1943-MONOMER
 

 
bcv:Bcav_1945 P-type HAD 
 

 
1.2082764e-6
 

 
1.0
 

 
P21829
 

 
0.00187333
 

 
3.0
 

 
0.1985294
 

 
272
 

 
T
 

 
NIL
 

 

 
     Evidence for phospho enol pyruvate carboxykinase (ATP), EC# 4.1.1.49   PEPCARBOXYKIN-RXN 
  

 

 
 Hit 
 

 
 Common 
 

 
 P 
 

 
 #Q 
 

 
 Best Qry 
 

 
 Best Eval 
 

 
 Avg Rank 
 

 
 Aln Len 
 

 
 Qry Len 
 

 
 IDOP? 
 

 
 adj? 
 

 

 

 
GKFG-817-MONOMER
 

 
mph:MLP_05080 pgi; glucos
 

 
2.1178706e-4
 

 
1.0
 

 
B0SFJ3
 

 
0.889956
 

 
1.0
 

 
0.17169811
 

 
530
 

 
T
 

 
NIL
 

 

 

 
GKFG-225-MONOMER
 

 
mcu:HMPREF0573_10801 pckG
 

 
1.3958463e-4
 

 
1.0
 

 
B9DN20
 

 
0.0071146
 

 
1.0
 

 
0.2773585
 

 
530
 

 
T
 

 
NIL
 

 

 

 
GKFG-1065-MONOMER
 

 
mcu:HMPREF0573_10379 thrC
 

 
1.3958463e-4
 

 
1.0
 

 
A5FK68
 

 
0.106071
 

 
1.0
 

 
0.28651685
 

 
534
 

 
T
 

 
NIL
 

 

 

 
GKFG-402-MONOMER
 

 
DNA repair protein RadA (
 

 
1.5421621e-5
 

 
2.0
 

 
P13735
 

 
0.195942
 

 
1.0
 

 
0.19576749
 

 
472
 

 
T
 

 
NIL
 

 

 

 
GKFG-247-MONOMER
 

 
bcv:Bcav_1855 glutamine s
 

 
1.5421621e-5
 

 
1.0
 

 
A9W382
 

 
0.134399
 

 
1.0
 

 
0.19666049
 

 
539
 

 
T
 

 
NIL
 

 

 

 
GKFG-1012-MONOMER
 

 
krh:KRH_10210 ilvB; aceto
 

 
1.5421621e-5
 

 
1.0
 

 
P22259
 

 
0.137015
 

 
1.0
 

 
0.20925926
 

 
540
 

 
T
 

 
NIL
 

 

 

 
GKFG-772-MONOMER
 

 
cell surface protein (db=
 

 
1.3945618e-5
 

 
1.0
 

 
Q2S1I3
 

 
0.693648
 

 
1.0
 

 
0.09906542
 

 
535
 

 
T
 

 
NIL
 

 

 

 
GKFG-1186-MONOMER
 

 
ele:Elen_1491 ABC transpo
 

 
1.3945618e-5
 

 
1.0
 

 
A5GBF9
 

 
0.414766
 

 
1.0
 

 
0.11132076
 

 
530
 

 
T
 

 
NIL
 

 

 

 
GKFG-140-MONOMER
 

 
hypothetical protein; K07
 

 
1.3945618e-5
 

 
1.0
 

 
Q9T074
 

 
0.233533
 

 
1.0
 

 
0.05514158
 

 
671
 

 
T
 

 
NIL
 

 

 

 
GKFG-13-MONOMER
 

 
bcv:Bcav_3279 DNA repair 
 

 
2.795795e-6
 

 
2.0
 

 
P13735
 

 
0.195942
 

 
2.0
 

 
0.19576749
 

 
472
 

 
T
 

 
NIL
 

 

 

 
GKFG-684-MONOMER
 

 
bla:BLA_0177 ftsY; signal
 

 
1.2082764e-6
 

 
1.0
 

 
P13735
 

 
0.261814
 

 
3.0
 

 
0.19067797
 

 
472
 

 
T
 

 
NIL
 

 

 
     Evidence for malate dehydrogenase (oxaloacetate-decarboxylating), EC# 1.1.1.38   MALIC-NAD-RXN 
  

 

 
 Hit 
 

 
 Common 
 

 
 P 
 

 
 #Q 
 

 
 Best Qry 
 

 
 Best Eval 
 

 
 Avg Rank 
 

 
 Aln Len 
 

 
 Qry Len 
 

 
 IDOP? 
 

 
 adj? 
 

 

 

 
GKFG-402-MONOMER
 

 
DNA repair protein RadA (
 

 
1.5421621e-5
 

 
1.0
 

 
P26616
 

 
0.587798
 

 
1.0
 

 
0.21769911
 

 
565
 

 
T
 

 
NIL
 

 

 

 
GKFG-1543-MONOMER
 

 
UBA/ThiF-type NAD/FAD bin
 

 
1.5421621e-5
 

 
1.0
 

 
P54572
 

 
0.142789
 

 
1.0
 

 
0.12528473
 

 
439
 

 
T
 

 
NIL
 

 

 

 
GKFG-350-MONOMER
 

 
mcu:HMPREF0573_10218 suga
 

 
1.5421621e-5
 

 
1.0
 

 
P45868
 

 
0.079458
 

 
1.0
 

 
0.14776632
 

 
582
 

 
T
 

 
NIL
 

 

 

 
GKFG-142-MONOMER
 

 
dsy:DSY3812 hypothetical 
 

 
1.3945618e-5
 

 
1.0
 

 
P40375
 

 
0.19882
 

 
1.0
 

 
0.08672567
 

 
565
 

 
T
 

 
NIL
 

 

 

 
GKFG-13-MONOMER
 

 
bcv:Bcav_3279 DNA repair 
 

 
2.795795e-6
 

 
1.0
 

 
P26616
 

 
0.587798
 

 
2.0
 

 
0.21769911
 

 
565
 

 
T
 

 
NIL
 

 

 
     Evidence for malate dehydrogenase (oxaloacetate-decarboxylating) (NADP  +  ), EC# 1.1.1.40   MALIC-NADP-RXN 
  

 

 
 Hit 
 

 
 Common 
 

 
 P 
 

 
 #Q 
 

 
 Best Qry 
 

 
 Best Eval 
 

 
 Avg Rank 
 

 
 Aln Len 
 

 
 Qry Len 
 

 
 IDOP? 
 

 
 adj? 
 

 

 

 
GKFG-1633-MONOMER
 

 
mcu:HMPREF0573_10221 pta;
 

 
0.14671241
 

 
4.0
 

 
Q9ZFV8
 

 
7.31785e-46
 

 
1.0
 

 
0.4300506
 

 
759
 

 
T
 

 
NIL
 

 

 
     Evidence for phospho enol pyruvate carboxylase, EC# 4.1.1.31   PEPCARBOX-RXN 
  

 

 
 Hit 
 

 
 Common 
 

 
 P 
 

 
 #Q 
 

 
 Best Qry 
 

 
 Best Eval 
 

 
 Avg Rank 
 

 
 Aln Len 
 

 
 Qry Len 
 

 
 IDOP? 
 

 
 adj? 
 

 

 

 
GKFG-1046-MONOMER
 

 
xce:Xcel_0723 ATP-depende
 

 
1.3945618e-5
 

 
1.0
 

 
O32483
 

 
0.139123
 

 
1.0
 

 
0.053418804
 

 
936
 

 
T
 

 
NIL
 

 

 

 
GKFG-289-MONOMER
 

 
mcu:HMPREF0573_10751 puta
 

 
1.5421621e-5
 

 
1.0
 

 
A8AW99
 

 
0.703679
 

 
1.0
 

 
0.21518987
 

 
948
 

 
T
 

 
NIL
 

 

 

 
GKFG-1465-MONOMER
 

 
iva:Isova_1268 UvrD/REP h
 

 
1.5421621e-5
 

 
1.0
 

 
A4VN44
 

 
0.215022
 

 
1.0
 

 
0.1592719
 

 
879
 

 
T
 

 
NIL
 

 

 

 
GKFG-1767-MONOMER
 

 
hypothetical protein (db=
 

 
1.5421621e-5
 

 
1.0
 

 
Q0STS8
 

 
0.046729
 

 
1.0
 

 
0.1359404
 

 
537
 

 
T
 

 
NIL
 

 

 

 
GKFG-594-MONOMER
 

 
hypothetical protein (db=
 

 
1.5421621e-5
 

 
1.0
 

 
B0R7F9
 

 
0.884405
 

 
1.0
 

 
0.14634146
 

 
492
 

 
T
 

 
NIL
 

 

 

 
GKFG-765-MONOMER
 

 
cfl:Cfla_2325 Preprotein 
 

 
1.5421621e-5
 

 
1.0
 

 
Q8TZL5
 

 
0.14614
 

 
1.0
 

 
0.17372881
 

 
472
 

 
T
 

 
NIL
 

 

 

 
GKFG-142-MONOMER
 

 
dsy:DSY3812 hypothetical 
 

 
1.3945618e-5
 

 
1.0
 

 
Q8PS70
 

 
0.667179
 

 
1.0
 

 
0.10076046
 

 
526
 

 
T
 

 
NIL
 

 

 

 
GKFG-1621-MONOMER
 

 
tcu:Tcur_3714 3-phosphosh
 

 
1.3945618e-5
 

 
1.0
 

 
O28786
 

 
0.102436
 

 
1.0
 

 
0.10403397
 

 
471
 

 
T
 

 
NIL
 

 

 

 
GKFG-739-MONOMER
 

 
bcv:Bcav_1600 DEAD/DEAH b
 

 
1.3945618e-5
 

 
1.0
 

 
A1R2V3
 

 
0.792382
 

 
1.0
 

 
0.04828326
 

 
932
 

 
T
 

 
NIL
 

 

 

 
GKFG-1304-MONOMER
 

 
UniRef90_UPI000051013A hy
 

 
1.3945618e-5
 

 
1.0
 

 
Q5SKL7
 

 
0.671567
 

 
1.0
 

 
0.09440559
 

 
858
 

 
T
 

 
NIL
 

 

 

 
GKFG-732-MONOMER
 

 
ahe:Arch_1078 LuxR family
 

 
1.3945618e-5
 

 
1.0
 

 
Q2SL23
 

 
0.350988
 

 
1.0
 

 
0.08399546
 

 
881
 

 
T
 

 
NIL
 

 

 

 
GKFG-1347-MONOMER
 

 
aur:HMPREF9243_0883 hypot
 

 
1.3945618e-5
 

 
1.0
 

 
A4JGV5
 

 
0.0294267
 

 
1.0
 

 
0.055165496
 

 
997
 

 
T
 

 
NIL
 

 

 

 
GKFG-1068-MONOMER
 

 
ckp:ckrop_1874 hypothetic
 

 
1.3945618e-5
 

 
1.0
 

 
Q93MH3
 

 
0.252621
 

 
1.0
 

 
0.054406963
 

 
919
 

 
T
 

 
NIL
 

 

 

 
GKFG-86-MONOMER
 

 
Putative uncharacterized 
 

 
2.5282066e-6
 

 
2.0
 

 
O32483
 

 
0.238824
 

 
1.5
 

 
0.09476648
 

 
936
 

 
T
 

 
NIL
 

 

 

 
GKFG-1383-MONOMER
 

 
jde:Jden_1695 DEAD/DEAH b
 

 
2.5282066e-6
 

 
2.0
 

 
A4JGV5
 

 
0.0496707
 

 
1.5
 

 
0.070196435
 

 
997
 

 
T
 

 
NIL
 

 

 

 
GKFG-844-MONOMER
 

 
ahe:Arch_0366 ATP synthas
 

 
2.5282066e-6
 

 
1.0
 

 
A8AW99
 

 
0.828791
 

 
2.0
 

 
0.07489452
 

 
948
 

 
T
 

 
NIL
 

 

 

 
GKFG-177-MONOMER
 

 
ubiE; methylase; K03183 u
 

 
1.0926308e-6
 

 
1.0
 

 
O32483
 

 
0.310147
 

 
3.0
 

 
0.086538464
 

 
936
 

 
T
 

 
NIL
 

 

 
     

  galactose degradation III     Total # of reactions in pathway = 6   Present reactions: 3
  

 

 
 Reaction 
 

 
 Protein(s) 
 

 

 

 
GALACTOKIN-RXN
 

 
(# )
 

 

 

 
UDPGLUCEPIM-RXN
 

 
(# )
 

 

 

 
GLUC1PURIDYLTRANS-RXN
 

 
(# )
 

 

 
   Missing reactions: 3
  

 

 
 UTPHEXPURIDYLYLTRANS-RXN 
 

 

 

 
 SUCROSE-SYNTHASE-RXN 
 

 

 

 
 UGD-RXN 
 

 

 
        Evidence for UTP&mdash;hexose-1-phosphate uridylyltransferase, EC# 2.7.7.10   UTPHEXPURIDYLYLTRANS-RXN 
  

 

 
 Hit 
 

 
 Common 
 

 
 P 
 

 
 #Q 
 

 
 Best Qry 
 

 
 Best Eval 
 

 
 Avg Rank 
 

 
 Aln Len 
 

 
 Qry Len 
 

 
 IDOP? 
 

 
 adj? 
 

 

 

 
GKFG-833-MONOMER
 

 
mlu:Mlut_15870 hypothetic
 

 
0.0051016076
 

 
1.0
 

 
O33836
 

 
3.75269e-5
 

 
1.0
 

 
0.6383648
 

 
318
 

 
T
 

 
NIL
 

 

 

 
GKFG-1796-MONOMER
 

 
pac:PPA0489 UTP-glucose-1
 

 
0.0019139076
 

 
1.0
 

 
Q0GZS3
 

 
2.89989e-7
 

 
1.0
 

 
0.24918567
 

 
614
 

 
T
 

 
NIL
 

 

 

 
GKFG-1818-MONOMER
 

 
histidine triad (HIT) pro
 

 
5.771539e-5
 

 
1.0
 

 
O33836
 

 
5.02155e-4
 

 
2.0
 

 
0.3899371
 

 
318
 

 
T
 

 
NIL
 

 

 
     Evidence for Sucrose synthase, EC# 2.4.1.13   SUCROSE-SYNTHASE-RXN 
  

 

 
 Hit 
 

 
 Common 
 

 
 P 
 

 
 #Q 
 

 
 Best Qry 
 

 
 Best Eval 
 

 
 Avg Rank 
 

 
 Aln Len 
 

 
 Qry Len 
 

 
 IDOP? 
 

 
 adj? 
 

 

 

 
GKFG-1991-MONOMER
 

 
ahe:Arch_0927 glycogen sy
 

 
0.0017570792
 

 
1.0
 

 
P49040
 

 
5.00427e-11
 

 
1.0
 

 
0.14232673
 

 
808
 

 
T
 

 
NIL
 

 

 
     Evidence for UDP-glucose 6-dehydrogenase, EC# 1.1.1.22   UGD-RXN 
  

 

 
 Hit 
 

 
 Common 
 

 
 P 
 

 
 #Q 
 

 
 Best Qry 
 

 
 Best Eval 
 

 
 Avg Rank 
 

 
 Aln Len 
 

 
 Qry Len 
 

 
 IDOP? 
 

 
 adj? 
 

 

 

 
GKFG-935-MONOMER
 

 
ahe:Arch_0038 alcohol deh
 

 
0.95801926
 

 
1.0
 

 
Q6IVK8
 

 
3.53234e-32
 

 
1.0
 

 
0.99473685
 

 
380
 

 
T
 

 
NIL
 

 

 

 
GKFG-1882-MONOMER
 

 
pcn:TIB1ST10_03215 oxidor
 

 
0.5143494
 

 
1.0
 

 
Q6IVK8
 

 
6.53754e-21
 

 
2.0
 

 
0.95
 

 
380
 

 
T
 

 
NIL
 

 

 

 
GKFG-1960-MONOMER
 

 
mcu:HMPREF0573_10477 adhC
 

 
0.3304678
 

 
1.0
 

 
Q6IVK8
 

 
2.59804e-16
 

 
4.0
 

 
0.92105263
 

 
380
 

 
T
 

 
NIL
 

 

 

 
GKFG-860-MONOMER
 

 
msm:MSMEG_3094 oxidoreduc
 

 
0.31399453
 

 
1.0
 

 
Q6IVK8
 

 
7.17819e-21
 

 
3.0
 

 
0.9368421
 

 
380
 

 
T
 

 
NIL
 

 

 

 
GKFG-1236-MONOMER
 

 
mlu:Mlut_00880 glutamate 
 

 
9.087932e-5
 

 
1.0
 

 
Q6IVK8
 

 
0.748367
 

 
6.0
 

 
0.2973684
 

 
380
 

 
T
 

 
NIL
 

 

 

 
GKFG-631-MONOMER
 

 
cfi:Celf_1585 L-lactate d
 

 
3.972156e-6
 

 
1.0
 

 
Q6IVK8
 

 
0.288913
 

 
5.0
 

 
0.110526316
 

 
380
 

 
T
 

 
NIL
 

 

 
     

  starch biosynthesis     Total # of reactions in pathway = 5   Present reactions: 4
  

 

 
 Reaction 
 

 
 Protein(s) 
 

 

 

 
PGLUCISOM-RXN
 

 
(# )
 

 

 

 
PHOSPHOGLUCMUT-RXN
 

 
(# )
 

 

 

 
GLUC1PADENYLTRANS-RXN
 

 
(# )
 

 

 

 
RXN-7710
 

 
(# )
 

 

 
   Missing reactions: 1
  

 

 
 GLYCOGENSYN-RXN 
 

 

 
        Evidence for starch synthase, EC# 2.4.1.21   GLYCOGENSYN-RXN 
  

 

 
 Hit 
 

 
 Common 
 

 
 P 
 

 
 #Q 
 

 
 Best Qry 
 

 
 Best Eval 
 

 
 Avg Rank 
 

 
 Aln Len 
 

 
 Qry Len 
 

 
 IDOP? 
 

 
 adj? 
 

 

 

 
GKFG-1991-MONOMER
 

 
ahe:Arch_0927 glycogen sy
 

 
0.9836061
 

 
93.0
 

 
B5YG91
 

 
1.23498e-16
 

 
1.0
 

 
0.5655277
 

 
497
 

 
T
 

 
T
 

 

 

 
GKFG-1814-MONOMER
 

 
cfl:Cfla_1787 group 1 gly
 

 
2.175075e-4
 

 
15.0
 

 
P58395
 

 
1.79878e-6
 

 
2.0
 

 
0.29457405
 

 
438
 

 
T
 

 
NIL
 

 

 

 
GKFG-1046-MONOMER
 

 
xce:Xcel_0723 ATP-depende
 

 
2.5307965e-5
 

 
1.0
 

 
Q9EUT5
 

 
0.11956
 

 
2.0
 

 
0.26458332
 

 
480
 

 
T
 

 
NIL
 

 

 

 
GKFG-477-MONOMER
 

 
hypothetical protein (db=
 

 
2.9678517e-6
 

 
1.0
 

 
B8J8R4
 

 
0.579055
 

 
3.0
 

 
0.06918239
 

 
477
 

 
NIL
 

 
NIL
 

 

 

 
GKFG-566-MONOMER
 

 
cfl:Cfla_1720 deoxyxylulo
 

 
2.795795e-6
 

 
3.0
 

 
A5GKI7
 

 
0.0085899
 

 
2.0
 

 
0.14153872
 

 
524
 

 
T
 

 
NIL
 

 

 

 
GKFG-1051-MONOMER
 

 
aau:AAur_1266 exopolyphos
 

 
2.795795e-6
 

 
1.0
 

 
Q9FNF2
 

 
0.728748
 

 
2.0
 

 
0.148773
 

 
652
 

 
T
 

 
NIL
 

 

 

 
GKFG-1529-MONOMER
 

 
sco:SCO7547 SC5F1.01, SC8
 

 
2.795795e-6
 

 
1.0
 

 
O82627
 

 
0.158441
 

 
2.0
 

 
0.13486843
 

 
608
 

 
T
 

 
NIL
 

 

 

 
GKFG-957-MONOMER
 

 
mcu:HMPREF0573_10357 fusA
 

 
2.795795e-6
 

 
1.0
 

 
Q0BPL3
 

 
0.443686
 

 
2.0
 

 
0.12291667
 

 
480
 

 
T
 

 
NIL
 

 

 

 
GKFG-1024-MONOMER
 

 
glycosyl transferase fami
 

 
2.795795e-6
 

 
1.0
 

 
Q6MAS9
 

 
0.0768933
 

 
2.0
 

 
0.16
 

 
500
 

 
T
 

 
NIL
 

 

 

 
GKFG-1474-MONOMER
 

 
mcu:HMPREF0573_11576 pept
 

 
2.795795e-6
 

 
1.0
 

 
B2A6E4
 

 
0.439593
 

 
2.0
 

 
0.18951613
 

 
496
 

 
T
 

 
NIL
 

 

 

 
GKFG-1635-MONOMER
 

 
fbl:Fbal_3251 hypothetica
 

 
2.795795e-6
 

 
1.0
 

 
B2KE25
 

 
0.5614
 

 
2.0
 

 
0.14315353
 

 
482
 

 
T
 

 
NIL
 

 

 

 
GKFG-1867-MONOMER
 

 
Hly-III family protein; K
 

 
2.795795e-6
 

 
1.0
 

 
P0A6U8
 

 
0.596502
 

 
2.0
 

 
0.1341719
 

 
477
 

 
T
 

 
NIL
 

 

 

 
GKFG-281-MONOMER
 

 
bfa:Bfae_19800 copper/sil
 

 
2.5282066e-6
 

 
1.0
 

 
B8J8R4
 

 
0.348369
 

 
2.0
 

 
0.077568136
 

 
477
 

 
T
 

 
NIL
 

 

 

 
GKFG-1013-MONOMER
 

 
ahe:Arch_0427 YidE/YbjL d
 

 
2.5282066e-6
 

 
1.0
 

 
B3W9A1
 

 
0.242191
 

 
2.0
 

 
0.08108108
 

 
481
 

 
T
 

 
NIL
 

 

 

 
GKFG-1180-MONOMER
 

 
cde:CDHC02_1634 N-acetylm
 

 
2.5282066e-6
 

 
1.0
 

 
Q43846
 

 
0.288299
 

 
2.0
 

 
0.055284552
 

 
1230
 

 
T
 

 
NIL
 

 

 

 
GKFG-1087-MONOMER
 

 
146999..147151 - ( gc_con
 

 
2.5282066e-6
 

 
1.0
 

 
Q2RS50
 

 
0.334827
 

 
2.0
 

 
0.059793815
 

 
485
 

 
T
 

 
NIL
 

 

 

 
GKFG-419-MONOMER
 

 
family 2 glycosyl transfe
 

 
2.5282066e-6
 

 
1.0
 

 
B0SFJ4
 

 
0.888439
 

 
2.0
 

 
0.073529415
 

 
476
 

 
T
 

 
NIL
 

 

 

 
GKFG-1057-MONOMER
 

 
bbi:BBIF_1733 sialidase; 
 

 
2.5282066e-6
 

 
1.0
 

 
A9NFK6
 

 
0.192045
 

 
2.0
 

 
0.08995816
 

 
478
 

 
T
 

 
NIL
 

 

 

 
GKFG-232-MONOMER
 

 
cfi:Celf_0608 RNA methylt
 

 
2.5282066e-6
 

 
1.0
 

 
Q8UK38
 

 
0.747021
 

 
2.0
 

 
0.11394892
 

 
509
 

 
T
 

 
NIL
 

 

 

 
GKFG-1772-MONOMER
 

 
nca:Noca_1107 HsdR family
 

 
2.5282066e-6
 

 
1.0
 

 
Q0DDE3
 

 
0.920783
 

 
2.0
 

 
0.09382716
 

 
810
 

 
T
 

 
NIL
 

 

 

 
GKFG-651-MONOMER
 

 
mcu:HMPREF0573_11422 proS
 

 
1.2082764e-6
 

 
1.0
 

 
Q6CZK3
 

 
0.283704
 

 
3.0
 

 
0.1565762
 

 
479
 

 
T
 

 
NIL
 

 

 

 
GKFG-598-MONOMER
 

 
krh:KRH_14360 sigA; RNA p
 

 
1.0926308e-6
 

 
1.0
 

 
Q43847
 

 
0.753439
 

 
3.0
 

 
0.059973925
 

 
767
 

 
T
 

 
NIL
 

 

 

 
GKFG-659-MONOMER
 

 
ribosome recycling factor
 

 
1.0926308e-6
 

 
1.0
 

 
Q43846
 

 
0.66133
 

 
3.0
 

 
0.055284552
 

 
1230
 

 
T
 

 
NIL
 

 

 

 
GKFG-1792-MONOMER
 

 
cfl:Cfla_3205 bile acid:s
 

 
1.0926308e-6
 

 
1.0
 

 
B3W9A1
 

 
0.3946
 

 
3.0
 

 
0.10602911
 

 
481
 

 
T
 

 
NIL
 

 

 

 
GKFG-1785-MONOMER
 

 
mcu:HMPREF0573_10184 puta
 

 
1.0926308e-6
 

 
1.0
 

 
A5FUS8
 

 
0.527914
 

 
3.0
 

 
0.06263048
 

 
479
 

 
T
 

 
NIL
 

 

 

 
GKFG-331-MONOMER
 

 
NUDIX hydrolase (db=KEGG 
 

 
1.0926308e-6
 

 
1.0
 

 
Q1IWV2
 

 
0.0971355
 

 
3.0
 

 
0.109619685
 

 
447
 

 
T
 

 
NIL
 

 

 
     

  seleno-amino acid biosynthesis     Total # of reactions in pathway = 5   Present reactions: 3
  

 

 
 Reaction 
 

 
 Protein(s) 
 

 

 

 
RXN-12726
 

 
(# 
 # )
 

 

 

 
RXN-12730
 

 
(# )
 

 

 

 
SERINE-O-ACETTRAN-RXN
 

 
(# )
 

 

 
   Missing reactions: 2
  

 

 
 RXN-12728 
 

 

 

 
 RXN-12729 
 

 

 
        Evidence for , EC# 2.5.1.-   RXN-12728 
  

 

 
 Hit 
 

 
 Common 
 

 
 P 
 

 
 #Q 
 

 
 Best Qry 
 

 
 Best Eval 
 

 
 Avg Rank 
 

 
 Aln Len 
 

 
 Qry Len 
 

 
 IDOP? 
 

 
 adj? 
 

 

 

 
GKFG-1592-MONOMER
 

 
iva:Isova_2663 O-acetylho
 

 
0.999776
 

 
1.0
 

 
P55217
 

 
1.12456e-55
 

 
1.0
 

 
0.74422735
 

 
563
 

 
T
 

 
T
 

 

 
     Evidence for , EC# 4.4.1.8   RXN-12729      

  2-methylbutyrate biosynthesis     Total # of reactions in pathway = 5   Present reactions: 3
  

 

 
 Reaction 
 

 
 Protein(s) 
 

 

 

 
METHYLACETOACETYLCOATHIOL-RXN
 

 
(# )
 

 

 

 
PROPIONATE-COA-TRANSFERASE-RXN
 

 
(# )
 

 

 

 
2-METHYLACYL-COA-DEHYDROGENASE-RXN
 

 
(# )
 

 

 
   Missing reactions: 2
  

 

 
 1.1.1.178-RXN 
 

 

 

 
 TIGLYLCOA-HYDROXY-RXN 
 

 

 
        Evidence for 3-hydroxy-2-methylbutyryl-CoA dehydrogenase, EC# 1.1.1.178   1.1.1.178-RXN 
  

 

 
 Hit 
 

 
 Common 
 

 
 P 
 

 
 #Q 
 

 
 Best Qry 
 

 
 Best Eval 
 

 
 Avg Rank 
 

 
 Aln Len 
 

 
 Qry Len 
 

 
 IDOP? 
 

 
 adj? 
 

 

 

 
GKFG-720-MONOMER
 

 
fabG; 3-ketoacyl-ACP redu
 

 
0.8538447
 

 
2.0
 

 
O02691
 

 
6.32325e-29
 

 
1.0
 

 
1.0
 

 
261
 

 
T
 

 
NIL
 

 

 

 
GKFG-803-MONOMER
 

 
short-chain dehydrogenase
 

 
0.007427732
 

 
2.0
 

 
O02691
 

 
2.45176e-10
 

 
2.0
 

 
0.9594095
 

 
261
 

 
T
 

 
NIL
 

 

 

 
GKFG-246-MONOMER
 

 
bcv:Bcav_0731 Inositol 2-
 

 
2.9708795e-5
 

 
1.0
 

 
O18404
 

 
0.0177281
 

 
3.0
 

 
0.2784314
 

 
255
 

 
NIL
 

 
NIL
 

 

 
     Evidence for Tiglyl-CoA hydratase, EC# 4.2.1.17   TIGLYLCOA-HYDROXY-RXN 
  

 

 
 Hit 
 

 
 Common 
 

 
 P 
 

 
 #Q 
 

 
 Best Qry 
 

 
 Best Eval 
 

 
 Avg Rank 
 

 
 Aln Len 
 

 
 Qry Len 
 

 
 IDOP? 
 

 
 adj? 
 

 

 

 
GKFG-964-MONOMER
 

 
pak:HMPREF0675_3964 menB;
 

 
0.8249092
 

 
1.0
 

 
Q58DM8
 

 
1.38228e-16
 

 
1.0
 

 
0.9896552
 

 
290
 

 
T
 

 
NIL
 

 

 
     

  autoinducer AI-2 biosynthesis II ( Vibrio )     Total # of reactions in pathway = 3   Present reactions: 2
  

 

 
 Reaction 
 

 
 Protein(s) 
 

 

 

 
ADENOSYLHOMOCYSTEINE-NUCLEOSIDASE-RXN
 

 
(# )
 

 

 

 
RIBOSYLHOMOCYSTEINASE-RXN
 

 
(# )
 

 

 
   Missing reactions: 1
  

 

 
 RXN-7605 
 

 

 
        Evidence for , EC# 2.1.1.-   RXN-7605      

  autoinducer AI-2 biosynthesis I     Total # of reactions in pathway = 3   Present reactions: 2
  

 

 
 Reaction 
 

 
 Protein(s) 
 

 

 

 
ADENOSYLHOMOCYSTEINE-NUCLEOSIDASE-RXN
 

 
(# )
 

 

 

 
RIBOSYLHOMOCYSTEINASE-RXN
 

 
(# )
 

 

 
   Missing reactions: 1
  

 

 
 RXN-7605 
 

 

 
        Evidence for , EC# 2.1.1.-   RXN-7605      

  teichoic acid (poly-glycerol) biosynthesis     Total # of reactions in pathway = 8   Present reactions: 1
  

 

 
 Reaction 
 

 
 Protein(s) 
 

 

 

 
UDPGLCNACEPIM-RXN
 

 
(# )
 

 

 
   Missing reactions: 7
  

 

 
 TEICHOICSYN2-RXN 
 

 

 

 
 TEICHOICSYN3-RXN 
 

 

 

 
 TEICHOICSYN5-RXN 
 

 

 

 
 TEICHOICSYN8-RXN 
 

 

 

 
 TEICHOICSYN4-RXN 
 

 

 

 
 2.7.7.39-RXN 
 

 

 

 
 GLCNACPTRANS-RXN 
 

 

 
        Evidence for  N -acetylglucosaminyldiphosphoundecaprenol  N -acetyl-&beta;- D -mannosaminyltransferase, EC# 2.4.1.187   TEICHOICSYN2-RXN 
  
no BLAST hits found
 
     Evidence for , EC# 2.7.8.-   TEICHOICSYN3-RXN 
  

 

 
 Hit 
 

 
 Common 
 

 
 P 
 

 
 #Q 
 

 
 Best Qry 
 

 
 Best Eval 
 

 
 Avg Rank 
 

 
 Aln Len 
 

 
 Qry Len 
 

 
 IDOP? 
 

 
 adj? 
 

 

 

 
GKFG-1794-MONOMER
 

 
iva:Isova_1635 aspartyl-t
 

 
3.787886e-5
 

 
1.0
 

 
P27621
 

 
0.569188
 

 
1.0
 

 
0.0839895
 

 
381
 

 
NIL
 

 
NIL
 

 

 
     Evidence for , EC# 2.4.1.52   TEICHOICSYN5-RXN 
  

 

 
 Hit 
 

 
 Common 
 

 
 P 
 

 
 #Q 
 

 
 Best Qry 
 

 
 Best Eval 
 

 
 Avg Rank 
 

 
 Aln Len 
 

 
 Qry Len 
 

 
 IDOP? 
 

 
 adj? 
 

 

 

 
GKFG-1991-MONOMER
 

 
ahe:Arch_0927 glycogen sy
 

 
3.7905585e-4
 

 
1.0
 

 
P13484
 

 
0.0036545
 

 
1.0
 

 
0.24219911
 

 
673
 

 
NIL
 

 
NIL
 

 

 
     Evidence for , EC# 2.7.8.-   TEICHOICSYN8-RXN      Evidence for , EC# 2.7.8.12   TEICHOICSYN4-RXN 
  
no BLAST hits found
 
     Evidence for glycerol-3-phosphate cytidylyltransferase, EC# 2.7.7.39   2.7.7.39-RXN 
  

 

 
 Hit 
 

 
 Common 
 

 
 P 
 

 
 #Q 
 

 
 Best Qry 
 

 
 Best Eval 
 

 
 Avg Rank 
 

 
 Aln Len 
 

 
 Qry Len 
 

 
 IDOP? 
 

 
 adj? 
 

 

 

 
GKFG-737-MONOMER
 

 
Phosphopantetheine adenyl
 

 
3.7905585e-4
 

 
1.0
 

 
P27623
 

 
1.88193e-4
 

 
1.0
 

 
0.30232558
 

 
129
 

 
NIL
 

 
NIL
 

 

 

 
GKFG-1383-MONOMER
 

 
jde:Jden_1695 DEAD/DEAH b
 

 
1.5675386e-4
 

 
1.0
 

 
P27623
 

 
0.316051
 

 
2.0
 

 
0.43410853
 

 
129
 

 
NIL
 

 
NIL
 

 

 
     Evidence for UDP-GlcNAc:undecaprenyl-phosphate GlcNAc-1-phosphate transferase, EC# 2.7.8.33   GLCNACPTRANS-RXN 
  

 

 
 Hit 
 

 
 Common 
 

 
 P 
 

 
 #Q 
 

 
 Best Qry 
 

 
 Best Eval 
 

 
 Avg Rank 
 

 
 Aln Len 
 

 
 Qry Len 
 

 
 IDOP? 
 

 
 adj? 
 

 

 

 
GKFG-851-MONOMER
 

 
ske:Sked_09950 UDP-N-acet
 

 
0.98375326
 

 
2.0
 

 
O34753
 

 
1.1607e-50
 

 
1.0
 

 
0.9021052
 

 
358
 

 
NIL
 

 
NIL
 

 

 

 
GKFG-1943-MONOMER
 

 
bcv:Bcav_1945 P-type HAD 
 

 
7.594039e-6
 

 
1.0
 

 
O34753
 

 
0.115644
 

 
2.0
 

 
0.15363128
 

 
358
 

 
NIL
 

 
NIL
 

 

 
     

  UDP- N -acetylmuramoyl-pentapeptide biosynthesis II (lysine-containing)     Total # of reactions in pathway = 8   Present reactions: 7
  

 

 
 Reaction 
 

 
 Protein(s) 
 

 

 

 
UDPNACETYLGLUCOSAMENOLPYRTRANS-RXN
 

 
(# )
 

 

 

 
UDPNACETYLMURAMATEDEHYDROG-RXN
 

 
(# )
 

 

 

 
UDP-NACMUR-ALA-LIG-RXN
 

 
(# 
 # )
 

 

 

 
UDP-NACMURALA-GLU-LIG-RXN
 

 
(# )
 

 

 

 
GLUTRACE-RXN
 

 
(# )
 

 

 

 
DALADALALIG-RXN
 

 
(# )
 

 

 

 
6.3.2.10-RXN
 

 
(# )
 

 

 
   Missing reactions: 1
  

 

 
 6.3.2.7-RXN 
 

 

 
        Evidence for UDP- N -acetylmuramoyl- L -alanyl- D -glutamate&mdash; L -lysine ligase, EC# 6.3.2.7   6.3.2.7-RXN 
  

 

 
 Hit 
 

 
 Common 
 

 
 P 
 

 
 #Q 
 

 
 Best Qry 
 

 
 Best Eval 
 

 
 Avg Rank 
 

 
 Aln Len 
 

 
 Qry Len 
 

 
 IDOP? 
 

 
 adj? 
 

 

 

 
GKFG-16-MONOMER
 

 
(db=HMMPfam db_id=PF05738
 

 
1.3958463e-4
 

 
1.0
 

 
Q838A4
 

 
0.890994
 

 
1.0
 

 
0.27952754
 

 
508
 

 
T
 

 
NIL
 

 

 

 
GKFG-1796-MONOMER
 

 
pac:PPA0489 UTP-glucose-1
 

 
1.5421621e-5
 

 
1.0
 

 
Q8DYT2
 

 
0.0270483
 

 
1.0
 

 
0.19008264
 

 
484
 

 
T
 

 
NIL
 

 

 
     

  peptidoglycan biosynthesis III (mycobacteria)     Total # of reactions in pathway = 7   Present reactions: 5
  

 

 
 Reaction 
 

 
 Protein(s) 
 

 

 

 
RXN-11029
 

 
(# )
 

 

 

 
PWY-6387
 

 
NIL
 

 

 

 
RXN-11031
 

 
(# )
 

 

 

 
RXN-11301
 

 
(# 
 # 
 # )
 

 

 

 
2.4.1.129-RXN
 

 
(# 
 # 
 # )
 

 

 
   Missing reactions: 2
  

 

 
 RXN-11028 
 

 

 

 
 RXN-11030 
 

 

 
        Evidence for , EC# 2.7.8.-   RXN-11028 
  

 

 
 Hit 
 

 
 Common 
 

 
 P 
 

 
 #Q 
 

 
 Best Qry 
 

 
 Best Eval 
 

 
 Avg Rank 
 

 
 Aln Len 
 

 
 Qry Len 
 

 
 IDOP? 
 

 
 adj? 
 

 

 

 
GKFG-851-MONOMER
 

 
ske:Sked_09950 UDP-N-acet
 

 
0.8249092
 

 
1.0
 

 
P64259
 

 
1.05956e-16
 

 
1.0
 

 
1.0
 

 
359
 

 
T
 

 
NIL
 

 

 

 
GKFG-1869-MONOMER
 

 
mcu:HMPREF0573_10933 inte
 

 
2.5282066e-6
 

 
1.0
 

 
P64259
 

 
0.236795
 

 
2.0
 

 
0.06963788
 

 
359
 

 
T
 

 
NIL
 

 

 
     Evidence for    RXN-11030 
  

 

 
 Hit 
 

 
 Common 
 

 
 P 
 

 
 #Q 
 

 
 Best Qry 
 

 
 Best Eval 
 

 
 Avg Rank 
 

 
 Aln Len 
 

 
 Qry Len 
 

 
 IDOP? 
 

 
 adj? 
 

 

 

 
GKFG-825-MONOMER
 

 
seg (db=Seg db_id=seg fro
 

 
1.5421621e-5
 

 
1.0
 

 
O53638
 

 
0.125073
 

 
1.0
 

 
0.23505977
 

 
251
 

 
T
 

 
NIL
 

 

 
     

  peptidoglycan biosynthesis V (&beta;-lactam resistance)     Total # of reactions in pathway = 10   Present reactions: 5
  

 

 
 Reaction 
 

 
 Protein(s) 
 

 

 

 
PWY-6386
 

 
NIL
 

 

 

 
RXN-11347
 

 
(# )
 

 

 

 
RXN-11346
 

 
(# )
 

 

 

 
RXN-11348
 

 
(# 
 # 
 # )
 

 

 

 
2.4.1.129-RXN
 

 
(# 
 # 
 # )
 

 

 
   Missing reactions: 5
  

 

 
 3.4.17.14-RXN 
 

 

 

 
 RXN-11345 
 

 

 

 
 RXN-11344 
 

 

 

 
 RXN-11343 
 

 

 

 
 RXN-11349 
 

 

 
        Evidence for zinc  D -Ala- D -Ala carboxypeptidase, EC# 3.4.17.14   3.4.17.14-RXN 
  

 

 
 Hit 
 

 
 Common 
 

 
 P 
 

 
 #Q 
 

 
 Best Qry 
 

 
 Best Eval 
 

 
 Avg Rank 
 

 
 Aln Len 
 

 
 Qry Len 
 

 
 IDOP? 
 

 
 adj? 
 

 

 

 
GKFG-416-MONOMER
 

 
mcu:HMPREF0573_10944 hypo
 

 
0.0018147272
 

 
1.0
 

 
P00733
 

 
0.00389997
 

 
1.0
 

 
0.48235294
 

 
255
 

 
T
 

 
NIL
 

 

 
     Evidence for    RXN-11345      Evidence for , EC# 6.3.1.12   RXN-11344      Evidence for    RXN-11343      Evidence for , EC# 3.4.16.-   RXN-11349 
  
no BLAST hits found
 
     

  coenzyme M biosynthesis II     Total # of reactions in pathway = 6   Present reactions: 1
  

 

 
 Reaction 
 

 
 Protein(s) 
 

 

 

 
RXN-11737
 

 
(# )
 

 

 
   Missing reactions: 5
  

 

 
 R231-RXN 
 

 

 

 
 R232-RXN 
 

 

 

 
 R233-RXN 
 

 

 

 
 R234-RXN 
 

 

 

 
 RXN-11108 
 

 

 
        Evidence for sulfopyruvate decarboxylase, EC# 4.1.1.79   R231-RXN 
  

 

 
 Hit 
 

 
 Common 
 

 
 P 
 

 
 #Q 
 

 
 Best Qry 
 

 
 Best Eval 
 

 
 Avg Rank 
 

 
 Aln Len 
 

 
 Qry Len 
 

 
 IDOP? 
 

 
 adj? 
 

 

 

 
GKFG-1950-MONOMER
 

 
bcv:Bcav_2215 pyruvate ki
 

 
4.188785e-5
 

 
1.0
 

 
O27274
 

 
0.070742
 

 
1.0
 

 
0.2060606
 

 
165
 

 
NIL
 

 
NIL
 

 

 

 
GKFG-261-MONOMER
 

 
twh:TWT600 ABC transporte
 

 
1.5421621e-5
 

 
1.0
 

 
P58415
 

 
0.144626
 

 
1.0
 

 
0.19526628
 

 
169
 

 
T
 

 
NIL
 

 

 
     Evidence for    R232-RXN      Evidence for    R233-RXN      Evidence for    R234-RXN      Evidence for cysteate synthase, EC# 2.5.1.76   RXN-11108 
  

 

 
 Hit 
 

 
 Common 
 

 
 P 
 

 
 #Q 
 

 
 Best Qry 
 

 
 Best Eval 
 

 
 Avg Rank 
 

 
 Aln Len 
 

 
 Qry Len 
 

 
 IDOP? 
 

 
 adj? 
 

 

 

 
GKFG-1065-MONOMER
 

 
mcu:HMPREF0573_10379 thrC
 

 
0.97486
 

 
8.0
 

 
A7IA69
 

 
2.97592e-13
 

 
1.125
 

 
0.8771593
 

 
424
 

 
NIL
 

 
NIL
 

 

 

 
GKFG-966-MONOMER
 

 
cfi:Celf_1658 pyridoxal-p
 

 
4.188785e-5
 

 
1.0
 

 
A0B6Z6
 

 
0.00951012
 

 
1.0
 

 
0.13253012
 

 
415
 

 
NIL
 

 
NIL
 

 

 

 
GKFG-485-MONOMER
 

 
pfr:PFREUD_10040 ABC tran
 

 
7.594039e-6
 

 
1.0
 

 
A3CRP6
 

 
0.109848
 

 
2.0
 

 
0.1767442
 

 
430
 

 
NIL
 

 
NIL
 

 

 

 
GKFG-1067-MONOMER
 

 
iva:Isova_2318 diaminopim
 

 
7.594039e-6
 

 
1.0
 

 
D1Z042
 

 
0.991664
 

 
2.0
 

 
0.1615925
 

 
427
 

 
NIL
 

 
NIL
 

 

 

 
GKFG-1551-MONOMER
 

 
threonine dehydratase (EC
 

 
7.594039e-6
 

 
1.0
 

 
Q8Q0K3
 

 
0.0409708
 

 
2.0
 

 
0.20432693
 

 
416
 

 
NIL
 

 
NIL
 

 

 

 
GKFG-1294-MONOMER
 

 
hypothetical protein (db=
 

 
3.2819723e-6
 

 
2.0
 

 
Q8Q0K3
 

 
0.211666
 

 
3.0
 

 
0.14681605
 

 
416
 

 
NIL
 

 
NIL
 

 

 

 
GKFG-1425-MONOMER
 

 
mcu:HMPREF0573_10788 hypD
 

 
2.9678517e-6
 

 
1.0
 

 
A3CRP6
 

 
0.529079
 

 
3.0
 

 
0.093023255
 

 
430
 

 
NIL
 

 
NIL
 

 

 

 
GKFG-75-MONOMER
 

 
Bacterial adhesins (db=su
 

 
2.795795e-6
 

 
1.0
 

 
A7IA69
 

 
0.206663
 

 
2.0
 

 
0.18160377
 

 
424
 

 
T
 

 
NIL
 

 

 
     

  molybdenum cofactor biosynthesis     Total # of reactions in pathway = 7   Present reactions: 5
  

 

 
 Reaction 
 

 
 Protein(s) 
 

 

 

 
RXN-8342
 

 
NIL
 

 

 

 
RXN-8348
 

 
(# 
 # 
 # 
 # )
 

 

 

 
RXN-11361
 

 
NIL
 

 

 

 
RXN-12473
 

 
NIL
 

 

 

 
RXN0-308
 

 
(# 
 # )
 

 

 
   Missing reactions: 2
  

 

 
 RXN-8340 
 

 

 

 
 RXN-8344 
 

 

 
        Evidence for cyclic pyranopterin monophosphate synthase, EC# 4.1.99.18   RXN-8340 
  

 

 
 Hit 
 

 
 Common 
 

 
 P 
 

 
 #Q 
 

 
 Best Qry 
 

 
 Best Eval 
 

 
 Avg Rank 
 

 
 Aln Len 
 

 
 Qry Len 
 

 
 IDOP? 
 

 
 adj? 
 

 

 

 
GKFG-1526-MONOMER
 

 
bbv:HMPREF9228_0352 anaer
 

 
3.182795e-4
 

 
1.0
 

 
Q39055
 

 
0.00471257
 

 
1.0
 

 
0.47435898
 

 
390
 

 
T
 

 
NIL
 

 

 
     Evidence for molybdopterin adenylyltransferase, EC# 2.7.7.75   RXN-8344 
  

 

 
 Hit 
 

 
 Common 
 

 
 P 
 

 
 #Q 
 

 
 Best Qry 
 

 
 Best Eval 
 

 
 Avg Rank 
 

 
 Aln Len 
 

 
 Qry Len 
 

 
 IDOP? 
 

 
 adj? 
 

 

 

 
GKFG-203-MONOMER
 

 
moaB; Molybdenum cofactor
 

 
0.36918753
 

 
1.0
 

 
P0AF03
 

 
8.80216e-11
 

 
1.0
 

 
0.71794873
 

 
195
 

 
T
 

 
NIL
 

 

 
     

  NAD phosphorylation and dephosphorylation     Total # of reactions in pathway = 3   Present reactions: 2
  

 

 
 Reaction 
 

 
 Protein(s) 
 

 

 

 
NAD-KIN-RXN
 

 
(# )
 

 

 

 
TRANS-RXN0-277
 

 
(# 
 # )
 

 

 
   Missing reactions: 1
  

 

 
 RXN-5822 
 

 

 
        Evidence for , EC# 3.1.3.2   RXN-5822 
  
no BLAST hits found
 
     

  NAD biosynthesis I (from aspartate)     Total # of reactions in pathway = 6   Present reactions: 5
  

 

 
 Reaction 
 

 
 Protein(s) 
 

 

 

 
NICONUCADENYLYLTRAN-RXN
 

 
(# )
 

 

 

 
NAD-SYNTH-GLN-RXN
 

 
(# 
 # )
 

 

 

 
NAD-SYNTH-NH3-RXN
 

 
(# )
 

 

 

 
QUINOPRIBOTRANS-RXN
 

 
(# )
 

 

 

 
L-ASPARTATE-OXID-RXN
 

 
(# )
 

 

 
   Missing reactions: 1
  

 

 
 QUINOLINATE-SYNTHA-RXN 
 

 

 
        Evidence for quinolinate synthase, EC# 2.5.1.72   QUINOLINATE-SYNTHA-RXN 
  

 

 
 Hit 
 

 
 Common 
 

 
 P 
 

 
 #Q 
 

 
 Best Qry 
 

 
 Best Eval 
 

 
 Avg Rank 
 

 
 Aln Len 
 

 
 Qry Len 
 

 
 IDOP? 
 

 
 adj? 
 

 

 

 
GKFG-2018-MONOMER
 

 
jde:Jden_1127 Thiamin pyr
 

 
1.5421621e-5
 

 
1.0
 

 
Q65GN4
 

 
0.808345
 

 
1.0
 

 
0.17073171
 

 
369
 

 
T
 

 
NIL
 

 

 

 
GKFG-728-MONOMER
 

 
UniRef90_F2UVP8 Adenylyl 
 

 
1.5421621e-5
 

 
1.0
 

 
Q972D1
 

 
0.0194623
 

 
1.0
 

 
0.14641744
 

 
321
 

 
T
 

 
NIL
 

 

 

 
GKFG-1118-MONOMER
 

 
cdz:CD31A_0171 iutA; iron
 

 
1.5421621e-5
 

 
1.0
 

 
Q7UNR5
 

 
0.0452859
 

 
1.0
 

 
0.12755102
 

 
392
 

 
T
 

 
NIL
 

 

 

 
GKFG-372-MONOMER
 

 
bpb:bpr_I2635 feoB1; ferr
 

 
1.5421621e-5
 

 
1.0
 

 
Q8TME2
 

 
0.114156
 

 
1.0
 

 
0.20967741
 

 
310
 

 
T
 

 
NIL
 

 

 

 
GKFG-1988-MONOMER
 

 
cga:Celgi_1384 (Glutamate
 

 
1.5421621e-5
 

 
1.0
 

 
B8ZR90
 

 
0.106544
 

 
1.0
 

 
0.15056819
 

 
352
 

 
T
 

 
NIL
 

 

 

 
GKFG-331-MONOMER
 

 
NUDIX hydrolase (db=KEGG 
 

 
1.5421621e-5
 

 
1.0
 

 
A9B1Q2
 

 
0.0733861
 

 
1.0
 

 
0.17866667
 

 
375
 

 
T
 

 
NIL
 

 

 

 
GKFG-1492-MONOMER
 

 
ROK family protein; K0084
 

 
1.5421621e-5
 

 
1.0
 

 
Q7V7S6
 

 
0.415633
 

 
1.0
 

 
0.2072072
 

 
333
 

 
T
 

 
NIL
 

 

 
     

  NAD salvage pathway I     Total # of reactions in pathway = 8   Present reactions: 4
  

 

 
 Reaction 
 

 
 Protein(s) 
 

 

 

 
NICONUCADENYLYLTRAN-RXN
 

 
(# )
 

 

 

 
NADPYROPHOSPHAT-RXN
 

 
(# )
 

 

 

 
NICOTINATEPRIBOSYLTRANS-RXN
 

 
(# )
 

 

 

 
NAD-SYNTH-GLN-RXN
 

 
(# 
 # )
 

 

 
   Missing reactions: 4
  

 

 
 NMNAMIDOHYDRO-RXN 
 

 

 

 
 NADNUCLEOSID-RXN 
 

 

 

 
 NMNNUCLEOSID-RXN 
 

 

 

 
 NICOTINAMID-RXN 
 

 

 
        Evidence for nicotinamide-nucleotide amidase, EC# 3.5.1.42   NMNAMIDOHYDRO-RXN 
  
no BLAST hits found
 
     Evidence for NAD  +   nucleosidase, EC# 3.2.2.5   NADNUCLEOSID-RXN 
  
no BLAST hits found
 
     Evidence for NMN nucleosidase, EC# 3.2.2.14   NMNNUCLEOSID-RXN      Evidence for nicotinamidase, EC# 3.5.1.19   NICOTINAMID-RXN 
  
no BLAST hits found
 
     

  mono- trans , poly- cis  decaprenyl phosphate biosynthesis     Total # of reactions in pathway = 5   Present reactions: 2
  

 

 
 Reaction 
 

 
 Protein(s) 
 

 

 

 
2.5.1.68-RXN
 

 
(# )
 

 

 

 
GPPSYN-RXN
 

 
(# )
 

 

 
   Missing reactions: 3
  

 

 
 RXN-11023 
 

 

 

 
 IPPISOM-RXN 
 

 

 

 
 RXN-11027 
 

 

 
        Evidence for  trans , polycis -decaprenyl diphosphate synthase, EC# 2.5.1.86   RXN-11023 
  
no BLAST hits found
 
     Evidence for isopentenyl-diphosphate &Delta;-isomerase, EC# 5.3.3.2   IPPISOM-RXN 
  

 

 
 Hit 
 

 
 Common 
 

 
 P 
 

 
 #Q 
 

 
 Best Qry 
 

 
 Best Eval 
 

 
 Avg Rank 
 

 
 Aln Len 
 

 
 Qry Len 
 

 
 IDOP? 
 

 
 adj? 
 

 

 

 
GKFG-553-MONOMER
 

 
NUDIX hydrolase (db=KEGG 
 

 
0.0062982515
 

 
6.0
 

 
Q6D3F5
 

 
0.00677511
 

 
1.0
 

 
0.26905265
 

 
179
 

 
NIL
 

 
NIL
 

 

 

 
GKFG-1959-MONOMER
 

 
kra:Krad_1866 ATPase AAA;
 

 
0.0018147272
 

 
1.0
 

 
A5VK00
 

 
0.302708
 

 
1.0
 

 
0.4827586
 

 
348
 

 
T
 

 
NIL
 

 

 

 
GKFG-473-MONOMER
 

 
cfi:Celf_0593 phosphate A
 

 
3.7905585e-4
 

 
1.0
 

 
Q9HHE4
 

 
0.825297
 

 
1.0
 

 
0.26666668
 

 
360
 

 
NIL
 

 
NIL
 

 

 

 
GKFG-16-MONOMER
 

 
(db=HMMPfam db_id=PF05738
 

 
3.7905585e-4
 

 
1.0
 

 
Q989L5
 

 
0.144486
 

 
1.0
 

 
0.25356126
 

 
351
 

 
NIL
 

 
NIL
 

 

 

 
GKFG-1821-MONOMER
 

 
ahe:Arch_1786 queuine tRN
 

 
1.3958463e-4
 

 
1.0
 

 
A1BDG7
 

 
0.0727092
 

 
1.0
 

 
0.2644628
 

 
363
 

 
T
 

 
NIL
 

 

 

 
GKFG-820-MONOMER
 

 
periplasmic binding prote
 

 
1.3958463e-4
 

 
1.0
 

 
Q2RIU8
 

 
0.62249
 

 
1.0
 

 
0.25433525
 

 
346
 

 
T
 

 
NIL
 

 

 

 
GKFG-361-MONOMER
 

 
two-component system resp
 

 
6.873975e-5
 

 
1.0
 

 
Q5N019
 

 
0.45846
 

 
2.0
 

 
0.33908045
 

 
348
 

 
NIL
 

 
NIL
 

 

 

 
GKFG-1723-MONOMER
 

 
mcu:HMPREF0573_11697 hypo
 

 
5.771539e-5
 

 
1.0
 

 
Q8TX99
 

 
0.947296
 

 
2.0
 

 
0.36712328
 

 
365
 

 
T
 

 
NIL
 

 

 

 
GKFG-1657-MONOMER
 

 
cur:cur_0538 hypothetical
 

 
5.771539e-5
 

 
1.0
 

 
Q6D3F5
 

 
0.89502
 

 
2.0
 

 
0.47486034
 

 
179
 

 
T
 

 
NIL
 

 

 

 
GKFG-1954-MONOMER
 

 
bcv:Bcav_2235 Imidazole g
 

 
4.6728557e-5
 

 
7.0
 

 
Q5N019
 

 
0.259728
 

 
1.2857143
 

 
0.1824349
 

 
348
 

 
T
 

 
NIL
 

 

 

 
GKFG-424-MONOMER
 

 
ahe:Arch_0047 myo-inosito
 

 
4.188785e-5
 

 
2.0
 

 
Q67NT4
 

 
0.0066731
 

 
1.0
 

 
0.1753785
 

 
363
 

 
NIL
 

 
NIL
 

 

 

 
GKFG-184-MONOMER
 

 
npdA; Sir2 family NAD-dep
 

 
4.188785e-5
 

 
1.0
 

 
O26154
 

 
0.512534
 

 
1.0
 

 
0.12320917
 

 
349
 

 
NIL
 

 
NIL
 

 

 

 
GKFG-357-MONOMER
 

 
ske:Sked_08700 UDP-N-acet
 

 
4.188785e-5
 

 
1.0
 

 
A1WXH5
 

 
0.231409
 

 
1.0
 

 
0.17714286
 

 
175
 

 
NIL
 

 
NIL
 

 

 

 
GKFG-378-MONOMER
 

 
bbp:BBPR_0460 hypothetica
 

 
3.787886e-5
 

 
1.0
 

 
Q8TX99
 

 
0.654758
 

 
1.0
 

 
0.07945205
 

 
365
 

 
NIL
 

 
NIL
 

 

 

 
GKFG-1229-MONOMER
 

 
hypothetical protein; K09
 

 
2.5307965e-5
 

 
1.0
 

 
A7IAG4
 

 
0.907186
 

 
2.0
 

 
0.2506964
 

 
359
 

 
T
 

 
NIL
 

 

 

 
GKFG-988-MONOMER
 

 
Putative uncharacterized 
 

 
1.5421621e-5
 

 
2.0
 

 
Q9KWF6
 

 
0.14659
 

 
1.0
 

 
0.14457229
 

 
364
 

 
T
 

 
NIL
 

 

 

 
GKFG-1883-MONOMER
 

 
transmembrane_regions (db
 

 
1.5421621e-5
 

 
1.0
 

 
A7IAG4
 

 
0.519547
 

 
1.0
 

 
0.18662953
 

 
359
 

 
T
 

 
NIL
 

 

 

 
GKFG-1942-MONOMER
 

 
mcu:HMPREF0573_11680 puta
 

 
1.5421621e-5
 

 
1.0
 

 
C5D3G3
 

 
0.0869735
 

 
1.0
 

 
0.21776505
 

 
349
 

 
T
 

 
NIL
 

 

 

 
GKFG-1055-MONOMER
 

 
UniRef90_B5CMC4 Putative 
 

 
1.5421621e-5
 

 
1.0
 

 
Q0RBQ7
 

 
0.815905
 

 
1.0
 

 
0.15463917
 

 
194
 

 
T
 

 
NIL
 

 

 

 
GKFG-666-MONOMER
 

 
DNA protecting protein Dp
 

 
1.5421621e-5
 

 
1.0
 

 
Q9RVE2
 

 
0.620603
 

 
1.0
 

 
0.1923077
 

 
338
 

 
T
 

 
NIL
 

 

 

 
GKFG-890-MONOMER
 

 
aau:AAur_3646 inosine-uri
 

 
1.5421621e-5
 

 
1.0
 

 
Q88WB6
 

 
0.113454
 

 
1.0
 

 
0.123563215
 

 
348
 

 
T
 

 
NIL
 

 

 

 
GKFG-1988-MONOMER
 

 
cga:Celgi_1384 (Glutamate
 

 
1.5421621e-5
 

 
1.0
 

 
P15496
 

 
0.0417931
 

 
1.0
 

 
0.15625
 

 
288
 

 
T
 

 
NIL
 

 

 

 
GKFG-1617-MONOMER
 

 
exodeoxyribonuclease I su
 

 
1.3945618e-5
 

 
1.0
 

 
B1YA32
 

 
0.0876083
 

 
1.0
 

 
0.118644066
 

 
354
 

 
T
 

 
NIL
 

 

 

 
GKFG-858-MONOMER
 

 
pmmB; phosphomannomutase 
 

 
2.795795e-6
 

 
5.0
 

 
Q4ULD7
 

 
0.0326872
 

 
1.2
 

 
0.21881667
 

 
345
 

 
T
 

 
NIL
 

 

 

 
GKFG-1797-MONOMER
 

 
iva:Isova_1643 hypothetic
 

 
2.795795e-6
 

 
1.0
 

 
B1YA32
 

 
0.916221
 

 
2.0
 

 
0.14971751
 

 
354
 

 
T
 

 
NIL
 

 

 

 
GKFG-830-MONOMER
 

 
Putative uncharacterized 
 

 
2.795795e-6
 

 
1.0
 

 
P60923
 

 
0.969489
 

 
2.0
 

 
0.22950819
 

 
183
 

 
T
 

 
NIL
 

 

 

 
GKFG-1870-MONOMER
 

 
Acyl-CoA N-acyltransferas
 

 
2.5282066e-6
 

 
1.0
 

 
Q9RVE2
 

 
0.648577
 

 
2.0
 

 
0.10650887
 

 
338
 

 
T
 

 
NIL
 

 

 
     Evidence for , EC# 3.6.1.-   RXN-11027 
  

 

 
 Hit 
 

 
 Common 
 

 
 P 
 

 
 #Q 
 

 
 Best Qry 
 

 
 Best Eval 
 

 
 Avg Rank 
 

 
 Aln Len 
 

 
 Qry Len 
 

 
 IDOP? 
 

 
 adj? 
 

 

 

 
GKFG-1964-MONOMER
 

 
ahe:Arch_0909 undecapreno
 

 
0.97496676
 

 
1.0
 

 
O06239
 

 
4.95943e-73
 

 
1.0
 

 
0.9751773
 

 
282
 

 
T
 

 
NIL
 

 

 

 
GKFG-442-MONOMER
 

 
mcu:HMPREF0573_10293 glmS
 

 
1.5675386e-4
 

 
1.0
 

 
O06239
 

 
0.0945449
 

 
2.0
 

 
0.37943262
 

 
282
 

 
NIL
 

 
NIL
 

 

 
     

  methylerythritol phosphate pathway     Total # of reactions in pathway = 9   Present reactions: 8
  

 

 
 Reaction 
 

 
 Protein(s) 
 

 

 

 
DXS-RXN
 

 
(# )
 

 

 

 
DXPREDISOM-RXN
 

 
(# )
 

 

 

 
RXN0-302
 

 
(# )
 

 

 

 
RXN0-882
 

 
(# )
 

 

 

 
RXN0-884
 

 
(# )
 

 

 

 
ISPH2-RXN
 

 
(# )
 

 

 

 
2.7.1.148-RXN
 

 
(# )
 

 

 

 
2.7.7.60-RXN
 

 
(# )
 

 

 
   Missing reactions: 1
  

 

 
 IPPISOM-RXN 
 

 

 
        Evidence for isopentenyl-diphosphate &Delta;-isomerase, EC# 5.3.3.2   IPPISOM-RXN 
  

 

 
 Hit 
 

 
 Common 
 

 
 P 
 

 
 #Q 
 

 
 Best Qry 
 

 
 Best Eval 
 

 
 Avg Rank 
 

 
 Aln Len 
 

 
 Qry Len 
 

 
 IDOP? 
 

 
 adj? 
 

 

 

 
GKFG-553-MONOMER
 

 
NUDIX hydrolase (db=KEGG 
 

 
0.0062982515
 

 
6.0
 

 
Q6D3F5
 

 
0.00677511
 

 
1.0
 

 
0.26905265
 

 
179
 

 
NIL
 

 
NIL
 

 

 

 
GKFG-1959-MONOMER
 

 
kra:Krad_1866 ATPase AAA;
 

 
0.0018147272
 

 
1.0
 

 
A5VK00
 

 
0.302708
 

 
1.0
 

 
0.4827586
 

 
348
 

 
T
 

 
NIL
 

 

 

 
GKFG-473-MONOMER
 

 
cfi:Celf_0593 phosphate A
 

 
3.7905585e-4
 

 
1.0
 

 
Q9HHE4
 

 
0.825297
 

 
1.0
 

 
0.26666668
 

 
360
 

 
NIL
 

 
NIL
 

 

 

 
GKFG-1821-MONOMER
 

 
ahe:Arch_1786 queuine tRN
 

 
1.3958463e-4
 

 
1.0
 

 
A1BDG7
 

 
0.0727092
 

 
1.0
 

 
0.2644628
 

 
363
 

 
T
 

 
NIL
 

 

 

 
GKFG-820-MONOMER
 

 
periplasmic binding prote
 

 
1.3958463e-4
 

 
1.0
 

 
Q2RIU8
 

 
0.62249
 

 
1.0
 

 
0.25433525
 

 
346
 

 
T
 

 
NIL
 

 

 

 
GKFG-16-MONOMER
 

 
(db=HMMPfam db_id=PF05738
 

 
3.7905585e-4
 

 
1.0
 

 
Q989L5
 

 
0.144486
 

 
1.0
 

 
0.25356126
 

 
351
 

 
NIL
 

 
NIL
 

 

 

 
GKFG-1723-MONOMER
 

 
mcu:HMPREF0573_11697 hypo
 

 
5.771539e-5
 

 
1.0
 

 
Q8TX99
 

 
0.947296
 

 
2.0
 

 
0.36712328
 

 
365
 

 
T
 

 
NIL
 

 

 

 
GKFG-1657-MONOMER
 

 
cur:cur_0538 hypothetical
 

 
5.771539e-5
 

 
1.0
 

 
Q6D3F5
 

 
0.89502
 

 
2.0
 

 
0.47486034
 

 
179
 

 
T
 

 
NIL
 

 

 

 
GKFG-1954-MONOMER
 

 
bcv:Bcav_2235 Imidazole g
 

 
4.6728557e-5
 

 
7.0
 

 
Q5N019
 

 
0.259728
 

 
1.2857143
 

 
0.1824349
 

 
348
 

 
T
 

 
NIL
 

 

 

 
GKFG-1229-MONOMER
 

 
hypothetical protein; K09
 

 
2.5307965e-5
 

 
1.0
 

 
A7IAG4
 

 
0.907186
 

 
2.0
 

 
0.2506964
 

 
359
 

 
T
 

 
NIL
 

 

 

 
GKFG-361-MONOMER
 

 
two-component system resp
 

 
6.873975e-5
 

 
1.0
 

 
Q5N019
 

 
0.45846
 

 
2.0
 

 
0.33908045
 

 
348
 

 
NIL
 

 
NIL
 

 

 

 
GKFG-424-MONOMER
 

 
ahe:Arch_0047 myo-inosito
 

 
4.188785e-5
 

 
2.0
 

 
Q67NT4
 

 
0.0066731
 

 
1.0
 

 
0.1753785
 

 
363
 

 
NIL
 

 
NIL
 

 

 

 
GKFG-988-MONOMER
 

 
Putative uncharacterized 
 

 
1.5421621e-5
 

 
2.0
 

 
Q9KWF6
 

 
0.14659
 

 
1.0
 

 
0.14457229
 

 
364
 

 
T
 

 
NIL
 

 

 

 
GKFG-1883-MONOMER
 

 
transmembrane_regions (db
 

 
1.5421621e-5
 

 
1.0
 

 
A7IAG4
 

 
0.519547
 

 
1.0
 

 
0.18662953
 

 
359
 

 
T
 

 
NIL
 

 

 

 
GKFG-184-MONOMER
 

 
npdA; Sir2 family NAD-dep
 

 
4.188785e-5
 

 
1.0
 

 
O26154
 

 
0.512534
 

 
1.0
 

 
0.12320917
 

 
349
 

 
NIL
 

 
NIL
 

 

 

 
GKFG-1942-MONOMER
 

 
mcu:HMPREF0573_11680 puta
 

 
1.5421621e-5
 

 
1.0
 

 
C5D3G3
 

 
0.0869735
 

 
1.0
 

 
0.21776505
 

 
349
 

 
T
 

 
NIL
 

 

 

 
GKFG-1055-MONOMER
 

 
UniRef90_B5CMC4 Putative 
 

 
1.5421621e-5
 

 
1.0
 

 
Q0RBQ7
 

 
0.815905
 

 
1.0
 

 
0.15463917
 

 
194
 

 
T
 

 
NIL
 

 

 

 
GKFG-666-MONOMER
 

 
DNA protecting protein Dp
 

 
1.5421621e-5
 

 
1.0
 

 
Q9RVE2
 

 
0.620603
 

 
1.0
 

 
0.1923077
 

 
338
 

 
T
 

 
NIL
 

 

 

 
GKFG-890-MONOMER
 

 
aau:AAur_3646 inosine-uri
 

 
1.5421621e-5
 

 
1.0
 

 
Q88WB6
 

 
0.113454
 

 
1.0
 

 
0.123563215
 

 
348
 

 
T
 

 
NIL
 

 

 

 
GKFG-1988-MONOMER
 

 
cga:Celgi_1384 (Glutamate
 

 
1.5421621e-5
 

 
1.0
 

 
P15496
 

 
0.0417931
 

 
1.0
 

 
0.15625
 

 
288
 

 
T
 

 
NIL
 

 

 

 
GKFG-357-MONOMER
 

 
ske:Sked_08700 UDP-N-acet
 

 
4.188785e-5
 

 
1.0
 

 
A1WXH5
 

 
0.231409
 

 
1.0
 

 
0.17714286
 

 
175
 

 
NIL
 

 
NIL
 

 

 

 
GKFG-1617-MONOMER
 

 
exodeoxyribonuclease I su
 

 
1.3945618e-5
 

 
1.0
 

 
B1YA32
 

 
0.0876083
 

 
1.0
 

 
0.118644066
 

 
354
 

 
T
 

 
NIL
 

 

 

 
GKFG-378-MONOMER
 

 
bbp:BBPR_0460 hypothetica
 

 
3.787886e-5
 

 
1.0
 

 
Q8TX99
 

 
0.654758
 

 
1.0
 

 
0.07945205
 

 
365
 

 
NIL
 

 
NIL
 

 

 

 
GKFG-858-MONOMER
 

 
pmmB; phosphomannomutase 
 

 
2.795795e-6
 

 
5.0
 

 
Q4ULD7
 

 
0.0326872
 

 
1.2
 

 
0.21881667
 

 
345
 

 
T
 

 
NIL
 

 

 

 
GKFG-1797-MONOMER
 

 
iva:Isova_1643 hypothetic
 

 
2.795795e-6
 

 
1.0
 

 
B1YA32
 

 
0.916221
 

 
2.0
 

 
0.14971751
 

 
354
 

 
T
 

 
NIL
 

 

 

 
GKFG-830-MONOMER
 

 
Putative uncharacterized 
 

 
2.795795e-6
 

 
1.0
 

 
P60923
 

 
0.969489
 

 
2.0
 

 
0.22950819
 

 
183
 

 
T
 

 
NIL
 

 

 

 
GKFG-1870-MONOMER
 

 
Acyl-CoA N-acyltransferas
 

 
2.5282066e-6
 

 
1.0
 

 
Q9RVE2
 

 
0.648577
 

 
2.0
 

 
0.10650887
 

 
338
 

 
T
 

 
NIL
 

 

 
     

   trans, trans -farnesyl diphosphate biosynthesis     Total # of reactions in pathway = 3   Present reactions: 2
  

 

 
 Reaction 
 

 
 Protein(s) 
 

 

 

 
GPPSYN-RXN
 

 
(# )
 

 

 

 
FPPSYN-RXN
 

 
(# )
 

 

 
   Missing reactions: 1
  

 

 
 IPPISOM-RXN 
 

 

 
        Evidence for isopentenyl-diphosphate &Delta;-isomerase, EC# 5.3.3.2   IPPISOM-RXN 
  

 

 
 Hit 
 

 
 Common 
 

 
 P 
 

 
 #Q 
 

 
 Best Qry 
 

 
 Best Eval 
 

 
 Avg Rank 
 

 
 Aln Len 
 

 
 Qry Len 
 

 
 IDOP? 
 

 
 adj? 
 

 

 

 
GKFG-553-MONOMER
 

 
NUDIX hydrolase (db=KEGG 
 

 
0.0062982515
 

 
6.0
 

 
Q6D3F5
 

 
0.00677511
 

 
1.0
 

 
0.26905265
 

 
179
 

 
NIL
 

 
NIL
 

 

 

 
GKFG-1959-MONOMER
 

 
kra:Krad_1866 ATPase AAA;
 

 
0.0018147272
 

 
1.0
 

 
A5VK00
 

 
0.302708
 

 
1.0
 

 
0.4827586
 

 
348
 

 
T
 

 
NIL
 

 

 

 
GKFG-473-MONOMER
 

 
cfi:Celf_0593 phosphate A
 

 
3.7905585e-4
 

 
1.0
 

 
Q9HHE4
 

 
0.825297
 

 
1.0
 

 
0.26666668
 

 
360
 

 
NIL
 

 
NIL
 

 

 

 
GKFG-16-MONOMER
 

 
(db=HMMPfam db_id=PF05738
 

 
3.7905585e-4
 

 
1.0
 

 
Q989L5
 

 
0.144486
 

 
1.0
 

 
0.25356126
 

 
351
 

 
NIL
 

 
NIL
 

 

 

 
GKFG-1821-MONOMER
 

 
ahe:Arch_1786 queuine tRN
 

 
1.3958463e-4
 

 
1.0
 

 
A1BDG7
 

 
0.0727092
 

 
1.0
 

 
0.2644628
 

 
363
 

 
T
 

 
NIL
 

 

 

 
GKFG-820-MONOMER
 

 
periplasmic binding prote
 

 
1.3958463e-4
 

 
1.0
 

 
Q2RIU8
 

 
0.62249
 

 
1.0
 

 
0.25433525
 

 
346
 

 
T
 

 
NIL
 

 

 

 
GKFG-361-MONOMER
 

 
two-component system resp
 

 
6.873975e-5
 

 
1.0
 

 
Q5N019
 

 
0.45846
 

 
2.0
 

 
0.33908045
 

 
348
 

 
NIL
 

 
NIL
 

 

 

 
GKFG-1723-MONOMER
 

 
mcu:HMPREF0573_11697 hypo
 

 
5.771539e-5
 

 
1.0
 

 
Q8TX99
 

 
0.947296
 

 
2.0
 

 
0.36712328
 

 
365
 

 
T
 

 
NIL
 

 

 

 
GKFG-1657-MONOMER
 

 
cur:cur_0538 hypothetical
 

 
5.771539e-5
 

 
1.0
 

 
Q6D3F5
 

 
0.89502
 

 
2.0
 

 
0.47486034
 

 
179
 

 
T
 

 
NIL
 

 

 

 
GKFG-1954-MONOMER
 

 
bcv:Bcav_2235 Imidazole g
 

 
4.6728557e-5
 

 
7.0
 

 
Q5N019
 

 
0.259728
 

 
1.2857143
 

 
0.1824349
 

 
348
 

 
T
 

 
NIL
 

 

 

 
GKFG-424-MONOMER
 

 
ahe:Arch_0047 myo-inosito
 

 
4.188785e-5
 

 
2.0
 

 
Q67NT4
 

 
0.0066731
 

 
1.0
 

 
0.1753785
 

 
363
 

 
NIL
 

 
NIL
 

 

 

 
GKFG-184-MONOMER
 

 
npdA; Sir2 family NAD-dep
 

 
4.188785e-5
 

 
1.0
 

 
O26154
 

 
0.512534
 

 
1.0
 

 
0.12320917
 

 
349
 

 
NIL
 

 
NIL
 

 

 

 
GKFG-357-MONOMER
 

 
ske:Sked_08700 UDP-N-acet
 

 
4.188785e-5
 

 
1.0
 

 
A1WXH5
 

 
0.231409
 

 
1.0
 

 
0.17714286
 

 
175
 

 
NIL
 

 
NIL
 

 

 

 
GKFG-378-MONOMER
 

 
bbp:BBPR_0460 hypothetica
 

 
3.787886e-5
 

 
1.0
 

 
Q8TX99
 

 
0.654758
 

 
1.0
 

 
0.07945205
 

 
365
 

 
NIL
 

 
NIL
 

 

 

 
GKFG-1229-MONOMER
 

 
hypothetical protein; K09
 

 
2.5307965e-5
 

 
1.0
 

 
A7IAG4
 

 
0.907186
 

 
2.0
 

 
0.2506964
 

 
359
 

 
T
 

 
NIL
 

 

 

 
GKFG-988-MONOMER
 

 
Putative uncharacterized 
 

 
1.5421621e-5
 

 
2.0
 

 
Q9KWF6
 

 
0.14659
 

 
1.0
 

 
0.14457229
 

 
364
 

 
T
 

 
NIL
 

 

 

 
GKFG-1883-MONOMER
 

 
transmembrane_regions (db
 

 
1.5421621e-5
 

 
1.0
 

 
A7IAG4
 

 
0.519547
 

 
1.0
 

 
0.18662953
 

 
359
 

 
T
 

 
NIL
 

 

 

 
GKFG-1942-MONOMER
 

 
mcu:HMPREF0573_11680 puta
 

 
1.5421621e-5
 

 
1.0
 

 
C5D3G3
 

 
0.0869735
 

 
1.0
 

 
0.21776505
 

 
349
 

 
T
 

 
NIL
 

 

 

 
GKFG-1055-MONOMER
 

 
UniRef90_B5CMC4 Putative 
 

 
1.5421621e-5
 

 
1.0
 

 
Q0RBQ7
 

 
0.815905
 

 
1.0
 

 
0.15463917
 

 
194
 

 
T
 

 
NIL
 

 

 

 
GKFG-666-MONOMER
 

 
DNA protecting protein Dp
 

 
1.5421621e-5
 

 
1.0
 

 
Q9RVE2
 

 
0.620603
 

 
1.0
 

 
0.1923077
 

 
338
 

 
T
 

 
NIL
 

 

 

 
GKFG-890-MONOMER
 

 
aau:AAur_3646 inosine-uri
 

 
1.5421621e-5
 

 
1.0
 

 
Q88WB6
 

 
0.113454
 

 
1.0
 

 
0.123563215
 

 
348
 

 
T
 

 
NIL
 

 

 

 
GKFG-1988-MONOMER
 

 
cga:Celgi_1384 (Glutamate
 

 
1.5421621e-5
 

 
1.0
 

 
P15496
 

 
0.0417931
 

 
1.0
 

 
0.15625
 

 
288
 

 
T
 

 
NIL
 

 

 

 
GKFG-1617-MONOMER
 

 
exodeoxyribonuclease I su
 

 
1.3945618e-5
 

 
1.0
 

 
B1YA32
 

 
0.0876083
 

 
1.0
 

 
0.118644066
 

 
354
 

 
T
 

 
NIL
 

 

 

 
GKFG-858-MONOMER
 

 
pmmB; phosphomannomutase 
 

 
2.795795e-6
 

 
5.0
 

 
Q4ULD7
 

 
0.0326872
 

 
1.2
 

 
0.21881667
 

 
345
 

 
T
 

 
NIL
 

 

 

 
GKFG-1797-MONOMER
 

 
iva:Isova_1643 hypothetic
 

 
2.795795e-6
 

 
1.0
 

 
B1YA32
 

 
0.916221
 

 
2.0
 

 
0.14971751
 

 
354
 

 
T
 

 
NIL
 

 

 

 
GKFG-830-MONOMER
 

 
Putative uncharacterized 
 

 
2.795795e-6
 

 
1.0
 

 
P60923
 

 
0.969489
 

 
2.0
 

 
0.22950819
 

 
183
 

 
T
 

 
NIL
 

 

 

 
GKFG-1870-MONOMER
 

 
Acyl-CoA N-acyltransferas
 

 
2.5282066e-6
 

 
1.0
 

 
Q9RVE2
 

 
0.648577
 

 
2.0
 

 
0.10650887
 

 
338
 

 
T
 

 
NIL
 

 

 
     

  superpathway of heme biosynthesis from uroporphyrinogen-III     Total # of reactions in pathway = 4   Present reactions: 2
  

 

 
 Reaction 
 

 
 Protein(s) 
 

 

 

 
UROGENDECARBOX-RXN
 

 
(# )
 

 

 

 
PROTOHEMEFERROCHELAT-RXN
 

 
(# )
 

 

 
   Missing reactions: 2
  

 

 
 PROTOPORGENOXI-RXN 
 

 

 

 
 RXN0-1461 
 

 

 
        Evidence for protoporphyrinogen oxidase, EC# 1.3.3.4   PROTOPORGENOXI-RXN 
  

 

 
 Hit 
 

 
 Common 
 

 
 P 
 

 
 #Q 
 

 
 Best Qry 
 

 
 Best Eval 
 

 
 Avg Rank 
 

 
 Aln Len 
 

 
 Qry Len 
 

 
 IDOP? 
 

 
 adj? 
 

 

 

 
GKFG-1193-MONOMER
 

 
pfr:PFREUD_12990 glpA; an
 

 
0.013736963
 

 
1.0
 

 
P56602
 

 
0.602915
 

 
1.0
 

 
0.60714287
 

 
28
 

 
NIL
 

 
NIL
 

 

 

 
GKFG-795-MONOMER
 

 
bcv:Bcav_1171 UDP-galacto
 

 
4.188785e-5
 

 
3.0
 

 
O32434
 

 
0.0216156
 

 
1.0
 

 
0.21702842
 

 
527
 

 
NIL
 

 
NIL
 

 

 

 
GKFG-772-MONOMER
 

 
cell surface protein (db=
 

 
4.188785e-5
 

 
1.0
 

 
P32397
 

 
0.0268242
 

 
1.0
 

 
0.14893617
 

 
470
 

 
NIL
 

 
NIL
 

 

 

 
GKFG-1723-MONOMER
 

 
mcu:HMPREF0573_11697 hypo
 

 
4.188785e-5
 

 
1.0
 

 
P56601
 

 
0.24699
 

 
1.0
 

 
0.13375796
 

 
471
 

 
NIL
 

 
NIL
 

 

 

 
GKFG-1192-MONOMER
 

 
pfr:PFREUD_12980 glpB; an
 

 
3.787886e-5
 

 
1.0
 

 
Q94IG7
 

 
0.446095
 

 
1.0
 

 
0.054613937
 

 
531
 

 
NIL
 

 
NIL
 

 

 

 
GKFG-445-MONOMER
 

 
toxin-antitoxin system, t
 

 
1.5421621e-5
 

 
1.0
 

 
P0ACB4
 

 
0.161445
 

 
1.0
 

 
0.22099447
 

 
181
 

 
T
 

 
NIL
 

 

 

 
GKFG-1305-MONOMER
 

 
5,10-methenyltetrahydrofo
 

 
7.594039e-6
 

 
1.0
 

 
P0A5A7
 

 
0.343379
 

 
2.0
 

 
0.19469027
 

 
452
 

 
NIL
 

 
NIL
 

 

 

 
GKFG-900-MONOMER
 

 
ahe:Arch_1354 electron-tr
 

 
6.867209e-6
 

 
3.0
 

 
P40012
 

 
0.118097
 

 
1.6666666
 

 
0.11888475
 

 
539
 

 
NIL
 

 
NIL
 

 

 

 
GKFG-497-MONOMER
 

 
nrdG; anaerobic ribonucle
 

 
6.867209e-6
 

 
1.0
 

 
O24163
 

 
0.289888
 

 
2.0
 

 
0.09854015
 

 
548
 

 
NIL
 

 
NIL
 

 

 

 
GKFG-611-MONOMER
 

 
Peptidyl-prolyl cis-trans
 

 
6.867209e-6
 

 
1.0
 

 
P40012
 

 
0.662416
 

 
2.0
 

 
0.051948052
 

 
539
 

 
NIL
 

 
NIL
 

 

 

 
GKFG-332-MONOMER
 

 
mcu:HMPREF0573_10437 ppk;
 

 
1.2082764e-6
 

 
1.0
 

 
P0A5A7
 

 
0.915227
 

 
3.0
 

 
0.17035398
 

 
452
 

 
T
 

 
NIL
 

 

 
     Evidence for coproporphyrinogen oxidase, EC# 1.3.3.3   RXN0-1461 
  

 

 
 Hit 
 

 
 Common 
 

 
 P 
 

 
 #Q 
 

 
 Best Qry 
 

 
 Best Eval 
 

 
 Avg Rank 
 

 
 Aln Len 
 

 
 Qry Len 
 

 
 IDOP? 
 

 
 adj? 
 

 

 

 
GKFG-225-MONOMER
 

 
mcu:HMPREF0573_10801 pckG
 

 
1.5421621e-5
 

 
1.0
 

 
Q54IA7
 

 
0.31405
 

 
1.0
 

 
0.18975903
 

 
332
 

 
T
 

 
NIL
 

 

 
     

  1,4-dihydroxy-2-naphthoate biosynthesis I     Total # of reactions in pathway = 7   Present reactions: 5
  

 

 
 Reaction 
 

 
 Protein(s) 
 

 

 

 
O-SUCCINYLBENZOATE-COA-LIG-RXN
 

 
(# )
 

 

 

 
O-SUCCINYLBENZOATE-COA-SYN-RXN
 

 
(# )
 

 

 

 
ISOCHORSYN-RXN
 

 
(# )
 

 

 

 
2.5.1.64-RXN
 

 
(# )
 

 

 

 
NAPHTHOATE-SYN-RXN
 

 
(# )
 

 

 
   Missing reactions: 2
  

 

 
 RXN-9310 
 

 

 

 
 RXN-9311 
 

 

 
        Evidence for 2-succinyl-6-hydroxy-2,4-cyclohexadiene-1-carboxylate synthase, EC# 4.2.99.20   RXN-9310 
  

 

 
 Hit 
 

 
 Common 
 

 
 P 
 

 
 #Q 
 

 
 Best Qry 
 

 
 Best Eval 
 

 
 Avg Rank 
 

 
 Aln Len 
 

 
 Qry Len 
 

 
 IDOP? 
 

 
 adj? 
 

 

 

 
GKFG-961-MONOMER
 

 
2-succinyl-5-enolpyruvyl-
 

 
0.47213823
 

 
1.0
 

 
Q15KI9
 

 
8.3716e-16
 

 
1.0
 

 
0.113119535
 

 
1715
 

 
T
 

 
T
 

 

 

 
GKFG-836-MONOMER
 

 
alpha/beta hydrolase (db=
 

 
0.026486674
 

 
6.0
 

 
A4WCP8
 

 
1.55489e-5
 

 
1.8333334
 

 
0.73743796
 

 
258
 

 
T
 

 
NIL
 

 

 

 
GKFG-1906-MONOMER
 

 
mcu:HMPREF0573_11357 puta
 

 
0.015300237
 

 
6.0
 

 
A8GGZ0
 

 
7.61997e-8
 

 
2.0
 

 
0.6615186
 

 
255
 

 
T
 

 
NIL
 

 

 

 
GKFG-962-MONOMER
 

 
menC; O-succinylbenzoate-
 

 
0.0015685969
 

 
1.0
 

 
Q15KI9
 

 
5.87414e-9
 

 
2.0
 

 
0.13877551
 

 
1715
 

 
T
 

 
T
 

 

 

 
GKFG-372-MONOMER
 

 
bpb:bpr_I2635 feoB1; ferr
 

 
0.0014584069
 

 
1.0
 

 
A8GGZ0
 

 
0.0121158
 

 
5.0
 

 
0.61960787
 

 
255
 

 
T
 

 
NIL
 

 

 

 
GKFG-864-MONOMER
 

 
crd:CRES_0981 pip; prolin
 

 
5.175587e-4
 

 
2.0
 

 
A4WCP8
 

 
0.0428064
 

 
4.5
 

 
0.5109211
 

 
258
 

 
T
 

 
NIL
 

 

 

 
GKFG-1169-MONOMER
 

 
UniRef90_E6M3C9 Acyl-CoA 
 

 
1.6156636e-6
 

 
2.0
 

 
A8GGZ0
 

 
0.00410829
 

 
3.5
 

 
0.2253113
 

 
255
 

 
T
 

 
NIL
 

 

 

 
GKFG-1003-MONOMER
 

 
mcu:HMPREF0573_10303 xylu
 

 
1.6156636e-6
 

 
1.0
 

 
A4WCP8
 

 
0.751663
 

 
4.0
 

 
0.20542635
 

 
258
 

 
T
 

 
NIL
 

 

 

 
GKFG-1919-MONOMER
 

 
putative acyl-CoA thioest
 

 
1.2082764e-6
 

 
4.0
 

 
A8GGZ0
 

 
0.00127649
 

 
2.25
 

 
0.23798625
 

 
255
 

 
T
 

 
NIL
 

 

 

 
GKFG-1012-MONOMER
 

 
krh:KRH_10210 ilvB; aceto
 

 
1.0926308e-6
 

 
1.0
 

 
Q15KI9
 

 
3.19572e-4
 

 
3.0
 

 
0.09154519
 

 
1715
 

 
T
 

 
NIL
 

 

 
     Evidence for 1,4-dihydroxy-2-naphthoyl-CoA hydrolase, EC# 3.1.2.28   RXN-9311 
  

 

 
 Hit 
 

 
 Common 
 

 
 P 
 

 
 #Q 
 

 
 Best Qry 
 

 
 Best Eval 
 

 
 Avg Rank 
 

 
 Aln Len 
 

 
 Qry Len 
 

 
 IDOP? 
 

 
 adj? 
 

 

 

 
GKFG-1834-MONOMER
 

 
pseudouridine synthase; K
 

 
0.008898924
 

 
1.0
 

 
A5GP76
 

 
0.0230322
 

 
1.0
 

 
0.83006537
 

 
153
 

 
T
 

 
NIL
 

 

 

 
GKFG-1942-MONOMER
 

 
mcu:HMPREF0573_11680 puta
 

 
3.182795e-4
 

 
1.0
 

 
Q55777
 

 
0.180808
 

 
1.0
 

 
0.4057971
 

 
138
 

 
T
 

 
NIL
 

 

 

 
GKFG-1303-MONOMER
 

 
mcu:HMPREF0573_10204 phag
 

 
1.5421621e-5
 

 
1.0
 

 
B0CEN8
 

 
0.134384
 

 
1.0
 

 
0.16788322
 

 
137
 

 
T
 

 
NIL
 

 

 

 
GKFG-1112-MONOMER
 

 
jde:Jden_1798 iron-contai
 

 
1.5421621e-5
 

 
1.0
 

 
B1XL26
 

 
0.357236
 

 
1.0
 

 
0.1849315
 

 
146
 

 
T
 

 
NIL
 

 

 
     

  ubiquinol-6 biosynthesis (eukaryotic)     Total # of reactions in pathway = 8   Present reactions: 1
  

 

 
 Reaction 
 

 
 Protein(s) 
 

 

 

 
RXN3O-54
 

 
(# )
 

 

 
   Missing reactions: 7
  

 

 
 RXN3O-58 
 

 

 

 
 2.1.1.114-RXN 
 

 

 

 
 RXN3O-73 
 

 

 

 
 RXN3O-12 
 

 

 

 
 RXN3O-75 
 

 

 

 
 RXN3O-102 
 

 

 

 
 RXN-9003 
 

 

 
        Evidence for , EC# 1.14.13.-   RXN3O-58      Evidence for hexaprenyldihydroxybenzoate methyltransferase, EC# 2.1.1.114   2.1.1.114-RXN 
  

 

 
 Hit 
 

 
 Common 
 

 
 P 
 

 
 #Q 
 

 
 Best Qry 
 

 
 Best Eval 
 

 
 Avg Rank 
 

 
 Aln Len 
 

 
 Qry Len 
 

 
 IDOP? 
 

 
 adj? 
 

 

 

 
GKFG-860-MONOMER
 

 
msm:MSMEG_3094 oxidoreduc
 

 
3.7905585e-4
 

 
1.0
 

 
P27680
 

 
0.00663104
 

 
1.0
 

 
0.28846154
 

 
312
 

 
NIL
 

 
NIL
 

 

 

 
GKFG-855-MONOMER
 

 
methylase of polypeptide 
 

 
7.594039e-6
 

 
1.0
 

 
P27680
 

 
0.0133433
 

 
2.0
 

 
0.23076923
 

 
312
 

 
NIL
 

 
NIL
 

 

 
     Evidence for , EC# 4.1.1.-   RXN3O-73      Evidence for , EC# 1.14.13.-   RXN3O-12 
  

 

 
 Hit 
 

 
 Common 
 

 
 P 
 

 
 #Q 
 

 
 Best Qry 
 

 
 Best Eval 
 

 
 Avg Rank 
 

 
 Aln Len 
 

 
 Qry Len 
 

 
 IDOP? 
 

 
 adj? 
 

 

 

 
GKFG-865-MONOMER
 

 
bfa:Bfae_24100 IMP cycloh
 

 
6.873975e-5
 

 
1.0
 

 
P53318
 

 
0.188963
 

 
2.0
 

 
0.2651357
 

 
479
 

 
NIL
 

 
NIL
 

 

 

 
GKFG-1289-MONOMER
 

 
cpg:Cp316_1400 glgP; glyc
 

 
4.188785e-5
 

 
1.0
 

 
P53318
 

 
0.081293
 

 
1.0
 

 
0.22338204
 

 
479
 

 
NIL
 

 
NIL
 

 

 

 
GKFG-1193-MONOMER
 

 
pfr:PFREUD_12990 glpA; an
 

 
3.2819723e-6
 

 
1.0
 

 
P53318
 

 
0.294768
 

 
3.0
 

 
0.1920668
 

 
479
 

 
NIL
 

 
NIL
 

 

 
     Evidence for , EC# 1.14.-.-   RXN3O-75 
  
no BLAST hits found
 
     Evidence for 3-demethylubiquinol-6 3-O-methyltransferase, EC# 2.1.1.64   RXN3O-102 
  

 

 
 Hit 
 

 
 Common 
 

 
 P 
 

 
 #Q 
 

 
 Best Qry 
 

 
 Best Eval 
 

 
 Avg Rank 
 

 
 Aln Len 
 

 
 Qry Len 
 

 
 IDOP? 
 

 
 adj? 
 

 

 

 
GKFG-860-MONOMER
 

 
msm:MSMEG_3094 oxidoreduc
 

 
3.7905585e-4
 

 
1.0
 

 
P27680
 

 
0.00663104
 

 
1.0
 

 
0.28846154
 

 
312
 

 
NIL
 

 
NIL
 

 

 

 
GKFG-855-MONOMER
 

 
methylase of polypeptide 
 

 
7.594039e-6
 

 
1.0
 

 
P27680
 

 
0.0133433
 

 
2.0
 

 
0.23076923
 

 
312
 

 
NIL
 

 
NIL
 

 

 
     Evidence for , EC# 2.5.1.39   RXN-9003 
  

 

 
 Hit 
 

 
 Common 
 

 
 P 
 

 
 #Q 
 

 
 Best Qry 
 

 
 Best Eval 
 

 
 Avg Rank 
 

 
 Aln Len 
 

 
 Qry Len 
 

 
 IDOP? 
 

 
 adj? 
 

 

 

 
GKFG-997-MONOMER
 

 
ssm:Spirs_0789 aldehyde d
 

 
3.7905585e-4
 

 
1.0
 

 
P32378
 

 
0.0306776
 

 
1.0
 

 
0.25268817
 

 
372
 

 
NIL
 

 
NIL
 

 

 
     

  ubiquinol-8 biosynthesis (prokaryotic)     Total # of reactions in pathway = 8   Present reactions: 1
  

 

 
 Reaction 
 

 
 Protein(s) 
 

 

 

 
2-OCTAPRENYL-METHOXY-BENZOQ-METH-RXN
 

 
(# )
 

 

 
   Missing reactions: 7
  

 

 
 OCTAPRENYL-METHYL-METHOXY-BENZOQ-OH-RXN 
 

 

 

 
 DHHB-METHYLTRANSFER-RXN 
 

 

 

 
 2-OCTAPRENYL-6-METHOXYPHENOL-HYDROX-RXN 
 

 

 

 
 2-OCTAPRENYL-6-OHPHENOL-METHY-RXN 
 

 

 

 
 2-OCTAPRENYLPHENOL-HYDROX-RXN 
 

 

 

 
 3-OCTAPRENYL-4-OHBENZOATE-DECARBOX-RXN 
 

 

 

 
 4OHBENZOATE-OCTAPRENYLTRANSFER-RXN 
 

 

 
        Evidence for , EC# 1.14.-.-   OCTAPRENYL-METHYL-METHOXY-BENZOQ-OH-RXN 
  

 

 
 Hit 
 

 
 Common 
 

 
 P 
 

 
 #Q 
 

 
 Best Qry 
 

 
 Best Eval 
 

 
 Avg Rank 
 

 
 Aln Len 
 

 
 Qry Len 
 

 
 IDOP? 
 

 
 adj? 
 

 

 

 
GKFG-176-MONOMER
 

 
bcv:Bcav_3230 geranylgera
 

 
5.7020097e-4
 

 
1.0
 

 
P75728
 

 
0.0810031
 

 
1.0
 

 
0.0971867
 

 
391
 

 
T
 

 
T
 

 

 
     Evidence for 3-demethylubiquinone-8 3-O-methyltransferase, EC# 2.1.1.64   DHHB-METHYLTRANSFER-RXN 
  

 

 
 Hit 
 

 
 Common 
 

 
 P 
 

 
 #Q 
 

 
 Best Qry 
 

 
 Best Eval 
 

 
 Avg Rank 
 

 
 Aln Len 
 

 
 Qry Len 
 

 
 IDOP? 
 

 
 adj? 
 

 

 

 
GKFG-177-MONOMER
 

 
ubiE; methylase; K03183 u
 

 
0.36183572
 

 
1.0
 

 
P17993
 

 
2.2385e-7
 

 
1.0
 

 
0.9125
 

 
240
 

 
T
 

 
NIL
 

 

 

 
GKFG-476-MONOMER
 

 
SAM-dependent methyltrans
 

 
1.5675386e-4
 

 
1.0
 

 
P17993
 

 
6.20499e-6
 

 
2.0
 

 
0.425
 

 
240
 

 
NIL
 

 
NIL
 

 

 

 
GKFG-1443-MONOMER
 

 
methyltransferase small (
 

 
2.9708795e-5
 

 
1.0
 

 
P17993
 

 
0.0934736
 

 
3.0
 

 
0.29166666
 

 
240
 

 
NIL
 

 
NIL
 

 

 
     Evidence for , EC# 1.14.13.-   2-OCTAPRENYL-6-METHOXYPHENOL-HYDROX-RXN 
  

 

 
 Hit 
 

 
 Common 
 

 
 P 
 

 
 #Q 
 

 
 Best Qry 
 

 
 Best Eval 
 

 
 Avg Rank 
 

 
 Aln Len 
 

 
 Qry Len 
 

 
 IDOP? 
 

 
 adj? 
 

 

 

 
GKFG-900-MONOMER
 

 
ahe:Arch_1354 electron-tr
 

 
0.004913946
 

 
1.0
 

 
P25534
 

 
0.0245448
 

 
1.0
 

 
0.57908165
 

 
392
 

 
NIL
 

 
NIL
 

 

 

 
GKFG-225-MONOMER
 

 
mcu:HMPREF0573_10801 pckG
 

 
2.5282066e-6
 

 
1.0
 

 
P25534
 

 
0.624331
 

 
2.0
 

 
0.10714286
 

 
392
 

 
T
 

 
NIL
 

 

 
     Evidence for 2-octaprenyl-6-hydroxyphenyl methylase, EC# 2.1.1.222   2-OCTAPRENYL-6-OHPHENOL-METHY-RXN 
  

 

 
 Hit 
 

 
 Common 
 

 
 P 
 

 
 #Q 
 

 
 Best Qry 
 

 
 Best Eval 
 

 
 Avg Rank 
 

 
 Aln Len 
 

 
 Qry Len 
 

 
 IDOP? 
 

 
 adj? 
 

 

 

 
GKFG-177-MONOMER
 

 
ubiE; methylase; K03183 u
 

 
0.36183572
 

 
1.0
 

 
P17993
 

 
2.2385e-7
 

 
1.0
 

 
0.9125
 

 
240
 

 
T
 

 
NIL
 

 

 

 
GKFG-476-MONOMER
 

 
SAM-dependent methyltrans
 

 
1.5675386e-4
 

 
1.0
 

 
P17993
 

 
6.20499e-6
 

 
2.0
 

 
0.425
 

 
240
 

 
NIL
 

 
NIL
 

 

 

 
GKFG-1443-MONOMER
 

 
methyltransferase small (
 

 
2.9708795e-5
 

 
1.0
 

 
P17993
 

 
0.0934736
 

 
3.0
 

 
0.29166666
 

 
240
 

 
NIL
 

 
NIL
 

 

 
     Evidence for , EC# 1.14.13.-   2-OCTAPRENYLPHENOL-HYDROX-RXN 
  
no BLAST hits found
 
     Evidence for , EC# 4.1.1.-   3-OCTAPRENYL-4-OHBENZOATE-DECARBOX-RXN 
  

 

 
 Hit 
 

 
 Common 
 

 
 P 
 

 
 #Q 
 

 
 Best Qry 
 

 
 Best Eval 
 

 
 Avg Rank 
 

 
 Aln Len 
 

 
 Qry Len 
 

 
 IDOP? 
 

 
 adj? 
 

 

 

 
GKFG-87-MONOMER
 

 
173553..173783 - ( gc_con
 

 
3.182795e-4
 

 
1.0
 

 
P0AG03
 

 
0.0225876
 

 
1.0
 

 
0.3968254
 

 
189
 

 
T
 

 
NIL
 

 

 
     Evidence for , EC# 2.5.1.39   4OHBENZOATE-OCTAPRENYLTRANSFER-RXN 
  
no BLAST hits found
 
     

  tetrapyrrole biosynthesis I     Total # of reactions in pathway = 6   Present reactions: 5
  

 

 
 Reaction 
 

 
 Protein(s) 
 

 

 

 
OHMETHYLBILANESYN-RXN
 

 
(# )
 

 

 

 
PORPHOBILSYNTH-RXN
 

 
(# )
 

 

 

 
GSAAMINOTRANS-RXN
 

 
(# 
 # )
 

 

 

 
GLUTRNAREDUCT-RXN
 

 
(# 
 # )
 

 

 

 
GLURS-RXN
 

 
(# 
 # )
 

 

 
   Missing reactions: 1
  

 

 
 UROGENIIISYN-RXN 
 

 

 
        Evidence for uroporphyrinogen-III synthase, EC# 4.2.1.75   UROGENIIISYN-RXN 
  

 

 
 Hit 
 

 
 Common 
 

 
 P 
 

 
 #Q 
 

 
 Best Qry 
 

 
 Best Eval 
 

 
 Avg Rank 
 

 
 Aln Len 
 

 
 Qry Len 
 

 
 IDOP? 
 

 
 adj? 
 

 

 

 
GKFG-1549-MONOMER
 

 
mcu:HMPREF0573_10017 tyrS
 

 
0.0018147272
 

 
1.0
 

 
Q59683
 

 
0.470649
 

 
1.0
 

 
0.50753766
 

 
199
 

 
T
 

 
NIL
 

 

 

 
GKFG-1998-MONOMER
 

 
mcu:HMPREF0573_10570 DNA-
 

 
3.182795e-4
 

 
1.0
 

 
Q8KCJ3
 

 
0.54541
 

 
1.0
 

 
0.37398374
 

 
246
 

 
T
 

 
NIL
 

 

 

 
GKFG-1745-MONOMER
 

 
winged helix family two c
 

 
3.182795e-4
 

 
1.0
 

 
P42452
 

 
0.242274
 

 
1.0
 

 
0.40151516
 

 
264
 

 
T
 

 
NIL
 

 

 

 
GKFG-1049-MONOMER
 

 
putative methyltransferas
 

 
3.182795e-4
 

 
1.0
 

 
Q59294
 

 
0.0407459
 

 
1.0
 

 
0.46031746
 

 
504
 

 
T
 

 
NIL
 

 

 

 
GKFG-1590-MONOMER
 

 
lipoate-protein ligase B 
 

 
1.5421621e-5
 

 
1.0
 

 
P21248
 

 
0.125903
 

 
1.0
 

 
0.15648855
 

 
262
 

 
T
 

 
NIL
 

 

 

 
GKFG-441-MONOMER
 

 
bcv:Bcav_3074 carbohydrat
 

 
2.795795e-6
 

 
1.0
 

 
P42452
 

 
0.95341
 

 
2.0
 

 
0.22348484
 

 
264
 

 
T
 

 
NIL
 

 

 
     

  flavin biosynthesis I (bacteria and plants)     Total # of reactions in pathway = 9   Present reactions: 8
  

 

 
 Reaction 
 

 
 Protein(s) 
 

 

 

 
RIBOFLAVINKIN-RXN
 

 
(# )
 

 

 

 
FADSYN-RXN
 

 
(# )
 

 

 

 
RIBOFLAVIN-SYN-RXN
 

 
(# 
 # )
 

 

 

 
LUMAZINESYN-RXN
 

 
(# )
 

 

 

 
DIOHBUTANONEPSYN-RXN
 

 
(# )
 

 

 

 
RIBOFLAVINSYNREDUC-RXN
 

 
(# )
 

 

 

 
RIBOFLAVINSYNDEAM-RXN
 

 
(# )
 

 

 

 
GTP-CYCLOHYDRO-II-RXN
 

 
(# )
 

 

 
   Missing reactions: 1
  

 

 
 RIBOPHOSPHAT-RXN 
 

 

 
        Evidence for    RIBOPHOSPHAT-RXN      

  formylTHF biosynthesis I     Total # of reactions in pathway = 11   Present reactions: 7
  

 

 
 Reaction 
 

 
 Protein(s) 
 

 

 

 
METHENYLTHFCYCLOHYDRO-RXN
 

 
(# )
 

 

 

 
FORMYLTHFDEFORMYL-RXN
 

 
(# )
 

 

 

 
DIHYDROFOLATEREDUCT-RXN
 

 
(# )
 

 

 

 
GLYOHMETRANS-RXN
 

 
(# )
 

 

 

 
METHYLENETHFDEHYDROG-NADP-RXN
 

 
(# )
 

 

 

 
THYMIDYLATESYN-RXN
 

 
(# )
 

 

 

 
DIHYDROFOLATESYNTH-RXN
 

 
(# )
 

 

 
   Missing reactions: 4
  

 

 
 FORMATETHFLIG-RXN 
 

 

 

 
 GCVMULTI-RXN 
 

 

 

 
 1.5.1.20-RXN 
 

 

 

 
 HOMOCYSMETB12-RXN 
 

 

 
        Evidence for formate&mdash;tetrahydrofolate ligase, EC# 6.3.4.3   FORMATETHFLIG-RXN 
  

 

 
 Hit 
 

 
 Common 
 

 
 P 
 

 
 #Q 
 

 
 Best Qry 
 

 
 Best Eval 
 

 
 Avg Rank 
 

 
 Aln Len 
 

 
 Qry Len 
 

 
 IDOP? 
 

 
 adj? 
 

 

 

 
GKFG-718-MONOMER
 

 
mta:Moth_1259 propionate 
 

 
1.5421621e-5
 

 
1.0
 

 
A9L3Z6
 

 
0.461455
 

 
1.0
 

 
0.15897436
 

 
585
 

 
T
 

 
NIL
 

 

 

 
GKFG-1648-MONOMER
 

 
str:Sterm_2106 phosphorib
 

 
1.5421621e-5
 

 
1.0
 

 
Q8EUQ1
 

 
0.161018
 

 
1.0
 

 
0.15660377
 

 
530
 

 
T
 

 
NIL
 

 

 

 
GKFG-1338-MONOMER
 

 
putative ribosomal-protei
 

 
1.5421621e-5
 

 
1.0
 

 
Q2LU82
 

 
0.677867
 

 
1.0
 

 
0.139823
 

 
565
 

 
T
 

 
NIL
 

 

 

 
GKFG-1950-MONOMER
 

 
bcv:Bcav_2215 pyruvate ki
 

 
1.5421621e-5
 

 
1.0
 

 
A4SPE5
 

 
0.837003
 

 
1.0
 

 
0.19827586
 

 
580
 

 
T
 

 
NIL
 

 

 

 
GKFG-751-MONOMER
 

 
ComEC/Rec2-like protein; 
 

 
1.5421621e-5
 

 
1.0
 

 
A5G276
 

 
0.477171
 

 
1.0
 

 
0.12926391
 

 
557
 

 
T
 

 
NIL
 

 

 

 
GKFG-864-MONOMER
 

 
crd:CRES_0981 pip; prolin
 

 
1.3945618e-5
 

 
1.0
 

 
Q72GY9
 

 
0.532631
 

 
1.0
 

 
0.06998158
 

 
543
 

 
T
 

 
NIL
 

 

 

 
GKFG-1603-MONOMER
 

 
xce:Xcel_0763 hypothetica
 

 
1.3945618e-5
 

 
1.0
 

 
A6L4P0
 

 
0.140098
 

 
1.0
 

 
0.06666667
 

 
555
 

 
T
 

 
NIL
 

 

 

 
GKFG-1196-MONOMER
 

 
rha:RHA1_ro05116 sugar AB
 

 
1.3945618e-5
 

 
1.0
 

 
Q18JB5
 

 
0.751631
 

 
1.0
 

 
0.08448276
 

 
580
 

 
T
 

 
NIL
 

 

 

 
GKFG-900-MONOMER
 

 
ahe:Arch_1354 electron-tr
 

 
1.3945618e-5
 

 
1.0
 

 
O96553
 

 
0.105535
 

 
1.0
 

 
0.045454547
 

 
968
 

 
T
 

 
NIL
 

 

 

 
GKFG-701-MONOMER
 

 
ach:Achl_1330 fibronectin
 

 
1.3945618e-5
 

 
1.0
 

 
P09440
 

 
0.107791
 

 
1.0
 

 
0.055384614
 

 
975
 

 
T
 

 
NIL
 

 

 

 
GKFG-434-MONOMER
 

 
ahe:Arch_1331 succinate d
 

 
7.594039e-6
 

 
1.0
 

 
Q2LU82
 

 
0.71449
 

 
2.0
 

 
0.2300885
 

 
565
 

 
NIL
 

 
NIL
 

 

 

 
GKFG-1837-MONOMER
 

 
bcv:Bcav_2156 Cobyrinic a
 

 
2.795795e-6
 

 
5.0
 

 
A0JZ10
 

 
0.00114204
 

 
1.4
 

 
0.12315416
 

 
565
 

 
T
 

 
NIL
 

 

 

 
GKFG-998-MONOMER
 

 
class II aldolase/adducin
 

 
2.795795e-6
 

 
2.0
 

 
A4J0S6
 

 
0.0519834
 

 
1.5
 

 
0.12180573
 

 
567
 

 
T
 

 
NIL
 

 

 

 
GKFG-1993-MONOMER
 

 
xylulokinase; K00854 xylu
 

 
2.5282066e-6
 

 
1.0
 

 
O96553
 

 
0.707519
 

 
2.0
 

 
0.044421487
 

 
968
 

 
T
 

 
NIL
 

 

 

 
GKFG-651-MONOMER
 

 
mcu:HMPREF0573_11422 proS
 

 
1.0926308e-6
 

 
1.0
 

 
C5CI89
 

 
0.904584
 

 
3.0
 

 
0.11510792
 

 
556
 

 
T
 

 
NIL
 

 

 
     Evidence for    GCVMULTI-RXN 
  

 

 
 Hit 
 

 
 Common 
 

 
 P 
 

 
 #Q 
 

 
 Best Qry 
 

 
 Best Eval 
 

 
 Avg Rank 
 

 
 Aln Len 
 

 
 Qry Len 
 

 
 IDOP? 
 

 
 adj? 
 

 

 

 
GKFG-669-MONOMER
 

 
ahe:Arch_1322 alkyl hydro
 

 
3.182795e-4
 

 
1.0
 

 
P0A9P0
 

 
3.51896e-10
 

 
1.0
 

 
0.36919832
 

 
474
 

 
T
 

 
NIL
 

 

 

 
GKFG-900-MONOMER
 

 
ahe:Arch_1354 electron-tr
 

 
2.4944004e-5
 

 
1.0
 

 
P0A9P0
 

 
0.310782
 

 
3.0
 

 
0.37130803
 

 
474
 

 
T
 

 
NIL
 

 

 

 
GKFG-549-MONOMER
 

 
iva:Isova_3010 thioredoxi
 

 
6.867209e-6
 

 
1.0
 

 
P0A9P0
 

 
0.0052998
 

 
2.0
 

 
0.08438819
 

 
474
 

 
NIL
 

 
NIL
 

 

 

 
GKFG-1950-MONOMER
 

 
bcv:Bcav_2215 pyruvate ki
 

 
4.3925725e-6
 

 
1.0
 

 
P0A9P0
 

 
0.768297
 

 
5.0
 

 
0.1392405
 

 
474
 

 
T
 

 
NIL
 

 

 

 
GKFG-1661-MONOMER
 

 
aau:AAur_3325 gdhA; gluta
 

 
1.6156636e-6
 

 
1.0
 

 
P0A9P0
 

 
0.366188
 

 
4.0
 

 
0.12236287
 

 
474
 

 
T
 

 
NIL
 

 

 
     Evidence for methylenetetrahydrofolate reductase [NAD(P)H], EC# 1.5.1.20   1.5.1.20-RXN 
  

 

 
 Hit 
 

 
 Common 
 

 
 P 
 

 
 #Q 
 

 
 Best Qry 
 

 
 Best Eval 
 

 
 Avg Rank 
 

 
 Aln Len 
 

 
 Qry Len 
 

 
 IDOP? 
 

 
 adj? 
 

 

 

 
GKFG-1734-MONOMER
 

 
pad:TIIST44_04425 HAD hyd
 

 
1.3958463e-4
 

 
1.0
 

 
O67422
 

 
0.391231
 

 
1.0
 

 
0.2972973
 

 
296
 

 
T
 

 
NIL
 

 

 

 
GKFG-1812-MONOMER
 

 
NUDIX hydrolase (db=KEGG 
 

 
1.3958463e-4
 

 
1.0
 

 
P71319
 

 
0.206618
 

 
1.0
 

 
0.2785235
 

 
298
 

 
T
 

 
NIL
 

 

 

 
GKFG-206-MONOMER
 

 
cef:CE1295 respiratory ni
 

 
3.787886e-5
 

 
1.0
 

 
O06745
 

 
0.0946223
 

 
1.0
 

 
0.10130719
 

 
612
 

 
NIL
 

 
NIL
 

 

 

 
GKFG-617-MONOMER
 

 
cga:Celgi_1403 dihydrooro
 

 
1.3945618e-5
 

 
1.0
 

 
Q17693
 

 
0.0528396
 

 
1.0
 

 
0.09803922
 

 
663
 

 
T
 

 
NIL
 

 

 

 
GKFG-580-MONOMER
 

 
fma:FMG_1392 hypothetical
 

 
1.3945618e-5
 

 
1.0
 

 
P46151
 

 
0.139483
 

 
1.0
 

 
0.07762557
 

 
657
 

 
T
 

 
NIL
 

 

 

 
GKFG-1065-MONOMER
 

 
mcu:HMPREF0573_10379 thrC
 

 
1.3945618e-5
 

 
1.0
 

 
O74927
 

 
0.113862
 

 
1.0
 

 
0.07488299
 

 
641
 

 
T
 

 
NIL
 

 

 

 
GKFG-1813-MONOMER
 

 
membrane protein (db=KEGG
 

 
2.5282066e-6
 

 
1.0
 

 
O06745
 

 
0.408125
 

 
2.0
 

 
0.0375817
 

 
612
 

 
T
 

 
NIL
 

 

 

 
GKFG-972-MONOMER
 

 
LacI family transcription
 

 
2.5282066e-6
 

 
1.0
 

 
O74927
 

 
0.692905
 

 
2.0
 

 
0.101404056
 

 
641
 

 
T
 

 
NIL
 

 

 
     Evidence for methionine synthase, EC# 2.1.1.13   HOMOCYSMETB12-RXN 
  

 

 
 Hit 
 

 
 Common 
 

 
 P 
 

 
 #Q 
 

 
 Best Qry 
 

 
 Best Eval 
 

 
 Avg Rank 
 

 
 Aln Len 
 

 
 Qry Len 
 

 
 IDOP? 
 

 
 adj? 
 

 

 

 
GKFG-1062-MONOMER
 

 
mcu:HMPREF0573_10372 rho;
 

 
1.3945618e-5
 

 
1.0
 

 
Q49775
 

 
0.789321
 

 
1.0
 

 
0.04477612
 

 
1206
 

 
T
 

 
NIL
 

 

 

 
GKFG-1196-MONOMER
 

 
rha:RHA1_ro05116 sugar AB
 

 
1.3945618e-5
 

 
1.0
 

 
P13009
 

 
0.0662266
 

 
1.0
 

 
0.05378973
 

 
1227
 

 
T
 

 
NIL
 

 

 

 
GKFG-1944-MONOMER
 

 
bcv:Bcav_2184 excinucleas
 

 
2.5282066e-6
 

 
1.0
 

 
P13009
 

 
0.333957
 

 
2.0
 

 
0.051344745
 

 
1227
 

 
T
 

 
NIL
 

 

 

 
GKFG-1242-MONOMER
 

 
cfi:Celf_2761 alpha amyla
 

 
1.0926308e-6
 

 
1.0
 

 
P13009
 

 
0.624379
 

 
3.0
 

 
0.072534636
 

 
1227
 

 
T
 

 
NIL
 

 

 
     

  folate transformations I     Total # of reactions in pathway = 12   Present reactions: 6
  

 

 
 Reaction 
 

 
 Protein(s) 
 

 

 

 
METHENYLTHFCYCLOHYDRO-RXN
 

 
(# )
 

 

 

 
FORMYLTHFDEFORMYL-RXN
 

 
(# )
 

 

 

 
GLYOHMETRANS-RXN
 

 
(# )
 

 

 

 
METHYLENETHFDEHYDROG-NADP-RXN
 

 
(# )
 

 

 

 
5-FORMYL-THF-CYCLO-LIGASE-RXN
 

 
(# )
 

 

 

 
RXN-5061
 

 
NIL
 

 

 
   Missing reactions: 6
  

 

 
 FORMATETHFLIG-RXN 
 

 

 

 
 GCVMULTI-RXN 
 

 

 

 
 HOMOCYSMETB12-RXN 
 

 

 

 
 2.1.1.19-RXN 
 

 

 

 
 1.5.1.20-RXN 
 

 

 

 
 1.5.1.15-RXN 
 

 

 
        Evidence for formate&mdash;tetrahydrofolate ligase, EC# 6.3.4.3   FORMATETHFLIG-RXN 
  

 

 
 Hit 
 

 
 Common 
 

 
 P 
 

 
 #Q 
 

 
 Best Qry 
 

 
 Best Eval 
 

 
 Avg Rank 
 

 
 Aln Len 
 

 
 Qry Len 
 

 
 IDOP? 
 

 
 adj? 
 

 

 

 
GKFG-718-MONOMER
 

 
mta:Moth_1259 propionate 
 

 
1.5421621e-5
 

 
1.0
 

 
A9L3Z6
 

 
0.461455
 

 
1.0
 

 
0.15897436
 

 
585
 

 
T
 

 
NIL
 

 

 

 
GKFG-1648-MONOMER
 

 
str:Sterm_2106 phosphorib
 

 
1.5421621e-5
 

 
1.0
 

 
Q8EUQ1
 

 
0.161018
 

 
1.0
 

 
0.15660377
 

 
530
 

 
T
 

 
NIL
 

 

 

 
GKFG-1338-MONOMER
 

 
putative ribosomal-protei
 

 
1.5421621e-5
 

 
1.0
 

 
Q2LU82
 

 
0.677867
 

 
1.0
 

 
0.139823
 

 
565
 

 
T
 

 
NIL
 

 

 

 
GKFG-1950-MONOMER
 

 
bcv:Bcav_2215 pyruvate ki
 

 
1.5421621e-5
 

 
1.0
 

 
A4SPE5
 

 
0.837003
 

 
1.0
 

 
0.19827586
 

 
580
 

 
T
 

 
NIL
 

 

 

 
GKFG-751-MONOMER
 

 
ComEC/Rec2-like protein; 
 

 
1.5421621e-5
 

 
1.0
 

 
A5G276
 

 
0.477171
 

 
1.0
 

 
0.12926391
 

 
557
 

 
T
 

 
NIL
 

 

 

 
GKFG-864-MONOMER
 

 
crd:CRES_0981 pip; prolin
 

 
1.3945618e-5
 

 
1.0
 

 
Q72GY9
 

 
0.532631
 

 
1.0
 

 
0.06998158
 

 
543
 

 
T
 

 
NIL
 

 

 

 
GKFG-1603-MONOMER
 

 
xce:Xcel_0763 hypothetica
 

 
1.3945618e-5
 

 
1.0
 

 
A6L4P0
 

 
0.140098
 

 
1.0
 

 
0.06666667
 

 
555
 

 
T
 

 
NIL
 

 

 

 
GKFG-1196-MONOMER
 

 
rha:RHA1_ro05116 sugar AB
 

 
1.3945618e-5
 

 
1.0
 

 
Q18JB5
 

 
0.751631
 

 
1.0
 

 
0.08448276
 

 
580
 

 
T
 

 
NIL
 

 

 

 
GKFG-900-MONOMER
 

 
ahe:Arch_1354 electron-tr
 

 
1.3945618e-5
 

 
1.0
 

 
O96553
 

 
0.105535
 

 
1.0
 

 
0.045454547
 

 
968
 

 
T
 

 
NIL
 

 

 

 
GKFG-701-MONOMER
 

 
ach:Achl_1330 fibronectin
 

 
1.3945618e-5
 

 
1.0
 

 
P09440
 

 
0.107791
 

 
1.0
 

 
0.055384614
 

 
975
 

 
T
 

 
NIL
 

 

 

 
GKFG-434-MONOMER
 

 
ahe:Arch_1331 succinate d
 

 
7.594039e-6
 

 
1.0
 

 
Q2LU82
 

 
0.71449
 

 
2.0
 

 
0.2300885
 

 
565
 

 
NIL
 

 
NIL
 

 

 

 
GKFG-1837-MONOMER
 

 
bcv:Bcav_2156 Cobyrinic a
 

 
2.795795e-6
 

 
5.0
 

 
A0JZ10
 

 
0.00114204
 

 
1.4
 

 
0.12315416
 

 
565
 

 
T
 

 
NIL
 

 

 

 
GKFG-998-MONOMER
 

 
class II aldolase/adducin
 

 
2.795795e-6
 

 
2.0
 

 
A4J0S6
 

 
0.0519834
 

 
1.5
 

 
0.12180573
 

 
567
 

 
T
 

 
NIL
 

 

 

 
GKFG-1993-MONOMER
 

 
xylulokinase; K00854 xylu
 

 
2.5282066e-6
 

 
1.0
 

 
O96553
 

 
0.707519
 

 
2.0
 

 
0.044421487
 

 
968
 

 
T
 

 
NIL
 

 

 

 
GKFG-651-MONOMER
 

 
mcu:HMPREF0573_11422 proS
 

 
1.0926308e-6
 

 
1.0
 

 
C5CI89
 

 
0.904584
 

 
3.0
 

 
0.11510792
 

 
556
 

 
T
 

 
NIL
 

 

 
     Evidence for    GCVMULTI-RXN 
  

 

 
 Hit 
 

 
 Common 
 

 
 P 
 

 
 #Q 
 

 
 Best Qry 
 

 
 Best Eval 
 

 
 Avg Rank 
 

 
 Aln Len 
 

 
 Qry Len 
 

 
 IDOP? 
 

 
 adj? 
 

 

 

 
GKFG-669-MONOMER
 

 
ahe:Arch_1322 alkyl hydro
 

 
3.182795e-4
 

 
1.0
 

 
P0A9P0
 

 
3.51896e-10
 

 
1.0
 

 
0.36919832
 

 
474
 

 
T
 

 
NIL
 

 

 

 
GKFG-900-MONOMER
 

 
ahe:Arch_1354 electron-tr
 

 
2.4944004e-5
 

 
1.0
 

 
P0A9P0
 

 
0.310782
 

 
3.0
 

 
0.37130803
 

 
474
 

 
T
 

 
NIL
 

 

 

 
GKFG-549-MONOMER
 

 
iva:Isova_3010 thioredoxi
 

 
6.867209e-6
 

 
1.0
 

 
P0A9P0
 

 
0.0052998
 

 
2.0
 

 
0.08438819
 

 
474
 

 
NIL
 

 
NIL
 

 

 

 
GKFG-1950-MONOMER
 

 
bcv:Bcav_2215 pyruvate ki
 

 
4.3925725e-6
 

 
1.0
 

 
P0A9P0
 

 
0.768297
 

 
5.0
 

 
0.1392405
 

 
474
 

 
T
 

 
NIL
 

 

 

 
GKFG-1661-MONOMER
 

 
aau:AAur_3325 gdhA; gluta
 

 
1.6156636e-6
 

 
1.0
 

 
P0A9P0
 

 
0.366188
 

 
4.0
 

 
0.12236287
 

 
474
 

 
T
 

 
NIL
 

 

 
     Evidence for methionine synthase, EC# 2.1.1.13   HOMOCYSMETB12-RXN 
  

 

 
 Hit 
 

 
 Common 
 

 
 P 
 

 
 #Q 
 

 
 Best Qry 
 

 
 Best Eval 
 

 
 Avg Rank 
 

 
 Aln Len 
 

 
 Qry Len 
 

 
 IDOP? 
 

 
 adj? 
 

 

 

 
GKFG-1062-MONOMER
 

 
mcu:HMPREF0573_10372 rho;
 

 
1.3945618e-5
 

 
1.0
 

 
Q49775
 

 
0.789321
 

 
1.0
 

 
0.04477612
 

 
1206
 

 
T
 

 
NIL
 

 

 

 
GKFG-1196-MONOMER
 

 
rha:RHA1_ro05116 sugar AB
 

 
1.3945618e-5
 

 
1.0
 

 
P13009
 

 
0.0662266
 

 
1.0
 

 
0.05378973
 

 
1227
 

 
T
 

 
NIL
 

 

 

 
GKFG-1944-MONOMER
 

 
bcv:Bcav_2184 excinucleas
 

 
2.5282066e-6
 

 
1.0
 

 
P13009
 

 
0.333957
 

 
2.0
 

 
0.051344745
 

 
1227
 

 
T
 

 
NIL
 

 

 

 
GKFG-1242-MONOMER
 

 
cfi:Celf_2761 alpha amyla
 

 
1.0926308e-6
 

 
1.0
 

 
P13009
 

 
0.624379
 

 
3.0
 

 
0.072534636
 

 
1227
 

 
T
 

 
NIL
 

 

 
     Evidence for trimethylsulfonium&mdash;tetrahydrofolate  N -methyltransferase, EC# 2.1.1.19   2.1.1.19-RXN      Evidence for methylenetetrahydrofolate reductase [NAD(P)H], EC# 1.5.1.20   1.5.1.20-RXN 
  

 

 
 Hit 
 

 
 Common 
 

 
 P 
 

 
 #Q 
 

 
 Best Qry 
 

 
 Best Eval 
 

 
 Avg Rank 
 

 
 Aln Len 
 

 
 Qry Len 
 

 
 IDOP? 
 

 
 adj? 
 

 

 

 
GKFG-1734-MONOMER
 

 
pad:TIIST44_04425 HAD hyd
 

 
1.3958463e-4
 

 
1.0
 

 
O67422
 

 
0.391231
 

 
1.0
 

 
0.2972973
 

 
296
 

 
T
 

 
NIL
 

 

 

 
GKFG-1812-MONOMER
 

 
NUDIX hydrolase (db=KEGG 
 

 
1.3958463e-4
 

 
1.0
 

 
P71319
 

 
0.206618
 

 
1.0
 

 
0.2785235
 

 
298
 

 
T
 

 
NIL
 

 

 

 
GKFG-206-MONOMER
 

 
cef:CE1295 respiratory ni
 

 
3.787886e-5
 

 
1.0
 

 
O06745
 

 
0.0946223
 

 
1.0
 

 
0.10130719
 

 
612
 

 
NIL
 

 
NIL
 

 

 

 
GKFG-617-MONOMER
 

 
cga:Celgi_1403 dihydrooro
 

 
1.3945618e-5
 

 
1.0
 

 
Q17693
 

 
0.0528396
 

 
1.0
 

 
0.09803922
 

 
663
 

 
T
 

 
NIL
 

 

 

 
GKFG-580-MONOMER
 

 
fma:FMG_1392 hypothetical
 

 
1.3945618e-5
 

 
1.0
 

 
P46151
 

 
0.139483
 

 
1.0
 

 
0.07762557
 

 
657
 

 
T
 

 
NIL
 

 

 

 
GKFG-1065-MONOMER
 

 
mcu:HMPREF0573_10379 thrC
 

 
1.3945618e-5
 

 
1.0
 

 
O74927
 

 
0.113862
 

 
1.0
 

 
0.07488299
 

 
641
 

 
T
 

 
NIL
 

 

 

 
GKFG-1813-MONOMER
 

 
membrane protein (db=KEGG
 

 
2.5282066e-6
 

 
1.0
 

 
O06745
 

 
0.408125
 

 
2.0
 

 
0.0375817
 

 
612
 

 
T
 

 
NIL
 

 

 

 
GKFG-972-MONOMER
 

 
LacI family transcription
 

 
2.5282066e-6
 

 
1.0
 

 
O74927
 

 
0.692905
 

 
2.0
 

 
0.101404056
 

 
641
 

 
T
 

 
NIL
 

 

 
     Evidence for methylenetetrahydrofolate dehydrogenase (NAD  +  ), EC# 1.5.1.15   1.5.1.15-RXN 
  

 

 
 Hit 
 

 
 Common 
 

 
 P 
 

 
 #Q 
 

 
 Best Qry 
 

 
 Best Eval 
 

 
 Avg Rank 
 

 
 Aln Len 
 

 
 Qry Len 
 

 
 IDOP? 
 

 
 adj? 
 

 

 

 
GKFG-720-MONOMER
 

 
fabG; 3-ketoacyl-ACP redu
 

 
1.5421621e-5
 

 
2.0
 

 
Q86KU6
 

 
0.00651359
 

 
1.0
 

 
0.16963358
 

 
313
 

 
T
 

 
NIL
 

 

 

 
GKFG-715-MONOMER
 

 
mcu:HMPREF0573_11674 glxK
 

 
1.5421621e-5
 

 
1.0
 

 
P18155
 

 
0.100218
 

 
1.0
 

 
0.14
 

 
350
 

 
T
 

 
NIL
 

 

 

 
GKFG-391-MONOMER
 

 
Inorganic diphosphatase; 
 

 
6.867209e-6
 

 
1.0
 

 
Q86KU6
 

 
0.407512
 

 
2.0
 

 
0.10223642
 

 
313
 

 
NIL
 

 
NIL
 

 

 
     

  6-hydroxymethyl-dihydropterin diphosphate biosynthesis I     Total # of reactions in pathway = 5   Present reactions: 2
  

 

 
 Reaction 
 

 
 Protein(s) 
 

 

 

 
H2PTERIDINEPYROPHOSPHOKIN-RXN
 

 
(# 
 # )
 

 

 

 
GTP-CYCLOHYDRO-I-RXN
 

 
(# 
 # )
 

 

 
   Missing reactions: 3
  

 

 
 H2NEOPTERINALDOL-RXN 
 

 

 

 
 DIHYDRONEOPTERIN-MONO-P-DEPHOS-RXN 
 

 

 

 
 H2NEOPTERINP3PYROPHOSPHOHYDRO-RXN 
 

 

 
        Evidence for dihydroneopterin aldolase, EC# 4.1.2.25   H2NEOPTERINALDOL-RXN 
  

 

 
 Hit 
 

 
 Common 
 

 
 P 
 

 
 #Q 
 

 
 Best Qry 
 

 
 Best Eval 
 

 
 Avg Rank 
 

 
 Aln Len 
 

 
 Qry Len 
 

 
 IDOP? 
 

 
 adj? 
 

 

 

 
GKFG-521-MONOMER
 

 
UniRef90_E6KT22 Stage 0 D
 

 
0.1008187
 

 
1.0
 

 
Q9Z7E9
 

 
0.649884
 

 
1.0
 

 
0.880597
 

 
134
 

 
NIL
 

 
NIL
 

 

 

 
GKFG-623-MONOMER
 

 
seg (db=Seg db_id=seg fro
 

 
3.787886e-5
 

 
1.0
 

 
Q54YD9
 

 
0.683788
 

 
1.0
 

 
0.07001522
 

 
657
 

 
NIL
 

 
NIL
 

 

 

 
GKFG-1566-MONOMER
 

 
hypothetical protein; K06
 

 
3.787886e-5
 

 
1.0
 

 
P29251
 

 
0.959351
 

 
1.0
 

 
0.10135135
 

 
740
 

 
NIL
 

 
NIL
 

 

 
     Evidence for , EC# 3.6.1.-   DIHYDRONEOPTERIN-MONO-P-DEPHOS-RXN      Evidence for , EC# 3.6.1.-   H2NEOPTERINP3PYROPHOSPHOHYDRO-RXN 
  

 

 
 Hit 
 

 
 Common 
 

 
 P 
 

 
 #Q 
 

 
 Best Qry 
 

 
 Best Eval 
 

 
 Avg Rank 
 

 
 Aln Len 
 

 
 Qry Len 
 

 
 IDOP? 
 

 
 adj? 
 

 

 

 
GKFG-553-MONOMER
 

 
NUDIX hydrolase (db=KEGG 
 

 
6.873975e-5
 

 
2.0
 

 
Q9CGE1
 

 
0.00221168
 

 
1.5
 

 
0.29333335
 

 
165
 

 
NIL
 

 
NIL
 

 

 

 
GKFG-1812-MONOMER
 

 
NUDIX hydrolase (db=KEGG 
 

 
6.873975e-5
 

 
2.0
 

 
P0AFC0
 

 
1.50565e-6
 

 
1.5
 

 
0.3
 

 
150
 

 
NIL
 

 
NIL
 

 

 

 
GKFG-1311-MONOMER
 

 
UniRef90_E6KRL9 Deoxyribo
 

 
2.9708795e-5
 

 
1.0
 

 
P0AFC0
 

 
0.223707
 

 
3.0
 

 
0.28666666
 

 
150
 

 
NIL
 

 
NIL
 

 

 
     

  phosphopantothenate biosynthesis I     Total # of reactions in pathway = 4   Present reactions: 2
  

 

 
 Reaction 
 

 
 Protein(s) 
 

 

 

 
PANTOATE-BETA-ALANINE-LIG-RXN
 

 
(# )
 

 

 

 
PANTOTHENATE-KIN-RXN
 

 
(# )
 

 

 
   Missing reactions: 2
  

 

 
 2-DEHYDROPANTOATE-REDUCT-RXN 
 

 

 

 
 3-CH3-2-OXOBUTANOATE-OH-CH3-XFER-RXN 
 

 

 
        Evidence for 2-dehydropantoate 2-reductase, EC# 1.1.1.169   2-DEHYDROPANTOATE-REDUCT-RXN 
  

 

 
 Hit 
 

 
 Common 
 

 
 P 
 

 
 #Q 
 

 
 Best Qry 
 

 
 Best Eval 
 

 
 Avg Rank 
 

 
 Aln Len 
 

 
 Qry Len 
 

 
 IDOP? 
 

 
 adj? 
 

 

 

 
GKFG-961-MONOMER
 

 
2-succinyl-5-enolpyruvyl-
 

 
1.3958463e-4
 

 
1.0
 

 
P38787
 

 
0.408509
 

 
1.0
 

 
0.26121372
 

 
379
 

 
T
 

 
NIL
 

 

 

 
GKFG-633-MONOMER
 

 
bcv:Bcav_2436 GTP-binding
 

 
2.5307965e-5
 

 
1.0
 

 
Q5XCQ0
 

 
0.863934
 

 
2.0
 

 
0.30944625
 

 
307
 

 
T
 

 
NIL
 

 

 

 
GKFG-343-MONOMER
 

 
ABC transporter; K02003 p
 

 
2.5307965e-5
 

 
1.0
 

 
Q50648
 

 
0.628473
 

 
2.0
 

 
0.2881356
 

 
295
 

 
T
 

 
NIL
 

 

 

 
GKFG-631-MONOMER
 

 
cfi:Celf_1585 L-lactate d
 

 
2.5307965e-5
 

 
1.0
 

 
O34661
 

 
0.457699
 

 
2.0
 

 
0.26510066
 

 
298
 

 
T
 

 
NIL
 

 

 

 
GKFG-1422-MONOMER
 

 
UniRef90_C0VZY6 Methicill
 

 
1.5421621e-5
 

 
1.0
 

 
P37402
 

 
0.840157
 

 
1.0
 

 
0.122112215
 

 
303
 

 
T
 

 
NIL
 

 

 

 
GKFG-1544-MONOMER
 

 
bcv:Bcav_2890 hypothetica
 

 
1.5421621e-5
 

 
1.0
 

 
Q5XCQ0
 

 
0.0738175
 

 
1.0
 

 
0.22149837
 

 
307
 

 
T
 

 
NIL
 

 

 

 
GKFG-1078-MONOMER
 

 
fal:FRAAL3251 nadE; NAD s
 

 
1.5421621e-5
 

 
1.0
 

 
Q9HW09
 

 
0.538094
 

 
1.0
 

 
0.20462047
 

 
303
 

 
T
 

 
NIL
 

 

 

 
GKFG-16-MONOMER
 

 
(db=HMMPfam db_id=PF05738
 

 
1.5421621e-5
 

 
1.0
 

 
Q987N5
 

 
0.102264
 

 
1.0
 

 
0.1595092
 

 
326
 

 
T
 

 
NIL
 

 

 

 
GKFG-900-MONOMER
 

 
ahe:Arch_1354 electron-tr
 

 
1.5421621e-5
 

 
1.0
 

 
O34661
 

 
0.250256
 

 
1.0
 

 
0.13422818
 

 
298
 

 
T
 

 
NIL
 

 

 

 
GKFG-806-MONOMER
 

 
beta-lactamase (db=KEGG e
 

 
1.5421621e-5
 

 
1.0
 

 
Q50648
 

 
0.108513
 

 
1.0
 

 
0.13220339
 

 
295
 

 
T
 

 
NIL
 

 

 

 
GKFG-66-MONOMER
 

 
kfl:Kfla_6989 hypothetica
 

 
1.3945618e-5
 

 
1.0
 

 
O50098
 

 
0.779399
 

 
1.0
 

 
0.08305648
 

 
301
 

 
T
 

 
NIL
 

 

 

 
GKFG-1036-MONOMER
 

 
mav:MAV_4707 groEL; chape
 

 
2.795795e-6
 

 
1.0
 

 
Q9HW09
 

 
0.611226
 

 
2.0
 

 
0.22442244
 

 
303
 

 
T
 

 
NIL
 

 

 

 
GKFG-456-MONOMER
 

 
tRNA pseudouridine syntha
 

 
2.5282066e-6
 

 
1.0
 

 
Q987N5
 

 
0.558693
 

 
2.0
 

 
0.1196319
 

 
326
 

 
T
 

 
NIL
 

 

 

 
GKFG-1088-MONOMER
 

 
hypothetical protein (db=
 

 
1.2082764e-6
 

 
1.0
 

 
Q987N5
 

 
0.569895
 

 
3.0
 

 
0.14723927
 

 
326
 

 
T
 

 
NIL
 

 

 
     Evidence for 3-methyl-2-oxobutanoate hydroxymethyltransferase, EC# 2.1.2.11   3-CH3-2-OXOBUTANOATE-OH-CH3-XFER-RXN 
  

 

 
 Hit 
 

 
 Common 
 

 
 P 
 

 
 #Q 
 

 
 Best Qry 
 

 
 Best Eval 
 

 
 Avg Rank 
 

 
 Aln Len 
 

 
 Qry Len 
 

 
 IDOP? 
 

 
 adj? 
 

 

 

 
GKFG-1734-MONOMER
 

 
pad:TIIST44_04425 HAD hyd
 

 
1.3958463e-4
 

 
1.0
 

 
Q11F82
 

 
0.933829
 

 
1.0
 

 
0.25622776
 

 
281
 

 
T
 

 
NIL
 

 

 

 
GKFG-1429-MONOMER
 

 
mcu:HMPREF0573_10785 hydr
 

 
1.3958463e-4
 

 
1.0
 

 
Q3AS53
 

 
0.822358
 

 
1.0
 

 
0.31407943
 

 
277
 

 
T
 

 
NIL
 

 

 

 
GKFG-672-MONOMER
 

 
putative ribonuclease H (
 

 
1.3958463e-4
 

 
1.0
 

 
Q8XW45
 

 
0.471408
 

 
1.0
 

 
0.35766423
 

 
274
 

 
T
 

 
NIL
 

 

 

 
GKFG-1866-MONOMER
 

 
transcription elongation 
 

 
1.3958463e-4
 

 
1.0
 

 
Q12F40
 

 
0.053729
 

 
1.0
 

 
0.31666666
 

 
300
 

 
T
 

 
NIL
 

 

 

 
GKFG-668-MONOMER
 

 
hypothetical protein; K07
 

 
2.5307965e-5
 

 
1.0
 

 
A9W3L5
 

 
0.98009
 

 
2.0
 

 
0.24087591
 

 
274
 

 
T
 

 
NIL
 

 

 

 
GKFG-991-MONOMER
 

 
mcu:HMPREF0573_11504 hypo
 

 
1.5421621e-5
 

 
2.0
 

 
Q09672
 

 
0.0670498
 

 
1.0
 

 
0.12423392
 

 
267
 

 
T
 

 
NIL
 

 

 

 
GKFG-91-MONOMER
 

 
ahe:Arch_0351 arginyl-tRN
 

 
1.5421621e-5
 

 
1.0
 

 
B1YHQ9
 

 
0.581067
 

 
1.0
 

 
0.18996416
 

 
279
 

 
T
 

 
NIL
 

 

 

 
GKFG-697-MONOMER
 

 
119351..120019 - ( gc_con
 

 
1.5421621e-5
 

 
1.0
 

 
A1WUU5
 

 
0.115711
 

 
1.0
 

 
0.19272727
 

 
275
 

 
T
 

 
NIL
 

 

 

 
GKFG-1247-MONOMER
 

 
hypothetical protein (db=
 

 
1.5421621e-5
 

 
1.0
 

 
A1VBJ4
 

 
0.0542304
 

 
1.0
 

 
0.12052117
 

 
307
 

 
T
 

 
NIL
 

 

 

 
GKFG-982-MONOMER
 

 
hypothetical protein (db=
 

 
1.5421621e-5
 

 
1.0
 

 
Q83GK7
 

 
0.214739
 

 
1.0
 

 
0.1764706
 

 
272
 

 
T
 

 
NIL
 

 

 

 
GKFG-1065-MONOMER
 

 
mcu:HMPREF0573_10379 thrC
 

 
1.5421621e-5
 

 
1.0
 

 
Q0AB69
 

 
0.826771
 

 
1.0
 

 
0.19133574
 

 
277
 

 
T
 

 
NIL
 

 

 

 
GKFG-196-MONOMER
 

 
molybdopterin synthase su
 

 
1.5421621e-5
 

 
1.0
 

 
Q9RR81
 

 
0.279414
 

 
1.0
 

 
0.2112676
 

 
284
 

 
T
 

 
NIL
 

 

 

 
GKFG-1491-MONOMER
 

 
bbv:HMPREF9228_0173 thiaz
 

 
1.5421621e-5
 

 
1.0
 

 
A7ZEL6
 

 
0.00319181
 

 
1.0
 

 
0.18081181
 

 
271
 

 
T
 

 
NIL
 

 

 

 
GKFG-1487-MONOMER
 

 
sro:Sros_3060 beta-N-acet
 

 
1.5421621e-5
 

 
1.0
 

 
Q89TZ6
 

 
0.0362115
 

 
1.0
 

 
0.19325154
 

 
326
 

 
T
 

 
NIL
 

 

 

 
GKFG-340-MONOMER
 

 
UniRef90_C0VYI5 ABC super
 

 
1.3945618e-5
 

 
1.0
 

 
Q9YE97
 

 
0.547153
 

 
1.0
 

 
0.10948905
 

 
274
 

 
T
 

 
NIL
 

 

 

 
GKFG-2012-MONOMER
 

 
mcu:HMPREF0573_11882 ABC 
 

 
1.3945618e-5
 

 
1.0
 

 
A9W3L5
 

 
0.359364
 

 
1.0
 

 
0.11313868
 

 
274
 

 
T
 

 
NIL
 

 

 

 
GKFG-1961-MONOMER
 

 
ske:Sked_20910 tRNA (aden
 

 
1.3945618e-5
 

 
1.0
 

 
O82357
 

 
0.0978591
 

 
1.0
 

 
0.063400574
 

 
347
 

 
T
 

 
NIL
 

 

 

 
GKFG-1486-MONOMER
 

 
bbi:BBIF_1317 alpha-L-fuc
 

 
1.3945618e-5
 

 
1.0
 

 
Q9Y7B6
 

 
0.321758
 

 
1.0
 

 
0.09169055
 

 
349
 

 
T
 

 
NIL
 

 

 

 
GKFG-178-MONOMER
 

 
HAD superfamily hydrolase
 

 
2.795795e-6
 

 
1.0
 

 
A1WUU5
 

 
0.659852
 

 
2.0
 

 
0.16
 

 
275
 

 
T
 

 
NIL
 

 

 
     

  thiamin diphosphate biosynthesis I (E. coli)     Total # of reactions in pathway = 2   Present reactions: 1
  

 

 
 Reaction 
 

 
 Protein(s) 
 

 

 

 
THI-P-KIN-RXN
 

 
(# )
 

 

 
   Missing reactions: 1
  

 

 
 RXN-12611 
 

 

 
        Evidence for thiamin phosphate synthase, EC# 2.5.1.3   RXN-12611 
  
no BLAST hits found
 
     

  thiamin diphosphate biosynthesis II (Bacillus)     Total # of reactions in pathway = 2   Present reactions: 1
  

 

 
 Reaction 
 

 
 Protein(s) 
 

 

 

 
THI-P-KIN-RXN
 

 
(# )
 

 

 
   Missing reactions: 1
  

 

 
 RXN-12610 
 

 

 
        Evidence for thiamine-phosphate diphosphorylase, EC# 2.5.1.3   RXN-12610 
  
no BLAST hits found
 
     

  thiamin salvage I     Total # of reactions in pathway = 2   Present reactions: 1
  

 

 
 Reaction 
 

 
 Protein(s) 
 

 

 

 
THI-P-KIN-RXN
 

 
(# )
 

 

 
   Missing reactions: 1
  

 

 
 THIKIN-RXN 
 

 

 
        Evidence for thiamine kinase, EC# 2.7.1.89   THIKIN-RXN 
  

 

 
 Hit 
 

 
 Common 
 

 
 P 
 

 
 #Q 
 

 
 Best Qry 
 

 
 Best Eval 
 

 
 Avg Rank 
 

 
 Aln Len 
 

 
 Qry Len 
 

 
 IDOP? 
 

 
 adj? 
 

 

 

 
GKFG-1745-MONOMER
 

 
winged helix family two c
 

 
3.7905585e-4
 

 
1.0
 

 
P75948
 

 
0.649498
 

 
1.0
 

 
0.33941606
 

 
274
 

 
NIL
 

 
NIL
 

 

 

 
GKFG-651-MONOMER
 

 
mcu:HMPREF0573_11422 proS
 

 
4.188785e-5
 

 
1.0
 

 
B5FK99
 

 
0.16443
 

 
1.0
 

 
0.12773722
 

 
274
 

 
NIL
 

 
NIL
 

 

 

 
GKFG-1484-MONOMER
 

 
mcu:HMPREF0573_11157 leuS
 

 
7.594039e-6
 

 
1.0
 

 
B5FK99
 

 
0.530903
 

 
2.0
 

 
0.17518248
 

 
274
 

 
NIL
 

 
NIL
 

 

 
     

  pyridoxal 5'-phosphate biosynthesis I     Total # of reactions in pathway = 7   Present reactions: 3
  

 

 
 Reaction 
 

 
 Protein(s) 
 

 

 

 
DXS-RXN
 

 
(# )
 

 

 

 
ERYTHRON4PDEHYDROG-RXN
 

 
(# )
 

 

 

 
PSERTRANSAMPYR-RXN
 

 
(# )
 

 

 
   Missing reactions: 4
  

 

 
 PNPOXI-RXN 
 

 

 

 
 ERYTH4PDEHYDROG-RXN 
 

 

 

 
 PDXJ-RXN 
 

 

 

 
 1.1.1.262-RXN 
 

 

 
        Evidence for pyridoxal 5&prime;-phosphate synthase, EC# 1.4.3.5   PNPOXI-RXN 
  

 

 
 Hit 
 

 
 Common 
 

 
 P 
 

 
 #Q 
 

 
 Best Qry 
 

 
 Best Eval 
 

 
 Avg Rank 
 

 
 Aln Len 
 

 
 Qry Len 
 

 
 IDOP? 
 

 
 adj? 
 

 

 

 
GKFG-1057-MONOMER
 

 
bbi:BBIF_1733 sialidase; 
 

 
0.0051016076
 

 
1.0
 

 
P0AFI7
 

 
0.749268
 

 
1.0
 

 
0.61009175
 

 
218
 

 
T
 

 
NIL
 

 

 

 
GKFG-339-MONOMER
 

 
ABC transporter permease;
 

 
0.004913946
 

 
1.0
 

 
B0SGE9
 

 
0.22063
 

 
1.0
 

 
0.5613208
 

 
212
 

 
NIL
 

 
NIL
 

 

 

 
GKFG-494-MONOMER
 

 
hypothetical protein (db=
 

 
3.7905585e-4
 

 
2.0
 

 
Q1J188
 

 
0.32368
 

 
1.0
 

 
0.25569764
 

 
218
 

 
NIL
 

 
NIL
 

 

 

 
GKFG-240-MONOMER
 

 
hemA; putative glutamyl-t
 

 
3.7905585e-4
 

 
1.0
 

 
Q0A757
 

 
0.32967
 

 
1.0
 

 
0.34183672
 

 
196
 

 
NIL
 

 
NIL
 

 

 

 
GKFG-282-MONOMER
 

 
kfl:Kfla_6664 phosphoenol
 

 
3.7905585e-4
 

 
1.0
 

 
Q73G09
 

 
0.0126561
 

 
1.0
 

 
0.2638889
 

 
216
 

 
NIL
 

 
NIL
 

 

 

 
GKFG-777-MONOMER
 

 
NlpC/P60 family protein (
 

 
3.182795e-4
 

 
1.0
 

 
Q9PDP2
 

 
0.27091
 

 
1.0
 

 
0.39423078
 

 
208
 

 
T
 

 
NIL
 

 

 

 
GKFG-874-MONOMER
 

 
rer:RER_18960 phage porta
 

 
1.3958463e-4
 

 
1.0
 

 
P21159
 

 
0.0265495
 

 
1.0
 

 
0.28181818
 

 
220
 

 
T
 

 
NIL
 

 

 

 
GKFG-99-MONOMER
 

 
mcu:HMPREF0573_10968 rpoB
 

 
4.188785e-5
 

 
1.0
 

 
Q2IHV2
 

 
0.594725
 

 
1.0
 

 
0.14141414
 

 
198
 

 
NIL
 

 
NIL
 

 

 

 
GKFG-29-MONOMER
 

 
std:SPPN_11055 Zinc metal
 

 
4.188785e-5
 

 
1.0
 

 
Q31IR4
 

 
0.486269
 

 
1.0
 

 
0.12037037
 

 
216
 

 
NIL
 

 
NIL
 

 

 

 
GKFG-314-MONOMER
 

 
D-tyrosyl-tRNA(Tyr) deacy
 

 
3.787886e-5
 

 
1.0
 

 
Q6MK45
 

 
0.962053
 

 
1.0
 

 
0.10204082
 

 
196
 

 
NIL
 

 
NIL
 

 

 

 
GKFG-591-MONOMER
 

 
type I phosphodiesterase/
 

 
1.5421621e-5
 

 
1.0
 

 
Q1QWA6
 

 
0.748392
 

 
1.0
 

 
0.13023256
 

 
215
 

 
T
 

 
NIL
 

 

 

 
GKFG-772-MONOMER
 

 
cell surface protein (db=
 

 
1.5421621e-5
 

 
1.0
 

 
A0LR32
 

 
0.226948
 

 
1.0
 

 
0.17857143
 

 
224
 

 
T
 

 
NIL
 

 

 

 
GKFG-975-MONOMER
 

 
ccu:Ccur_10800 pyridoxal 
 

 
1.5421621e-5
 

 
1.0
 

 
Q5WZ02
 

 
0.803822
 

 
1.0
 

 
0.17209302
 

 
215
 

 
T
 

 
NIL
 

 

 

 
GKFG-1627-MONOMER
 

 
cfi:Celf_2457 3-isopropyl
 

 
1.5421621e-5
 

 
1.0
 

 
Q11U72
 

 
0.65124
 

 
1.0
 

 
0.13488372
 

 
215
 

 
T
 

 
NIL
 

 

 

 
GKFG-818-MONOMER
 

 
mcu:HMPREF0573_10271 pfkL
 

 
1.5421621e-5
 

 
1.0
 

 
A0R420
 

 
0.0452024
 

 
1.0
 

 
0.22608696
 

 
230
 

 
T
 

 
NIL
 

 

 

 
GKFG-1988-MONOMER
 

 
cga:Celgi_1384 (Glutamate
 

 
1.5421621e-5
 

 
1.0
 

 
A1TQY3
 

 
0.671527
 

 
1.0
 

 
0.14222223
 

 
225
 

 
T
 

 
NIL
 

 

 

 
GKFG-1515-MONOMER
 

 
seg (db=Seg db_id=seg fro
 

 
1.3945618e-5
 

 
1.0
 

 
A5FWR6
 

 
0.557661
 

 
1.0
 

 
0.108490564
 

 
212
 

 
T
 

 
NIL
 

 

 

 
GKFG-1356-MONOMER
 

 
acyltransferase (db=KEGG 
 

 
1.3945618e-5
 

 
1.0
 

 
Q5Z236
 

 
0.221726
 

 
1.0
 

 
0.08444444
 

 
225
 

 
T
 

 
NIL
 

 

 

 
GKFG-151-MONOMER
 

 
cga:Celgi_0340 FAD linked
 

 
7.594039e-6
 

 
1.0
 

 
A5FWR6
 

 
0.86902
 

 
2.0
 

 
0.1273585
 

 
212
 

 
NIL
 

 
NIL
 

 

 

 
GKFG-1069-MONOMER
 

 
abortive infection protei
 

 
2.795795e-6
 

 
1.0
 

 
Q1J188
 

 
0.523524
 

 
2.0
 

 
0.14678898
 

 
218
 

 
T
 

 
NIL
 

 

 
     Evidence for erythrose-4-phosphate dehydrogenase, EC# 1.2.1.72   ERYTH4PDEHYDROG-RXN 
  

 

 
 Hit 
 

 
 Common 
 

 
 P 
 

 
 #Q 
 

 
 Best Qry 
 

 
 Best Eval 
 

 
 Avg Rank 
 

 
 Aln Len 
 

 
 Qry Len 
 

 
 IDOP? 
 

 
 adj? 
 

 

 

 
GKFG-1633-MONOMER
 

 
mcu:HMPREF0573_10221 pta;
 

 
2.5307965e-5
 

 
1.0
 

 
P0A9B6
 

 
0.545017
 

 
2.0
 

 
0.27433628
 

 
339
 

 
T
 

 
NIL
 

 

 

 
GKFG-1281-MONOMER
 

 
bpb:bpr_I0298 diaminopime
 

 
1.3945618e-5
 

 
3.0
 

 
A1SVN9
 

 
4.20055e-4
 

 
1.0
 

 
0.07693078
 

 
371
 

 
T
 

 
NIL
 

 

 
     Evidence for pyridoxine 5&prime;-phosphate synthase, EC# 2.6.99.2   PDXJ-RXN 
  

 

 
 Hit 
 

 
 Common 
 

 
 P 
 

 
 #Q 
 

 
 Best Qry 
 

 
 Best Eval 
 

 
 Avg Rank 
 

 
 Aln Len 
 

 
 Qry Len 
 

 
 IDOP? 
 

 
 adj? 
 

 

 

 
GKFG-233-MONOMER
 

 
ahe:Arch_1659 cysteinyl-t
 

 
3.7905585e-4
 

 
1.0
 

 
Q8D304
 

 
0.301282
 

 
1.0
 

 
0.25306123
 

 
245
 

 
NIL
 

 
NIL
 

 

 

 
GKFG-1242-MONOMER
 

 
cfi:Celf_2761 alpha amyla
 

 
1.3958463e-4
 

 
1.0
 

 
B3ELU3
 

 
0.31419
 

 
1.0
 

 
0.2857143
 

 
238
 

 
T
 

 
NIL
 

 

 

 
GKFG-1810-MONOMER
 

 
ruvC; crossover junction 
 

 
1.3958463e-4
 

 
1.0
 

 
B1ZNA6
 

 
0.879215
 

 
1.0
 

 
0.3548387
 

 
248
 

 
T
 

 
NIL
 

 

 

 
GKFG-1057-MONOMER
 

 
bbi:BBIF_1733 sialidase; 
 

 
1.3958463e-4
 

 
1.0
 

 
Q11PM4
 

 
0.0879337
 

 
1.0
 

 
0.26050422
 

 
238
 

 
T
 

 
NIL
 

 

 

 
GKFG-526-MONOMER
 

 
YidC/Oxa1 family membrane
 

 
4.188785e-5
 

 
1.0
 

 
B0SBH6
 

 
0.463919
 

 
1.0
 

 
0.14615385
 

 
260
 

 
NIL
 

 
NIL
 

 

 

 
GKFG-119-MONOMER
 

 
FHA domain-containing pro
 

 
3.787886e-5
 

 
1.0
 

 
Q4FUV3
 

 
0.660926
 

 
1.0
 

 
0.09469697
 

 
264
 

 
NIL
 

 
NIL
 

 

 

 
GKFG-1497-MONOMER
 

 
regulatory protein GntR H
 

 
1.5421621e-5
 

 
1.0
 

 
Q17ZN1
 

 
0.905903
 

 
1.0
 

 
0.17175573
 

 
262
 

 
T
 

 
NIL
 

 

 

 
GKFG-1239-MONOMER
 

 
(db=HMMPfam db_id=PF05738
 

 
1.5421621e-5
 

 
1.0
 

 
Q8UDU5
 

 
0.788975
 

 
1.0
 

 
0.23505977
 

 
251
 

 
T
 

 
NIL
 

 

 

 
GKFG-847-MONOMER
 

 
ATP synthase subunit b; K
 

 
1.5421621e-5
 

 
1.0
 

 
B2U984
 

 
0.978061
 

 
1.0
 

 
0.2243346
 

 
263
 

 
T
 

 
NIL
 

 

 

 
GKFG-1772-MONOMER
 

 
nca:Noca_1107 HsdR family
 

 
2.795795e-6
 

 
1.0
 

 
Q4FUV3
 

 
0.715152
 

 
2.0
 

 
0.18560606
 

 
264
 

 
T
 

 
NIL
 

 

 
     Evidence for 4-hydroxythreonine-4-phosphate dehydrogenase, EC# 1.1.1.262   1.1.1.262-RXN 
  

 

 
 Hit 
 

 
 Common 
 

 
 P 
 

 
 #Q 
 

 
 Best Qry 
 

 
 Best Eval 
 

 
 Avg Rank 
 

 
 Aln Len 
 

 
 Qry Len 
 

 
 IDOP? 
 

 
 adj? 
 

 

 

 
GKFG-720-MONOMER
 

 
fabG; 3-ketoacyl-ACP redu
 

 
0.0018147272
 

 
1.0
 

 
O67019
 

 
0.0829071
 

 
1.0
 

 
0.503125
 

 
320
 

 
T
 

 
NIL
 

 

 

 
GKFG-317-MONOMER
 

 
ckp:ckrop_1873 nanT; puta
 

 
8.6405425e-4
 

 
1.0
 

 
Q89ZK3
 

 
0.178327
 

 
1.0
 

 
0.36263737
 

 
364
 

 
NIL
 

 
NIL
 

 

 

 
GKFG-16-MONOMER
 

 
(db=HMMPfam db_id=PF05738
 

 
8.6405425e-4
 

 
1.0
 

 
Q9A7N4
 

 
0.281638
 

 
1.0
 

 
0.4174174
 

 
333
 

 
NIL
 

 
NIL
 

 

 

 
GKFG-67-MONOMER
 

 
art:Arth_4214 hypothetica
 

 
3.7905585e-4
 

 
1.0
 

 
Q5FNS3
 

 
0.414237
 

 
1.0
 

 
0.25748503
 

 
334
 

 
NIL
 

 
NIL
 

 

 

 
GKFG-633-MONOMER
 

 
bcv:Bcav_2436 GTP-binding
 

 
1.3958463e-4
 

 
1.0
 

 
A0LIN6
 

 
0.482078
 

 
1.0
 

 
0.31594202
 

 
345
 

 
T
 

 
NIL
 

 

 

 
GKFG-1066-MONOMER
 

 
bcv:Bcav_1281 Homoserine 
 

 
1.3958463e-4
 

 
1.0
 

 
Q3V818
 

 
0.353353
 

 
1.0
 

 
0.24776119
 

 
335
 

 
T
 

 
NIL
 

 

 

 
GKFG-1009-MONOMER
 

 
mcu:HMPREF0573_11482 leuB
 

 
7.610347e-5
 

 
16.0
 

 
B2V9E6
 

 
0.00169389
 

 
1.1875
 

 
0.105642416
 

 
322
 

 
T
 

 
NIL
 

 

 

 
GKFG-91-MONOMER
 

 
ahe:Arch_0351 arginyl-tRN
 

 
6.873975e-5
 

 
2.0
 

 
A1K9J6
 

 
0.0754686
 

 
1.5
 

 
0.35606062
 

 
330
 

 
NIL
 

 
NIL
 

 

 

 
GKFG-705-MONOMER
 

 
car:cauri_0599 hypothetic
 

 
5.771539e-5
 

 
1.0
 

 
B2IDU5
 

 
0.244417
 

 
2.0
 

 
0.40356082
 

 
337
 

 
T
 

 
NIL
 

 

 

 
GKFG-178-MONOMER
 

 
HAD superfamily hydrolase
 

 
4.188785e-5
 

 
1.0
 

 
Q89MT9
 

 
0.465366
 

 
1.0
 

 
0.1594203
 

 
345
 

 
NIL
 

 
NIL
 

 

 

 
GKFG-1429-MONOMER
 

 
mcu:HMPREF0573_10785 hydr
 

 
1.5421621e-5
 

 
1.0
 

 
Q475Q2
 

 
0.210349
 

 
1.0
 

 
0.19308357
 

 
347
 

 
T
 

 
NIL
 

 

 

 
GKFG-855-MONOMER
 

 
methylase of polypeptide 
 

 
1.5421621e-5
 

 
1.0
 

 
Q9JX42
 

 
0.271517
 

 
1.0
 

 
0.16413374
 

 
329
 

 
T
 

 
NIL
 

 

 

 
GKFG-735-MONOMER
 

 
hypothetical protein; K07
 

 
1.5421621e-5
 

 
1.0
 

 
B5EL82
 

 
0.36859
 

 
1.0
 

 
0.13813815
 

 
333
 

 
T
 

 
NIL
 

 

 

 
GKFG-1952-MONOMER
 

 
UniRef90_E8JFA8 Indole-3-
 

 
1.5421621e-5
 

 
1.0
 

 
A4XZJ3
 

 
0.411276
 

 
1.0
 

 
0.1454006
 

 
337
 

 
T
 

 
NIL
 

 

 

 
GKFG-281-MONOMER
 

 
bfa:Bfae_19800 copper/sil
 

 
7.594039e-6
 

 
2.0
 

 
A7IJ81
 

 
0.0395419
 

 
2.0
 

 
0.14851566
 

 
350
 

 
NIL
 

 
NIL
 

 

 

 
GKFG-760-MONOMER
 

 
iva:Isova_1293 cell divis
 

 
2.795795e-6
 

 
1.0
 

 
A1B0G5
 

 
0.716234
 

 
2.0
 

 
0.124223605
 

 
322
 

 
T
 

 
NIL
 

 

 

 
GKFG-807-MONOMER
 

 
TetR family transcription
 

 
2.5282066e-6
 

 
1.0
 

 
A7IJ81
 

 
0.506503
 

 
2.0
 

 
0.08857143
 

 
350
 

 
T
 

 
NIL
 

 

 

 
GKFG-1808-MONOMER
 

 
Holliday junction DNA hel
 

 
2.5282066e-6
 

 
1.0
 

 
B1ZAJ4
 

 
0.667249
 

 
2.0
 

 
0.068767905
 

 
349
 

 
T
 

 
NIL
 

 

 
     

  juvenile hormone III biosynthesis I     Total # of reactions in pathway = 6   Present reactions: 1
  

 

 
 Reaction 
 

 
 Protein(s) 
 

 

 

 
RXN-11619
 

 
(# )
 

 

 
   Missing reactions: 5
  

 

 
 RXN-11587 
 

 

 

 
 RXN-11621 
 

 

 

 
 FARNESOL-DEHYDROGENASE-RXN 
 

 

 

 
 RXN-11777 
 

 

 

 
 RXN-11776 
 

 

 
        Evidence for , EC# 2.1.1.-   RXN-11587 
  
no BLAST hits found
 
     Evidence for , EC# 1.14.13.-   RXN-11621 
  

 

 
 Hit 
 

 
 Common 
 

 
 P 
 

 
 #Q 
 

 
 Best Qry 
 

 
 Best Eval 
 

 
 Avg Rank 
 

 
 Aln Len 
 

 
 Qry Len 
 

 
 IDOP? 
 

 
 adj? 
 

 

 

 
GKFG-132-MONOMER
 

 
pfr:PFREUD_19030 iolG1; m
 

 
3.787886e-5
 

 
1.0
 

 
Q6R7M3
 

 
0.441736
 

 
1.0
 

 
0.10150376
 

 
532
 

 
NIL
 

 
NIL
 

 

 
     Evidence for farnesol dehydrogenase, EC# 1.1.1.216   FARNESOL-DEHYDROGENASE-RXN 
  

 

 
 Hit 
 

 
 Common 
 

 
 P 
 

 
 #Q 
 

 
 Best Qry 
 

 
 Best Eval 
 

 
 Avg Rank 
 

 
 Aln Len 
 

 
 Qry Len 
 

 
 IDOP? 
 

 
 adj? 
 

 

 

 
GKFG-720-MONOMER
 

 
fabG; 3-ketoacyl-ACP redu
 

 
0.8249092
 

 
2.0
 

 
D2WKD9
 

 
1.76182e-13
 

 
1.0
 

 
0.94478923
 

 
245
 

 
T
 

 
NIL
 

 

 

 
GKFG-803-MONOMER
 

 
short-chain dehydrogenase
 

 
0.15667598
 

 
2.0
 

 
D2WKD9
 

 
5.92199e-12
 

 
2.0
 

 
0.78527105
 

 
245
 

 
T
 

 
NIL
 

 

 
     Evidence for farnesyl monophosphate phosphatase, EC# 3.1.3.-   RXN-11777 
  

 

 
 Hit 
 

 
 Common 
 

 
 P 
 

 
 #Q 
 

 
 Best Qry 
 

 
 Best Eval 
 

 
 Avg Rank 
 

 
 Aln Len 
 

 
 Qry Len 
 

 
 IDOP? 
 

 
 adj? 
 

 

 

 
GKFG-2023-MONOMER
 

 
mcu:HMPREF0573_11895 hypo
 

 
0.8249092
 

 
1.0
 

 
Q9VYT0
 

 
6.80316e-11
 

 
1.0
 

 
0.9285714
 

 
308
 

 
T
 

 
NIL
 

 

 

 
GKFG-1734-MONOMER
 

 
pad:TIIST44_04425 HAD hyd
 

 
0.007427732
 

 
1.0
 

 
Q9VYT0
 

 
1.72256e-9
 

 
2.0
 

 
0.9285714
 

 
308
 

 
T
 

 
NIL
 

 

 
     Evidence for farnesyl diphosphate phosphatase, EC# 3.6.1.-   RXN-11776 
  

 

 
 Hit 
 

 
 Common 
 

 
 P 
 

 
 #Q 
 

 
 Best Qry 
 

 
 Best Eval 
 

 
 Avg Rank 
 

 
 Aln Len 
 

 
 Qry Len 
 

 
 IDOP? 
 

 
 adj? 
 

 

 

 
GKFG-2023-MONOMER
 

 
mcu:HMPREF0573_11895 hypo
 

 
0.8249092
 

 
1.0
 

 
Q9VYT0
 

 
6.80316e-11
 

 
1.0
 

 
0.9285714
 

 
308
 

 
T
 

 
NIL
 

 

 

 
GKFG-1734-MONOMER
 

 
pad:TIIST44_04425 HAD hyd
 

 
0.007427732
 

 
1.0
 

 
Q9VYT0
 

 
1.72256e-9
 

 
2.0
 

 
0.9285714
 

 
308
 

 
T
 

 
NIL
 

 

 
     

  juvenile hormone III biosynthesis II     Total # of reactions in pathway = 5   Present reactions: 1
  

 

 
 Reaction 
 

 
 Protein(s) 
 

 

 

 
RXN-11619
 

 
(# )
 

 

 
   Missing reactions: 4
  

 

 
 RXN-8617 
 

 

 

 
 FARNESOL-DEHYDROGENASE-RXN 
 

 

 

 
 RXN-11770 
 

 

 

 
 RXN-11771 
 

 

 
        Evidence for farnesyl diphosphatase, EC# 3.1.7.6   RXN-8617 
  
no BLAST hits found
 
     Evidence for farnesol dehydrogenase, EC# 1.1.1.216   FARNESOL-DEHYDROGENASE-RXN 
  

 

 
 Hit 
 

 
 Common 
 

 
 P 
 

 
 #Q 
 

 
 Best Qry 
 

 
 Best Eval 
 

 
 Avg Rank 
 

 
 Aln Len 
 

 
 Qry Len 
 

 
 IDOP? 
 

 
 adj? 
 

 

 

 
GKFG-720-MONOMER
 

 
fabG; 3-ketoacyl-ACP redu
 

 
0.8249092
 

 
2.0
 

 
D2WKD9
 

 
1.76182e-13
 

 
1.0
 

 
0.94478923
 

 
245
 

 
T
 

 
NIL
 

 

 

 
GKFG-803-MONOMER
 

 
short-chain dehydrogenase
 

 
0.15667598
 

 
2.0
 

 
D2WKD9
 

 
5.92199e-12
 

 
2.0
 

 
0.78527105
 

 
245
 

 
T
 

 
NIL
 

 

 
     Evidence for , EC# 1.14.13.-   RXN-11770      Evidence for , EC# 2.1.1.-   RXN-11771 
  
no BLAST hits found
 
     

  salidroside biosynthesis     Total # of reactions in pathway = 4   Present reactions: 1
  

 

 
 Reaction 
 

 
 Protein(s) 
 

 

 

 
RXN3O-4113
 

 
(# 
 # )
 

 

 
   Missing reactions: 3
  

 

 
 RXN-12370 
 

 

 

 
 RXN-5821 
 

 

 

 
 TYROSINE-DECARBOXYLASE-RXN 
 

 

 
        Evidence for    RXN-12370 
  
no BLAST hits found
 
     Evidence for    RXN-5821      Evidence for tyrosine decarboxylase, EC# 4.1.1.25   TYROSINE-DECARBOXYLASE-RXN 
  

 

 
 Hit 
 

 
 Common 
 

 
 P 
 

 
 #Q 
 

 
 Best Qry 
 

 
 Best Eval 
 

 
 Avg Rank 
 

 
 Aln Len 
 

 
 Qry Len 
 

 
 IDOP? 
 

 
 adj? 
 

 

 

 
GKFG-206-MONOMER
 

 
cef:CE1295 respiratory ni
 

 
4.188785e-5
 

 
1.0
 

 
Q2FSD2
 

 
0.456139
 

 
1.0
 

 
0.14092141
 

 
369
 

 
NIL
 

 
NIL
 

 

 

 
GKFG-607-MONOMER
 

 
response regulator receiv
 

 
1.3945618e-5
 

 
1.0
 

 
Q2NHY7
 

 
0.311958
 

 
1.0
 

 
0.08226221
 

 
389
 

 
T
 

 
NIL
 

 

 

 
GKFG-922-MONOMER
 

 
short-chain dehydrogenase
 

 
1.3945618e-5
 

 
1.0
 

 
Q0W498
 

 
0.199603
 

 
1.0
 

 
0.061333332
 

 
375
 

 
T
 

 
NIL
 

 

 

 
GKFG-1295-MONOMER
 

 
ATP-dependent DNA helicas
 

 
2.5282066e-6
 

 
1.0
 

 
Q2NHY7
 

 
0.673001
 

 
2.0
 

 
0.095115684
 

 
389
 

 
T
 

 
NIL
 

 

 
     

  phenylethanol biosynthesis     Total # of reactions in pathway = 4   Present reactions: 1
  

 

 
 Reaction 
 

 
 Protein(s) 
 

 

 

 
RXN-7700
 

 
(# 
 # )
 

 

 
   Missing reactions: 3
  

 

 
 PHENYLALANINE-DECARBOXYLASE-RXN 
 

 

 

 
 AMINEPHEN-RXN 
 

 

 

 
 RXN-8990 
 

 

 
        Evidence for phenylalanine decarboxylase, EC# 4.1.1.53   PHENYLALANINE-DECARBOXYLASE-RXN 
  
no BLAST hits found
 
     Evidence for , EC# 1.4.3.21   AMINEPHEN-RXN 
  
no BLAST hits found
 
     Evidence for    RXN-8990 
  

 

 
 Hit 
 

 
 Common 
 

 
 P 
 

 
 #Q 
 

 
 Best Qry 
 

 
 Best Eval 
 

 
 Avg Rank 
 

 
 Aln Len 
 

 
 Qry Len 
 

 
 IDOP? 
 

 
 adj? 
 

 

 

 
GKFG-1777-MONOMER
 

 
cur:cur_0492 surB; cell s
 

 
1.5421621e-5
 

 
1.0
 

 
Q0ZS27
 

 
0.834116
 

 
1.0
 

 
0.22440945
 

 
508
 

 
T
 

 
NIL
 

 

 
     

  3-amino-5-hydroxybenzoate biosynthesis     Total # of reactions in pathway = 7   Present reactions: 2
  

 

 
 Reaction 
 

 
 Protein(s) 
 

 

 

 
RXN-9583
 

 
(# )
 

 

 

 
RXN-9584
 

 
(# )
 

 

 
   Missing reactions: 5
  

 

 
 RXN-9581 
 

 

 

 
 RXN-9582 
 

 

 

 
 RXN-9585 
 

 

 

 
 RXN-9586 
 

 

 

 
 RXN-9587 
 

 

 
        Evidence for    RXN-9581 
  

 

 
 Hit 
 

 
 Common 
 

 
 P 
 

 
 #Q 
 

 
 Best Qry 
 

 
 Best Eval 
 

 
 Avg Rank 
 

 
 Aln Len 
 

 
 Qry Len 
 

 
 IDOP? 
 

 
 adj? 
 

 

 

 
GKFG-1492-MONOMER
 

 
ROK family protein; K0084
 

 
0.8249092
 

 
1.0
 

 
O52554
 

 
6.53866e-12
 

 
1.0
 

 
0.93191487
 

 
235
 

 
T
 

 
NIL
 

 

 

 
GKFG-427-MONOMER
 

 
pfr:PFREUD_19060 iolC; my
 

 
9.0592475e-5
 

 
1.0
 

 
O52554
 

 
0.559962
 

 
4.0
 

 
0.3617021
 

 
235
 

 
NIL
 

 
NIL
 

 

 

 
GKFG-1528-MONOMER
 

 
ROK family protein (db=KE
 

 
5.771539e-5
 

 
1.0
 

 
O52554
 

 
3.46914e-6
 

 
2.0
 

 
0.45957446
 

 
235
 

 
T
 

 
NIL
 

 

 

 
GKFG-1150-MONOMER
 

 
iva:Isova_1870 polyphosph
 

 
1.09376415e-5
 

 
1.0
 

 
O52554
 

 
0.0465786
 

 
3.0
 

 
0.33617023
 

 
235
 

 
T
 

 
NIL
 

 

 
     Evidence for    RXN-9582      Evidence for    RXN-9585      Evidence for    RXN-9586      Evidence for    RXN-9587 
  

 

 
 Hit 
 

 
 Common 
 

 
 P 
 

 
 #Q 
 

 
 Best Qry 
 

 
 Best Eval 
 

 
 Avg Rank 
 

 
 Aln Len 
 

 
 Qry Len 
 

 
 IDOP? 
 

 
 adj? 
 

 

 

 
GKFG-1134-MONOMER
 

 
seg (db=Seg db_id=seg fro
 

 
1.3945618e-5
 

 
1.0
 

 
tr|O52552|O52552_AMYMD
 

 
0.403721
 

 
1.0
 

 
0.09793814
 

 
388
 

 
T
 

 
NIL
 

 

 

 
GKFG-714-MONOMER
 

 
bpo:BP951000_0640 putativ
 

 
2.5282066e-6
 

 
1.0
 

 
tr|O52552|O52552_AMYMD
 

 
0.937362
 

 
2.0
 

 
0.087628864
 

 
388
 

 
T
 

 
NIL
 

 

 
     

  streptomycin biosynthesis     Total # of reactions in pathway = 18   Present reactions: 3
  

 

 
 Reaction 
 

 
 Protein(s) 
 

 

 

 
MYO-INOSITOL-2-DEHYDROGENASE-RXN
 

 
(# 
 # 
 # 
 # )
 

 

 

 
2.6.1.50-RXN
 

 
(# )
 

 

 

 
PHOSPHOGLUCMUT-RXN
 

 
(# )
 

 

 
   Missing reactions: 15
  

 

 
 SCYLLO-INOSAMINE-KINASE-RXN 
 

 

 

 
 2.1.4.2-RXN 
 

 

 

 
 2.4.2.27-RXN 
 

 

 

 
 RXN-9470 
 

 

 

 
 RXN-9471 
 

 

 

 
 STREPTOMYCIN-6-PHOSPHATASE-RXN 
 

 

 

 
 RXN-9467 
 

 

 

 
 RXN-9469 
 

 

 

 
 2.7.7.33-RXN 
 

 

 

 
 STREPTOMYCIN-6-KINASE-RXN 
 

 

 

 
 3.1.3.40-RXN 
 

 

 

 
 RXN-11097 
 

 

 

 
 2.6.1.56-RXN 
 

 

 

 
 RXN-11098 
 

 

 

 
 RXN-11099 
 

 

 
        Evidence for  scyllo -inosamine 4-kinase, EC# 2.7.1.65   SCYLLO-INOSAMINE-KINASE-RXN      Evidence for  scyllo -inosamine-4-phosphate amidinotransferase, EC# 2.1.4.2   2.1.4.2-RXN 
  

 

 
 Hit 
 

 
 Common 
 

 
 P 
 

 
 #Q 
 

 
 Best Qry 
 

 
 Best Eval 
 

 
 Avg Rank 
 

 
 Aln Len 
 

 
 Qry Len 
 

 
 IDOP? 
 

 
 adj? 
 

 

 

 
GKFG-494-MONOMER
 

 
hypothetical protein (db=
 

 
4.188785e-5
 

 
1.0
 

 
P29780
 

 
0.5291
 

 
1.0
 

 
0.14899713
 

 
349
 

 
NIL
 

 
NIL
 

 

 
     Evidence for dTDP-dihydrostreptose&mdash;streptidine-6-phosphate dihydrostreptosyltransferase, EC# 2.4.2.27   2.4.2.27-RXN 
  
no BLAST hits found
 
     Evidence for    RXN-9470      Evidence for    RXN-9471      Evidence for streptomycin-6-phosphatase, EC# 3.1.3.39   STREPTOMYCIN-6-PHOSPHATASE-RXN 
  

 

 
 Hit 
 

 
 Common 
 

 
 P 
 

 
 #Q 
 

 
 Best Qry 
 

 
 Best Eval 
 

 
 Avg Rank 
 

 
 Aln Len 
 

 
 Qry Len 
 

 
 IDOP? 
 

 
 adj? 
 

 

 

 
GKFG-997-MONOMER
 

 
ssm:Spirs_0789 aldehyde d
 

 
1.5421621e-5
 

 
1.0
 

 
P09401
 

 
0.245307
 

 
1.0
 

 
0.12917595
 

 
449
 

 
T
 

 
NIL
 

 

 
     Evidence for    RXN-9467      Evidence for    RXN-9469      Evidence for glucose-1-phosphate cytidylyltransferase, EC# 2.7.7.33   2.7.7.33-RXN 
  
no BLAST hits found
 
     Evidence for streptomycin 6-kinase, EC# 2.7.1.72   STREPTOMYCIN-6-KINASE-RXN 
  

 

 
 Hit 
 

 
 Common 
 

 
 P 
 

 
 #Q 
 

 
 Best Qry 
 

 
 Best Eval 
 

 
 Avg Rank 
 

 
 Aln Len 
 

 
 Qry Len 
 

 
 IDOP? 
 

 
 adj? 
 

 

 

 
GKFG-1240-MONOMER
 

 
hypothetical protein (db=
 

 
2.5307965e-5
 

 
1.0
 

 
P18622
 

 
0.75145
 

 
2.0
 

 
0.247557
 

 
307
 

 
T
 

 
NIL
 

 

 

 
GKFG-239-MONOMER
 

 
bcv:Bcav_1088 hemH; ferro
 

 
1.5421621e-5
 

 
2.0
 

 
P08077
 

 
0.130737
 

 
1.0
 

 
0.22312704
 

 
307
 

 
T
 

 
NIL
 

 

 
     Evidence for guanidinodeoxy- scyllo -inositol-4-phosphatase, EC# 3.1.3.40   3.1.3.40-RXN      Evidence for 1-guanidino-1-deoxy-scyllo-inositol dehydrogenase   RXN-11097      Evidence for 1 D -1-guanidino-3-amino-1,3-dideoxy- scyllo -inositol transaminase, EC# 2.6.1.56   2.6.1.56-RXN      Evidence for ATP:1D-1-guanidino-3-amino-1,3-dideoxy-scyllo-inositol 6-phosphotransferase   RXN-11098      Evidence for  N 1-amidinostreptamine 6-phosphate aminotransferase   RXN-11099 
  
no BLAST hits found
 
     

  D- myo -inositol (1,4,5)-trisphosphate degradation     Total # of reactions in pathway = 3   Present reactions: 1
  

 

 
 Reaction 
 

 
 Protein(s) 
 

 

 

 
RXN-10952
 

 
(# 
 # )
 

 

 
   Missing reactions: 2
  

 

 
 3.1.3.57-RXN 
 

 

 

 
 3.1.3.56-RXN 
 

 

 
        Evidence for inositol-1,4-bisphosphate 1-phosphatase, EC# 3.1.3.57   3.1.3.57-RXN 
  

 

 
 Hit 
 

 
 Common 
 

 
 P 
 

 
 #Q 
 

 
 Best Qry 
 

 
 Best Eval 
 

 
 Avg Rank 
 

 
 Aln Len 
 

 
 Qry Len 
 

 
 IDOP? 
 

 
 adj? 
 

 

 

 
GKFG-681-MONOMER
 

 
archaeal fructose-1,6-bis
 

 
0.07274204
 

 
2.0
 

 
Q8GY63
 

 
0.00199453
 

 
1.0
 

 
0.34399784
 

 
357
 

 
T
 

 
T
 

 

 
     Evidence for inositol-polyphosphate 5-phosphatase, EC# 3.1.3.56   3.1.3.56-RXN 
  

 

 
 Hit 
 

 
 Common 
 

 
 P 
 

 
 #Q 
 

 
 Best Qry 
 

 
 Best Eval 
 

 
 Avg Rank 
 

 
 Aln Len 
 

 
 Qry Len 
 

 
 IDOP? 
 

 
 adj? 
 

 

 

 
GKFG-1616-MONOMER
 

 
bfa:Bfae_18570 transcript
 

 
1.3945618e-5
 

 
1.0
 

 
Q15735
 

 
0.618958
 

 
1.0
 

 
0.040755466
 

 
1006
 

 
T
 

 
NIL
 

 

 
     

  mycolate biosynthesis     Total # of reactions in pathway = 192   Present reactions: 28
  

 

 
 Reaction 
 

 
 Protein(s) 
 

 

 

 
RXN1G-469
 

 
(# )
 

 

 

 
RXN1G-157
 

 
(# )
 

 

 

 
RXN1G-72
 

 
(# )
 

 

 

 
RXN1G-364
 

 
(# )
 

 

 

 
RXN1G-240
 

 
(# )
 

 

 

 
RXN1G-163
 

 
(# )
 

 

 

 
RXN1G-260
 

 
(# )
 

 

 

 
RXN1G-358
 

 
(# )
 

 

 

 
RXN1G-881
 

 
(# )
 

 

 

 
RXN1G-1053
 

 
(# )
 

 

 

 
RXN1G-287
 

 
(# )
 

 

 

 
RXN1G-408
 

 
(# )
 

 

 

 
RXN1G-262
 

 
(# )
 

 

 

 
RXN1G-252
 

 
(# )
 

 

 

 
RXN1G-637
 

 
(# )
 

 

 

 
RXN1G-203
 

 
(# )
 

 

 

 
RXN1G-481
 

 
(# )
 

 

 

 
RXN1G-717
 

 
(# )
 

 

 

 
RXN1G-951
 

 
(# )
 

 

 

 
RXN1G-182
 

 
(# )
 

 

 

 
RXN1G-384
 

 
(# )
 

 

 

 
RXN1G-613
 

 
(# )
 

 

 

 
RXN1G-853
 

 
(# )
 

 

 

 
RXN1G-1050
 

 
(# )
 

 

 

 
RXN1G-1247
 

 
(# )
 

 

 

 
RXN1G-184
 

 
(# )
 

 

 

 
RXN1G-409
 

 
(# )
 

 

 

 
RXN1G-617
 

 
(# )
 

 

 
   Missing reactions: 164
  

 

 
 RXN1G-368 
 

 

 

 
 RXN1G-349 
 

 

 

 
 RXN1G-320 
 

 

 

 
 RXN1G-395 
 

 

 

 
 RXN1G-445 
 

 

 

 
 RXN1G-479 
 

 

 

 
 RXN1G-488 
 

 

 

 
 RXN1G-499 
 

 

 

 
 RXN1G-508 
 

 

 

 
 RXN1G-517 
 

 

 

 
 RXN1G-526 
 

 

 

 
 RXN1G-536 
 

 

 

 
 RXN1G-574 
 

 

 

 
 RXN1G-460 
 

 

 

 
 RXN1G-363 
 

 

 

 
 RXN1G-30 
 

 

 

 
 RXN1G-26 
 

 

 

 
 RXN1G-27 
 

 

 

 
 RXN1G-171 
 

 

 

 
 RXN1G-306 
 

 

 

 
 RXN1G-420 
 

 

 

 
 RXN1G-37 
 

 

 

 
 RXN1G-218 
 

 

 

 
 RXN1G-266 
 

 

 

 
 RXN1G-285 
 

 

 

 
 RXN1G-138 
 

 

 

 
 RXN1G-188 
 

 

 

 
 RXN1G-210 
 

 

 

 
 RXN1G-236 
 

 

 

 
 RXN1G-279 
 

 

 

 
 RXN1G-299 
 

 

 

 
 RXN1G-324 
 

 

 

 
 RXN1G-377 
 

 

 

 
 RXN1G-396 
 

 

 

 
 RXN1G-674 
 

 

 

 
 RXN1G-840 
 

 

 

 
 RXN1G-912 
 

 

 

 
 RXN1G-962 
 

 

 

 
 RXN1G-1003 
 

 

 

 
 RXN1G-1084 
 

 

 

 
 RXN1G-1130 
 

 

 

 
 RXN1G-132 
 

 

 

 
 RXN1G-355 
 

 

 

 
 RXN1G-53 
 

 

 

 
 RXN1G-202 
 

 

 

 
 RXN1G-464 
 

 

 

 
 RXN1G-193 
 

 

 

 
 RXN1G-89 
 

 

 

 
 RXN1G-308 
 

 

 

 
 RXN1G-339 
 

 

 

 
 RXN1G-172 
 

 

 

 
 RXN1G-309 
 

 

 

 
 RXN1G-509 
 

 

 

 
 RXN1G-580 
 

 

 

 
 RXN1G-127 
 

 

 

 
 RXN1G-196 
 

 

 

 
 RXN1G-32 
 

 

 

 
 RXN1G-294 
 

 

 

 
 RXN1G-337 
 

 

 

 
 RXN1G-424 
 

 

 

 
 RXN1G-554 
 

 

 

 
 RXN1G-607 
 

 

 

 
 RXN1G-660 
 

 

 

 
 RXN1G-760 
 

 

 

 
 RXN1G-820 
 

 

 

 
 RXN1G-883 
 

 

 

 
 RXN1G-1004 
 

 

 

 
 RXN1G-1057 
 

 

 

 
 RXN1G-79 
 

 

 

 
 RXN1G-214 
 

 

 

 
 RXN1G-248 
 

 

 

 
 RXN1G-334 
 

 

 

 
 RXN1G-425 
 

 

 

 
 RXN1G-468 
 

 

 

 
 RXN1G-557 
 

 

 

 
 RXN1G-645 
 

 

 

 
 RXN1G-686 
 

 

 

 
 RXN1G-818 
 

 

 

 
 RXN1G-884 
 

 

 

 
 RXN1G-915 
 

 

 

 
 RXN1G-1015 
 

 

 

 
 RXN1G-1081 
 

 

 

 
 RXN1G-1117 
 

 

 

 
 RXN1G-1212 
 

 

 

 
 RXN1G-1278 
 

 

 

 
 RXN1G-1325 
 

 

 

 
 RXN1G-94 
 

 

 

 
 RXN1G-220 
 

 

 

 
 RXN1G-276 
 

 

 

 
 RXN1G-374 
 

 

 

 
 RXN1G-440 
 

 

 

 
 RXN1G-471 
 

 

 

 
 RXN1G-582 
 

 

 

 
 RXN1G-648 
 

 

 

 
 RXN1G-679 
 

 

 

 
 RXN1G-45 
 

 

 

 
 RXN1G-2527 
 

 

 

 
 RXN1G-2544 
 

 

 

 
 RXN1G-3232 
 

 

 

 
 RXN1G-3256 
 

 

 

 
 RXN1G-3613 
 

 

 

 
 RXN1G-3641 
 

 

 

 
 RXN1G-3660 
 

 

 

 
 RXN1G-3667 
 

 

 

 
 RXN1G-3993 
 

 

 

 
 RXN1G-4140 
 

 

 

 
 RXN1G-4141 
 

 

 

 
 RXN1G-4142 
 

 

 

 
 RXN1G-4143 
 

 

 

 
 RXN1G-4355 
 

 

 

 
 RXN1G-181 
 

 

 

 
 RXN1G-98 
 

 

 

 
 RXN1G-297 
 

 

 

 
 RXN1G-295 
 

 

 

 
 RXN1G-296 
 

 

 

 
 RXN1G-576 
 

 

 

 
 RXN1G-697 
 

 

 

 
 RXN1G-867 
 

 

 

 
 RXN1G-865 
 

 

 

 
 RXN1G-866 
 

 

 

 
 RXN1G-886 
 

 

 

 
 RXN1G-887 
 

 

 

 
 RXN1G-888 
 

 

 

 
 RXN1G-890 
 

 

 

 
 RXN1G-891 
 

 

 

 
 RXN1G-1486 
 

 

 

 
 RXN1G-1527 
 

 

 

 
 RXN1G-1528 
 

 

 

 
 RXN1G-1529 
 

 

 

 
 RXN1G-1530 
 

 

 

 
 RXN1G-288 
 

 

 

 
 RXN1G-289 
 

 

 

 
 RXN1G-290 
 

 

 

 
 RXN1G-291 
 

 

 

 
 RXN1G-292 
 

 

 

 
 RXN1G-807 
 

 

 

 
 RXN1G-808 
 

 

 

 
 RXN1G-809 
 

 

 

 
 RXN1G-810 
 

 

 

 
 RXN1G-811 
 

 

 

 
 RXN1G-1435 
 

 

 

 
 RXN1G-1436 
 

 

 

 
 RXN1G-1437 
 

 

 

 
 RXN1G-1438 
 

 

 

 
 RXN1G-1439 
 

 

 

 
 RXN1G-137 
 

 

 

 
 RXN1G-470 
 

 

 

 
 RXN1G-472 
 

 

 

 
 RXN1G-473 
 

 

 

 
 RXN1G-474 
 

 

 

 
 RXN1G-874 
 

 

 

 
 RXN1G-1149 
 

 

 

 
 RXN1G-1150 
 

 

 

 
 RXN1G-1151 
 

 

 

 
 RXN1G-1152 
 

 

 

 
 RXN1G-1633 
 

 

 

 
 RXN1G-1634 
 

 

 

 
 RXN1G-1635 
 

 

 

 
 RXN1G-1636 
 

 

 

 
 RXN1G-1637 
 

 

 

 
 RXN-10059 
 

 

 

 
 RXN-10060 
 

 

 

 
 RXN-10061 
 

 

 

 
 RXN-10062 
 

 

 
        Evidence for 3-oxo-arachidoyl-[acp] synthase, EC# 2.3.1.-   RXN1G-368 
  
no BLAST hits found
 
     Evidence for 3-oxo-arachidoyl-[acp] reductase, EC# 2.3.1.-   RXN1G-349 
  
no BLAST hits found
 
     Evidence for 3-hydroxyarchidoyl-[acp] dehydrase, EC# 2.3.1.-   RXN1G-320 
  
no BLAST hits found
 
     Evidence for trans-eicos-2-enoyl-[acyl-carrier protein] reductase (NADPH, B-specific), EC# 2.3.1.-   RXN1G-395 
  
no BLAST hits found
 
     Evidence for 3-oxo-behenoyl-[acp] synthase, EC# 2.3.1.-   RXN1G-445 
  
no BLAST hits found
 
     Evidence for 3-hydroxybehenoyl-[acp] dehydrase, EC# 4.2.1.-   RXN1G-479 
  
no BLAST hits found
 
     Evidence for trans-docos-2-enoyl-[acyl-carrier protein] reductase (NADPH, B-specific), EC# 2.3.1.-   RXN1G-488 
  
no BLAST hits found
 
     Evidence for 3-oxo-lignoceroyl-[acyl-carrier protein] synthase, EC# 2.3.1.-   RXN1G-499 
  
no BLAST hits found
 
     Evidence for 3-oxo-lignoceroyl-[acyl-carrier protein] reductase, EC# 2.3.1.-   RXN1G-508 
  
no BLAST hits found
 
     Evidence for 3-hydroxylignoceroyl-[acyl-carrier protein] dehydratase, EC# 2.3.1.-   RXN1G-517 
  
no BLAST hits found
 
     Evidence for trans-tetracos-2-enoyl-[acyl-carrier protein] reductase (NADPH, B-specific), EC# 2.3.1.-   RXN1G-526 
  
no BLAST hits found
 
     Evidence for cerotoyl-[acyl-carrier protein] reductase, EC# 2.3.1.-   RXN1G-536 
  
no BLAST hits found
 
     Evidence for arachidoyl-[acyl-carrier protein] reductase, EC# 2.3.1.-   RXN1G-574 
  
no BLAST hits found
 
     Evidence for 3-oxo-behenoyl-[acyl-carrier protein] synthase, EC# 2.3.1.41   RXN1G-460      Evidence for 3-hydroxybehenoyl-[acyl-carrier protein] dehydratase   RXN1G-363 
  

 

 
 Hit 
 

 
 Common 
 

 
 P 
 

 
 #Q 
 

 
 Best Qry 
 

 
 Best Eval 
 

 
 Avg Rank 
 

 
 Aln Len 
 

 
 Qry Len 
 

 
 IDOP? 
 

 
 adj? 
 

 

 

 
GKFG-904-MONOMER
 

 
pfr:PFREUD_02450 maoC; Ma
 

 
0.0051016076
 

 
1.0
 

 
tr|P96927|P96927_MYCTU
 

 
0.00452189
 

 
1.0
 

 
0.6126761
 

 
142
 

 
T
 

 
NIL
 

 

 

 
GKFG-1225-MONOMER
 

 
mcu:HMPREF0573_11305 hypo
 

 
9.2874106e-4
 

 
1.0
 

 
tr|P96927|P96927_MYCTU
 

 
0.879797
 

 
2.0
 

 
0.64788735
 

 
142
 

 
T
 

 
NIL
 

 

 
     Evidence for trans-docos-2-enoyl-[acyl-carrier protein] reductase   RXN1G-30      Evidence for cis-delta5-3-oxo-C24:1-[acyl-carrier protein] synthase, EC# 2.3.1.41   RXN1G-26      Evidence for cis-delta5-3-hydroxyC24:1-[acyl-carrier protein] dehydratase   RXN1G-27 
  

 

 
 Hit 
 

 
 Common 
 

 
 P 
 

 
 #Q 
 

 
 Best Qry 
 

 
 Best Eval 
 

 
 Avg Rank 
 

 
 Aln Len 
 

 
 Qry Len 
 

 
 IDOP? 
 

 
 adj? 
 

 

 

 
GKFG-904-MONOMER
 

 
pfr:PFREUD_02450 maoC; Ma
 

 
0.0051016076
 

 
1.0
 

 
tr|P96927|P96927_MYCTU
 

 
0.00452189
 

 
1.0
 

 
0.6126761
 

 
142
 

 
T
 

 
NIL
 

 

 

 
GKFG-1225-MONOMER
 

 
mcu:HMPREF0573_11305 hypo
 

 
9.2874106e-4
 

 
1.0
 

 
tr|P96927|P96927_MYCTU
 

 
0.879797
 

 
2.0
 

 
0.64788735
 

 
142
 

 
T
 

 
NIL
 

 

 
     Evidence for trans-delta2-cis-delta5-C24:2-[acyl-carrier protein] reductase (NADH, B-specific), EC# 1.3.1.10   RXN1G-171      Evidence for cis-delta7-3-oxo-C26:1-[acyl-carrier protein] synthase, EC# 2.3.1.41   RXN1G-306      Evidence for cis-delta7-3-hydroxyC26:1-[acyl-carrier protein] dehydratase   RXN1G-420 
  

 

 
 Hit 
 

 
 Common 
 

 
 P 
 

 
 #Q 
 

 
 Best Qry 
 

 
 Best Eval 
 

 
 Avg Rank 
 

 
 Aln Len 
 

 
 Qry Len 
 

 
 IDOP? 
 

 
 adj? 
 

 

 

 
GKFG-904-MONOMER
 

 
pfr:PFREUD_02450 maoC; Ma
 

 
0.0051016076
 

 
1.0
 

 
tr|P96927|P96927_MYCTU
 

 
0.00452189
 

 
1.0
 

 
0.6126761
 

 
142
 

 
T
 

 
NIL
 

 

 

 
GKFG-1225-MONOMER
 

 
mcu:HMPREF0573_11305 hypo
 

 
9.2874106e-4
 

 
1.0
 

 
tr|P96927|P96927_MYCTU
 

 
0.879797
 

 
2.0
 

 
0.64788735
 

 
142
 

 
T
 

 
NIL
 

 

 
     Evidence for trans-delta2-cis-delta7-C26:2-[acyl-carrier protein] reductase (NADH, B-specific), EC# 1.3.1.10   RXN1G-37      Evidence for cis-delta9-3-oxo-C28:1-[acyl-carrier protein] synthase, EC# 2.3.1.41   RXN1G-218      Evidence for cis-delta9-3-hydroxyC28:1-[acyl-carrier protein] dehydratase   RXN1G-266 
  

 

 
 Hit 
 

 
 Common 
 

 
 P 
 

 
 #Q 
 

 
 Best Qry 
 

 
 Best Eval 
 

 
 Avg Rank 
 

 
 Aln Len 
 

 
 Qry Len 
 

 
 IDOP? 
 

 
 adj? 
 

 

 

 
GKFG-904-MONOMER
 

 
pfr:PFREUD_02450 maoC; Ma
 

 
0.0051016076
 

 
1.0
 

 
tr|P96927|P96927_MYCTU
 

 
0.00452189
 

 
1.0
 

 
0.6126761
 

 
142
 

 
T
 

 
NIL
 

 

 

 
GKFG-1225-MONOMER
 

 
mcu:HMPREF0573_11305 hypo
 

 
9.2874106e-4
 

 
1.0
 

 
tr|P96927|P96927_MYCTU
 

 
0.879797
 

 
2.0
 

 
0.64788735
 

 
142
 

 
T
 

 
NIL
 

 

 
     Evidence for trans-delta2-cis-delta9-C28:2-[acyl-carrier protein] reductase (NADH, B-specific), EC# 1.3.1.10   RXN1G-285      Evidence for cis-delta11-3-oxo-C30:1-[acyl-carrier protein] synthase, EC# 2.3.1.41   RXN1G-138      Evidence for cis-delta11-3-hydroxyC30:1-[acyl-carrier protein] dehydratase   RXN1G-188 
  

 

 
 Hit 
 

 
 Common 
 

 
 P 
 

 
 #Q 
 

 
 Best Qry 
 

 
 Best Eval 
 

 
 Avg Rank 
 

 
 Aln Len 
 

 
 Qry Len 
 

 
 IDOP? 
 

 
 adj? 
 

 

 

 
GKFG-904-MONOMER
 

 
pfr:PFREUD_02450 maoC; Ma
 

 
0.0051016076
 

 
1.0
 

 
tr|P96927|P96927_MYCTU
 

 
0.00452189
 

 
1.0
 

 
0.6126761
 

 
142
 

 
T
 

 
NIL
 

 

 

 
GKFG-1225-MONOMER
 

 
mcu:HMPREF0573_11305 hypo
 

 
9.2874106e-4
 

 
1.0
 

 
tr|P96927|P96927_MYCTU
 

 
0.879797
 

 
2.0
 

 
0.64788735
 

 
142
 

 
T
 

 
NIL
 

 

 
     Evidence for trans-delta2-cis-delta11-C30:2[acyl-carrier protein] reductase (NADH, B-specific), EC# 1.3.1.10   RXN1G-210      Evidence for cis-delta13-3-oxo-C32:1-[acyl-carrier protein] synthase, EC# 2.3.1.41   RXN1G-236      Evidence for cis-delta13-3-hydroxyC32:1-[acyl-carrier protein] dehydratase   RXN1G-279 
  

 

 
 Hit 
 

 
 Common 
 

 
 P 
 

 
 #Q 
 

 
 Best Qry 
 

 
 Best Eval 
 

 
 Avg Rank 
 

 
 Aln Len 
 

 
 Qry Len 
 

 
 IDOP? 
 

 
 adj? 
 

 

 

 
GKFG-904-MONOMER
 

 
pfr:PFREUD_02450 maoC; Ma
 

 
0.0051016076
 

 
1.0
 

 
tr|P96927|P96927_MYCTU
 

 
0.00452189
 

 
1.0
 

 
0.6126761
 

 
142
 

 
T
 

 
NIL
 

 

 

 
GKFG-1225-MONOMER
 

 
mcu:HMPREF0573_11305 hypo
 

 
9.2874106e-4
 

 
1.0
 

 
tr|P96927|P96927_MYCTU
 

 
0.879797
 

 
2.0
 

 
0.64788735
 

 
142
 

 
T
 

 
NIL
 

 

 
     Evidence for trans-delta2-cis-delta13-C32:2-[acyl-carrier protein] reductase (NADPH, B-specific), EC# 1.3.1.10   RXN1G-299      Evidence for cis-delta15-3-oxo-C34:1-[acyl-carrier protein] synthase, EC# 2.3.1.41   RXN1G-324      Evidence for cis-delta15-3-hydroxyC34:1-[acyl-carrier protein] dehydratase   RXN1G-377 
  

 

 
 Hit 
 

 
 Common 
 

 
 P 
 

 
 #Q 
 

 
 Best Qry 
 

 
 Best Eval 
 

 
 Avg Rank 
 

 
 Aln Len 
 

 
 Qry Len 
 

 
 IDOP? 
 

 
 adj? 
 

 

 

 
GKFG-904-MONOMER
 

 
pfr:PFREUD_02450 maoC; Ma
 

 
0.0051016076
 

 
1.0
 

 
tr|P96927|P96927_MYCTU
 

 
0.00452189
 

 
1.0
 

 
0.6126761
 

 
142
 

 
T
 

 
NIL
 

 

 

 
GKFG-1225-MONOMER
 

 
mcu:HMPREF0573_11305 hypo
 

 
9.2874106e-4
 

 
1.0
 

 
tr|P96927|P96927_MYCTU
 

 
0.879797
 

 
2.0
 

 
0.64788735
 

 
142
 

 
T
 

 
NIL
 

 

 
     Evidence for trans-delta2-cis-delta15-C34:2-[acyl-carrier protein] reductase (NADPH, B-specific), EC# 1.3.1.10   RXN1G-396      Evidence for trans-delta2-cis-delta15-C34:2-[acyl-carrier protein] reductase   RXN1G-674      Evidence for cis-delta17-3-oxo-C36:1-[acyl-carrier protein] synthase, EC# 2.3.1.41   RXN1G-840      Evidence for cis-delta17-3-hydroxyC36:1-[acyl-carrier protein] dehydratase   RXN1G-912 
  

 

 
 Hit 
 

 
 Common 
 

 
 P 
 

 
 #Q 
 

 
 Best Qry 
 

 
 Best Eval 
 

 
 Avg Rank 
 

 
 Aln Len 
 

 
 Qry Len 
 

 
 IDOP? 
 

 
 adj? 
 

 

 

 
GKFG-904-MONOMER
 

 
pfr:PFREUD_02450 maoC; Ma
 

 
0.0051016076
 

 
1.0
 

 
tr|P96927|P96927_MYCTU
 

 
0.00452189
 

 
1.0
 

 
0.6126761
 

 
142
 

 
T
 

 
NIL
 

 

 

 
GKFG-1225-MONOMER
 

 
mcu:HMPREF0573_11305 hypo
 

 
9.2874106e-4
 

 
1.0
 

 
tr|P96927|P96927_MYCTU
 

 
0.879797
 

 
2.0
 

 
0.64788735
 

 
142
 

 
T
 

 
NIL
 

 

 
     Evidence for trans-delta2-cis-delta17-C36:2-[acyl-carrier protein] reductase, EC# 1.3.1.10   RXN1G-962      Evidence for cis-delta19-3-oxo-C38:1-[acyl-carrier protein] synthase, EC# 2.3.1.41   RXN1G-1003      Evidence for cis-delta19-3-hydroxyC38:1-[acyl-carrier protein] dehydratase   RXN1G-1084 
  

 

 
 Hit 
 

 
 Common 
 

 
 P 
 

 
 #Q 
 

 
 Best Qry 
 

 
 Best Eval 
 

 
 Avg Rank 
 

 
 Aln Len 
 

 
 Qry Len 
 

 
 IDOP? 
 

 
 adj? 
 

 

 

 
GKFG-904-MONOMER
 

 
pfr:PFREUD_02450 maoC; Ma
 

 
0.0051016076
 

 
1.0
 

 
tr|P96927|P96927_MYCTU
 

 
0.00452189
 

 
1.0
 

 
0.6126761
 

 
142
 

 
T
 

 
NIL
 

 

 

 
GKFG-1225-MONOMER
 

 
mcu:HMPREF0573_11305 hypo
 

 
9.2874106e-4
 

 
1.0
 

 
tr|P96927|P96927_MYCTU
 

 
0.879797
 

 
2.0
 

 
0.64788735
 

 
142
 

 
T
 

 
NIL
 

 

 
     Evidence for trans-delta2-cis-delta19-C38:2-[acyl-carrier protein] reductase, EC# 1.3.1.10   RXN1G-1130      Evidence for cis-delta21-3-oxo-C40:1-[acyl-carrier protein] synthase, EC# 2.3.1.41   RXN1G-132      Evidence for cis-delta21-3-hydroxyC40:1-[acyl-carrier protein] dehydratase   RXN1G-355 
  

 

 
 Hit 
 

 
 Common 
 

 
 P 
 

 
 #Q 
 

 
 Best Qry 
 

 
 Best Eval 
 

 
 Avg Rank 
 

 
 Aln Len 
 

 
 Qry Len 
 

 
 IDOP? 
 

 
 adj? 
 

 

 

 
GKFG-904-MONOMER
 

 
pfr:PFREUD_02450 maoC; Ma
 

 
0.0051016076
 

 
1.0
 

 
tr|P96927|P96927_MYCTU
 

 
0.00452189
 

 
1.0
 

 
0.6126761
 

 
142
 

 
T
 

 
NIL
 

 

 

 
GKFG-1225-MONOMER
 

 
mcu:HMPREF0573_11305 hypo
 

 
9.2874106e-4
 

 
1.0
 

 
tr|P96927|P96927_MYCTU
 

 
0.879797
 

 
2.0
 

 
0.64788735
 

 
142
 

 
T
 

 
NIL
 

 

 
     Evidence for trans-delta2-cis-delta21-C40:2-[acyl-carrier protein] reductase   RXN1G-53      Evidence for cis,cis-delta5,17-3-oxo-C36:2-[acyl-carrier protein] synthase, EC# 2.3.1.179   RXN1G-202      Evidence for cis,cis-delta5,17-3-hydroxyC36:2-[acyl-carrier protein] dehydratase   RXN1G-464 
  

 

 
 Hit 
 

 
 Common 
 

 
 P 
 

 
 #Q 
 

 
 Best Qry 
 

 
 Best Eval 
 

 
 Avg Rank 
 

 
 Aln Len 
 

 
 Qry Len 
 

 
 IDOP? 
 

 
 adj? 
 

 

 

 
GKFG-904-MONOMER
 

 
pfr:PFREUD_02450 maoC; Ma
 

 
0.0051016076
 

 
1.0
 

 
tr|P96927|P96927_MYCTU
 

 
0.00452189
 

 
1.0
 

 
0.6126761
 

 
142
 

 
T
 

 
NIL
 

 

 

 
GKFG-1225-MONOMER
 

 
mcu:HMPREF0573_11305 hypo
 

 
9.2874106e-4
 

 
1.0
 

 
tr|P96927|P96927_MYCTU
 

 
0.879797
 

 
2.0
 

 
0.64788735
 

 
142
 

 
T
 

 
NIL
 

 

 
     Evidence for trans-delta2-cis,cis-5,17-C36:3-[acyl-carrier protein] reductase (NADPH, B-specific), EC# 1.3.1.10   RXN1G-193      Evidence for cis,cis-delta7,19-3-oxo-C38:2-[acyl-carrier protein] synthase, EC# 2.3.1.179   RXN1G-89      Evidence for cis,cis-delta7,19-3-hydroxyC38:2-[acyl-carrier protein] dehydratase   RXN1G-308 
  

 

 
 Hit 
 

 
 Common 
 

 
 P 
 

 
 #Q 
 

 
 Best Qry 
 

 
 Best Eval 
 

 
 Avg Rank 
 

 
 Aln Len 
 

 
 Qry Len 
 

 
 IDOP? 
 

 
 adj? 
 

 

 

 
GKFG-904-MONOMER
 

 
pfr:PFREUD_02450 maoC; Ma
 

 
0.0051016076
 

 
1.0
 

 
tr|P96927|P96927_MYCTU
 

 
0.00452189
 

 
1.0
 

 
0.6126761
 

 
142
 

 
T
 

 
NIL
 

 

 

 
GKFG-1225-MONOMER
 

 
mcu:HMPREF0573_11305 hypo
 

 
9.2874106e-4
 

 
1.0
 

 
tr|P96927|P96927_MYCTU
 

 
0.879797
 

 
2.0
 

 
0.64788735
 

 
142
 

 
T
 

 
NIL
 

 

 
     Evidence for trans-delta2-cis-cis-delta7-19-C38:3-[acyl-carrier protein] reductase (NADPH, B-specific), EC# 1.3.1.10   RXN1G-339      Evidence for cis,cis-delta9,21-3-oxo-C40:2-[acyl-carrier protein] synthase, EC# 2.3.1.179   RXN1G-172      Evidence for cis,cis-delta9,21-3-hydroxyC40:2-[acyl-carrier protein] dehydratase   RXN1G-309 
  

 

 
 Hit 
 

 
 Common 
 

 
 P 
 

 
 #Q 
 

 
 Best Qry 
 

 
 Best Eval 
 

 
 Avg Rank 
 

 
 Aln Len 
 

 
 Qry Len 
 

 
 IDOP? 
 

 
 adj? 
 

 

 

 
GKFG-904-MONOMER
 

 
pfr:PFREUD_02450 maoC; Ma
 

 
0.0051016076
 

 
1.0
 

 
tr|P96927|P96927_MYCTU
 

 
0.00452189
 

 
1.0
 

 
0.6126761
 

 
142
 

 
T
 

 
NIL
 

 

 

 
GKFG-1225-MONOMER
 

 
mcu:HMPREF0573_11305 hypo
 

 
9.2874106e-4
 

 
1.0
 

 
tr|P96927|P96927_MYCTU
 

 
0.879797
 

 
2.0
 

 
0.64788735
 

 
142
 

 
T
 

 
NIL
 

 

 
     Evidence for trans-delta2-cis,cis-9,21-C40:3-[acyl-carrier protein] reductase (NADPH, B-specific), EC# 1.3.1.10   RXN1G-509      Evidence for cis,cis-delta11,23-3-oxo-C42:2-[acyl-carrier protein] synthase, EC# 2.3.1.179   RXN1G-580      Evidence for cis,cis-delta11,23-3-hydroxyC42:2-[acyl-carrier protein] dehydratase   RXN1G-127 
  

 

 
 Hit 
 

 
 Common 
 

 
 P 
 

 
 #Q 
 

 
 Best Qry 
 

 
 Best Eval 
 

 
 Avg Rank 
 

 
 Aln Len 
 

 
 Qry Len 
 

 
 IDOP? 
 

 
 adj? 
 

 

 

 
GKFG-904-MONOMER
 

 
pfr:PFREUD_02450 maoC; Ma
 

 
0.0051016076
 

 
1.0
 

 
tr|P96927|P96927_MYCTU
 

 
0.00452189
 

 
1.0
 

 
0.6126761
 

 
142
 

 
T
 

 
NIL
 

 

 

 
GKFG-1225-MONOMER
 

 
mcu:HMPREF0573_11305 hypo
 

 
9.2874106e-4
 

 
1.0
 

 
tr|P96927|P96927_MYCTU
 

 
0.879797
 

 
2.0
 

 
0.64788735
 

 
142
 

 
T
 

 
NIL
 

 

 
     Evidence for trans-delta2-cis,cis-delta11,23-C42:3-[acyl-carrier protein] reductase (NADPH, B-specific), EC# 1.3.1.10   RXN1G-196      Evidence for cis,cis-delta13,25-3-oxo-C44:2-[acyl-carrier protein] synthase, EC# 2.3.1.179   RXN1G-32      Evidence for 3-cis,cis-delta13,25-3-hydroxyC44:2-[acyl-carrier protein] dehydratase   RXN1G-294 
  

 

 
 Hit 
 

 
 Common 
 

 
 P 
 

 
 #Q 
 

 
 Best Qry 
 

 
 Best Eval 
 

 
 Avg Rank 
 

 
 Aln Len 
 

 
 Qry Len 
 

 
 IDOP? 
 

 
 adj? 
 

 

 

 
GKFG-904-MONOMER
 

 
pfr:PFREUD_02450 maoC; Ma
 

 
0.0051016076
 

 
1.0
 

 
tr|P96927|P96927_MYCTU
 

 
0.00452189
 

 
1.0
 

 
0.6126761
 

 
142
 

 
T
 

 
NIL
 

 

 

 
GKFG-1225-MONOMER
 

 
mcu:HMPREF0573_11305 hypo
 

 
9.2874106e-4
 

 
1.0
 

 
tr|P96927|P96927_MYCTU
 

 
0.879797
 

 
2.0
 

 
0.64788735
 

 
142
 

 
T
 

 
NIL
 

 

 

 
GKFG-1026-MONOMER
 

 
hypothetical protein (db=
 

 
1.5421621e-5
 

 
1.0
 

 
P96928
 

 
0.571235
 

 
1.0
 

 
0.21686748
 

 
166
 

 
T
 

 
NIL
 

 

 
     Evidence for trans-delta2-cis,cis-delta13,25-C44:3-[acyl-carrier protein] reductase (NADPH, B-specific), EC# 1.3.1.10   RXN1G-337      Evidence for cis,cis-delta15,27-3-oxo-C46:2-[acyl-carrier protein] synthase, EC# 2.3.1.179   RXN1G-424      Evidence for 3-cis,cis-delta15,27-3-hydroxyC46:2-[acyl-carrier protein] dehydratase   RXN1G-554 
  

 

 
 Hit 
 

 
 Common 
 

 
 P 
 

 
 #Q 
 

 
 Best Qry 
 

 
 Best Eval 
 

 
 Avg Rank 
 

 
 Aln Len 
 

 
 Qry Len 
 

 
 IDOP? 
 

 
 adj? 
 

 

 

 
GKFG-904-MONOMER
 

 
pfr:PFREUD_02450 maoC; Ma
 

 
0.0051016076
 

 
1.0
 

 
tr|P96927|P96927_MYCTU
 

 
0.00452189
 

 
1.0
 

 
0.6126761
 

 
142
 

 
T
 

 
NIL
 

 

 

 
GKFG-1225-MONOMER
 

 
mcu:HMPREF0573_11305 hypo
 

 
9.2874106e-4
 

 
1.0
 

 
tr|P96927|P96927_MYCTU
 

 
0.879797
 

 
2.0
 

 
0.64788735
 

 
142
 

 
T
 

 
NIL
 

 

 

 
GKFG-1026-MONOMER
 

 
hypothetical protein (db=
 

 
1.5421621e-5
 

 
1.0
 

 
P96928
 

 
0.571235
 

 
1.0
 

 
0.21686748
 

 
166
 

 
T
 

 
NIL
 

 

 
     Evidence for trans-delta2-cis,cis-delta15,27-C46:3-[acyl-carrier protein] reductase (NADPH, B-specific), EC# 1.3.1.10   RXN1G-607      Evidence for cis,cis-delta17,29-3-oxo-C48:2-[acyl-carrier protein] synthase, EC# 2.3.1.179   RXN1G-660      Evidence for 3-cis,cis-delta17,29-3-hydroxyC48:2-[acyl-carrier protein] dehydratase   RXN1G-760 
  

 

 
 Hit 
 

 
 Common 
 

 
 P 
 

 
 #Q 
 

 
 Best Qry 
 

 
 Best Eval 
 

 
 Avg Rank 
 

 
 Aln Len 
 

 
 Qry Len 
 

 
 IDOP? 
 

 
 adj? 
 

 

 

 
GKFG-904-MONOMER
 

 
pfr:PFREUD_02450 maoC; Ma
 

 
0.0051016076
 

 
1.0
 

 
tr|P96927|P96927_MYCTU
 

 
0.00452189
 

 
1.0
 

 
0.6126761
 

 
142
 

 
T
 

 
NIL
 

 

 

 
GKFG-1225-MONOMER
 

 
mcu:HMPREF0573_11305 hypo
 

 
9.2874106e-4
 

 
1.0
 

 
tr|P96927|P96927_MYCTU
 

 
0.879797
 

 
2.0
 

 
0.64788735
 

 
142
 

 
T
 

 
NIL
 

 

 

 
GKFG-1026-MONOMER
 

 
hypothetical protein (db=
 

 
1.5421621e-5
 

 
1.0
 

 
P96928
 

 
0.571235
 

 
1.0
 

 
0.21686748
 

 
166
 

 
T
 

 
NIL
 

 

 
     Evidence for trans-delta2-cis,cis-delta17,29-C48:3-[acyl-carrier protein] reductase (NADPH, B-specific), EC# 1.3.1.10   RXN1G-820      Evidence for cis,cis-delta19,31-3-oxo-C50:2-[acyl-carrier protein] synthase, EC# 2.3.1.179   RXN1G-883      Evidence for 3-cis,cis-delta19,31-3-hydroxyC50:2-[acyl-carrier protein] dehydratase   RXN1G-1004 
  

 

 
 Hit 
 

 
 Common 
 

 
 P 
 

 
 #Q 
 

 
 Best Qry 
 

 
 Best Eval 
 

 
 Avg Rank 
 

 
 Aln Len 
 

 
 Qry Len 
 

 
 IDOP? 
 

 
 adj? 
 

 

 

 
GKFG-904-MONOMER
 

 
pfr:PFREUD_02450 maoC; Ma
 

 
0.0051016076
 

 
1.0
 

 
tr|P96927|P96927_MYCTU
 

 
0.00452189
 

 
1.0
 

 
0.6126761
 

 
142
 

 
T
 

 
NIL
 

 

 

 
GKFG-1225-MONOMER
 

 
mcu:HMPREF0573_11305 hypo
 

 
9.2874106e-4
 

 
1.0
 

 
tr|P96927|P96927_MYCTU
 

 
0.879797
 

 
2.0
 

 
0.64788735
 

 
142
 

 
T
 

 
NIL
 

 

 

 
GKFG-1026-MONOMER
 

 
hypothetical protein (db=
 

 
1.5421621e-5
 

 
1.0
 

 
P96928
 

 
0.571235
 

 
1.0
 

 
0.21686748
 

 
166
 

 
T
 

 
NIL
 

 

 
     Evidence for trans-delta2-cis,cis-delta19,31-C50:3-[acyl-carrier protein] reductase (NADPH, B-specific), EC# 1.3.1.10   RXN1G-1057      Evidence for cis,cis-delta5,23-3-oxo-C42:2-[acyl-carrier protein] synthase, EC# 2.3.1.179   RXN1G-79      Evidence for cis,cis-delta5,23-3-hydroxyC42:2-[acyl-carrier protein] dehydratase   RXN1G-214 
  

 

 
 Hit 
 

 
 Common 
 

 
 P 
 

 
 #Q 
 

 
 Best Qry 
 

 
 Best Eval 
 

 
 Avg Rank 
 

 
 Aln Len 
 

 
 Qry Len 
 

 
 IDOP? 
 

 
 adj? 
 

 

 

 
GKFG-904-MONOMER
 

 
pfr:PFREUD_02450 maoC; Ma
 

 
0.0051016076
 

 
1.0
 

 
tr|P96927|P96927_MYCTU
 

 
0.00452189
 

 
1.0
 

 
0.6126761
 

 
142
 

 
T
 

 
NIL
 

 

 

 
GKFG-1225-MONOMER
 

 
mcu:HMPREF0573_11305 hypo
 

 
9.2874106e-4
 

 
1.0
 

 
tr|P96927|P96927_MYCTU
 

 
0.879797
 

 
2.0
 

 
0.64788735
 

 
142
 

 
T
 

 
NIL
 

 

 
     Evidence for trans-delta2-cis,cis-delta5,23-C42:3-[acyl-carrier protein] reductase (NADPH, B-specific), EC# 1.3.1.10   RXN1G-248      Evidence for cis,cis-delta7,25-3-oxo-C44:2-[acyl-carrier protein] synthase, EC# 2.3.1.179   RXN1G-334      Evidence for 3-cis,cis-delta7,25-3-hydroxyC44:2-[acyl-carrier protein] dehydratase   RXN1G-425 
  

 

 
 Hit 
 

 
 Common 
 

 
 P 
 

 
 #Q 
 

 
 Best Qry 
 

 
 Best Eval 
 

 
 Avg Rank 
 

 
 Aln Len 
 

 
 Qry Len 
 

 
 IDOP? 
 

 
 adj? 
 

 

 

 
GKFG-904-MONOMER
 

 
pfr:PFREUD_02450 maoC; Ma
 

 
0.0051016076
 

 
1.0
 

 
tr|P96927|P96927_MYCTU
 

 
0.00452189
 

 
1.0
 

 
0.6126761
 

 
142
 

 
T
 

 
NIL
 

 

 

 
GKFG-1225-MONOMER
 

 
mcu:HMPREF0573_11305 hypo
 

 
9.2874106e-4
 

 
1.0
 

 
tr|P96927|P96927_MYCTU
 

 
0.879797
 

 
2.0
 

 
0.64788735
 

 
142
 

 
T
 

 
NIL
 

 

 

 
GKFG-1026-MONOMER
 

 
hypothetical protein (db=
 

 
1.5421621e-5
 

 
1.0
 

 
P96928
 

 
0.571235
 

 
1.0
 

 
0.21686748
 

 
166
 

 
T
 

 
NIL
 

 

 
     Evidence for trans-delta2-cis,cis-delta7,25-C44:3-[acyl-carrier protein] reductase (NADPH, B-specific), EC# 1.3.1.10   RXN1G-468      Evidence for cis,cis-delta9,27-3-oxo-C46:2-[acyl-carrier protein] synthase, EC# 2.3.1.179   RXN1G-557      Evidence for 3-cis,cis-delta9,27-3-hydroxyC46:2-[acyl-carrier protein] dehydratase   RXN1G-645 
  

 

 
 Hit 
 

 
 Common 
 

 
 P 
 

 
 #Q 
 

 
 Best Qry 
 

 
 Best Eval 
 

 
 Avg Rank 
 

 
 Aln Len 
 

 
 Qry Len 
 

 
 IDOP? 
 

 
 adj? 
 

 

 

 
GKFG-904-MONOMER
 

 
pfr:PFREUD_02450 maoC; Ma
 

 
0.0051016076
 

 
1.0
 

 
tr|P96927|P96927_MYCTU
 

 
0.00452189
 

 
1.0
 

 
0.6126761
 

 
142
 

 
T
 

 
NIL
 

 

 

 
GKFG-1225-MONOMER
 

 
mcu:HMPREF0573_11305 hypo
 

 
9.2874106e-4
 

 
1.0
 

 
tr|P96927|P96927_MYCTU
 

 
0.879797
 

 
2.0
 

 
0.64788735
 

 
142
 

 
T
 

 
NIL
 

 

 

 
GKFG-1026-MONOMER
 

 
hypothetical protein (db=
 

 
1.5421621e-5
 

 
1.0
 

 
P96928
 

 
0.571235
 

 
1.0
 

 
0.21686748
 

 
166
 

 
T
 

 
NIL
 

 

 
     Evidence for trans-delta2-cis,cis-delta9,27-C46:3-[acyl-carrier protein] reductase (NADPH, B-specific), EC# 1.3.1.10   RXN1G-686      Evidence for cis,cis-delta11,29-3-oxo-C48:2-[acyl-carrier protein] synthase, EC# 2.3.1.179   RXN1G-818      Evidence for cis,cis-delta11,29-3-hydroxyC48:2-[acyl-carrier protein] dehydratase   RXN1G-884 
  

 

 
 Hit 
 

 
 Common 
 

 
 P 
 

 
 #Q 
 

 
 Best Qry 
 

 
 Best Eval 
 

 
 Avg Rank 
 

 
 Aln Len 
 

 
 Qry Len 
 

 
 IDOP? 
 

 
 adj? 
 

 

 

 
GKFG-904-MONOMER
 

 
pfr:PFREUD_02450 maoC; Ma
 

 
0.0051016076
 

 
1.0
 

 
tr|P96927|P96927_MYCTU
 

 
0.00452189
 

 
1.0
 

 
0.6126761
 

 
142
 

 
T
 

 
NIL
 

 

 

 
GKFG-1225-MONOMER
 

 
mcu:HMPREF0573_11305 hypo
 

 
9.2874106e-4
 

 
1.0
 

 
tr|P96927|P96927_MYCTU
 

 
0.879797
 

 
2.0
 

 
0.64788735
 

 
142
 

 
T
 

 
NIL
 

 

 

 
GKFG-1026-MONOMER
 

 
hypothetical protein (db=
 

 
1.5421621e-5
 

 
1.0
 

 
P96928
 

 
0.571235
 

 
1.0
 

 
0.21686748
 

 
166
 

 
T
 

 
NIL
 

 

 
     Evidence for trans-delta2-cis,cis-delta11,29-C48:3-[acyl-carrier protein] reductase (NADPH, B-specific), EC# 1.3.1.10   RXN1G-915      Evidence for cis,cis-delta13,31-3-oxo-C50:2-[acyl-carrier protein] synthase, EC# 2.3.1.179   RXN1G-1015      Evidence for cis,cis-delta13,31-3-hydroxyC50:2-[acyl-carrier protein] dehydratase   RXN1G-1081 
  

 

 
 Hit 
 

 
 Common 
 

 
 P 
 

 
 #Q 
 

 
 Best Qry 
 

 
 Best Eval 
 

 
 Avg Rank 
 

 
 Aln Len 
 

 
 Qry Len 
 

 
 IDOP? 
 

 
 adj? 
 

 

 

 
GKFG-904-MONOMER
 

 
pfr:PFREUD_02450 maoC; Ma
 

 
0.0051016076
 

 
1.0
 

 
tr|P96927|P96927_MYCTU
 

 
0.00452189
 

 
1.0
 

 
0.6126761
 

 
142
 

 
T
 

 
NIL
 

 

 

 
GKFG-1225-MONOMER
 

 
mcu:HMPREF0573_11305 hypo
 

 
9.2874106e-4
 

 
1.0
 

 
tr|P96927|P96927_MYCTU
 

 
0.879797
 

 
2.0
 

 
0.64788735
 

 
142
 

 
T
 

 
NIL
 

 

 

 
GKFG-1026-MONOMER
 

 
hypothetical protein (db=
 

 
1.5421621e-5
 

 
1.0
 

 
P96928
 

 
0.571235
 

 
1.0
 

 
0.21686748
 

 
166
 

 
T
 

 
NIL
 

 

 
     Evidence for trans-delta2-cis,cis-delta13,31-C50:3-[acyl-carrier protein] reductase (NADPH, B-specific), EC# 1.3.1.10   RXN1G-1117      Evidence for cis,cis-delta15,33-3-oxo-C52:2-[acyl-carrier protein] synthase, EC# 2.3.1.179   RXN1G-1212      Evidence for cis,cis-delta15,33-3-hydroxyC52:2-[acyl-carrier protein] dehydratase   RXN1G-1278 
  

 

 
 Hit 
 

 
 Common 
 

 
 P 
 

 
 #Q 
 

 
 Best Qry 
 

 
 Best Eval 
 

 
 Avg Rank 
 

 
 Aln Len 
 

 
 Qry Len 
 

 
 IDOP? 
 

 
 adj? 
 

 

 

 
GKFG-904-MONOMER
 

 
pfr:PFREUD_02450 maoC; Ma
 

 
0.0051016076
 

 
1.0
 

 
tr|P96927|P96927_MYCTU
 

 
0.00452189
 

 
1.0
 

 
0.6126761
 

 
142
 

 
T
 

 
NIL
 

 

 

 
GKFG-1225-MONOMER
 

 
mcu:HMPREF0573_11305 hypo
 

 
9.2874106e-4
 

 
1.0
 

 
tr|P96927|P96927_MYCTU
 

 
0.879797
 

 
2.0
 

 
0.64788735
 

 
142
 

 
T
 

 
NIL
 

 

 

 
GKFG-1026-MONOMER
 

 
hypothetical protein (db=
 

 
1.5421621e-5
 

 
1.0
 

 
P96928
 

 
0.571235
 

 
1.0
 

 
0.21686748
 

 
166
 

 
T
 

 
NIL
 

 

 
     Evidence for trans-delta2-cis,cis-delta15,33-C52:3-[acyl-carrier protein] reductase (NADPH, B-specific), EC# 1.3.1.10   RXN1G-1325      Evidence for cis,cis-delta17,35-3-oxo-C54:2-[acyl-carrier protein] synthase, EC# 2.3.1.179   RXN1G-94      Evidence for cis,cis-delta17,35-3-hydroxyC54:2-[acyl-carrier protein] dehydratase   RXN1G-220 
  

 

 
 Hit 
 

 
 Common 
 

 
 P 
 

 
 #Q 
 

 
 Best Qry 
 

 
 Best Eval 
 

 
 Avg Rank 
 

 
 Aln Len 
 

 
 Qry Len 
 

 
 IDOP? 
 

 
 adj? 
 

 

 

 
GKFG-904-MONOMER
 

 
pfr:PFREUD_02450 maoC; Ma
 

 
0.0051016076
 

 
1.0
 

 
tr|P96927|P96927_MYCTU
 

 
0.00452189
 

 
1.0
 

 
0.6126761
 

 
142
 

 
T
 

 
NIL
 

 

 

 
GKFG-1225-MONOMER
 

 
mcu:HMPREF0573_11305 hypo
 

 
9.2874106e-4
 

 
1.0
 

 
tr|P96927|P96927_MYCTU
 

 
0.879797
 

 
2.0
 

 
0.64788735
 

 
142
 

 
T
 

 
NIL
 

 

 

 
GKFG-1026-MONOMER
 

 
hypothetical protein (db=
 

 
1.5421621e-5
 

 
1.0
 

 
P96928
 

 
0.571235
 

 
1.0
 

 
0.21686748
 

 
166
 

 
T
 

 
NIL
 

 

 
     Evidence for trans-delta2-cis,cis-delta17,35-C54:3-[acyl-carrier protein] reductase (NADPH, B-specific), EC# 1.3.1.10   RXN1G-276      Evidence for cis,cis-delta19,37-3-oxo-C56:2-[acyl-carrier protein] synthase, EC# 2.3.1.179   RXN1G-374      Evidence for 3-cis,cis-delta19,37-3-hydroxyC56:2-[acyl-carrier protein] dehydratase   RXN1G-440 
  

 

 
 Hit 
 

 
 Common 
 

 
 P 
 

 
 #Q 
 

 
 Best Qry 
 

 
 Best Eval 
 

 
 Avg Rank 
 

 
 Aln Len 
 

 
 Qry Len 
 

 
 IDOP? 
 

 
 adj? 
 

 

 

 
GKFG-904-MONOMER
 

 
pfr:PFREUD_02450 maoC; Ma
 

 
0.0051016076
 

 
1.0
 

 
tr|P96927|P96927_MYCTU
 

 
0.00452189
 

 
1.0
 

 
0.6126761
 

 
142
 

 
T
 

 
NIL
 

 

 

 
GKFG-1225-MONOMER
 

 
mcu:HMPREF0573_11305 hypo
 

 
9.2874106e-4
 

 
1.0
 

 
tr|P96927|P96927_MYCTU
 

 
0.879797
 

 
2.0
 

 
0.64788735
 

 
142
 

 
T
 

 
NIL
 

 

 

 
GKFG-1026-MONOMER
 

 
hypothetical protein (db=
 

 
1.5421621e-5
 

 
1.0
 

 
P96928
 

 
0.571235
 

 
1.0
 

 
0.21686748
 

 
166
 

 
T
 

 
NIL
 

 

 
     Evidence for trans-delta2-cis,cis-delta19,37-C56:3-[acyl-carrier protein] reductase (NADPH, B-specific), EC# 1.3.1.10   RXN1G-471      Evidence for cis,cis-delta21,39-3-oxo-C58:2-[acyl-carrier protein] synthase, EC# 2.3.1.179   RXN1G-582      Evidence for 3-cis-delta21,39-3-hydroxyC58:2-[acyl-carrier protein] dehydratase   RXN1G-648 
  

 

 
 Hit 
 

 
 Common 
 

 
 P 
 

 
 #Q 
 

 
 Best Qry 
 

 
 Best Eval 
 

 
 Avg Rank 
 

 
 Aln Len 
 

 
 Qry Len 
 

 
 IDOP? 
 

 
 adj? 
 

 

 

 
GKFG-904-MONOMER
 

 
pfr:PFREUD_02450 maoC; Ma
 

 
0.0051016076
 

 
1.0
 

 
tr|P96927|P96927_MYCTU
 

 
0.00452189
 

 
1.0
 

 
0.6126761
 

 
142
 

 
T
 

 
NIL
 

 

 

 
GKFG-1225-MONOMER
 

 
mcu:HMPREF0573_11305 hypo
 

 
9.2874106e-4
 

 
1.0
 

 
tr|P96927|P96927_MYCTU
 

 
0.879797
 

 
2.0
 

 
0.64788735
 

 
142
 

 
T
 

 
NIL
 

 

 

 
GKFG-1026-MONOMER
 

 
hypothetical protein (db=
 

 
1.5421621e-5
 

 
1.0
 

 
P96928
 

 
0.571235
 

 
1.0
 

 
0.21686748
 

 
166
 

 
T
 

 
NIL
 

 

 
     Evidence for trans-delta2-cis,cis-delta21,39-C58:3-[acyl-carrier protein] reductase (NADPH, B-specific), EC# 1.3.1.10   RXN1G-679      Evidence for cis,cis-&Delta;19,31-C50:2-[acp] reductase, EC# 2.1.1.-   RXN1G-45 
  
no BLAST hits found
 
     Evidence for cis,cis-&Delta;19-37-C56:2-[acp] reductase, EC# 2.1.1.-   RXN1G-2527      Evidence for cis-&Delta;19-37-hydroxy-38-methyl-C57:1-[acp] reductase, EC# 2.1.1.-   RXN1G-2544 
  
no BLAST hits found
 
     Evidence for cis-&Delta;19-37-hydroxy-38-methyl-C57:1-[acp] reductase, EC# 2.1.1.-   RXN1G-3232      Evidence for trans-&Delta;18-37-hydroxy-38-methyl-C58:1-[acp] reductase, EC# 2.1.1.79   RXN1G-3256 
  
no BLAST hits found
 
     Evidence for cis,cis-&Delta;21,39-C58:2-[acp] reductase, EC# 2.1.1.-   RXN1G-3613      Evidence for cis-&Delta;21-39-hydroxy-40-methyl-C59:1-[acp] reductase, EC# 2.1.1.-   RXN1G-3641 
  

 

 
 Hit 
 

 
 Common 
 

 
 P 
 

 
 #Q 
 

 
 Best Qry 
 

 
 Best Eval 
 

 
 Avg Rank 
 

 
 Aln Len 
 

 
 Qry Len 
 

 
 IDOP? 
 

 
 adj? 
 

 

 

 
GKFG-151-MONOMER
 

 
cga:Celgi_0340 FAD linked
 

 
0.9407177
 

 
1.0
 

 
O07406
 

 
2.78256e-21
 

 
1.0
 

 
1.0
 

 
449
 

 
NIL
 

 
NIL
 

 

 
     Evidence for cis-delta21-39-hydroxy-40-methyl-C59:1-[acp] synthase, EC# 2.1.1.-   RXN1G-3660      Evidence for trans-&Delta;20-39-hydroxy-40-methyl-C60:1-[acp] reductase, EC# 2.1.1.-   RXN1G-3667 
  

 

 
 Hit 
 

 
 Common 
 

 
 P 
 

 
 #Q 
 

 
 Best Qry 
 

 
 Best Eval 
 

 
 Avg Rank 
 

 
 Aln Len 
 

 
 Qry Len 
 

 
 IDOP? 
 

 
 adj? 
 

 

 

 
GKFG-151-MONOMER
 

 
cga:Celgi_0340 FAD linked
 

 
0.9407177
 

 
1.0
 

 
O07406
 

 
2.78256e-21
 

 
1.0
 

 
1.0
 

 
449
 

 
NIL
 

 
NIL
 

 

 
     Evidence for C52-&alpha;-meroacyl-[acp] synthetase, EC# 2.3.1.86   RXN1G-3993 
  

 

 
 Hit 
 

 
 Common 
 

 
 P 
 

 
 #Q 
 

 
 Best Qry 
 

 
 Best Eval 
 

 
 Avg Rank 
 

 
 Aln Len 
 

 
 Qry Len 
 

 
 IDOP? 
 

 
 adj? 
 

 

 

 
GKFG-714-MONOMER
 

 
bpo:BP951000_0640 putativ
 

 
0.36918753
 

 
1.0
 

 
O53580
 

 
3.33975e-11
 

 
1.0
 

 
0.6923077
 

 
637
 

 
T
 

 
NIL
 

 

 

 
GKFG-905-MONOMER
 

 
pfr:PFREUD_02440 caiC; cr
 

 
7.0298207e-4
 

 
1.0
 

 
O53580
 

 
6.694e-4
 

 
3.0
 

 
0.7864992
 

 
637
 

 
T
 

 
NIL
 

 

 

 
GKFG-1677-MONOMER
 

 
cfl:Cfla_1398 AMP-depende
 

 
2.5307965e-5
 

 
1.0
 

 
O53580
 

 
1.95768e-6
 

 
2.0
 

 
0.30141288
 

 
637
 

 
T
 

 
NIL
 

 

 
     Evidence for cis-methoxy-C59-meroacyl-[acp] synthetase, EC# 2.3.1.86   RXN1G-4140 
  

 

 
 Hit 
 

 
 Common 
 

 
 P 
 

 
 #Q 
 

 
 Best Qry 
 

 
 Best Eval 
 

 
 Avg Rank 
 

 
 Aln Len 
 

 
 Qry Len 
 

 
 IDOP? 
 

 
 adj? 
 

 

 

 
GKFG-714-MONOMER
 

 
bpo:BP951000_0640 putativ
 

 
0.36918753
 

 
1.0
 

 
O53580
 

 
3.33975e-11
 

 
1.0
 

 
0.6923077
 

 
637
 

 
T
 

 
NIL
 

 

 

 
GKFG-905-MONOMER
 

 
pfr:PFREUD_02440 caiC; cr
 

 
7.0298207e-4
 

 
1.0
 

 
O53580
 

 
6.694e-4
 

 
3.0
 

 
0.7864992
 

 
637
 

 
T
 

 
NIL
 

 

 

 
GKFG-1677-MONOMER
 

 
cfl:Cfla_1398 AMP-depende
 

 
2.5307965e-5
 

 
1.0
 

 
O53580
 

 
1.95768e-6
 

 
2.0
 

 
0.30141288
 

 
637
 

 
T
 

 
NIL
 

 

 
     Evidence for trans-methoxy-C60-meroacyl-[acp] synthetase, EC# 2.3.1.86   RXN1G-4141 
  

 

 
 Hit 
 

 
 Common 
 

 
 P 
 

 
 #Q 
 

 
 Best Qry 
 

 
 Best Eval 
 

 
 Avg Rank 
 

 
 Aln Len 
 

 
 Qry Len 
 

 
 IDOP? 
 

 
 adj? 
 

 

 

 
GKFG-714-MONOMER
 

 
bpo:BP951000_0640 putativ
 

 
0.36918753
 

 
1.0
 

 
O53580
 

 
3.33975e-11
 

 
1.0
 

 
0.6923077
 

 
637
 

 
T
 

 
NIL
 

 

 

 
GKFG-905-MONOMER
 

 
pfr:PFREUD_02440 caiC; cr
 

 
7.0298207e-4
 

 
1.0
 

 
O53580
 

 
6.694e-4
 

 
3.0
 

 
0.7864992
 

 
637
 

 
T
 

 
NIL
 

 

 

 
GKFG-1677-MONOMER
 

 
cfl:Cfla_1398 AMP-depende
 

 
2.5307965e-5
 

 
1.0
 

 
O53580
 

 
1.95768e-6
 

 
2.0
 

 
0.30141288
 

 
637
 

 
T
 

 
NIL
 

 

 
     Evidence for cis-keto-C60-meroacyl-[acp] synthetase, EC# 2.3.1.86   RXN1G-4142 
  

 

 
 Hit 
 

 
 Common 
 

 
 P 
 

 
 #Q 
 

 
 Best Qry 
 

 
 Best Eval 
 

 
 Avg Rank 
 

 
 Aln Len 
 

 
 Qry Len 
 

 
 IDOP? 
 

 
 adj? 
 

 

 

 
GKFG-714-MONOMER
 

 
bpo:BP951000_0640 putativ
 

 
0.36918753
 

 
1.0
 

 
O53580
 

 
3.33975e-11
 

 
1.0
 

 
0.6923077
 

 
637
 

 
T
 

 
NIL
 

 

 

 
GKFG-905-MONOMER
 

 
pfr:PFREUD_02440 caiC; cr
 

 
7.0298207e-4
 

 
1.0
 

 
O53580
 

 
6.694e-4
 

 
3.0
 

 
0.7864992
 

 
637
 

 
T
 

 
NIL
 

 

 

 
GKFG-1677-MONOMER
 

 
cfl:Cfla_1398 AMP-depende
 

 
2.5307965e-5
 

 
1.0
 

 
O53580
 

 
1.95768e-6
 

 
2.0
 

 
0.30141288
 

 
637
 

 
T
 

 
NIL
 

 

 
     Evidence for trans-keto-C61-meroacyl-[acp] synthetase, EC# 2.3.1.86   RXN1G-4143 
  

 

 
 Hit 
 

 
 Common 
 

 
 P 
 

 
 #Q 
 

 
 Best Qry 
 

 
 Best Eval 
 

 
 Avg Rank 
 

 
 Aln Len 
 

 
 Qry Len 
 

 
 IDOP? 
 

 
 adj? 
 

 

 

 
GKFG-714-MONOMER
 

 
bpo:BP951000_0640 putativ
 

 
0.36918753
 

 
1.0
 

 
O53580
 

 
3.33975e-11
 

 
1.0
 

 
0.6923077
 

 
637
 

 
T
 

 
NIL
 

 

 

 
GKFG-905-MONOMER
 

 
pfr:PFREUD_02440 caiC; cr
 

 
7.0298207e-4
 

 
1.0
 

 
O53580
 

 
6.694e-4
 

 
3.0
 

 
0.7864992
 

 
637
 

 
T
 

 
NIL
 

 

 

 
GKFG-1677-MONOMER
 

 
cfl:Cfla_1398 AMP-depende
 

 
2.5307965e-5
 

 
1.0
 

 
O53580
 

 
1.95768e-6
 

 
2.0
 

 
0.30141288
 

 
637
 

 
T
 

 
NIL
 

 

 
     Evidence for cerotoyl-CoA carboxylase, EC# 6.4.1.3   RXN1G-4355 
  

 

 
 Hit 
 

 
 Common 
 

 
 P 
 

 
 #Q 
 

 
 Best Qry 
 

 
 Best Eval 
 

 
 Avg Rank 
 

 
 Aln Len 
 

 
 Qry Len 
 

 
 IDOP? 
 

 
 adj? 
 

 

 

 
GKFG-471-MONOMER
 

 
11974..12138 - ( gc_cont=
 

 
3.787886e-5
 

 
1.0
 

 
P96885
 

 
0.3874
 

 
1.0
 

 
0.09854015
 

 
548
 

 
NIL
 

 
NIL
 

 

 
     Evidence for &alpha;-meroacyl-AMP transferase, EC# 2.1.1.-   RXN1G-181 
  
no BLAST hits found
 
     Evidence for cis-methoxy-meroacyl-AMP transferase, EC# 2.1.1.-   RXN1G-98 
  
no BLAST hits found
 
     Evidence for trans-keto-meroacyl-AMP transferase, EC# 2.1.1.-   RXN1G-297 
  
no BLAST hits found
 
     Evidence for trans-methoxy-meroacyl-AMP transferase, EC# 2.1.1.-   RXN1G-295 
  
no BLAST hits found
 
     Evidence for cis-keto-meroacyl-AMP transferase, EC# 2.1.1.-   RXN1G-296 
  
no BLAST hits found
 
     Evidence for &alpha;-meroacyl transferase, EC# 2.1.1.-   RXN1G-576 
  
no BLAST hits found
 
     Evidence for  cis -methoxy-meroacyl transferase, EC# 2.1.1.-   RXN1G-697 
  
no BLAST hits found
 
     Evidence for  trans -keto-meroacyl transferase, EC# 2.1.1.-   RXN1G-867 
  
no BLAST hits found
 
     Evidence for  trans -methoxy-meroacyl transferase, EC# 2.1.1.-   RXN1G-865 
  
no BLAST hits found
 
     Evidence for cis-keto-meroacyl-transferase, EC# 2.1.1.-   RXN1G-866 
  
no BLAST hits found
 
     Evidence for 3-oxo-C78-&alpha;-mycolate synthase, EC# 2.1.1.-   RXN1G-886 
  
no BLAST hits found
 
     Evidence for 3-oxo-C85-cis-methoxy-mycolate-synthase, EC# 2.1.1.-   RXN1G-887 
  
no BLAST hits found
 
     Evidence for 3-oxo-C86-trans-methoxy-mycolate-synthase, EC# 2.1.1.-   RXN1G-888 
  
no BLAST hits found
 
     Evidence for 3-oxo-C86-cis-keto-mycolate synthase, EC# 2.1.1.-   RXN1G-890 
  
no BLAST hits found
 
     Evidence for 3-oxo-C87-trans-mycolate synthase, EC# 2.1.1.-   RXN1G-891 
  
no BLAST hits found
 
     Evidence for 3-oxo-C78-&alpha;-mycolate-reductase, EC# 1.-.-.-   RXN1G-1486 
  
no BLAST hits found
 
     Evidence for 3-oxo-C85-cis-methoxy-mycolate reductase, EC# 1.-.-.-   RXN1G-1527 
  
no BLAST hits found
 
     Evidence for 3-oxo-C86-trans-methoxy-mycolate-reductase, EC# 1.-.-.-   RXN1G-1528 
  
no BLAST hits found
 
     Evidence for 3-oxo-C86-cis-keto-mycolate-reductase, EC# 1.-.-.-   RXN1G-1529 
  
no BLAST hits found
 
     Evidence for 3-oxo-C87-trans-keto-mycolate reductase, EC# 1.-.-.-   RXN1G-1530 
  
no BLAST hits found
 
     Evidence for C78-&alpha;-mycolate-O-mycolyltransferase   RXN1G-288      Evidence for C85-cis-mycolate O-mycolyltransferase   RXN1G-289      Evidence for C87-trans-mycolate O-mycolyltransferase   RXN1G-290      Evidence for C86-cis-keto-mycolate O-mycolyltransferase   RXN1G-291      Evidence for C87-trans-keto-mycolate O-mycolyltransferase   RXN1G-292      Evidence for 6-O-mycolyl-trehalose O-mycolyltransferase   RXN1G-807      Evidence for 6-O-cis-methoxy-mycolyl-trehalose O-mycolyltransferase   RXN1G-808      Evidence for 6-O-trans-methoxy-mycolyl-trehalose O-mycolyltransferase   RXN1G-809      Evidence for 6-O-cis-keto-mycolyl-trehalose O-mycolyltransferase   RXN1G-810      Evidence for 6-O-trans-keto-mycolyl-trehalose O-mycolyltransferase   RXN1G-811      Evidence for 6-O-mycolyl-trehalose-6-phosphatase, EC# 3.1.3.12   RXN1G-1435      Evidence for 6-O-cis-methoxy-mycolyl-trehalose-6-phosphatase, EC# 3.1.3.12   RXN1G-1436      Evidence for 6-O-trans-methoxy-mycolyl-trehalose-6-phosphatase, EC# 3.1.3.12   RXN1G-1437      Evidence for 6-O-cis-keto-mycolyl-trehalose-6-phosphatase, EC# 3.1.3.12   RXN1G-1438      Evidence for 6-O-trans-keto-mycolyl-trehalose-6-phosphatase, EC# 3.1.3.12   RXN1G-1439      Evidence for trehalose-mono-mycolate transport, EC# 3.6.3.-   RXN1G-137      Evidence for trehalose-cis-methoxy-mono-mycolate transport, EC# 3.6.3.-   RXN1G-470      Evidence for trehalose-trans-methoxy-mono-mycolate transport, EC# 3.6.3.-   RXN1G-472      Evidence for trehalose-cis-keto-mono-mycolate transport, EC# 3.6.3.-   RXN1G-473      Evidence for trehalose-trans-keto-mono-mycolate transport, EC# 3.6.3.-   RXN1G-474      Evidence for trehalose  O -mycolyltransferase, EC# 2.3.1.122   RXN1G-874 
  

 

 
 Hit 
 

 
 Common 
 

 
 P 
 

 
 #Q 
 

 
 Best Qry 
 

 
 Best Eval 
 

 
 Avg Rank 
 

 
 Aln Len 
 

 
 Qry Len 
 

 
 IDOP? 
 

 
 adj? 
 

 

 

 
GKFG-653-MONOMER
 

 
bcv:Bcav_2498 ispG; 4-hyd
 

 
3.182795e-4
 

 
1.0
 

 
P0A4V2
 

 
0.0388288
 

 
1.0
 

 
0.36094674
 

 
338
 

 
T
 

 
NIL
 

 

 
     Evidence for trehalose-cis-methoxy-mono-mycolate O-mycolyltransferase, EC# 2.3.1.-   RXN1G-1149 
  

 

 
 Hit 
 

 
 Common 
 

 
 P 
 

 
 #Q 
 

 
 Best Qry 
 

 
 Best Eval 
 

 
 Avg Rank 
 

 
 Aln Len 
 

 
 Qry Len 
 

 
 IDOP? 
 

 
 adj? 
 

 

 

 
GKFG-653-MONOMER
 

 
bcv:Bcav_2498 ispG; 4-hyd
 

 
3.182795e-4
 

 
1.0
 

 
P0A4V2
 

 
0.0388288
 

 
1.0
 

 
0.36094674
 

 
338
 

 
T
 

 
NIL
 

 

 
     Evidence for trehalose-trans-methoxy-mono-mycolate O-mycolyltransferase, EC# 2.3.1.-   RXN1G-1150 
  

 

 
 Hit 
 

 
 Common 
 

 
 P 
 

 
 #Q 
 

 
 Best Qry 
 

 
 Best Eval 
 

 
 Avg Rank 
 

 
 Aln Len 
 

 
 Qry Len 
 

 
 IDOP? 
 

 
 adj? 
 

 

 

 
GKFG-653-MONOMER
 

 
bcv:Bcav_2498 ispG; 4-hyd
 

 
3.182795e-4
 

 
1.0
 

 
P0A4V2
 

 
0.0388288
 

 
1.0
 

 
0.36094674
 

 
338
 

 
T
 

 
NIL
 

 

 
     Evidence for trehalose-cis-keto-mono-mycolate O-mycolyltransferase, EC# 2.3.1.-   RXN1G-1151 
  

 

 
 Hit 
 

 
 Common 
 

 
 P 
 

 
 #Q 
 

 
 Best Qry 
 

 
 Best Eval 
 

 
 Avg Rank 
 

 
 Aln Len 
 

 
 Qry Len 
 

 
 IDOP? 
 

 
 adj? 
 

 

 

 
GKFG-653-MONOMER
 

 
bcv:Bcav_2498 ispG; 4-hyd
 

 
3.182795e-4
 

 
1.0
 

 
P0A4V2
 

 
0.0388288
 

 
1.0
 

 
0.36094674
 

 
338
 

 
T
 

 
NIL
 

 

 
     Evidence for trehalose-trans-keto-mono-mycolate O-mycolyltransferase, EC# 2.3.1.-   RXN1G-1152 
  

 

 
 Hit 
 

 
 Common 
 

 
 P 
 

 
 #Q 
 

 
 Best Qry 
 

 
 Best Eval 
 

 
 Avg Rank 
 

 
 Aln Len 
 

 
 Qry Len 
 

 
 IDOP? 
 

 
 adj? 
 

 

 

 
GKFG-653-MONOMER
 

 
bcv:Bcav_2498 ispG; 4-hyd
 

 
3.182795e-4
 

 
1.0
 

 
P0A4V2
 

 
0.0388288
 

 
1.0
 

 
0.36094674
 

 
338
 

 
T
 

 
NIL
 

 

 
     Evidence for trehalose-mono-mycolate O-mycolyltransferase, EC# 2.3.1.-   RXN1G-1633 
  

 

 
 Hit 
 

 
 Common 
 

 
 P 
 

 
 #Q 
 

 
 Best Qry 
 

 
 Best Eval 
 

 
 Avg Rank 
 

 
 Aln Len 
 

 
 Qry Len 
 

 
 IDOP? 
 

 
 adj? 
 

 

 

 
GKFG-653-MONOMER
 

 
bcv:Bcav_2498 ispG; 4-hyd
 

 
3.182795e-4
 

 
1.0
 

 
P0A4V2
 

 
0.0388288
 

 
1.0
 

 
0.36094674
 

 
338
 

 
T
 

 
NIL
 

 

 
     Evidence for trehalose-cis-methoxy-mono-mycolate O-mycolyltransferase, EC# 2.3.1.-   RXN1G-1634 
  

 

 
 Hit 
 

 
 Common 
 

 
 P 
 

 
 #Q 
 

 
 Best Qry 
 

 
 Best Eval 
 

 
 Avg Rank 
 

 
 Aln Len 
 

 
 Qry Len 
 

 
 IDOP? 
 

 
 adj? 
 

 

 

 
GKFG-653-MONOMER
 

 
bcv:Bcav_2498 ispG; 4-hyd
 

 
3.182795e-4
 

 
1.0
 

 
P0A4V2
 

 
0.0388288
 

 
1.0
 

 
0.36094674
 

 
338
 

 
T
 

 
NIL
 

 

 
     Evidence for trehalose-trans-methoxy-mono-mycolate O-mycolyltransferase, EC# 2.3.1.-   RXN1G-1635 
  

 

 
 Hit 
 

 
 Common 
 

 
 P 
 

 
 #Q 
 

 
 Best Qry 
 

 
 Best Eval 
 

 
 Avg Rank 
 

 
 Aln Len 
 

 
 Qry Len 
 

 
 IDOP? 
 

 
 adj? 
 

 

 

 
GKFG-653-MONOMER
 

 
bcv:Bcav_2498 ispG; 4-hyd
 

 
3.182795e-4
 

 
1.0
 

 
P0A4V2
 

 
0.0388288
 

 
1.0
 

 
0.36094674
 

 
338
 

 
T
 

 
NIL
 

 

 
     Evidence for trehalose-cis-keto-mono-mycolate O-mycolyltransferase, EC# 2.3.1.-   RXN1G-1636 
  

 

 
 Hit 
 

 
 Common 
 

 
 P 
 

 
 #Q 
 

 
 Best Qry 
 

 
 Best Eval 
 

 
 Avg Rank 
 

 
 Aln Len 
 

 
 Qry Len 
 

 
 IDOP? 
 

 
 adj? 
 

 

 

 
GKFG-653-MONOMER
 

 
bcv:Bcav_2498 ispG; 4-hyd
 

 
3.182795e-4
 

 
1.0
 

 
P0A4V2
 

 
0.0388288
 

 
1.0
 

 
0.36094674
 

 
338
 

 
T
 

 
NIL
 

 

 
     Evidence for trehalose-trans-keto-mono-mycolate O-mycolyltransferase, EC# 2.3.1.-   RXN1G-1637 
  

 

 
 Hit 
 

 
 Common 
 

 
 P 
 

 
 #Q 
 

 
 Best Qry 
 

 
 Best Eval 
 

 
 Avg Rank 
 

 
 Aln Len 
 

 
 Qry Len 
 

 
 IDOP? 
 

 
 adj? 
 

 

 

 
GKFG-653-MONOMER
 

 
bcv:Bcav_2498 ispG; 4-hyd
 

 
3.182795e-4
 

 
1.0
 

 
P0A4V2
 

 
0.0388288
 

 
1.0
 

 
0.36094674
 

 
338
 

 
T
 

 
NIL
 

 

 
     Evidence for , EC# 2.3.1.-   RXN-10059 
  
no BLAST hits found
 
     Evidence for , EC# 2.3.1.-   RXN-10060 
  
no BLAST hits found
 
     Evidence for 3-hydroxycerotyl-[acp] dehydrase, EC# 2.3.1.-   RXN-10061 
  
no BLAST hits found
 
     Evidence for trans-hexacos-2-enoyl-[acp] reductase, EC# 2.3.1.-   RXN-10062 
  
no BLAST hits found
 
     

  CDP-diacylglycerol biosynthesis II     Total # of reactions in pathway = 4   Present reactions: 3
  

 

 
 Reaction 
 

 
 Protein(s) 
 

 

 

 
CDPDIGLYSYN-RXN
 

 
(# )
 

 

 

 
GLYC3PDEHYDROGBIOSYN-RXN
 

 
(# 
 # 
 # 
 # )
 

 

 

 
1-ACYLGLYCEROL-3-P-ACYLTRANSFER-RXN
 

 
(# )
 

 

 
   Missing reactions: 1
  

 

 
 RXN-10462 
 

 

 
        Evidence for acyl-ACP:sn-glycerol-3-phosphate 1-O-acyltransferase, EC# 2.3.1.15   RXN-10462 
  
no BLAST hits found
 
     

  CDP-diacylglycerol biosynthesis I     Total # of reactions in pathway = 4   Present reactions: 3
  

 

 
 Reaction 
 

 
 Protein(s) 
 

 

 

 
CDPDIGLYSYN-RXN
 

 
(# )
 

 

 

 
RXN-1623
 

 
(# )
 

 

 

 
GLYC3PDEHYDROGBIOSYN-RXN
 

 
(# 
 # 
 # 
 # )
 

 

 
   Missing reactions: 1
  

 

 
 RXN-1381 
 

 

 
        Evidence for glycerol-3-phosphate  O -acyltransferase, EC# 2.3.1.15   RXN-1381 
  

 

 
 Hit 
 

 
 Common 
 

 
 P 
 

 
 #Q 
 

 
 Best Qry 
 

 
 Best Eval 
 

 
 Avg Rank 
 

 
 Aln Len 
 

 
 Qry Len 
 

 
 IDOP? 
 

 
 adj? 
 

 

 

 
GKFG-604-MONOMER
 

 
bcv:Bcav_1904 phospholipi
 

 
0.008591636
 

 
1.0
 

 
P32784
 

 
0.0274184
 

 
1.0
 

 
0.15678525
 

 
759
 

 
T
 

 
T
 

 

 

 
GKFG-304-MONOMER
 

 
hypothetical protein (db=
 

 
4.188785e-5
 

 
1.0
 

 
A9MPV6
 

 
0.740774
 

 
1.0
 

 
0.2
 

 
205
 

 
NIL
 

 
NIL
 

 

 

 
GKFG-1729-MONOMER
 

 
hypothetical protein (db=
 

 
1.5421621e-5
 

 
1.0
 

 
Q43869
 

 
0.660302
 

 
1.0
 

 
0.13347457
 

 
472
 

 
T
 

 
NIL
 

 

 
     

  phosphatidylglycerol biosynthesis II (non-plastidic)     Total # of reactions in pathway = 3   Present reactions: 2
  

 

 
 Reaction 
 

 
 Protein(s) 
 

 

 

 
PWY-5667
 

 
NIL
 

 

 

 
PHOSPHAGLYPSYN-RXN
 

 
(# 
 # )
 

 

 
   Missing reactions: 1
  

 

 
 PGPPHOSPHA-RXN 
 

 

 
        Evidence for phosphatidylglycerophosphatase, EC# 3.1.3.27   PGPPHOSPHA-RXN 
  

 

 
 Hit 
 

 
 Common 
 

 
 P 
 

 
 #Q 
 

 
 Best Qry 
 

 
 Best Eval 
 

 
 Avg Rank 
 

 
 Aln Len 
 

 
 Qry Len 
 

 
 IDOP? 
 

 
 adj? 
 

 

 

 
GKFG-1239-MONOMER
 

 
(db=HMMPfam db_id=PF05738
 

 
3.182795e-4
 

 
1.0
 

 
P44570
 

 
0.194269
 

 
1.0
 

 
0.406639
 

 
241
 

 
T
 

 
NIL
 

 

 

 
GKFG-1734-MONOMER
 

 
pad:TIIST44_04425 HAD hyd
 

 
1.3958463e-4
 

 
1.0
 

 
P38812
 

 
0.00913501
 

 
1.0
 

 
0.27567568
 

 
185
 

 
T
 

 
NIL
 

 

 

 
GKFG-1528-MONOMER
 

 
ROK family protein (db=KE
 

 
2.5307965e-5
 

 
1.0
 

 
P44570
 

 
0.495145
 

 
2.0
 

 
0.2780083
 

 
241
 

 
T
 

 
NIL
 

 

 
     

  tRNA processing pathway I     Total # of reactions in pathway = 10   Present reactions: 4
  

 

 
 Reaction 
 

 
 Protein(s) 
 

 

 

 
RXN0-6482
 

 
(# )
 

 

 

 
RXN0-6484
 

 
(# )
 

 

 

 
RXN0-6483
 

 
(# )
 

 

 

 
RXN0-6481
 

 
(# )
 

 

 
   Missing reactions: 6
  

 

 
 RXN0-6478 
 

 

 

 
 RXN0-6479 
 

 

 

 
 RXN0-4222 
 

 

 

 
 RXN0-6480 
 

 

 

 
 3.1.26.5-RXN 
 

 

 

 
 RXN0-6485 
 

 

 
        Evidence for , EC# 3.1.26.12   RXN0-6478 
  

 

 
 Hit 
 

 
 Common 
 

 
 P 
 

 
 #Q 
 

 
 Best Qry 
 

 
 Best Eval 
 

 
 Avg Rank 
 

 
 Aln Len 
 

 
 Qry Len 
 

 
 IDOP? 
 

 
 adj? 
 

 

 

 
GKFG-739-MONOMER
 

 
bcv:Bcav_1600 DEAD/DEAH b
 

 
1.3945618e-5
 

 
1.0
 

 
P21513
 

 
0.208462
 

 
1.0
 

 
0.08765316
 

 
1061
 

 
T
 

 
NIL
 

 

 

 
GKFG-2023-MONOMER
 

 
mcu:HMPREF0573_11895 hypo
 

 
2.5282066e-6
 

 
1.0
 

 
P21513
 

 
0.294566
 

 
2.0
 

 
0.05466541
 

 
1061
 

 
T
 

 
NIL
 

 

 
     Evidence for , EC# 3.1.13.1   RXN0-6479 
  

 

 
 Hit 
 

 
 Common 
 

 
 P 
 

 
 #Q 
 

 
 Best Qry 
 

 
 Best Eval 
 

 
 Avg Rank 
 

 
 Aln Len 
 

 
 Qry Len 
 

 
 IDOP? 
 

 
 adj? 
 

 

 

 
GKFG-1540-MONOMER
 

 
rdn:HMPREF0733_11743 rph;
 

 
0.07274204
 

 
1.0
 

 
P05055
 

 
3.06709e-5
 

 
1.0
 

 
0.28410688
 

 
711
 

 
T
 

 
T
 

 

 

 
GKFG-1064-MONOMER
 

 
homoserine kinase; K00872
 

 
1.3945618e-5
 

 
1.0
 

 
P30850
 

 
0.2925
 

 
1.0
 

 
0.0931677
 

 
644
 

 
T
 

 
NIL
 

 

 

 
GKFG-679-MONOMER
 

 
hypothetical protein; K06
 

 
2.5282066e-6
 

 
1.0
 

 
P05055
 

 
0.211937
 

 
2.0
 

 
0.06610408
 

 
711
 

 
T
 

 
NIL
 

 

 
     Evidence for exoribonuclease II, EC# 3.1.13.1   RXN0-4222 
  

 

 
 Hit 
 

 
 Common 
 

 
 P 
 

 
 #Q 
 

 
 Best Qry 
 

 
 Best Eval 
 

 
 Avg Rank 
 

 
 Aln Len 
 

 
 Qry Len 
 

 
 IDOP? 
 

 
 adj? 
 

 

 

 
GKFG-1055-MONOMER
 

 
UniRef90_B5CMC4 Putative 
 

 
3.182795e-4
 

 
1.0
 

 
P0A8V0
 

 
0.255498
 

 
1.0
 

 
0.3967213
 

 
305
 

 
T
 

 
NIL
 

 

 

 
GKFG-844-MONOMER
 

 
ahe:Arch_0366 ATP synthas
 

 
1.5421621e-5
 

 
1.0
 

 
Q89AM0
 

 
0.139558
 

 
1.0
 

 
0.14705883
 

 
646
 

 
T
 

 
NIL
 

 

 

 
GKFG-1566-MONOMER
 

 
hypothetical protein; K06
 

 
1.5421621e-5
 

 
1.0
 

 
Q9JL16
 

 
0.582302
 

 
1.0
 

 
0.17333333
 

 
300
 

 
T
 

 
NIL
 

 

 

 
GKFG-1242-MONOMER
 

 
cfi:Celf_2761 alpha amyla
 

 
1.3945618e-5
 

 
1.0
 

 
A5UFG3
 

 
0.774875
 

 
1.0
 

 
0.06980273
 

 
659
 

 
T
 

 
NIL
 

 

 

 
GKFG-1739-MONOMER
 

 
bfa:Bfae_03150 uracil-xan
 

 
2.795795e-6
 

 
1.0
 

 
P0A8V0
 

 
0.900366
 

 
2.0
 

 
0.15081967
 

 
305
 

 
T
 

 
NIL
 

 

 
     Evidence for , EC# 3.1.26.5   RXN0-6480 
  

 

 
 Hit 
 

 
 Common 
 

 
 P 
 

 
 #Q 
 

 
 Best Qry 
 

 
 Best Eval 
 

 
 Avg Rank 
 

 
 Aln Len 
 

 
 Qry Len 
 

 
 IDOP? 
 

 
 adj? 
 

 

 

 
GKFG-528-MONOMER
 

 
Ribonuclease P protein co
 

 
1.5421621e-5
 

 
1.0
 

 
P0A7Y8
 

 
0.0151732
 

 
1.0
 

 
0.2184874
 

 
119
 

 
T
 

 
NIL
 

 

 
     Evidence for ribonuclease P, EC# 3.1.26.5   3.1.26.5-RXN 
  

 

 
 Hit 
 

 
 Common 
 

 
 P 
 

 
 #Q 
 

 
 Best Qry 
 

 
 Best Eval 
 

 
 Avg Rank 
 

 
 Aln Len 
 

 
 Qry Len 
 

 
 IDOP? 
 

 
 adj? 
 

 

 

 
GKFG-342-MONOMER
 

 
transmembrane_regions (db
 

 
0.0051016076
 

 
1.0
 

 
A8ZRZ3
 

 
1.93048e-4
 

 
1.0
 

 
0.6528926
 

 
121
 

 
T
 

 
NIL
 

 

 

 
GKFG-1110-MONOMER
 

 
mph:MLP_51590 putative pe
 

 
0.0051016076
 

 
1.0
 

 
Q8U007
 

 
0.191927
 

 
1.0
 

 
0.62992126
 

 
127
 

 
T
 

 
NIL
 

 

 

 
GKFG-805-MONOMER
 

 
ske:Sked_35330 exodeoxyri
 

 
0.0051016076
 

 
1.0
 

 
Q2KTI7
 

 
0.500233
 

 
1.0
 

 
0.64166665
 

 
120
 

 
T
 

 
NIL
 

 

 

 
GKFG-1573-MONOMER
 

 
Putative uncharacterized 
 

 
0.0032236807
 

 
1.0
 

 
A1WWE2
 

 
0.237736
 

 
3.0
 

 
0.86885244
 

 
122
 

 
T
 

 
NIL
 

 

 

 
GKFG-1306-MONOMER
 

 
putative dihydrofolate re
 

 
0.0018147272
 

 
1.0
 

 
A9NE63
 

 
0.100767
 

 
1.0
 

 
0.4827586
 

 
116
 

 
T
 

 
NIL
 

 

 

 
GKFG-528-MONOMER
 

 
Ribonuclease P protein co
 

 
8.15234e-4
 

 
79.0
 

 
P48206
 

 
1.55819e-9
 

 
1.0253165
 

 
0.46840334
 

 
123
 

 
T
 

 
NIL
 

 

 

 
GKFG-614-MONOMER
 

 
Putative glycerate kinase
 

 
4.0159098e-4
 

 
1.0
 

 
B1VPE8
 

 
0.980085
 

 
3.0
 

 
0.60162604
 

 
123
 

 
T
 

 
NIL
 

 

 

 
GKFG-1015-MONOMER
 

 
raiA; putative ribosome-a
 

 
4.0159098e-4
 

 
1.0
 

 
Q0VKU6
 

 
0.633168
 

 
3.0
 

 
0.6694215
 

 
121
 

 
T
 

 
NIL
 

 

 

 
GKFG-1244-MONOMER
 

 
ahe:Arch_0389 tryptophany
 

 
4.0159098e-4
 

 
1.0
 

 
A6WYM5
 

 
0.603895
 

 
3.0
 

 
0.6028369
 

 
141
 

 
T
 

 
NIL
 

 

 

 
GKFG-839-MONOMER
 

 
bfa:Bfae_19090 hypothetic
 

 
3.294785e-4
 

 
1.0
 

 
Q4JSC2
 

 
0.0465126
 

 
2.0
 

 
0.5813953
 

 
129
 

 
T
 

 
NIL
 

 

 

 
GKFG-1596-MONOMER
 

 
DeoR family transcription
 

 
3.294785e-4
 

 
1.0
 

 
Q5KU54
 

 
0.0833065
 

 
2.0
 

 
0.5206612
 

 
121
 

 
T
 

 
NIL
 

 

 

 
GKFG-1745-MONOMER
 

 
winged helix family two c
 

 
3.294785e-4
 

 
1.0
 

 
A6WYM5
 

 
0.29435
 

 
2.0
 

 
0.5460993
 

 
141
 

 
T
 

 
NIL
 

 

 

 
GKFG-330-MONOMER
 

 
2C-methyl-D-erythritol 2,
 

 
3.294785e-4
 

 
1.0
 

 
Q0VKU6
 

 
0.372809
 

 
2.0
 

 
0.54545456
 

 
121
 

 
T
 

 
NIL
 

 

 

 
GKFG-701-MONOMER
 

 
ach:Achl_1330 fibronectin
 

 
3.294785e-4
 

 
1.0
 

 
C0ZVP7
 

 
0.33391
 

 
2.0
 

 
0.48412699
 

 
126
 

 
T
 

 
NIL
 

 

 

 
GKFG-1883-MONOMER
 

 
transmembrane_regions (db
 

 
3.182795e-4
 

 
1.0
 

 
Q6APZ0
 

 
0.308541
 

 
1.0
 

 
0.40833333
 

 
120
 

 
T
 

 
NIL
 

 

 

 
GKFG-1328-MONOMER
 

 
putative tRNA-pseudouridi
 

 
3.182795e-4
 

 
1.0
 

 
Q4A750
 

 
0.443843
 

 
1.0
 

 
0.45535713
 

 
112
 

 
T
 

 
NIL
 

 

 

 
GKFG-378-MONOMER
 

 
bbp:BBPR_0460 hypothetica
 

 
3.182795e-4
 

 
1.0
 

 
Q72LI2
 

 
0.0944725
 

 
1.0
 

 
0.38650307
 

 
163
 

 
T
 

 
NIL
 

 

 

 
GKFG-1887-MONOMER
 

 
nca:Noca_4168 phosphoenol
 

 
3.182795e-4
 

 
1.0
 

 
A9A5I7
 

 
0.0321164
 

 
1.0
 

 
0.4431818
 

 
88
 

 
T
 

 
NIL
 

 

 

 
GKFG-809-MONOMER
 

 
ahe:Arch_0180 ATP-depende
 

 
3.182795e-4
 

 
1.0
 

 
Q04CX7
 

 
0.266049
 

 
1.0
 

 
0.3852459
 

 
122
 

 
T
 

 
NIL
 

 

 

 
GKFG-316-MONOMER
 

 
putative secreted protein
 

 
3.182795e-4
 

 
1.0
 

 
Q12ZC2
 

 
0.144457
 

 
1.0
 

 
0.36099586
 

 
241
 

 
T
 

 
NIL
 

 

 

 
GKFG-1448-MONOMER
 

 
cdz:CD31A_0959 putative p
 

 
3.182795e-4
 

 
1.0
 

 
A8AA22
 

 
0.369365
 

 
1.0
 

 
0.45652175
 

 
92
 

 
T
 

 
NIL
 

 

 

 
GKFG-1048-MONOMER
 

 
bfa:Bfae_17560 methionyl-
 

 
1.3958463e-4
 

 
2.0
 

 
P38208
 

 
0.203032
 

 
1.0
 

 
0.33543232
 

 
133
 

 
T
 

 
NIL
 

 

 

 
GKFG-16-MONOMER
 

 
(db=HMMPfam db_id=PF05738
 

 
1.3958463e-4
 

 
2.0
 

 
C4KHD8
 

 
0.0778557
 

 
1.0
 

 
0.2968143
 

 
143
 

 
T
 

 
NIL
 

 

 

 
GKFG-42-MONOMER
 

 
140655..140831 + ( gc_con
 

 
1.3958463e-4
 

 
1.0
 

 
A0LLH1
 

 
0.173225
 

 
1.0
 

 
0.26890758
 

 
119
 

 
T
 

 
NIL
 

 

 

 
GKFG-656-MONOMER
 

 
hypothetical protein (db=
 

 
1.3958463e-4
 

 
1.0
 

 
B8GRD3
 

 
0.500724
 

 
1.0
 

 
0.275
 

 
120
 

 
T
 

 
NIL
 

 

 

 
GKFG-681-MONOMER
 

 
archaeal fructose-1,6-bis
 

 
1.3958463e-4
 

 
1.0
 

 
Q9YC00
 

 
0.660265
 

 
1.0
 

 
0.3222749
 

 
211
 

 
T
 

 
NIL
 

 

 

 
GKFG-1020-MONOMER
 

 
apn:Asphe3_25810 response
 

 
1.3958463e-4
 

 
1.0
 

 
A5CY49
 

 
0.180327
 

 
1.0
 

 
0.26785713
 

 
112
 

 
T
 

 
NIL
 

 

 

 
GKFG-1057-MONOMER
 

 
bbi:BBIF_1733 sialidase; 
 

 
1.3958463e-4
 

 
1.0
 

 
Q3Z7P1
 

 
0.0333027
 

 
1.0
 

 
0.34166667
 

 
120
 

 
T
 

 
NIL
 

 

 

 
GKFG-1216-MONOMER
 

 
blm:BLLJ_0965 cell surfac
 

 
1.3958463e-4
 

 
1.0
 

 
P28005
 

 
0.745822
 

 
1.0
 

 
0.24855492
 

 
173
 

 
T
 

 
NIL
 

 

 

 
GKFG-1221-MONOMER
 

 
thioredoxin domain-contai
 

 
1.3958463e-4
 

 
1.0
 

 
A7I9J3
 

 
0.127523
 

 
1.0
 

 
0.31730768
 

 
104
 

 
T
 

 
NIL
 

 

 

 
GKFG-1944-MONOMER
 

 
bcv:Bcav_2184 excinucleas
 

 
1.3958463e-4
 

 
1.0
 

 
Q469M7
 

 
0.31867
 

 
1.0
 

 
0.35555556
 

 
135
 

 
T
 

 
NIL
 

 

 

 
GKFG-1290-MONOMER
 

 
vma:VAB18032_29276 alpha 
 

 
1.3958463e-4
 

 
1.0
 

 
Q8TYB5
 

 
0.346584
 

 
1.0
 

 
0.27459016
 

 
244
 

 
T
 

 
NIL
 

 

 

 
GKFG-1956-MONOMER
 

 
bsd:BLASA_2671 pafA; pup-
 

 
1.3958463e-4
 

 
1.0
 

 
Q13SH2
 

 
0.00480038
 

 
1.0
 

 
0.352518
 

 
139
 

 
T
 

 
NIL
 

 

 

 
GKFG-192-MONOMER
 

 
fre:Franean1_4666 xylulok
 

 
1.3958463e-4
 

 
1.0
 

 
B0R594
 

 
0.694185
 

 
1.0
 

 
0.28658536
 

 
164
 

 
T
 

 
NIL
 

 

 

 
GKFG-1218-MONOMER
 

 
mcu:HMPREF0573_10985 lacZ
 

 
1.3958463e-4
 

 
1.0
 

 
Q5FPY1
 

 
0.133777
 

 
1.0
 

 
0.32258064
 

 
124
 

 
T
 

 
NIL
 

 

 

 
GKFG-30-MONOMER
 

 
integrase family protein 
 

 
5.771539e-5
 

 
1.0
 

 
A7I9J3
 

 
0.464407
 

 
2.0
 

 
0.43269232
 

 
104
 

 
T
 

 
NIL
 

 

 

 
GKFG-479-MONOMER
 

 
DNA replication and repai
 

 
5.771539e-5
 

 
1.0
 

 
Q3M7J6
 

 
0.528234
 

 
2.0
 

 
0.4
 

 
140
 

 
T
 

 
NIL
 

 

 

 
GKFG-507-MONOMER
 

 
iva:Isova_2863 glutamate-
 

 
5.771539e-5
 

 
1.0
 

 
A8L8W2
 

 
0.445416
 

 
2.0
 

 
0.41322315
 

 
121
 

 
T
 

 
NIL
 

 

 

 
GKFG-299-MONOMER
 

 
bcv:Bcav_0457 hypothetica
 

 
5.771539e-5
 

 
1.0
 

 
A9A5I7
 

 
0.503228
 

 
2.0
 

 
0.45454547
 

 
88
 

 
T
 

 
NIL
 

 

 

 
GKFG-1207-MONOMER
 

 
no description (db=HMMSma
 

 
5.771539e-5
 

 
1.0
 

 
B7V7A6
 

 
0.264337
 

 
2.0
 

 
0.42962962
 

 
135
 

 
T
 

 
NIL
 

 

 

 
GKFG-1024-MONOMER
 

 
glycosyl transferase fami
 

 
5.771539e-5
 

 
1.0
 

 
B1MN96
 

 
0.0833224
 

 
2.0
 

 
0.43307087
 

 
127
 

 
T
 

 
NIL
 

 

 

 
GKFG-708-MONOMER
 

 
120023..121090 - ( gc_con
 

 
5.771539e-5
 

 
1.0
 

 
A1WWE2
 

 
0.0209902
 

 
2.0
 

 
0.45901638
 

 
122
 

 
T
 

 
NIL
 

 

 

 
GKFG-239-MONOMER
 

 
bcv:Bcav_1088 hemH; ferro
 

 
2.5307965e-5
 

 
2.0
 

 
Q9BUL9
 

 
0.0713083
 

 
1.5
 

 
0.29709524
 

 
199
 

 
T
 

 
NIL
 

 

 

 
GKFG-1754-MONOMER
 

 
cfl:Cfla_1371 aminopeptid
 

 
2.5307965e-5
 

 
2.0
 

 
A4T4T3
 

 
0.0115349
 

 
1.5
 

 
0.24282448
 

 
124
 

 
T
 

 
NIL
 

 

 

 
GKFG-29-MONOMER
 

 
std:SPPN_11055 Zinc metal
 

 
2.5307965e-5
 

 
2.0
 

 
B8FMU9
 

 
0.212466
 

 
1.5
 

 
0.30955625
 

 
118
 

 
T
 

 
NIL
 

 

 

 
GKFG-1489-MONOMER
 

 
lacto-N-biosidase (EC:3.2
 

 
2.5307965e-5
 

 
1.0
 

 
Q0AE58
 

 
0.874249
 

 
2.0
 

 
0.28099173
 

 
121
 

 
T
 

 
NIL
 

 

 

 
GKFG-1082-MONOMER
 

 
144487..144945 - ( gc_con
 

 
2.5307965e-5
 

 
1.0
 

 
A6WGN2
 

 
0.192553
 

 
2.0
 

 
0.28333333
 

 
120
 

 
T
 

 
NIL
 

 

 

 
GKFG-1008-MONOMER
 

 
141281..141610 - ( gc_con
 

 
1.5421621e-5
 

 
1.0
 

 
Q2YZB7
 

 
0.427832
 

 
1.0
 

 
0.23076923
 

 
117
 

 
T
 

 
NIL
 

 

 

 
GKFG-1528-MONOMER
 

 
ROK family protein (db=KE
 

 
1.5421621e-5
 

 
1.0
 

 
Q8PWM8
 

 
0.284676
 

 
1.0
 

 
0.1682243
 

 
107
 

 
T
 

 
NIL
 

 

 

 
GKFG-1483-MONOMER
 

 
mcu:HMPREF0573_10024 argD
 

 
1.5421621e-5
 

 
1.0
 

 
Q7U524
 

 
0.488537
 

 
1.0
 

 
0.140625
 

 
128
 

 
T
 

 
NIL
 

 

 

 
GKFG-1829-MONOMER
 

 
putative ethanolamine two
 

 
1.5421621e-5
 

 
1.0
 

 
O26119
 

 
0.259069
 

 
1.0
 

 
0.22580644
 

 
93
 

 
T
 

 
NIL
 

 

 

 
GKFG-97-MONOMER
 

 
mcu:HMPREF0573_10969 rpoC
 

 
1.3945618e-5
 

 
1.0
 

 
Q9UTA4
 

 
0.957919
 

 
1.0
 

 
0.09885387
 

 
698
 

 
T
 

 
NIL
 

 

 

 
GKFG-1747-MONOMER
 

 
MerR family transcription
 

 
1.09376415e-5
 

 
1.0
 

 
Q1QS97
 

 
0.371838
 

 
3.0
 

 
0.2890625
 

 
128
 

 
T
 

 
NIL
 

 

 

 
GKFG-814-MONOMER
 

 
ahe:Arch_0168 chaperone p
 

 
1.09376415e-5
 

 
1.0
 

 
B7V7A6
 

 
0.938869
 

 
3.0
 

 
0.31111112
 

 
135
 

 
T
 

 
NIL
 

 

 

 
GKFG-427-MONOMER
 

 
pfr:PFREUD_19060 iolC; my
 

 
2.795795e-6
 

 
1.0
 

 
Q1QS97
 

 
0.160856
 

 
2.0
 

 
0.15625
 

 
128
 

 
T
 

 
NIL
 

 

 

 
GKFG-512-MONOMER
 

 
transmembrane_regions (db
 

 
2.795795e-6
 

 
1.0
 

 
B2J0Q5
 

 
0.423931
 

 
2.0
 

 
0.22857143
 

 
140
 

 
T
 

 
NIL
 

 

 

 
GKFG-1981-MONOMER
 

 
LPXTG_anchor: LPXTG-motif
 

 
2.795795e-6
 

 
1.0
 

 
B1I998
 

 
0.563229
 

 
2.0
 

 
0.16260162
 

 
123
 

 
T
 

 
NIL
 

 

 

 
GKFG-739-MONOMER
 

 
bcv:Bcav_1600 DEAD/DEAH b
 

 
2.795795e-6
 

 
1.0
 

 
Q602M8
 

 
0.672817
 

 
2.0
 

 
0.1875
 

 
128
 

 
T
 

 
NIL
 

 

 

 
GKFG-765-MONOMER
 

 
cfl:Cfla_2325 Preprotein 
 

 
2.795795e-6
 

 
1.0
 

 
Q7VN34
 

 
0.434445
 

 
2.0
 

 
0.17073171
 

 
123
 

 
T
 

 
NIL
 

 

 

 
GKFG-1418-MONOMER
 

 
mcu:HMPREF0573_10758 AAA+
 

 
2.5282066e-6
 

 
1.0
 

 
O26119
 

 
0.506193
 

 
2.0
 

 
0.10752688
 

 
93
 

 
T
 

 
NIL
 

 

 

 
GKFG-585-MONOMER
 

 
hypothetical protein (db=
 

 
1.2082764e-6
 

 
1.0
 

 
Q9BUL9
 

 
0.637758
 

 
3.0
 

 
0.19095477
 

 
199
 

 
T
 

 
NIL
 

 

 
     Evidence for , EC# 3.1.26.12   RXN0-6485 
  

 

 
 Hit 
 

 
 Common 
 

 
 P 
 

 
 #Q 
 

 
 Best Qry 
 

 
 Best Eval 
 

 
 Avg Rank 
 

 
 Aln Len 
 

 
 Qry Len 
 

 
 IDOP? 
 

 
 adj? 
 

 

 

 
GKFG-739-MONOMER
 

 
bcv:Bcav_1600 DEAD/DEAH b
 

 
1.3945618e-5
 

 
1.0
 

 
P21513
 

 
0.208462
 

 
1.0
 

 
0.08765316
 

 
1061
 

 
T
 

 
NIL
 

 

 

 
GKFG-2023-MONOMER
 

 
mcu:HMPREF0573_11895 hypo
 

 
2.5282066e-6
 

 
1.0
 

 
P21513
 

 
0.294566
 

 
2.0
 

 
0.05466541
 

 
1061
 

 
T
 

 
NIL
 

 

 
     

  queuosine biosynthesis     Total # of reactions in pathway = 4   Present reactions: 1
  

 

 
 Reaction 
 

 
 Protein(s) 
 

 

 

 
RXN0-1321
 

 
(# )
 

 

 
   Missing reactions: 3
  

 

 
 RXN0-4022 
 

 

 

 
 RXN0-1342 
 

 

 

 
 RXN-12104 
 

 

 
        Evidence for preQ  1   synthase, EC# 1.7.1.13   RXN0-4022 
  

 

 
 Hit 
 

 
 Common 
 

 
 P 
 

 
 #Q 
 

 
 Best Qry 
 

 
 Best Eval 
 

 
 Avg Rank 
 

 
 Aln Len 
 

 
 Qry Len 
 

 
 IDOP? 
 

 
 adj? 
 

 

 

 
GKFG-1234-MONOMER
 

 
mcu:HMPREF0573_11119 glgB
 

 
0.0051016076
 

 
1.0
 

 
Q7UVG9
 

 
0.0590844
 

 
1.0
 

 
0.6694215
 

 
121
 

 
T
 

 
NIL
 

 

 

 
GKFG-651-MONOMER
 

 
mcu:HMPREF0573_11422 proS
 

 
3.182795e-4
 

 
1.0
 

 
Q6ARX8
 

 
0.244398
 

 
1.0
 

 
0.41304347
 

 
276
 

 
T
 

 
NIL
 

 

 

 
GKFG-1382-MONOMER
 

 
mcu:HMPREF0573_10007 cls;
 

 
1.3958463e-4
 

 
1.0
 

 
Q46J47
 

 
0.151238
 

 
1.0
 

 
0.30714285
 

 
140
 

 
T
 

 
NIL
 

 

 

 
GKFG-552-MONOMER
 

 
hypothetical protein (db=
 

 
4.188785e-5
 

 
1.0
 

 
C5BR59
 

 
0.992894
 

 
1.0
 

 
0.22463769
 

 
276
 

 
NIL
 

 
NIL
 

 

 

 
GKFG-1678-MONOMER
 

 
mcu:HMPREF0573_11450 valS
 

 
1.5421621e-5
 

 
1.0
 

 
Q2SDT2
 

 
0.262336
 

 
1.0
 

 
0.13868614
 

 
274
 

 
T
 

 
NIL
 

 

 

 
GKFG-1648-MONOMER
 

 
str:Sterm_2106 phosphorib
 

 
1.5421621e-5
 

 
1.0
 

 
B3EMR0
 

 
0.0934885
 

 
1.0
 

 
0.23728813
 

 
118
 

 
T
 

 
NIL
 

 

 

 
GKFG-1968-MONOMER
 

 
5'-nucleotidase (EC:3.1.3
 

 
1.5421621e-5
 

 
1.0
 

 
B5EP57
 

 
0.688745
 

 
1.0
 

 
0.20567375
 

 
141
 

 
T
 

 
NIL
 

 

 

 
GKFG-1239-MONOMER
 

 
(db=HMMPfam db_id=PF05738
 

 
1.5421621e-5
 

 
1.0
 

 
B0CDX9
 

 
0.638168
 

 
1.0
 

 
0.2361111
 

 
144
 

 
T
 

 
NIL
 

 

 

 
GKFG-857-MONOMER
 

 
xylB; xylulose kinase (db
 

 
1.3945618e-5
 

 
2.0
 

 
Q47DJ9
 

 
0.103976
 

 
1.0
 

 
0.08203472
 

 
283
 

 
T
 

 
NIL
 

 

 

 
GKFG-772-MONOMER
 

 
cell surface protein (db=
 

 
1.3945618e-5
 

 
1.0
 

 
C1DRN1
 

 
0.473851
 

 
1.0
 

 
0.0942029
 

 
276
 

 
T
 

 
NIL
 

 

 
     Evidence for    RXN0-1342 
  

 

 
 Hit 
 

 
 Common 
 

 
 P 
 

 
 #Q 
 

 
 Best Qry 
 

 
 Best Eval 
 

 
 Avg Rank 
 

 
 Aln Len 
 

 
 Qry Len 
 

 
 IDOP? 
 

 
 adj? 
 

 

 

 
GKFG-2007-MONOMER
 

 
NifU family SUF system Fe
 

 
1.5421621e-5
 

 
1.0
 

 
P0A7F9
 

 
0.83679
 

 
1.0
 

 
0.16292135
 

 
356
 

 
T
 

 
NIL
 

 

 
     Evidence for    RXN-12104 
  
no BLAST hits found
 
     

  guanosine nucleotides  de novo  biosynthesis     Total # of reactions in pathway = 9   Present reactions: 8
  

 

 
 Reaction 
 

 
 Protein(s) 
 

 

 

 
IMP-DEHYDROG-RXN
 

 
(# 
 # 
 # )
 

 

 

 
GMP-SYN-GLUT-RXN
 

 
(# 
 # )
 

 

 

 
GUANYL-KIN-RXN
 

 
(# )
 

 

 

 
GDPKIN-RXN
 

 
(# )
 

 

 

 
GDPREDUCT-RXN
 

 
NIL
 

 

 

 
RXN0-746
 

 
NIL
 

 

 

 
RXN0-748
 

 
(# )
 

 

 

 
DGDPKIN-RXN
 

 
(# )
 

 

 
   Missing reactions: 1
  

 

 
 GMP-SYN-NH3-RXN 
 

 

 
        Evidence for GMP synthase, EC# 6.3.4.1   GMP-SYN-NH3-RXN 
  

 

 
 Hit 
 

 
 Common 
 

 
 P 
 

 
 #Q 
 

 
 Best Qry 
 

 
 Best Eval 
 

 
 Avg Rank 
 

 
 Aln Len 
 

 
 Qry Len 
 

 
 IDOP? 
 

 
 adj? 
 

 

 

 
GKFG-235-MONOMER
 

 
pfr:PFREUD_19110 iolT3; m
 

 
2.5307965e-5
 

 
1.0
 

 
A9GH25
 

 
0.785792
 

 
2.0
 

 
0.28099173
 

 
242
 

 
T
 

 
NIL
 

 

 

 
GKFG-791-MONOMER
 

 
iva:Isova_1606 carbamoyl-
 

 
1.5421621e-5
 

 
3.0
 

 
P04079
 

 
5.57254e-6
 

 
1.0
 

 
0.1275765
 

 
525
 

 
T
 

 
NIL
 

 

 

 
GKFG-1857-MONOMER
 

 
aai:AARI_29350 hsdM; type
 

 
2.5282066e-6
 

 
1.0
 

 
P04079
 

 
0.198005
 

 
2.0
 

 
0.099047616
 

 
525
 

 
T
 

 
NIL
 

 

 
     

  inosine-5'-phosphate biosynthesis I     Total # of reactions in pathway = 6   Present reactions: 5
  

 

 
 Reaction 
 

 
 Protein(s) 
 

 

 

 
SAICARSYN-RXN
 

 
(# 
 # )
 

 

 

 
AICARSYN-RXN
 

 
(# )
 

 

 

 
AICARTRANSFORM-RXN
 

 
(# )
 

 

 

 
IMPCYCLOHYDROLASE-RXN
 

 
(# )
 

 

 

 
RXN0-743
 

 
(# )
 

 

 
   Missing reactions: 1
  

 

 
 RXN0-742 
 

 

 
        Evidence for 5-(carboxyamino)imidazole ribonucleotide synthase, EC# 6.3.4.18   RXN0-742 
  

 

 
 Hit 
 

 
 Common 
 

 
 P 
 

 
 #Q 
 

 
 Best Qry 
 

 
 Best Eval 
 

 
 Avg Rank 
 

 
 Aln Len 
 

 
 Qry Len 
 

 
 IDOP? 
 

 
 adj? 
 

 

 

 
GKFG-790-MONOMER
 

 
bcv:Bcav_2038 carbamoyl-p
 

 
1.3958463e-4
 

 
3.0
 

 
Q7MGL1
 

 
6.08773e-4
 

 
1.0
 

 
0.31335223
 

 
377
 

 
T
 

 
NIL
 

 

 

 
GKFG-977-MONOMER
 

 
paz:TIA2EST2_02200 methyl
 

 
1.3958463e-4
 

 
1.0
 

 
P46701
 

 
0.229756
 

 
1.0
 

 
0.32346243
 

 
439
 

 
T
 

 
NIL
 

 

 

 
GKFG-1050-MONOMER
 

 
ach:Achl_1275 glycosyl tr
 

 
1.5421621e-5
 

 
1.0
 

 
O66608
 

 
0.242205
 

 
1.0
 

 
0.23013699
 

 
365
 

 
T
 

 
NIL
 

 

 

 
GKFG-251-MONOMER
 

 
LuxR family transcription
 

 
1.5421621e-5
 

 
1.0
 

 
O06457
 

 
0.878158
 

 
1.0
 

 
0.19178082
 

 
365
 

 
T
 

 
NIL
 

 

 

 
GKFG-362-MONOMER
 

 
histidine kinase (db=KEGG
 

 
1.5421621e-5
 

 
1.0
 

 
P52559
 

 
0.921985
 

 
1.0
 

 
0.12154696
 

 
362
 

 
T
 

 
NIL
 

 

 

 
GKFG-732-MONOMER
 

 
ahe:Arch_1078 LuxR family
 

 
1.5421621e-5
 

 
1.0
 

 
P74724
 

 
0.445368
 

 
1.0
 

 
0.14948453
 

 
388
 

 
T
 

 
NIL
 

 

 

 
GKFG-701-MONOMER
 

 
ach:Achl_1330 fibronectin
 

 
2.795795e-6
 

 
1.0
 

 
Q7MGL1
 

 
0.603538
 

 
2.0
 

 
0.20424403
 

 
377
 

 
T
 

 
NIL
 

 

 

 
GKFG-1464-MONOMER
 

 
mcu:HMPREF0573_11568 serA
 

 
2.5282066e-6
 

 
2.0
 

 
P72158
 

 
0.114415
 

 
2.0
 

 
0.083000004
 

 
360
 

 
T
 

 
NIL
 

 

 

 
GKFG-480-MONOMER
 

 
cfl:Cfla_0002 DNA polymer
 

 
2.5282066e-6
 

 
1.0
 

 
Q49WI9
 

 
0.296585
 

 
2.0
 

 
0.09066667
 

 
375
 

 
T
 

 
NIL
 

 

 
     

  pyrimidine ribonucleotides interconversion     Total # of reactions in pathway = 11   Present reactions: 3
  

 

 
 Reaction 
 

 
 Protein(s) 
 

 

 

 
CDPKIN-RXN
 

 
(# )
 

 

 

 
2.7.4.22-RXN
 

 
(# )
 

 

 

 
CMPKI-RXN
 

 
(# )
 

 

 
   Missing reactions: 8
  

 

 
 UDPKIN-RXN 
 

 

 

 
 CTPSYN-RXN 
 

 

 

 
 RXN-12200 
 

 

 

 
 RXN-12199 
 

 

 

 
 RXN-12198 
 

 

 

 
 RXN-12197 
 

 

 

 
 RXN-12196 
 

 

 

 
 RXN-12195 
 

 

 
        Evidence for , EC# 2.7.4.6   UDPKIN-RXN 
  

 

 
 Hit 
 

 
 Common 
 

 
 P 
 

 
 #Q 
 

 
 Best Qry 
 

 
 Best Eval 
 

 
 Avg Rank 
 

 
 Aln Len 
 

 
 Qry Len 
 

 
 IDOP? 
 

 
 adj? 
 

 

 

 
GKFG-689-MONOMER
 

 
ndk; nucleoside-diphospha
 

 
0.9968199
 

 
1.0
 

 
P0A763
 

 
6.57949e-32
 

 
1.0
 

 
0.951049
 

 
143
 

 
T
 

 
NIL
 

 

 
     Evidence for CTP synthase, EC# 6.3.4.2   CTPSYN-RXN 
  

 

 
 Hit 
 

 
 Common 
 

 
 P 
 

 
 #Q 
 

 
 Best Qry 
 

 
 Best Eval 
 

 
 Avg Rank 
 

 
 Aln Len 
 

 
 Qry Len 
 

 
 IDOP? 
 

 
 adj? 
 

 

 

 
GKFG-16-MONOMER
 

 
(db=HMMPfam db_id=PF05738
 

 
3.7905585e-4
 

 
1.0
 

 
B7IH88
 

 
0.0441708
 

 
1.0
 

 
0.27756655
 

 
526
 

 
NIL
 

 
NIL
 

 

 

 
GKFG-791-MONOMER
 

 
iva:Isova_1606 carbamoyl-
 

 
1.3958463e-4
 

 
5.0
 

 
Q9ZDF1
 

 
0.0162868
 

 
1.0
 

 
0.24080911
 

 
586
 

 
T
 

 
NIL
 

 

 

 
GKFG-1875-MONOMER
 

 
ahe:Arch_1100 glutamyl-tR
 

 
1.3958463e-4
 

 
1.0
 

 
Q98PQ3
 

 
0.0301851
 

 
1.0
 

 
0.30983302
 

 
539
 

 
T
 

 
NIL
 

 

 

 
GKFG-1661-MONOMER
 

 
aau:AAur_3325 gdhA; gluta
 

 
1.3958463e-4
 

 
1.0
 

 
Q93DW6
 

 
0.710726
 

 
1.0
 

 
0.34343433
 

 
99
 

 
T
 

 
NIL
 

 

 

 
GKFG-790-MONOMER
 

 
bcv:Bcav_2038 carbamoyl-p
 

 
1.5421621e-5
 

 
1.0
 

 
Q3B6P1
 

 
0.366192
 

 
1.0
 

 
0.1758794
 

 
597
 

 
T
 

 
NIL
 

 

 

 
GKFG-715-MONOMER
 

 
mcu:HMPREF0573_11674 glxK
 

 
1.5421621e-5
 

 
1.0
 

 
C1CWM4
 

 
0.0819759
 

 
1.0
 

 
0.19244604
 

 
556
 

 
T
 

 
NIL
 

 

 

 
GKFG-430-MONOMER
 

 
groES; co-chaperonin GroE
 

 
6.867209e-6
 

 
1.0
 

 
B9KHF1
 

 
0.962149
 

 
2.0
 

 
0.10535714
 

 
560
 

 
NIL
 

 
NIL
 

 

 

 
GKFG-387-MONOMER
 

 
cgt:cgR_2367 hypothetical
 

 
6.867209e-6
 

 
1.0
 

 
Q9ZDF1
 

 
0.440332
 

 
2.0
 

 
0.042662118
 

 
586
 

 
NIL
 

 
NIL
 

 

 

 
GKFG-1828-MONOMER
 

 
cfi:Celf_1839 DNA polymer
 

 
2.5282066e-6
 

 
1.0
 

 
Q939R0
 

 
0.180577
 

 
2.0
 

 
0.106571935
 

 
563
 

 
T
 

 
NIL
 

 

 

 
GKFG-1603-MONOMER
 

 
xce:Xcel_0763 hypothetica
 

 
2.5282066e-6
 

 
1.0
 

 
Q3B6P1
 

 
0.459358
 

 
2.0
 

 
0.07705192
 

 
597
 

 
T
 

 
NIL
 

 

 

 
GKFG-1827-MONOMER
 

 
AMP-dependent synthetase 
 

 
2.5282066e-6
 

 
1.0
 

 
Q98PQ3
 

 
0.683088
 

 
2.0
 

 
0.05380334
 

 
539
 

 
T
 

 
NIL
 

 

 

 
GKFG-1833-MONOMER
 

 
bcv:Bcav_2151 prephenate 
 

 
1.0926308e-6
 

 
1.0
 

 
Q3B6P1
 

 
0.679904
 

 
3.0
 

 
0.06365159
 

 
597
 

 
T
 

 
NIL
 

 

 
     Evidence for , EC# 3.6.1.5   RXN-12200 
  
no BLAST hits found
 
     Evidence for , EC# 3.6.1.5   RXN-12199 
  
no BLAST hits found
 
     Evidence for , EC# 3.6.1.6   RXN-12198      Evidence for , EC# 3.6.1.6   RXN-12197      Evidence for , EC# 3.6.1.15   RXN-12196      Evidence for , EC# 3.6.1.15   RXN-12195      

  pyrimidine deoxyribonucleotides  de novo  biosynthesis I     Total # of reactions in pathway = 13   Present reactions: 12
  

 

 
 Reaction 
 

 
 Protein(s) 
 

 

 

 
DCDPKIN-RXN
 

 
(# )
 

 

 

 
DCTP-DEAM-RXN
 

 
(# )
 

 

 

 
DUDPKIN-RXN
 

 
(# )
 

 

 

 
THYMIDYLATESYN-RXN
 

 
(# )
 

 

 

 
DTMPKI-RXN
 

 
(# )
 

 

 

 
DTDPKIN-RXN
 

 
(# )
 

 

 

 
UDPREDUCT-RXN
 

 
(# 
 # )
 

 

 

 
CDPREDUCT-RXN
 

 
NIL
 

 

 

 
RXN0-722
 

 
(# )
 

 

 

 
RIBONUCLEOSIDE-DIP-REDUCTII-RXN
 

 
(# )
 

 

 

 
RXN0-724
 

 
NIL
 

 

 

 
RXN0-723
 

 
NIL
 

 

 
   Missing reactions: 1
  

 

 
 DUTP-PYROP-RXN 
 

 

 
        Evidence for dUTP diphosphatase, EC# 3.6.1.23   DUTP-PYROP-RXN 
  

 

 
 Hit 
 

 
 Common 
 

 
 P 
 

 
 #Q 
 

 
 Best Qry 
 

 
 Best Eval 
 

 
 Avg Rank 
 

 
 Aln Len 
 

 
 Qry Len 
 

 
 IDOP? 
 

 
 adj? 
 

 

 

 
GKFG-1777-MONOMER
 

 
cur:cur_0492 surB; cell s
 

 
0.039642163
 

 
1.0
 

 
A8LKC5
 

 
0.111658
 

 
1.0
 

 
0.87096775
 

 
155
 

 
T
 

 
NIL
 

 

 

 
GKFG-1747-MONOMER
 

 
MerR family transcription
 

 
0.0051016076
 

 
1.0
 

 
Q6E4Q0
 

 
0.0429277
 

 
1.0
 

 
0.6503497
 

 
143
 

 
T
 

 
NIL
 

 

 

 
GKFG-961-MONOMER
 

 
2-succinyl-5-enolpyruvyl-
 

 
0.0018147272
 

 
1.0
 

 
Q7NKL2
 

 
0.660459
 

 
1.0
 

 
0.52380955
 

 
147
 

 
T
 

 
NIL
 

 

 

 
GKFG-1812-MONOMER
 

 
NUDIX hydrolase (db=KEGG 
 

 
0.0016251113
 

 
1.0
 

 
P52006
 

 
0.0893637
 

 
2.0
 

 
0.7659575
 

 
141
 

 
T
 

 
NIL
 

 

 

 
GKFG-141-MONOMER
 

 
dsy:DSY3811 hypothetical 
 

 
3.7905585e-4
 

 
2.0
 

 
A8IPW5
 

 
0.206499
 

 
1.0
 

 
0.3214873
 

 
157
 

 
NIL
 

 
NIL
 

 

 

 
GKFG-55-MONOMER
 

 
coiled-coil (db=Coil db_i
 

 
3.7905585e-4
 

 
1.0
 

 
B3DRA9
 

 
0.0398757
 

 
1.0
 

 
0.25949368
 

 
158
 

 
NIL
 

 
NIL
 

 

 

 
GKFG-51-MONOMER
 

 
seg (db=Seg db_id=seg fro
 

 
3.7905585e-4
 

 
1.0
 

 
Q6MEK7
 

 
0.142482
 

 
1.0
 

 
0.34666666
 

 
150
 

 
NIL
 

 
NIL
 

 

 

 
GKFG-1601-MONOMER
 

 
cfi:Celf_2888 hypothetica
 

 
3.294785e-4
 

 
1.0
 

 
B8FQZ6
 

 
0.527154
 

 
2.0
 

 
0.49006623
 

 
151
 

 
T
 

 
NIL
 

 

 

 
GKFG-1027-MONOMER
 

 
cfi:Celf_2525 mannose-6-p
 

 
3.182795e-4
 

 
2.0
 

 
Q6AFE0
 

 
0.11298
 

 
1.0
 

 
0.4243421
 

 
152
 

 
T
 

 
NIL
 

 

 

 
GKFG-1800-MONOMER
 

 
bcv:Bcav_1999 (p)ppGpp sy
 

 
3.182795e-4
 

 
1.0
 

 
P31625
 

 
0.11982
 

 
1.0
 

 
0.43944636
 

 
289
 

 
T
 

 
NIL
 

 

 

 
GKFG-1493-MONOMER
 

 
gvh:HMPREF9231_0059 dihyd
 

 
3.182795e-4
 

 
1.0
 

 
O31801
 

 
0.20118
 

 
1.0
 

 
0.47916666
 

 
144
 

 
T
 

 
NIL
 

 

 

 
GKFG-100-MONOMER
 

 
mcu:HMPREF0573_11536 ABC 
 

 
1.5675386e-4
 

 
1.0
 

 
B3DRA9
 

 
0.203132
 

 
2.0
 

 
0.43670887
 

 
158
 

 
NIL
 

 
NIL
 

 

 

 
GKFG-1012-MONOMER
 

 
krh:KRH_10210 ilvB; aceto
 

 
1.3958463e-4
 

 
1.0
 

 
Q91F87
 

 
0.0519787
 

 
1.0
 

 
0.27027026
 

 
296
 

 
T
 

 
NIL
 

 

 

 
GKFG-1180-MONOMER
 

 
cde:CDHC02_1634 N-acetylm
 

 
1.3958463e-4
 

 
1.0
 

 
Q6FYR2
 

 
0.248107
 

 
1.0
 

 
0.2972973
 

 
185
 

 
T
 

 
NIL
 

 

 

 
GKFG-1209-MONOMER
 

 
hypothetical protein (db=
 

 
1.3958463e-4
 

 
1.0
 

 
B8FQZ6
 

 
0.478645
 

 
1.0
 

 
0.3576159
 

 
151
 

 
T
 

 
NIL
 

 

 

 
GKFG-760-MONOMER
 

 
iva:Isova_1293 cell divis
 

 
1.3958463e-4
 

 
1.0
 

 
Q10FF9
 

 
0.675632
 

 
1.0
 

 
0.24561404
 

 
171
 

 
T
 

 
NIL
 

 

 

 
GKFG-1528-MONOMER
 

 
ROK family protein (db=KE
 

 
1.3958463e-4
 

 
1.0
 

 
A7N9S0
 

 
0.219305
 

 
1.0
 

 
0.3310811
 

 
148
 

 
T
 

 
NIL
 

 

 

 
GKFG-553-MONOMER
 

 
NUDIX hydrolase (db=KEGG 
 

 
2.5307965e-5
 

 
2.0
 

 
P52006
 

 
0.00140659
 

 
1.5
 

 
0.34223577
 

 
141
 

 
T
 

 
NIL
 

 

 

 
GKFG-1069-MONOMER
 

 
abortive infection protei
 

 
2.5307965e-5
 

 
1.0
 

 
Q6AFE0
 

 
0.707001
 

 
2.0
 

 
0.33552632
 

 
152
 

 
T
 

 
NIL
 

 

 

 
GKFG-649-MONOMER
 

 
hypothetical protein (db=
 

 
2.4944004e-5
 

 
1.0
 

 
P52006
 

 
0.493914
 

 
3.0
 

 
0.42553192
 

 
141
 

 
T
 

 
NIL
 

 

 

 
GKFG-1242-MONOMER
 

 
cfi:Celf_2761 alpha amyla
 

 
1.5421621e-5
 

 
1.0
 

 
Q6BRN7
 

 
0.154707
 

 
1.0
 

 
0.19375
 

 
160
 

 
T
 

 
NIL
 

 

 

 
GKFG-1874-MONOMER
 

 
kra:Krad_0299 hisS; histi
 

 
1.5421621e-5
 

 
1.0
 

 
P33316
 

 
0.835155
 

 
1.0
 

 
0.21428572
 

 
252
 

 
T
 

 
NIL
 

 

 

 
GKFG-1558-MONOMER
 

 
seg (db=Seg db_id=seg fro
 

 
1.5421621e-5
 

 
1.0
 

 
P28892
 

 
0.458418
 

 
1.0
 

 
0.1595092
 

 
326
 

 
T
 

 
NIL
 

 

 

 
GKFG-843-MONOMER
 

 
mcu:HMPREF0573_11102 atpD
 

 
1.5421621e-5
 

 
1.0
 

 
Q4JQW7
 

 
0.11449
 

 
1.0
 

 
0.19949494
 

 
396
 

 
T
 

 
NIL
 

 

 

 
GKFG-1173-MONOMER
 

 
UniRef90_C2KNW0 Conserved
 

 
1.5421621e-5
 

 
1.0
 

 
Q9YYS0
 

 
0.538193
 

 
1.0
 

 
0.20245399
 

 
163
 

 
T
 

 
NIL
 

 

 

 
GKFG-864-MONOMER
 

 
crd:CRES_0981 pip; prolin
 

 
1.3945618e-5
 

 
2.0
 

 
P16088
 

 
6.08767e-4
 

 
1.0
 

 
0.0942195
 

 
1124
 

 
T
 

 
NIL
 

 

 

 
GKFG-1222-MONOMER
 

 
hypothetical protein (db=
 

 
2.5282066e-6
 

 
1.0
 

 
P31625
 

 
0.353446
 

 
2.0
 

 
0.10380623
 

 
289
 

 
T
 

 
NIL
 

 

 
     

  glycine betaine biosynthesis II (Gram-positive bacteria)     Total # of reactions in pathway = 2   Present reactions: 1
  

 

 
 Reaction 
 

 
 Protein(s) 
 

 

 

 
RXN-6021
 

 
(# 
 # )
 

 

 
   Missing reactions: 1
  

 

 
 BADH-RXN 
 

 

 
        Evidence for betaine-aldehyde dehydrogenase, EC# 1.2.1.8   BADH-RXN 
  

 

 
 Hit 
 

 
 Common 
 

 
 P 
 

 
 #Q 
 

 
 Best Qry 
 

 
 Best Eval 
 

 
 Avg Rank 
 

 
 Aln Len 
 

 
 Qry Len 
 

 
 IDOP? 
 

 
 adj? 
 

 

 

 
GKFG-977-MONOMER
 

 
paz:TIA2EST2_02200 methyl
 

 
0.99739957
 

 
8.0
 

 
P17445
 

 
4.58405e-70
 

 
1.875
 

 
0.9406296
 

 
490
 

 
T
 

 
NIL
 

 

 

 
GKFG-1235-MONOMER
 

 
msm:MSMEG_0582 succinate-
 

 
0.9915977
 

 
8.0
 

 
Q8UH56
 

 
1.69019e-80
 

 
1.125
 

 
0.9322703
 

 
493
 

 
T
 

 
NIL
 

 

 

 
GKFG-328-MONOMER
 

 
mcu:HMPREF0573_11678 L-pr
 

 
0.95936644
 

 
8.0
 

 
O59808
 

 
5.33826e-32
 

 
4.0
 

 
0.7685056
 

 
500
 

 
NIL
 

 
NIL
 

 

 

 
GKFG-997-MONOMER
 

 
ssm:Spirs_0789 aldehyde d
 

 
0.86394465
 

 
8.0
 

 
P56533
 

 
8.2679e-46
 

 
3.0
 

 
0.77759314
 

 
503
 

 
T
 

 
NIL
 

 

 

 
GKFG-1001-MONOMER
 

 
faa:HMPREF0389_01374 hypo
 

 
9.067587e-5
 

 
1.0
 

 
Q8P5D8
 

 
0.00720292
 

 
5.0
 

 
0.46734694
 

 
490
 

 
T
 

 
NIL
 

 

 

 
GKFG-87-MONOMER
 

 
173553..173783 - ( gc_con
 

 
1.0789291e-5
 

 
1.0
 

 
P17445
 

 
0.466349
 

 
5.0
 

 
0.118367344
 

 
490
 

 
NIL
 

 
NIL
 

 

 

 
GKFG-1606-MONOMER
 

 
bcv:Bcav_2913 MgtE integr
 

 
1.0040111e-5
 

 
1.0
 

 
Q8P5D8
 

 
0.382938
 

 
6.0
 

 
0.23061225
 

 
490
 

 
T
 

 
NIL
 

 

 

 
GKFG-1965-MONOMER
 

 
hypothetical protein Nest
 

 
3.972156e-6
 

 
1.0
 

 
Q8UH56
 

 
0.520901
 

 
5.0
 

 
0.070993915
 

 
493
 

 
T
 

 
NIL
 

 

 

 
GKFG-1161-MONOMER
 

 
ahe:Arch_0727 methionine 
 

 
3.972156e-6
 

 
1.0
 

 
O59808
 

 
0.324418
 

 
5.0
 

 
0.106
 

 
500
 

 
T
 

 
NIL
 

 

 
     

   myo -inositol degradation     Total # of reactions in pathway = 6   Present reactions: 3
  

 

 
 Reaction 
 

 
 Protein(s) 
 

 

 

 
MYO-INOSITOL-2-DEHYDROGENASE-RXN
 

 
(# 
 # 
 # 
 # )
 

 

 

 
MYO-INOSOSE-2-DEHYDRATASE-RXN
 

 
(# 
 # )
 

 

 

 
5-DEHYDRO-2-DEOXYGLUCONOKINASE-RXN
 

 
(# )
 

 

 
   Missing reactions: 3
  

 

 
 R503-RXN 
 

 

 

 
 4.1.2.29-RXN 
 

 

 

 
 RXN-2902 
 

 

 
        Evidence for , EC# 3.7.1.-   R503-RXN      Evidence for 5-dehydro-2-deoxyphosphogluconate aldolase, EC# 4.1.2.29   4.1.2.29-RXN 
  

 

 
 Hit 
 

 
 Common 
 

 
 P 
 

 
 #Q 
 

 
 Best Qry 
 

 
 Best Eval 
 

 
 Avg Rank 
 

 
 Aln Len 
 

 
 Qry Len 
 

 
 IDOP? 
 

 
 adj? 
 

 

 

 
GKFG-801-MONOMER
 

 
ahe:Arch_0192 fructose-bi
 

 
0.8249092
 

 
2.0
 

 
Q65D09
 

 
3.03565e-19
 

 
1.0
 

 
1.0
 

 
292
 

 
T
 

 
NIL
 

 

 

 
GKFG-1625-MONOMER
 

 
cga:Celgi_2334 2-oxogluta
 

 
2.5307965e-5
 

 
1.0
 

 
Q65D09
 

 
0.111915
 

 
2.0
 

 
0.34931508
 

 
292
 

 
T
 

 
NIL
 

 

 
     Evidence for , EC# 1.2.1.18   RXN-2902      

  glycerol degradation I     Total # of reactions in pathway = 2   Present reactions: 1
  

 

 
 Reaction 
 

 
 Protein(s) 
 

 

 

 
GLYCEROL-KIN-RXN
 

 
(# )
 

 

 
   Missing reactions: 1
  

 

 
 RXN0-5258 
 

 

 
        Evidence for sn-glycerol 3-phosphate:ubiquinone oxidoreductase, EC# 1.1.5.3   RXN0-5258 
  

 

 
 Hit 
 

 
 Common 
 

 
 P 
 

 
 #Q 
 

 
 Best Qry 
 

 
 Best Eval 
 

 
 Avg Rank 
 

 
 Aln Len 
 

 
 Qry Len 
 

 
 IDOP? 
 

 
 adj? 
 

 

 

 
GKFG-1193-MONOMER
 

 
pfr:PFREUD_12990 glpA; an
 

 
0.5596166
 

 
1.0
 

 
P13035
 

 
2.61493e-24
 

 
1.0
 

 
0.8223553
 

 
501
 

 
T
 

 
NIL
 

 

 
     

  arginine degradation X (arginine monooxygenase pathway)     Total # of reactions in pathway = 3   Present reactions: 1
  

 

 
 Reaction 
 

 
 Protein(s) 
 

 

 

 
GUANIDINOBUTANAMIDE-NH3-RXN
 

 
(# )
 

 

 
   Missing reactions: 2
  

 

 
 GUANIDINOBUTYRASE-RXN 
 

 

 

 
 ARGININE-2-MONOOXYGENASE-RXN 
 

 

 
        Evidence for guanidinobutyrase, EC# 3.5.3.7   GUANIDINOBUTYRASE-RXN 
  
no BLAST hits found
 
     Evidence for arginine 2-monooxygenase, EC# 1.13.12.1   ARGININE-2-MONOOXYGENASE-RXN      

  isoleucine degradation I     Total # of reactions in pathway = 6   Present reactions: 3
  

 

 
 Reaction 
 

 
 Protein(s) 
 

 

 

 
BRANCHED-CHAINAMINOTRANSFERILEU-RXN
 

 
(# )
 

 

 

 
METHYLACETOACETYLCOATHIOL-RXN
 

 
(# )
 

 

 

 
2-METHYLACYL-COA-DEHYDROGENASE-RXN
 

 
(# )
 

 

 
   Missing reactions: 3
  

 

 
 1.1.1.178-RXN 
 

 

 

 
 TIGLYLCOA-HYDROXY-RXN 
 

 

 

 
 2KETO-3METHYLVALERATE-RXN 
 

 

 
        Evidence for 3-hydroxy-2-methylbutyryl-CoA dehydrogenase, EC# 1.1.1.178   1.1.1.178-RXN 
  

 

 
 Hit 
 

 
 Common 
 

 
 P 
 

 
 #Q 
 

 
 Best Qry 
 

 
 Best Eval 
 

 
 Avg Rank 
 

 
 Aln Len 
 

 
 Qry Len 
 

 
 IDOP? 
 

 
 adj? 
 

 

 

 
GKFG-720-MONOMER
 

 
fabG; 3-ketoacyl-ACP redu
 

 
0.8538447
 

 
2.0
 

 
O02691
 

 
6.32325e-29
 

 
1.0
 

 
1.0
 

 
261
 

 
T
 

 
NIL
 

 

 

 
GKFG-803-MONOMER
 

 
short-chain dehydrogenase
 

 
0.007427732
 

 
2.0
 

 
O02691
 

 
2.45176e-10
 

 
2.0
 

 
0.9594095
 

 
261
 

 
T
 

 
NIL
 

 

 

 
GKFG-246-MONOMER
 

 
bcv:Bcav_0731 Inositol 2-
 

 
2.9708795e-5
 

 
1.0
 

 
O18404
 

 
0.0177281
 

 
3.0
 

 
0.2784314
 

 
255
 

 
NIL
 

 
NIL
 

 

 
     Evidence for Tiglyl-CoA hydratase, EC# 4.2.1.17   TIGLYLCOA-HYDROXY-RXN 
  

 

 
 Hit 
 

 
 Common 
 

 
 P 
 

 
 #Q 
 

 
 Best Qry 
 

 
 Best Eval 
 

 
 Avg Rank 
 

 
 Aln Len 
 

 
 Qry Len 
 

 
 IDOP? 
 

 
 adj? 
 

 

 

 
GKFG-964-MONOMER
 

 
pak:HMPREF0675_3964 menB;
 

 
0.8249092
 

 
1.0
 

 
Q58DM8
 

 
1.38228e-16
 

 
1.0
 

 
0.9896552
 

 
290
 

 
T
 

 
NIL
 

 

 
     Evidence for    2KETO-3METHYLVALERATE-RXN 
  

 

 
 Hit 
 

 
 Common 
 

 
 P 
 

 
 #Q 
 

 
 Best Qry 
 

 
 Best Eval 
 

 
 Avg Rank 
 

 
 Aln Len 
 

 
 Qry Len 
 

 
 IDOP? 
 

 
 adj? 
 

 

 

 
GKFG-1625-MONOMER
 

 
cga:Celgi_2334 2-oxogluta
 

 
0.036253624
 

 
4.0
 

 
P37942
 

 
1.25142e-18
 

 
1.25
 

 
0.4813445
 

 
424
 

 
T
 

 
NIL
 

 

 

 
GKFG-566-MONOMER
 

 
cfl:Cfla_1720 deoxyxylulo
 

 
0.0018147272
 

 
1.0
 

 
P21953
 

 
1.68222e-5
 

 
1.0
 

 
0.5
 

 
392
 

 
T
 

 
NIL
 

 

 

 
GKFG-669-MONOMER
 

 
ahe:Arch_1322 alkyl hydro
 

 
1.3958463e-4
 

 
1.0
 

 
P21880
 

 
1.14734e-6
 

 
1.0
 

 
0.3148936
 

 
470
 

 
T
 

 
NIL
 

 

 

 
GKFG-1966-MONOMER
 

 
mcu:HMPREF0573_11649 hypo
 

 
2.5307965e-5
 

 
1.0
 

 
P21880
 

 
8.32113e-4
 

 
2.0
 

 
0.32765958
 

 
470
 

 
T
 

 
NIL
 

 

 

 
GKFG-549-MONOMER
 

 
iva:Isova_3010 thioredoxi
 

 
6.867209e-6
 

 
2.0
 

 
P09622
 

 
3.26875e-4
 

 
2.0
 

 
0.081845924
 

 
509
 

 
NIL
 

 
NIL
 

 

 
     

  citrulline degradation     Total # of reactions in pathway = 2   Present reactions: 1
  

 

 
 Reaction 
 

 
 Protein(s) 
 

 

 

 
ORNCARBAMTRANSFER-RXN
 

 
(# )
 

 

 
   Missing reactions: 1
  

 

 
 CARBAMATE-KINASE-RXN 
 

 

 
        Evidence for carbamate kinase, EC# 2.7.2.2   CARBAMATE-KINASE-RXN 
  

 

 
 Hit 
 

 
 Common 
 

 
 P 
 

 
 #Q 
 

 
 Best Qry 
 

 
 Best Eval 
 

 
 Avg Rank 
 

 
 Aln Len 
 

 
 Qry Len 
 

 
 IDOP? 
 

 
 adj? 
 

 

 

 
GKFG-900-MONOMER
 

 
ahe:Arch_1354 electron-tr
 

 
1.5421621e-5
 

 
1.0
 

 
Q97C45
 

 
0.554267
 

 
1.0
 

 
0.21639344
 

 
305
 

 
T
 

 
NIL
 

 

 

 
GKFG-933-MONOMER
 

 
HAD-superfamily hydrolase
 

 
1.3945618e-5
 

 
1.0
 

 
Q8G997
 

 
0.864432
 

 
1.0
 

 
0.11320755
 

 
318
 

 
T
 

 
NIL
 

 

 
     

  proline degradation     Total # of reactions in pathway = 2   Present reactions: 1
  

 

 
 Reaction 
 

 
 Protein(s) 
 

 

 

 
RXN-821
 

 
(# )
 

 

 
   Missing reactions: 1
  

 

 
 PYRROLINECARBDEHYDROG-RXN 
 

 

 
        Evidence for 1-pyrroline-5-carboxylate dehydrogenase, EC# 1.5.1.12   PYRROLINECARBDEHYDROG-RXN 
  

 

 
 Hit 
 

 
 Common 
 

 
 P 
 

 
 #Q 
 

 
 Best Qry 
 

 
 Best Eval 
 

 
 Avg Rank 
 

 
 Aln Len 
 

 
 Qry Len 
 

 
 IDOP? 
 

 
 adj? 
 

 

 

 
GKFG-328-MONOMER
 

 
mcu:HMPREF0573_11678 L-pr
 

 
0.9901008
 

 
2.0
 

 
P09546
 

 
1.77619e-50
 

 
2.0
 

 
0.5540942
 

 
1320
 

 
T
 

 
T
 

 

 

 
GKFG-1235-MONOMER
 

 
msm:MSMEG_0582 succinate-
 

 
0.81371343
 

 
2.0
 

 
P09546
 

 
6.92303e-53
 

 
1.0
 

 
0.5794598
 

 
1320
 

 
NIL
 

 
NIL
 

 

 

 
GKFG-977-MONOMER
 

 
paz:TIA2EST2_02200 methyl
 

 
0.17620532
 

 
2.0
 

 
P09546
 

 
4.32846e-31
 

 
3.0
 

 
0.5190909
 

 
1320
 

 
NIL
 

 
NIL
 

 

 

 
GKFG-997-MONOMER
 

 
ssm:Spirs_0789 aldehyde d
 

 
0.055755496
 

 
2.0
 

 
P09546
 

 
1.84032e-20
 

 
4.0
 

 
0.5280303
 

 
1320
 

 
NIL
 

 
NIL
 

 

 

 
GKFG-521-MONOMER
 

 
UniRef90_E6KT22 Stage 0 D
 

 
1.193123e-5
 

 
1.0
 

 
P07275
 

 
0.0955568
 

 
5.0
 

 
0.13391304
 

 
575
 

 
NIL
 

 
NIL
 

 

 
     

  tyrosine degradation III     Total # of reactions in pathway = 4   Present reactions: 1
  

 

 
 Reaction 
 

 
 Protein(s) 
 

 

 

 
RXN3O-4113
 

 
(# 
 # )
 

 

 
   Missing reactions: 3
  

 

 
 TYRAMINOTRANS-RXN 
 

 

 

 
 4.1.1.80-RXN 
 

 

 

 
 RXN3O-4157 
 

 

 
        Evidence for Tyrosine transaminase, EC# 2.6.1.57   TYRAMINOTRANS-RXN 
  

 

 
 Hit 
 

 
 Common 
 

 
 P 
 

 
 #Q 
 

 
 Best Qry 
 

 
 Best Eval 
 

 
 Avg Rank 
 

 
 Aln Len 
 

 
 Qry Len 
 

 
 IDOP? 
 

 
 adj? 
 

 

 

 
GKFG-510-MONOMER
 

 
bcv:Bcav_3969 histidinol-
 

 
3.7905585e-4
 

 
1.0
 

 
P04693
 

 
0.54132
 

 
1.0
 

 
0.24685138
 

 
397
 

 
NIL
 

 
NIL
 

 

 
     Evidence for 4-hydroxyphenylpyruvate decarboxylase, EC# 4.1.1.80   4.1.1.80-RXN 
  

 

 
 Hit 
 

 
 Common 
 

 
 P 
 

 
 #Q 
 

 
 Best Qry 
 

 
 Best Eval 
 

 
 Avg Rank 
 

 
 Aln Len 
 

 
 Qry Len 
 

 
 IDOP? 
 

 
 adj? 
 

 

 

 
GKFG-1493-MONOMER
 

 
gvh:HMPREF9231_0059 dihyd
 

 
0.8538447
 

 
2.0
 

 
C4WE40
 

 
1.31242e-28
 

 
1.0
 

 
0.9431388
 

 
344
 

 
T
 

 
NIL
 

 

 

 
GKFG-961-MONOMER
 

 
2-succinyl-5-enolpyruvyl-
 

 
2.4944004e-5
 

 
1.0
 

 
C4WE40
 

 
0.178079
 

 
3.0
 

 
0.47093022
 

 
344
 

 
T
 

 
NIL
 

 

 

 
GKFG-1954-MONOMER
 

 
bcv:Bcav_2235 Imidazole g
 

 
2.795795e-6
 

 
2.0
 

 
C0G532
 

 
0.0597395
 

 
2.0
 

 
0.22499716
 

 
307
 

 
T
 

 
NIL
 

 

 
     Evidence for , EC# 2.6.1.58   RXN3O-4157 
  
no BLAST hits found
 
     

  valine degradation II     Total # of reactions in pathway = 3   Present reactions: 2
  

 

 
 Reaction 
 

 
 Protein(s) 
 

 

 

 
BRANCHED-CHAINAMINOTRANSFERVAL-RXN
 

 
(# )
 

 

 

 
RXN-7657
 

 
(# 
 # )
 

 

 
   Missing reactions: 1
  

 

 
 RXN-7643 
 

 

 
        Evidence for , EC# 4.1.1.1   RXN-7643 
  

 

 
 Hit 
 

 
 Common 
 

 
 P 
 

 
 #Q 
 

 
 Best Qry 
 

 
 Best Eval 
 

 
 Avg Rank 
 

 
 Aln Len 
 

 
 Qry Len 
 

 
 IDOP? 
 

 
 adj? 
 

 

 

 
GKFG-1012-MONOMER
 

 
krh:KRH_10210 ilvB; aceto
 

 
0.8249092
 

 
4.0
 

 
tr|Q684J7|Q684J7_LACLL
 

 
9.45932e-18
 

 
1.0
 

 
0.9118164
 

 
548
 

 
T
 

 
NIL
 

 

 

 
GKFG-423-MONOMER
 

 
pfr:PFREUD_19090 iolD; ac
 

 
0.22371992
 

 
4.0
 

 
P16467
 

 
1.38219e-13
 

 
2.0
 

 
0.6366044
 

 
563
 

 
NIL
 

 
NIL
 

 

 

 
GKFG-29-MONOMER
 

 
std:SPPN_11055 Zinc metal
 

 
2.9678517e-6
 

 
2.0
 

 
P06169
 

 
0.0323221
 

 
3.0
 

 
0.06394316
 

 
563
 

 
NIL
 

 
NIL
 

 

 

 
GKFG-809-MONOMER
 

 
ahe:Arch_0180 ATP-depende
 

 
1.6156636e-6
 

 
1.0
 

 
P06169
 

 
0.905685
 

 
4.0
 

 
0.13854352
 

 
563
 

 
T
 

 
NIL
 

 

 
     

  CO 2  fixation into oxaloacetate (anapleurotic)     Total # of reactions in pathway = 2   Present reactions: 1
  

 

 
 Reaction 
 

 
 Protein(s) 
 

 

 

 
RXN0-5224
 

 
(# )
 

 

 
   Missing reactions: 1
  

 

 
 PEPCARBOX-RXN 
 

 

 
        Evidence for phospho enol pyruvate carboxylase, EC# 4.1.1.31   PEPCARBOX-RXN 
  

 

 
 Hit 
 

 
 Common 
 

 
 P 
 

 
 #Q 
 

 
 Best Qry 
 

 
 Best Eval 
 

 
 Avg Rank 
 

 
 Aln Len 
 

 
 Qry Len 
 

 
 IDOP? 
 

 
 adj? 
 

 

 

 
GKFG-289-MONOMER
 

 
mcu:HMPREF0573_10751 puta
 

 
1.5421621e-5
 

 
1.0
 

 
A8AW99
 

 
0.703679
 

 
1.0
 

 
0.21518987
 

 
948
 

 
T
 

 
NIL
 

 

 

 
GKFG-142-MONOMER
 

 
dsy:DSY3812 hypothetical 
 

 
1.3945618e-5
 

 
1.0
 

 
Q8PS70
 

 
0.667179
 

 
1.0
 

 
0.10076046
 

 
526
 

 
T
 

 
NIL
 

 

 

 
GKFG-1465-MONOMER
 

 
iva:Isova_1268 UvrD/REP h
 

 
1.5421621e-5
 

 
1.0
 

 
A4VN44
 

 
0.215022
 

 
1.0
 

 
0.1592719
 

 
879
 

 
T
 

 
NIL
 

 

 

 
GKFG-1767-MONOMER
 

 
hypothetical protein (db=
 

 
1.5421621e-5
 

 
1.0
 

 
Q0STS8
 

 
0.046729
 

 
1.0
 

 
0.1359404
 

 
537
 

 
T
 

 
NIL
 

 

 

 
GKFG-594-MONOMER
 

 
hypothetical protein (db=
 

 
1.5421621e-5
 

 
1.0
 

 
B0R7F9
 

 
0.884405
 

 
1.0
 

 
0.14634146
 

 
492
 

 
T
 

 
NIL
 

 

 

 
GKFG-765-MONOMER
 

 
cfl:Cfla_2325 Preprotein 
 

 
1.5421621e-5
 

 
1.0
 

 
Q8TZL5
 

 
0.14614
 

 
1.0
 

 
0.17372881
 

 
472
 

 
T
 

 
NIL
 

 

 

 
GKFG-1046-MONOMER
 

 
xce:Xcel_0723 ATP-depende
 

 
1.3945618e-5
 

 
1.0
 

 
O32483
 

 
0.139123
 

 
1.0
 

 
0.053418804
 

 
936
 

 
T
 

 
NIL
 

 

 

 
GKFG-1621-MONOMER
 

 
tcu:Tcur_3714 3-phosphosh
 

 
1.3945618e-5
 

 
1.0
 

 
O28786
 

 
0.102436
 

 
1.0
 

 
0.10403397
 

 
471
 

 
T
 

 
NIL
 

 

 

 
GKFG-739-MONOMER
 

 
bcv:Bcav_1600 DEAD/DEAH b
 

 
1.3945618e-5
 

 
1.0
 

 
A1R2V3
 

 
0.792382
 

 
1.0
 

 
0.04828326
 

 
932
 

 
T
 

 
NIL
 

 

 

 
GKFG-1304-MONOMER
 

 
UniRef90_UPI000051013A hy
 

 
1.3945618e-5
 

 
1.0
 

 
Q5SKL7
 

 
0.671567
 

 
1.0
 

 
0.09440559
 

 
858
 

 
T
 

 
NIL
 

 

 

 
GKFG-732-MONOMER
 

 
ahe:Arch_1078 LuxR family
 

 
1.3945618e-5
 

 
1.0
 

 
Q2SL23
 

 
0.350988
 

 
1.0
 

 
0.08399546
 

 
881
 

 
T
 

 
NIL
 

 

 

 
GKFG-1347-MONOMER
 

 
aur:HMPREF9243_0883 hypot
 

 
1.3945618e-5
 

 
1.0
 

 
A4JGV5
 

 
0.0294267
 

 
1.0
 

 
0.055165496
 

 
997
 

 
T
 

 
NIL
 

 

 

 
GKFG-1068-MONOMER
 

 
ckp:ckrop_1874 hypothetic
 

 
1.3945618e-5
 

 
1.0
 

 
Q93MH3
 

 
0.252621
 

 
1.0
 

 
0.054406963
 

 
919
 

 
T
 

 
NIL
 

 

 

 
GKFG-86-MONOMER
 

 
Putative uncharacterized 
 

 
2.5282066e-6
 

 
2.0
 

 
O32483
 

 
0.238824
 

 
1.5
 

 
0.09476648
 

 
936
 

 
T
 

 
NIL
 

 

 

 
GKFG-177-MONOMER
 

 
ubiE; methylase; K03183 u
 

 
1.0926308e-6
 

 
1.0
 

 
O32483
 

 
0.310147
 

 
3.0
 

 
0.086538464
 

 
936
 

 
T
 

 
NIL
 

 

 

 
GKFG-1383-MONOMER
 

 
jde:Jden_1695 DEAD/DEAH b
 

 
2.5282066e-6
 

 
2.0
 

 
A4JGV5
 

 
0.0496707
 

 
1.5
 

 
0.070196435
 

 
997
 

 
T
 

 
NIL
 

 

 

 
GKFG-844-MONOMER
 

 
ahe:Arch_0366 ATP synthas
 

 
2.5282066e-6
 

 
1.0
 

 
A8AW99
 

 
0.828791
 

 
2.0
 

 
0.07489452
 

 
948
 

 
T
 

 
NIL
 

 

 
     

  reductive monocarboxylic acid cycle     Total # of reactions in pathway = 2   Present reactions: 1
  

 

 
 Reaction 
 

 
 Protein(s) 
 

 

 

 
PYRUFLAVREDUCT-RXN
 

 
(# 
 # 
 # )
 

 

 
   Missing reactions: 1
  

 

 
 PYRUVFORMLY-RXN 
 

 

 
        Evidence for formate  C -acetyltransferase, EC# 2.3.1.54   PYRUVFORMLY-RXN 
  

 

 
 Hit 
 

 
 Common 
 

 
 P 
 

 
 #Q 
 

 
 Best Qry 
 

 
 Best Eval 
 

 
 Avg Rank 
 

 
 Aln Len 
 

 
 Qry Len 
 

 
 IDOP? 
 

 
 adj? 
 

 

 

 
GKFG-1796-MONOMER
 

 
pac:PPA0489 UTP-glucose-1
 

 
3.787886e-5
 

 
1.0
 

 
P43753
 

 
0.742193
 

 
1.0
 

 
0.10519481
 

 
770
 

 
NIL
 

 
NIL
 

 

 

 
GKFG-347-MONOMER
 

 
ahe:Arch_0333 N-acetylglu
 

 
1.3945618e-5
 

 
1.0
 

 
P42632
 

 
0.619868
 

 
1.0
 

 
0.107329845
 

 
764
 

 
T
 

 
NIL
 

 

 
     

  formaldehyde oxidation II (glutathione-dependent)     Total # of reactions in pathway = 2   Present reactions: 1
  

 

 
 Reaction 
 

 
 Protein(s) 
 

 

 

 
RXN-2962
 

 
(# )
 

 

 
   Missing reactions: 1
  

 

 
 S-FORMYLGLUTATHIONE-HYDROLASE-RXN 
 

 

 
        Evidence for  S -formylglutathione hydrolase, EC# 3.1.2.12   S-FORMYLGLUTATHIONE-HYDROLASE-RXN 
  

 

 
 Hit 
 

 
 Common 
 

 
 P 
 

 
 #Q 
 

 
 Best Qry 
 

 
 Best Eval 
 

 
 Avg Rank 
 

 
 Aln Len 
 

 
 Qry Len 
 

 
 IDOP? 
 

 
 adj? 
 

 

 

 
GKFG-1169-MONOMER
 

 
UniRef90_E6M3C9 Acyl-CoA 
 

 
3.3353957e-5
 

 
1.0
 

 
A1AXZ2
 

 
0.824361
 

 
4.0
 

 
0.45519713
 

 
279
 

 
T
 

 
NIL
 

 

 

 
GKFG-1919-MONOMER
 

 
putative acyl-CoA thioest
 

 
1.5421621e-5
 

 
2.0
 

 
A1AXZ2
 

 
0.0150541
 

 
1.0
 

 
0.17271587
 

 
279
 

 
T
 

 
NIL
 

 

 

 
GKFG-1861-MONOMER
 

 
esterase (db=KEGG evalue=
 

 
1.09376415e-5
 

 
1.0
 

 
A1AXZ2
 

 
0.324007
 

 
3.0
 

 
0.26881722
 

 
279
 

 
T
 

 
NIL
 

 

 

 
GKFG-1906-MONOMER
 

 
mcu:HMPREF0573_11357 puta
 

 
2.795795e-6
 

 
1.0
 

 
A1AXZ2
 

 
0.0713502
 

 
2.0
 

 
0.12903225
 

 
279
 

 
T
 

 
NIL
 

 

 
     

  acrylate degradation     Total # of reactions in pathway = 4   Present reactions: 1
  

 

 
 Reaction 
 

 
 Protein(s) 
 

 

 

 
RXN-9958
 

 
(# )
 

 

 
   Missing reactions: 3
  

 

 
 3-HYDROXYPROPIONATE-DEHYDROGENASE-RXN 
 

 

 

 
 RXN-10986 
 

 

 

 
 RXN-10985 
 

 

 
        Evidence for 3-hydroxypropionate dehydrogenase, EC# 1.1.1.59   3-HYDROXYPROPIONATE-DEHYDROGENASE-RXN 
  

 

 
 Hit 
 

 
 Common 
 

 
 P 
 

 
 #Q 
 

 
 Best Qry 
 

 
 Best Eval 
 

 
 Avg Rank 
 

 
 Aln Len 
 

 
 Qry Len 
 

 
 IDOP? 
 

 
 adj? 
 

 

 

 
GKFG-900-MONOMER
 

 
ahe:Arch_1354 electron-tr
 

 
1.3945618e-5
 

 
1.0
 

 
C8YX89
 

 
0.218063
 

 
1.0
 

 
0.089810014
 

 
579
 

 
T
 

 
NIL
 

 

 
     Evidence for    RXN-10986 
  

 

 
 Hit 
 

 
 Common 
 

 
 P 
 

 
 #Q 
 

 
 Best Qry 
 

 
 Best Eval 
 

 
 Avg Rank 
 

 
 Aln Len 
 

 
 Qry Len 
 

 
 IDOP? 
 

 
 adj? 
 

 

 

 
GKFG-964-MONOMER
 

 
pak:HMPREF0675_3964 menB;
 

 
0.8538447
 

 
1.0
 

 
C8YX87
 

 
1.075e-21
 

 
1.0
 

 
1.0
 

 
253
 

 
T
 

 
NIL
 

 

 

 
GKFG-750-MONOMER
 

 
DNA polymerase III, delta
 

 
9.2874106e-4
 

 
1.0
 

 
C8YX87
 

 
0.135554
 

 
2.0
 

 
0.71146244
 

 
253
 

 
T
 

 
NIL
 

 

 
     Evidence for    RXN-10985 
  

 

 
 Hit 
 

 
 Common 
 

 
 P 
 

 
 #Q 
 

 
 Best Qry 
 

 
 Best Eval 
 

 
 Avg Rank 
 

 
 Aln Len 
 

 
 Qry Len 
 

 
 IDOP? 
 

 
 adj? 
 

 

 

 
GKFG-906-MONOMER
 

 
ahe:Arch_1362 L-carnitine
 

 
0.20464507
 

 
1.0
 

 
C8YX88
 

 
8.67532e-21
 

 
1.0
 

 
0.5116279
 

 
473
 

 
T
 

 
NIL
 

 

 
     

  stachyose degradation     Total # of reactions in pathway = 9   Present reactions: 4
  

 

 
 Reaction 
 

 
 Protein(s) 
 

 

 

 
UDPGLUCEPIM-RXN
 

 
(# )
 

 

 

 
RXN-11501
 

 
(# )
 

 

 

 
GLUC1PURIDYLTRANS-RXN
 

 
(# )
 

 

 

 
GALACTOKIN-RXN
 

 
(# )
 

 

 
   Missing reactions: 5
  

 

 
 UTPHEXPURIDYLYLTRANS-RXN 
 

 

 

 
 RXN-11502 
 

 

 

 
 GALACTURIDYLYLTRANS-RXN 
 

 

 

 
 RXN-11505 
 

 

 

 
 2.7.7.64-RXN 
 

 

 
        Evidence for UTP&mdash;hexose-1-phosphate uridylyltransferase, EC# 2.7.7.10   UTPHEXPURIDYLYLTRANS-RXN 
  

 

 
 Hit 
 

 
 Common 
 

 
 P 
 

 
 #Q 
 

 
 Best Qry 
 

 
 Best Eval 
 

 
 Avg Rank 
 

 
 Aln Len 
 

 
 Qry Len 
 

 
 IDOP? 
 

 
 adj? 
 

 

 

 
GKFG-833-MONOMER
 

 
mlu:Mlut_15870 hypothetic
 

 
0.0051016076
 

 
1.0
 

 
O33836
 

 
3.75269e-5
 

 
1.0
 

 
0.6383648
 

 
318
 

 
T
 

 
NIL
 

 

 

 
GKFG-1796-MONOMER
 

 
pac:PPA0489 UTP-glucose-1
 

 
0.0019139076
 

 
1.0
 

 
Q0GZS3
 

 
2.89989e-7
 

 
1.0
 

 
0.24918567
 

 
614
 

 
T
 

 
NIL
 

 

 

 
GKFG-1818-MONOMER
 

 
histidine triad (HIT) pro
 

 
5.771539e-5
 

 
1.0
 

 
O33836
 

 
5.02155e-4
 

 
2.0
 

 
0.3899371
 

 
318
 

 
T
 

 
NIL
 

 

 
     Evidence for , EC# 3.2.1.-   RXN-11502 
  

 

 
 Hit 
 

 
 Common 
 

 
 P 
 

 
 #Q 
 

 
 Best Qry 
 

 
 Best Eval 
 

 
 Avg Rank 
 

 
 Aln Len 
 

 
 Qry Len 
 

 
 IDOP? 
 

 
 adj? 
 

 

 

 
GKFG-275-MONOMER
 

 
hau:Haur_2392 beta-galact
 

 
1.3945618e-5
 

 
1.0
 

 
Q94A08
 

 
0.733189
 

 
1.0
 

 
0.112548515
 

 
773
 

 
T
 

 
NIL
 

 

 
     Evidence for UDP-glucose&mdash;hexose-1-phosphate uridylyltransferase, EC# 2.7.7.12   GALACTURIDYLYLTRANS-RXN 
  

 

 
 Hit 
 

 
 Common 
 

 
 P 
 

 
 #Q 
 

 
 Best Qry 
 

 
 Best Eval 
 

 
 Avg Rank 
 

 
 Aln Len 
 

 
 Qry Len 
 

 
 IDOP? 
 

 
 adj? 
 

 

 

 
GKFG-1789-MONOMER
 

 
mcu:HMPREF0573_11540 ileS
 

 
0.0051016076
 

 
1.0
 

 
P13212
 

 
0.00561481
 

 
1.0
 

 
0.6158192
 

 
354
 

 
T
 

 
NIL
 

 

 

 
GKFG-833-MONOMER
 

 
mlu:Mlut_15870 hypothetic
 

 
3.182795e-4
 

 
4.0
 

 
Q9FK51
 

 
6.35756e-6
 

 
1.0
 

 
0.4117871
 

 
351
 

 
T
 

 
NIL
 

 

 

 
GKFG-1818-MONOMER
 

 
histidine triad (HIT) pro
 

 
2.5307965e-5
 

 
2.0
 

 
O33836
 

 
5.02155e-4
 

 
1.5
 

 
0.25746855
 

 
318
 

 
T
 

 
NIL
 

 

 

 
GKFG-1189-MONOMER
 

 
car:cauri_0786 ABC transp
 

 
2.795795e-6
 

 
1.0
 

 
Q7RYE7
 

 
0.430187
 

 
2.0
 

 
0.20408164
 

 
392
 

 
T
 

 
NIL
 

 

 
     Evidence for    RXN-11505 
  

 

 
 Hit 
 

 
 Common 
 

 
 P 
 

 
 #Q 
 

 
 Best Qry 
 

 
 Best Eval 
 

 
 Avg Rank 
 

 
 Aln Len 
 

 
 Qry Len 
 

 
 IDOP? 
 

 
 adj? 
 

 

 

 
GKFG-1796-MONOMER
 

 
pac:PPA0489 UTP-glucose-1
 

 
0.07274204
 

 
1.0
 

 
Q0GZS3
 

 
2.89989e-7
 

 
1.0
 

 
0.24918567
 

 
614
 

 
T
 

 
T
 

 

 
     Evidence for UTP-monosaccharide-1-phosphate uridylyltransferase, EC# 2.7.7.64   2.7.7.64-RXN 
  

 

 
 Hit 
 

 
 Common 
 

 
 P 
 

 
 #Q 
 

 
 Best Qry 
 

 
 Best Eval 
 

 
 Avg Rank 
 

 
 Aln Len 
 

 
 Qry Len 
 

 
 IDOP? 
 

 
 adj? 
 

 

 

 
GKFG-1796-MONOMER
 

 
pac:PPA0489 UTP-glucose-1
 

 
0.0019139076
 

 
2.0
 

 
Q0GZS3
 

 
2.89989e-7
 

 
1.0
 

 
0.32507986
 

 
614
 

 
T
 

 
NIL
 

 

 

 
GKFG-1282-MONOMER
 

 
glucosamine/galactosamine
 

 
2.5282066e-6
 

 
1.0
 

 
Q5Z8Y4
 

 
0.386662
 

 
2.0
 

 
0.08603896
 

 
616
 

 
T
 

 
NIL
 

 

 
     

  chitin degradation II     Total # of reactions in pathway = 5   Present reactions: 2
  

 

 
 Reaction 
 

 
 Protein(s) 
 

 

 

 
RXN-12625
 

 
(# 
 # 
 # )
 

 

 

 
RXN-12626
 

 
(# 
 # 
 # )
 

 

 
   Missing reactions: 3
  

 

 
 3.2.1.14-RXN 
 

 

 

 
 RXN-12623 
 

 

 

 
 RXN-12624 
 

 

 
        Evidence for chitinase, EC# 3.2.1.14   3.2.1.14-RXN 
  

 

 
 Hit 
 

 
 Common 
 

 
 P 
 

 
 #Q 
 

 
 Best Qry 
 

 
 Best Eval 
 

 
 Avg Rank 
 

 
 Aln Len 
 

 
 Qry Len 
 

 
 IDOP? 
 

 
 adj? 
 

 

 

 
GKFG-300-MONOMER
 

 
UniRef90_F9EI64 ABC super
 

 
1.5421621e-5
 

 
1.0
 

 
P13656
 

 
0.0882349
 

 
1.0
 

 
0.18394649
 

 
897
 

 
T
 

 
NIL
 

 

 

 
GKFG-1057-MONOMER
 

 
bbi:BBIF_1733 sialidase; 
 

 
1.3945618e-5
 

 
1.0
 

 
tr|A3DC30|A3DC30_CLOTH
 

 
0.104393
 

 
1.0
 

 
0.09504132
 

 
484
 

 
T
 

 
NIL
 

 

 
     Evidence for  N,N' -diacetylchitobiose synthase, EC# 3.2.1.14   RXN-12623 
  

 

 
 Hit 
 

 
 Common 
 

 
 P 
 

 
 #Q 
 

 
 Best Qry 
 

 
 Best Eval 
 

 
 Avg Rank 
 

 
 Aln Len 
 

 
 Qry Len 
 

 
 IDOP? 
 

 
 adj? 
 

 

 

 
GKFG-170-MONOMER
 

 
ica:Intca_0690 NADH dehyd
 

 
1.3945618e-5
 

 
1.0
 

 
P96156
 

 
0.257049
 

 
1.0
 

 
0.06883365
 

 
1046
 

 
T
 

 
NIL
 

 

 
     Evidence for chitotriose synthase, EC# 3.2.1.14   RXN-12624 
  

 

 
 Hit 
 

 
 Common 
 

 
 P 
 

 
 #Q 
 

 
 Best Qry 
 

 
 Best Eval 
 

 
 Avg Rank 
 

 
 Aln Len 
 

 
 Qry Len 
 

 
 IDOP? 
 

 
 adj? 
 

 

 

 
GKFG-170-MONOMER
 

 
ica:Intca_0690 NADH dehyd
 

 
1.3945618e-5
 

 
1.0
 

 
P96156
 

 
0.257049
 

 
1.0
 

 
0.06883365
 

 
1046
 

 
T
 

 
NIL
 

 

 
     

  chitin derivatives degradation     Total # of reactions in pathway = 7   Present reactions: 3
  

 

 
 Reaction 
 

 
 Protein(s) 
 

 

 

 
NAG6PDEACET-RXN
 

 
(# 
 # )
 

 

 

 
GLUCOSAMINE-6-P-DEAMIN-RXN
 

 
(# )
 

 

 

 
N-ACETYLGLUCOSAMINE-KINASE-RXN
 

 
(# )
 

 

 
   Missing reactions: 4
  

 

 
 RXN-12627 
 

 

 

 
 PHOSACETYLGLUCOSAMINEMUT-RXN 
 

 

 

 
 RXN-12629 
 

 

 

 
 GLUCOSAMINE-KINASE-RXN 
 

 

 
        Evidence for  N,N' -diacetylchitobiose phosphorylase, EC# 2.4.1.-   RXN-12627 
  
no BLAST hits found
 
     Evidence for phosphoacetylglucosamine mutase, EC# 5.4.2.3   PHOSACETYLGLUCOSAMINEMUT-RXN 
  

 

 
 Hit 
 

 
 Common 
 

 
 P 
 

 
 #Q 
 

 
 Best Qry 
 

 
 Best Eval 
 

 
 Avg Rank 
 

 
 Aln Len 
 

 
 Qry Len 
 

 
 IDOP? 
 

 
 adj? 
 

 

 

 
GKFG-1741-MONOMER
 

 
ABC transporter ATP-bindi
 

 
1.5421621e-5
 

 
1.0
 

 
Q868I7
 

 
0.836232
 

 
1.0
 

 
0.15882353
 

 
510
 

 
T
 

 
NIL
 

 

 

 
GKFG-1280-MONOMER
 

 
bcv:Bcav_1788 dnaG; DNA p
 

 
1.3945618e-5
 

 
1.0
 

 
Q6ZDQ1
 

 
0.196888
 

 
1.0
 

 
0.09608541
 

 
562
 

 
T
 

 
NIL
 

 

 
     Evidence for chitobiase, EC# 3.2.1.-   RXN-12629      Evidence for glucosamine kinase, EC# 2.7.1.8   GLUCOSAMINE-KINASE-RXN 
  

 

 
 Hit 
 

 
 Common 
 

 
 P 
 

 
 #Q 
 

 
 Best Qry 
 

 
 Best Eval 
 

 
 Avg Rank 
 

 
 Aln Len 
 

 
 Qry Len 
 

 
 IDOP? 
 

 
 adj? 
 

 

 

 
GKFG-1733-MONOMER
 

 
pfr:PFREUD_22400 carbohyd
 

 
1.3945618e-5
 

 
1.0
 

 
Q9KUA9
 

 
0.396548
 

 
1.0
 

 
0.10204082
 

 
294
 

 
T
 

 
NIL
 

 

 
     

  glycogen degradation II     Total # of reactions in pathway = 5   Present reactions: 4
  

 

 
 Reaction 
 

 
 Protein(s) 
 

 

 

 
GLYCOPHOSPHORYL-RXN
 

 
(# )
 

 

 

 
RXN-9023
 

 
(# )
 

 

 

 
RXN-9025
 

 
(# )
 

 

 

 
PHOSPHOGLUCMUT-RXN
 

 
(# )
 

 

 
   Missing reactions: 1
  

 

 
 3.2.1.33-RXN 
 

 

 
        Evidence for amylo-&alpha;-1,6-glucosidase, EC# 3.2.1.33   3.2.1.33-RXN 
  

 

 
 Hit 
 

 
 Common 
 

 
 P 
 

 
 #Q 
 

 
 Best Qry 
 

 
 Best Eval 
 

 
 Avg Rank 
 

 
 Aln Len 
 

 
 Qry Len 
 

 
 IDOP? 
 

 
 adj? 
 

 

 

 
GKFG-1241-MONOMER
 

 
ahe:Arch_0385 trehalose s
 

 
1.3945618e-5
 

 
2.0
 

 
Q06625
 

 
0.079835
 

 
1.0
 

 
0.04581031
 

 
1536
 

 
T
 

 
NIL
 

 

 

 
GKFG-1759-MONOMER
 

 
tnp3521; IS3521 transposa
 

 
2.5282066e-6
 

 
1.0
 

 
Q06625
 

 
0.228978
 

 
2.0
 

 
0.045572918
 

 
1536
 

 
T
 

 
NIL
 

 

 

 
GKFG-1217-MONOMER
 

 
ske:Sked_04300 LacI famil
 

 
1.0926308e-6
 

 
1.0
 

 
Q06625
 

 
0.349111
 

 
3.0
 

 
0.046223957
 

 
1536
 

 
T
 

 
NIL
 

 

 
     

  chondroitin sulfate degradation (metazoa)     Total # of reactions in pathway = 4   Present reactions: 1
  

 

 
 Reaction 
 

 
 Protein(s) 
 

 

 

 
RXN-11622
 

 
(# 
 # 
 # )
 

 

 
   Missing reactions: 3
  

 

 
 3.1.6.12-RXN 
 

 

 

 
 3.1.6.4-RXN 
 

 

 

 
 RXN-11620 
 

 

 
        Evidence for  N -acetylgalactosamine-4-sulfatase, EC# 3.1.6.12   3.1.6.12-RXN 
  

 

 
 Hit 
 

 
 Common 
 

 
 P 
 

 
 #Q 
 

 
 Best Qry 
 

 
 Best Eval 
 

 
 Avg Rank 
 

 
 Aln Len 
 

 
 Qry Len 
 

 
 IDOP? 
 

 
 adj? 
 

 

 

 
GKFG-1529-MONOMER
 

 
sco:SCO7547 SC5F1.01, SC8
 

 
0.035064183
 

 
2.0
 

 
P50429
 

 
6.2601e-11
 

 
1.0
 

 
0.43707693
 

 
534
 

 
T
 

 
NIL
 

 

 
     Evidence for  N -acetylgalactosamine-6-sulfatase, EC# 3.1.6.4   3.1.6.4-RXN 
  

 

 
 Hit 
 

 
 Common 
 

 
 P 
 

 
 #Q 
 

 
 Best Qry 
 

 
 Best Eval 
 

 
 Avg Rank 
 

 
 Aln Len 
 

 
 Qry Len 
 

 
 IDOP? 
 

 
 adj? 
 

 

 

 
GKFG-1529-MONOMER
 

 
sco:SCO7547 SC5F1.01, SC8
 

 
0.015683802
 

 
2.0
 

 
Q32KH5
 

 
5.6646e-13
 

 
1.0
 

 
0.25812715
 

 
522
 

 
T
 

 
NIL
 

 

 

 
GKFG-1227-MONOMER
 

 
hypothetical protein (db=
 

 
2.795795e-6
 

 
1.0
 

 
Q32KH5
 

 
0.362697
 

 
2.0
 

 
0.20306513
 

 
522
 

 
T
 

 
NIL
 

 

 

 
GKFG-1857-MONOMER
 

 
aai:AARI_29350 hsdM; type
 

 
2.5282066e-6
 

 
1.0
 

 
Q32KJ6
 

 
0.207991
 

 
2.0
 

 
0.04389313
 

 
524
 

 
T
 

 
NIL
 

 

 
     Evidence for , EC# 3.2.1.35   RXN-11620 
  

 

 
 Hit 
 

 
 Common 
 

 
 P 
 

 
 #Q 
 

 
 Best Qry 
 

 
 Best Eval 
 

 
 Avg Rank 
 

 
 Aln Len 
 

 
 Qry Len 
 

 
 IDOP? 
 

 
 adj? 
 

 

 

 
GKFG-852-MONOMER
 

 
putative phage capsid pro
 

 
1.5421621e-5
 

 
1.0
 

 
Q12794
 

 
0.116592
 

 
1.0
 

 
0.13103448
 

 
435
 

 
T
 

 
NIL
 

 

 
     

  dermatan sulfate degradation (metazoa)     Total # of reactions in pathway = 4   Present reactions: 1
  

 

 
 Reaction 
 

 
 Protein(s) 
 

 

 

 
RXN-11622
 

 
(# 
 # 
 # )
 

 

 
   Missing reactions: 3
  

 

 
 RXN-11569 
 

 

 

 
 3.2.1.76-RXN 
 

 

 

 
 RXN-11618 
 

 

 
        Evidence for iduronate-2-sulfatase, EC# 3.1.6.13   RXN-11569 
  

 

 
 Hit 
 

 
 Common 
 

 
 P 
 

 
 #Q 
 

 
 Best Qry 
 

 
 Best Eval 
 

 
 Avg Rank 
 

 
 Aln Len 
 

 
 Qry Len 
 

 
 IDOP? 
 

 
 adj? 
 

 

 

 
GKFG-1529-MONOMER
 

 
sco:SCO7547 SC5F1.01, SC8
 

 
0.5596166
 

 
2.0
 

 
P22304
 

 
9.30642e-24
 

 
1.0
 

 
0.7468083
 

 
550
 

 
T
 

 
NIL
 

 

 
     Evidence for  L -iduronidase, EC# 3.2.1.76   3.2.1.76-RXN 
  

 

 
 Hit 
 

 
 Common 
 

 
 P 
 

 
 #Q 
 

 
 Best Qry 
 

 
 Best Eval 
 

 
 Avg Rank 
 

 
 Aln Len 
 

 
 Qry Len 
 

 
 IDOP? 
 

 
 adj? 
 

 

 

 
GKFG-964-MONOMER
 

 
pak:HMPREF0675_3964 menB;
 

 
1.3945618e-5
 

 
1.0
 

 
P48441
 

 
0.684562
 

 
1.0
 

 
0.083596215
 

 
634
 

 
T
 

 
NIL
 

 

 
     Evidence for hyaluronoglucosaminidase, EC# 3.2.1.35   RXN-11618 
  

 

 
 Hit 
 

 
 Common 
 

 
 P 
 

 
 #Q 
 

 
 Best Qry 
 

 
 Best Eval 
 

 
 Avg Rank 
 

 
 Aln Len 
 

 
 Qry Len 
 

 
 IDOP? 
 

 
 adj? 
 

 

 

 
GKFG-852-MONOMER
 

 
putative phage capsid pro
 

 
1.5421621e-5
 

 
1.0
 

 
Q12794
 

 
0.116592
 

 
1.0
 

 
0.13103448
 

 
435
 

 
T
 

 
NIL
 

 

 
     

  starch degradation V     Total # of reactions in pathway = 4   Present reactions: 3
  

 

 
 Reaction 
 

 
 Protein(s) 
 

 

 

 
RXN-12171
 

 
(# )
 

 

 

 
PHOSPHOGLUCMUT-RXN
 

 
(# )
 

 

 

 
RXN-12193
 

 
(# )
 

 

 
   Missing reactions: 1
  

 

 
 RXN-12190 
 

 

 
        Evidence for &alpha;-amylase, EC# 3.2.1.-   RXN-12190 
  
no BLAST hits found
 
     

  xyloglucan degradation II (exoglucanase)     Total # of reactions in pathway = 6   Present reactions: 3
  

 

 
 Reaction 
 

 
 Protein(s) 
 

 

 

 
RXN-12398
 

 
(# 
 # )
 

 

 

 
RXN-12399
 

 
(# 
 # )
 

 

 

 
RXN-12400
 

 
(# 
 # )
 

 

 
   Missing reactions: 3
  

 

 
 3.2.1.120-RXN 
 

 

 

 
 RXN-12397 
 

 

 

 
 RXN-12402 
 

 

 
        Evidence for oligoxyloglucan &beta;-glycosidase, EC# 3.2.1.120   3.2.1.120-RXN      Evidence for , EC# 3.2.1.155   RXN-12397 
  
no BLAST hits found
 
     Evidence for isoprimeverose &alpha;-xylosidase, EC# 3.2.1.177   RXN-12402 
  
no BLAST hits found
 
     

  xyloglucan degradation I (endoglucanase)     Total # of reactions in pathway = 6   Present reactions: 3
  

 

 
 Reaction 
 

 
 Protein(s) 
 

 

 

 
RXN-12398
 

 
(# 
 # )
 

 

 

 
RXN-12399
 

 
(# 
 # )
 

 

 

 
RXN-12400
 

 
(# 
 # )
 

 

 
   Missing reactions: 3
  

 

 
 3.2.1.120-RXN 
 

 

 

 
 RXN-12401 
 

 

 

 
 RXN-12402 
 

 

 
        Evidence for oligoxyloglucan &beta;-glycosidase, EC# 3.2.1.120   3.2.1.120-RXN      Evidence for xyloglucan-specific endo-&beta;-1,4-glucanase, EC# 3.2.1.151   RXN-12401 
  
no BLAST hits found
 
     Evidence for isoprimeverose &alpha;-xylosidase, EC# 3.2.1.177   RXN-12402 
  
no BLAST hits found
 
     

  galactose degradation IV     Total # of reactions in pathway = 8   Present reactions: 1
  

 

 
 Reaction 
 

 
 Protein(s) 
 

 

 

 
RXN-11503
 

 
(# )
 

 

 
   Missing reactions: 7
  

 

 
 RXN-12078 
 

 

 

 
 RXN-12079 
 

 

 

 
 RXN-12080 
 

 

 

 
 RXN-12081 
 

 

 

 
 1.1.1.289-RXN 
 

 

 

 
 RXN-12082 
 

 

 

 
 RXN-12083 
 

 

 
        Evidence for &beta;-D-galactose reductase, EC# 1.1.1.21   RXN-12078 
  

 

 
 Hit 
 

 
 Common 
 

 
 P 
 

 
 #Q 
 

 
 Best Qry 
 

 
 Best Eval 
 

 
 Avg Rank 
 

 
 Aln Len 
 

 
 Qry Len 
 

 
 IDOP? 
 

 
 adj? 
 

 

 

 
GKFG-1089-MONOMER
 

 
147998..148516 - ( gc_con
 

 
3.182795e-4
 

 
1.0
 

 
Q0PGJ6
 

 
0.952555
 

 
1.0
 

 
0.43174604
 

 
315
 

 
T
 

 
NIL
 

 

 
     Evidence for D-galactitol dehydrogenase, EC# 1.1.1.-   RXN-12079 
  

 

 
 Hit 
 

 
 Common 
 

 
 P 
 

 
 #Q 
 

 
 Best Qry 
 

 
 Best Eval 
 

 
 Avg Rank 
 

 
 Aln Len 
 

 
 Qry Len 
 

 
 IDOP? 
 

 
 adj? 
 

 

 

 
GKFG-1882-MONOMER
 

 
pcn:TIB1ST10_03215 oxidor
 

 
0.8538447
 

 
1.0
 

 
Q96V44
 

 
2.25221e-24
 

 
1.0
 

 
0.8832891
 

 
377
 

 
T
 

 
NIL
 

 

 

 
GKFG-935-MONOMER
 

 
ahe:Arch_0038 alcohol deh
 

 
0.26960617
 

 
1.0
 

 
Q96V44
 

 
1.05236e-19
 

 
3.0
 

 
0.94164455
 

 
377
 

 
T
 

 
NIL
 

 

 

 
GKFG-860-MONOMER
 

 
msm:MSMEG_3094 oxidoreduc
 

 
0.15667598
 

 
1.0
 

 
Q96V44
 

 
5.98086e-20
 

 
2.0
 

 
0.8328912
 

 
377
 

 
T
 

 
NIL
 

 

 

 
GKFG-682-MONOMER
 

 
mcu:HMPREF0573_10178 amid
 

 
4.3925725e-6
 

 
1.0
 

 
Q96V44
 

 
0.172995
 

 
5.0
 

 
0.14854111
 

 
377
 

 
T
 

 
NIL
 

 

 

 
GKFG-1960-MONOMER
 

 
mcu:HMPREF0573_10477 adhC
 

 
1.6156636e-6
 

 
1.0
 

 
Q96V44
 

 
3.59925e-4
 

 
4.0
 

 
0.23076923
 

 
377
 

 
T
 

 
NIL
 

 

 
     Evidence for L-xylo-3-hexulose reductase   RXN-12080      Evidence for galactitol dehydrogenase   RXN-12081      Evidence for sorbose reductase, EC# 1.1.1.289   1.1.1.289-RXN 
  

 

 
 Hit 
 

 
 Common 
 

 
 P 
 

 
 #Q 
 

 
 Best Qry 
 

 
 Best Eval 
 

 
 Avg Rank 
 

 
 Aln Len 
 

 
 Qry Len 
 

 
 IDOP? 
 

 
 adj? 
 

 

 

 
GKFG-720-MONOMER
 

 
fabG; 3-ketoacyl-ACP redu
 

 
0.8538447
 

 
2.0
 

 
P87219
 

 
3.3903e-29
 

 
1.0
 

 
0.97777545
 

 
281
 

 
T
 

 
NIL
 

 

 

 
GKFG-803-MONOMER
 

 
short-chain dehydrogenase
 

 
0.0016251113
 

 
1.0
 

 
Q9Y6Z9
 

 
6.1688e-6
 

 
2.0
 

 
0.73333335
 

 
255
 

 
T
 

 
NIL
 

 

 
     Evidence for L-xylo-3-hexulose reductase   RXN-12082      Evidence for D-sorbitol dehydrogenase, EC# 1.1.1.-   RXN-12083 
  

 

 
 Hit 
 

 
 Common 
 

 
 P 
 

 
 #Q 
 

 
 Best Qry 
 

 
 Best Eval 
 

 
 Avg Rank 
 

 
 Aln Len 
 

 
 Qry Len 
 

 
 IDOP? 
 

 
 adj? 
 

 

 

 
GKFG-860-MONOMER
 

 
msm:MSMEG_3094 oxidoreduc
 

 
0.95801926
 

 
1.0
 

 
Q876R2
 

 
4.4843e-37
 

 
1.0
 

 
0.9338843
 

 
363
 

 
T
 

 
NIL
 

 

 

 
GKFG-1882-MONOMER
 

 
pcn:TIB1ST10_03215 oxidor
 

 
0.5143494
 

 
1.0
 

 
Q876R2
 

 
1.03035e-25
 

 
2.0
 

 
1.0
 

 
363
 

 
T
 

 
NIL
 

 

 

 
GKFG-935-MONOMER
 

 
ahe:Arch_0038 alcohol deh
 

 
0.31399453
 

 
1.0
 

 
Q876R2
 

 
1.67376e-21
 

 
3.0
 

 
0.94214875
 

 
363
 

 
T
 

 
NIL
 

 

 

 
GKFG-1960-MONOMER
 

 
mcu:HMPREF0573_10477 adhC
 

 
0.021276189
 

 
1.0
 

 
Q876R2
 

 
2.55409e-13
 

 
4.0
 

 
0.58126724
 

 
363
 

 
T
 

 
NIL
 

 

 

 
GKFG-1862-MONOMER
 

 
ahe:Arch_1499 hydroxymeth
 

 
9.067587e-5
 

 
1.0
 

 
Q876R2
 

 
5.07512e-4
 

 
5.0
 

 
0.3966942
 

 
363
 

 
T
 

 
NIL
 

 

 
     

  galactose degradation I (Leloir pathway)     Total # of reactions in pathway = 5   Present reactions: 3
  

 

 
 Reaction 
 

 
 Protein(s) 
 

 

 

 
GALACTOKIN-RXN
 

 
(# )
 

 

 

 
UDPGLUCEPIM-RXN
 

 
(# )
 

 

 

 
ALDOSE1EPIM-RXN
 

 
(# )
 

 

 
   Missing reactions: 2
  

 

 
 GALACTURIDYLYLTRANS-RXN 
 

 

 

 
 BETA-PHOSPHOGLUCOMUTASE-RXN 
 

 

 
        Evidence for UDP-glucose&mdash;hexose-1-phosphate uridylyltransferase, EC# 2.7.7.12   GALACTURIDYLYLTRANS-RXN 
  

 

 
 Hit 
 

 
 Common 
 

 
 P 
 

 
 #Q 
 

 
 Best Qry 
 

 
 Best Eval 
 

 
 Avg Rank 
 

 
 Aln Len 
 

 
 Qry Len 
 

 
 IDOP? 
 

 
 adj? 
 

 

 

 
GKFG-1789-MONOMER
 

 
mcu:HMPREF0573_11540 ileS
 

 
0.0051016076
 

 
1.0
 

 
P13212
 

 
0.00561481
 

 
1.0
 

 
0.6158192
 

 
354
 

 
T
 

 
NIL
 

 

 

 
GKFG-833-MONOMER
 

 
mlu:Mlut_15870 hypothetic
 

 
3.182795e-4
 

 
4.0
 

 
Q9FK51
 

 
6.35756e-6
 

 
1.0
 

 
0.4117871
 

 
351
 

 
T
 

 
NIL
 

 

 

 
GKFG-1818-MONOMER
 

 
histidine triad (HIT) pro
 

 
2.5307965e-5
 

 
2.0
 

 
O33836
 

 
5.02155e-4
 

 
1.5
 

 
0.25746855
 

 
318
 

 
T
 

 
NIL
 

 

 

 
GKFG-1189-MONOMER
 

 
car:cauri_0786 ABC transp
 

 
2.795795e-6
 

 
1.0
 

 
Q7RYE7
 

 
0.430187
 

 
2.0
 

 
0.20408164
 

 
392
 

 
T
 

 
NIL
 

 

 
     Evidence for &beta;-phosphoglucomutase, EC# 5.4.2.6   BETA-PHOSPHOGLUCOMUTASE-RXN 
  

 

 
 Hit 
 

 
 Common 
 

 
 P 
 

 
 #Q 
 

 
 Best Qry 
 

 
 Best Eval 
 

 
 Avg Rank 
 

 
 Aln Len 
 

 
 Qry Len 
 

 
 IDOP? 
 

 
 adj? 
 

 

 

 
GKFG-1963-MONOMER
 

 
HAD-superfamily hydrolase
 

 
0.007427732
 

 
3.0
 

 
P77366
 

 
9.82058e-7
 

 
1.3333334
 

 
0.84374255
 

 
219
 

 
T
 

 
NIL
 

 

 

 
GKFG-1968-MONOMER
 

 
5'-nucleotidase (EC:3.1.3
 

 
1.4241917e-4
 

 
2.0
 

 
P71447
 

 
4.47743e-4
 

 
3.0
 

 
0.5871041
 

 
221
 

 
T
 

 
NIL
 

 

 

 
GKFG-1581-MONOMER
 

 
phosphatase/phosphohexomu
 

 
5.771539e-5
 

 
3.0
 

 
P77366
 

 
1.71479e-4
 

 
2.0
 

 
0.4328808
 

 
219
 

 
T
 

 
NIL
 

 

 

 
GKFG-1734-MONOMER
 

 
pad:TIIST44_04425 HAD hyd
 

 
1.2082764e-6
 

 
1.0
 

 
O06995
 

 
0.0669041
 

 
3.0
 

 
0.19026549
 

 
226
 

 
T
 

 
NIL
 

 

 
     

  L-arabinose degradation I     Total # of reactions in pathway = 3   Present reactions: 2
  

 

 
 Reaction 
 

 
 Protein(s) 
 

 

 

 
RIBULPEPIM-RXN
 

 
(# )
 

 

 

 
RXN0-5116
 

 
(# )
 

 

 
   Missing reactions: 1
  

 

 
 ARABISOM-RXN 
 

 

 
        Evidence for  L -arabinose isomerase, EC# 5.3.1.4   ARABISOM-RXN 
  

 

 
 Hit 
 

 
 Common 
 

 
 P 
 

 
 #Q 
 

 
 Best Qry 
 

 
 Best Eval 
 

 
 Avg Rank 
 

 
 Aln Len 
 

 
 Qry Len 
 

 
 IDOP? 
 

 
 adj? 
 

 

 

 
GKFG-182-MONOMER
 

 
menA; 1,4-dihydroxy-2-nap
 

 
3.787886e-5
 

 
1.0
 

 
B1ZRZ1
 

 
0.935074
 

 
1.0
 

 
0.06944445
 

 
504
 

 
NIL
 

 
NIL
 

 

 

 
GKFG-1209-MONOMER
 

 
hypothetical protein (db=
 

 
1.5421621e-5
 

 
1.0
 

 
A0JRF5
 

 
0.319532
 

 
1.0
 

 
0.12915851
 

 
511
 

 
T
 

 
NIL
 

 

 

 
GKFG-905-MONOMER
 

 
pfr:PFREUD_02440 caiC; cr
 

 
1.3945618e-5
 

 
1.0
 

 
A8GE04
 

 
0.315744
 

 
1.0
 

 
0.07984032
 

 
501
 

 
T
 

 
NIL
 

 

 

 
GKFG-1002-MONOMER
 

 
AcrR family transcription
 

 
1.3945618e-5
 

 
1.0
 

 
Q97JE4
 

 
0.373483
 

 
1.0
 

 
0.11680328
 

 
488
 

 
T
 

 
NIL
 

 

 
     

  sucrose degradation V (mammalian)     Total # of reactions in pathway = 5   Present reactions: 2
  

 

 
 Reaction 
 

 
 Protein(s) 
 

 

 

 
RXN-8631
 

 
(# )
 

 

 

 
TRIOSEPISOMERIZATION-RXN
 

 
(# )
 

 

 
   Missing reactions: 3
  

 

 
 3.2.1.48-RXN 
 

 

 

 
 KETOHEXOKINASE-RXN 
 

 

 

 
 TRIOKINASE-RXN 
 

 

 
        Evidence for sucrose &alpha;-glucosidase, EC# 3.2.1.48   3.2.1.48-RXN 
  

 

 
 Hit 
 

 
 Common 
 

 
 P 
 

 
 #Q 
 

 
 Best Qry 
 

 
 Best Eval 
 

 
 Avg Rank 
 

 
 Aln Len 
 

 
 Qry Len 
 

 
 IDOP? 
 

 
 adj? 
 

 

 

 
GKFG-1518-MONOMER
 

 
surA; cell surface protei
 

 
0.039642163
 

 
1.0
 

 
P56729
 

 
0.636084
 

 
1.0
 

 
0.8548387
 

 
62
 

 
T
 

 
NIL
 

 

 

 
GKFG-670-MONOMER
 

 
peroxiredoxin; K03386 per
 

 
1.3945618e-5
 

 
1.0
 

 
P23739
 

 
0.0053332
 

 
1.0
 

 
0.046713743
 

 
1841
 

 
T
 

 
NIL
 

 

 
     Evidence for ketohexokinase, EC# 2.7.1.3   KETOHEXOKINASE-RXN 
  

 

 
 Hit 
 

 
 Common 
 

 
 P 
 

 
 #Q 
 

 
 Best Qry 
 

 
 Best Eval 
 

 
 Avg Rank 
 

 
 Aln Len 
 

 
 Qry Len 
 

 
 IDOP? 
 

 
 adj? 
 

 

 

 
GKFG-887-MONOMER
 

 
UniRef90_C2KTG6 Possible 
 

 
0.008898924
 

 
2.0
 

 
P50053
 

 
1.55697e-5
 

 
1.0
 

 
0.8271812
 

 
298
 

 
T
 

 
NIL
 

 

 

 
GKFG-1332-MONOMER
 

 
SCC77.07c; carbohydrate k
 

 
9.2874106e-4
 

 
2.0
 

 
Q02974
 

 
0.0171643
 

 
2.0
 

 
0.6392617
 

 
298
 

 
T
 

 
NIL
 

 

 

 
GKFG-681-MONOMER
 

 
archaeal fructose-1,6-bis
 

 
1.2082764e-6
 

 
2.0
 

 
Q02974
 

 
0.318987
 

 
3.0
 

 
0.16442953
 

 
298
 

 
T
 

 
NIL
 

 

 
     Evidence for triokinase, EC# 2.7.1.28   TRIOKINASE-RXN 
  

 

 
 Hit 
 

 
 Common 
 

 
 P 
 

 
 #Q 
 

 
 Best Qry 
 

 
 Best Eval 
 

 
 Avg Rank 
 

 
 Aln Len 
 

 
 Qry Len 
 

 
 IDOP? 
 

 
 adj? 
 

 

 

 
GKFG-284-MONOMER
 

 
pad:TIIST44_02530 dihydro
 

 
0.73190683
 

 
1.0
 

 
Q9HFC5
 

 
1.37386e-38
 

 
1.0
 

 
0.57240206
 

 
587
 

 
NIL
 

 
NIL
 

 

 

 
GKFG-283-MONOMER
 

 
dihydroxyacetone kinase, 
 

 
0.007785062
 

 
1.0
 

 
Q9HFC5
 

 
4.38481e-12
 

 
2.0
 

 
0.3339012
 

 
587
 

 
NIL
 

 
NIL
 

 

 

 
GKFG-1489-MONOMER
 

 
lacto-N-biosidase (EC:3.2
 

 
1.0926308e-6
 

 
1.0
 

 
Q9HFC5
 

 
0.799892
 

 
3.0
 

 
0.08688245
 

 
587
 

 
T
 

 
NIL
 

 

 
     

  fatty acid &beta;-oxidation I     Total # of reactions in pathway = 7   Present reactions: 4
  

 

 
 Reaction 
 

 
 Protein(s) 
 

 

 

 
OHACYL-COA-DEHYDROG-RXN
 

 
(# )
 

 

 

 
KETOACYLCOATHIOL-RXN
 

 
(# )
 

 

 

 
ACYLCOASYN-RXN
 

 
(# 
 # 
 # 
 # )
 

 

 

 
ACYLCOADEHYDROG-RXN
 

 
NIL
 

 

 
   Missing reactions: 3
  

 

 
 OHBUTYRYL-COA-EPIM-RXN 
 

 

 

 
 ENOYL-COA-DELTA-ISOM-RXN 
 

 

 

 
 ENOYL-COA-HYDRAT-RXN 
 

 

 
        Evidence for 3-hydroxybutyryl-CoA epimerase, EC# 5.1.2.3   OHBUTYRYL-COA-EPIM-RXN 
  

 

 
 Hit 
 

 
 Common 
 

 
 P 
 

 
 #Q 
 

 
 Best Qry 
 

 
 Best Eval 
 

 
 Avg Rank 
 

 
 Aln Len 
 

 
 Qry Len 
 

 
 IDOP? 
 

 
 adj? 
 

 

 

 
GKFG-964-MONOMER
 

 
pak:HMPREF0675_3964 menB;
 

 
1.5421621e-5
 

 
2.0
 

 
P77399
 

 
1.42041e-6
 

 
1.0
 

 
0.21160275
 

 
714
 

 
T
 

 
NIL
 

 

 

 
GKFG-653-MONOMER
 

 
bcv:Bcav_2498 ispG; 4-hyd
 

 
2.5282066e-6
 

 
1.0
 

 
P77399
 

 
0.0185922
 

 
2.0
 

 
0.072829135
 

 
714
 

 
T
 

 
NIL
 

 

 

 
GKFG-1246-MONOMER
 

 
asm:MOUSESFB_1050 type I 
 

 
1.2082764e-6
 

 
1.0
 

 
P77399
 

 
0.0335953
 

 
3.0
 

 
0.13025211
 

 
714
 

 
T
 

 
NIL
 

 

 
     Evidence for Dodecenoyl-CoA &delta;-isomerase, EC# 5.3.3.8   ENOYL-COA-DELTA-ISOM-RXN 
  

 

 
 Hit 
 

 
 Common 
 

 
 P 
 

 
 #Q 
 

 
 Best Qry 
 

 
 Best Eval 
 

 
 Avg Rank 
 

 
 Aln Len 
 

 
 Qry Len 
 

 
 IDOP? 
 

 
 adj? 
 

 

 

 
GKFG-964-MONOMER
 

 
pak:HMPREF0675_3964 menB;
 

 
1.5421621e-5
 

 
2.0
 

 
P77399
 

 
1.42041e-6
 

 
1.0
 

 
0.21160275
 

 
714
 

 
T
 

 
NIL
 

 

 

 
GKFG-653-MONOMER
 

 
bcv:Bcav_2498 ispG; 4-hyd
 

 
2.5282066e-6
 

 
1.0
 

 
P77399
 

 
0.0185922
 

 
2.0
 

 
0.072829135
 

 
714
 

 
T
 

 
NIL
 

 

 

 
GKFG-1246-MONOMER
 

 
asm:MOUSESFB_1050 type I 
 

 
1.2082764e-6
 

 
1.0
 

 
P77399
 

 
0.0335953
 

 
3.0
 

 
0.13025211
 

 
714
 

 
T
 

 
NIL
 

 

 
     Evidence for enoyl-CoA hydratase, EC# 4.2.1.17   ENOYL-COA-HYDRAT-RXN 
  

 

 
 Hit 
 

 
 Common 
 

 
 P 
 

 
 #Q 
 

 
 Best Qry 
 

 
 Best Eval 
 

 
 Avg Rank 
 

 
 Aln Len 
 

 
 Qry Len 
 

 
 IDOP? 
 

 
 adj? 
 

 

 

 
GKFG-964-MONOMER
 

 
pak:HMPREF0675_3964 menB;
 

 
0.8856589
 

 
20.0
 

 
P77467
 

 
3.58023e-24
 

 
1.0
 

 
0.53590816
 

 
262
 

 
T
 

 
NIL
 

 

 

 
GKFG-1235-MONOMER
 

 
msm:MSMEG_0582 succinate-
 

 
0.035064183
 

 
1.0
 

 
P77455
 

 
3.97539e-14
 

 
1.0
 

 
0.41556534
 

 
681
 

 
T
 

 
NIL
 

 

 

 
GKFG-997-MONOMER
 

 
ssm:Spirs_0789 aldehyde d
 

 
1.4241917e-4
 

 
1.0
 

 
P77455
 

 
7.06938e-8
 

 
3.0
 

 
0.56828195
 

 
681
 

 
T
 

 
NIL
 

 

 

 
GKFG-328-MONOMER
 

 
mcu:HMPREF0573_11678 L-pr
 

 
9.0592475e-5
 

 
1.0
 

 
P77455
 

 
2.09464e-4
 

 
4.0
 

 
0.42143905
 

 
681
 

 
NIL
 

 
NIL
 

 

 

 
GKFG-977-MONOMER
 

 
paz:TIA2EST2_02200 methyl
 

 
5.771539e-5
 

 
1.0
 

 
P77455
 

 
6.07981e-10
 

 
2.0
 

 
0.46108663
 

 
681
 

 
T
 

 
NIL
 

 

 

 
GKFG-1815-MONOMER
 

 
lipid A biosynthesis acyl
 

 
2.5307965e-5
 

 
1.0
 

 
Q73VC7
 

 
0.676487
 

 
2.0
 

 
0.26070037
 

 
257
 

 
T
 

 
NIL
 

 

 

 
GKFG-1304-MONOMER
 

 
UniRef90_UPI000051013A hy
 

 
1.3945618e-5
 

 
1.0
 

 
Q9ZCZ1
 

 
0.295484
 

 
1.0
 

 
0.06666667
 

 
720
 

 
T
 

 
NIL
 

 

 

 
GKFG-83-MONOMER
 

 
transmembrane_regions (db
 

 
7.594039e-6
 

 
1.0
 

 
Q6LTK3
 

 
0.745272
 

 
2.0
 

 
0.13706294
 

 
715
 

 
NIL
 

 
NIL
 

 

 

 
GKFG-1943-MONOMER
 

 
bcv:Bcav_1945 P-type HAD 
 

 
2.795795e-6
 

 
1.0
 

 
Q07ZP8
 

 
0.605252
 

 
2.0
 

 
0.122535214
 

 
710
 

 
T
 

 
NIL
 

 

 

 
GKFG-739-MONOMER
 

 
bcv:Bcav_1600 DEAD/DEAH b
 

 
2.795795e-6
 

 
1.0
 

 
P76082
 

 
0.180348
 

 
2.0
 

 
0.23137255
 

 
255
 

 
T
 

 
NIL
 

 

 

 
GKFG-653-MONOMER
 

 
bcv:Bcav_2498 ispG; 4-hyd
 

 
2.5282066e-6
 

 
2.0
 

 
P77399
 

 
0.0185922
 

 
2.0
 

 
0.070006296
 

 
714
 

 
T
 

 
NIL
 

 

 

 
GKFG-1746-MONOMER
 

 
two-component system sens
 

 
2.5282066e-6
 

 
1.0
 

 
A6TGM4
 

 
0.422863
 

 
2.0
 

 
0.061728396
 

 
729
 

 
T
 

 
NIL
 

 

 

 
GKFG-900-MONOMER
 

 
ahe:Arch_1354 electron-tr
 

 
2.5282066e-6
 

 
1.0
 

 
Q9ZCZ1
 

 
0.832152
 

 
2.0
 

 
0.03888889
 

 
720
 

 
T
 

 
NIL
 

 

 

 
GKFG-937-MONOMER
 

 
ahe:Arch_0039 sugar trans
 

 
1.2082764e-6
 

 
1.0
 

 
A4TM82
 

 
0.542098
 

 
3.0
 

 
0.125323
 

 
774
 

 
T
 

 
NIL
 

 

 

 
GKFG-1246-MONOMER
 

 
asm:MOUSESFB_1050 type I 
 

 
1.2082764e-6
 

 
1.0
 

 
P77399
 

 
0.0335953
 

 
3.0
 

 
0.13025211
 

 
714
 

 
T
 

 
NIL
 

 

 
     

  purine deoxyribonucleosides degradation     Total # of reactions in pathway = 7   Present reactions: 2
  

 

 
 Reaction 
 

 
 Protein(s) 
 

 

 

 
DEOXYRIBOSE-P-ALD-RXN
 

 
(# )
 

 

 

 
ACETALD-DEHYDROG-RXN
 

 
(# )
 

 

 
   Missing reactions: 5
  

 

 
 DEOXYGUANPHOSPHOR-RXN 
 

 

 

 
 DEOXYADENPHOSPHOR-RXN 
 

 

 

 
 DEOXYINOPHOSPHOR-RXN 
 

 

 

 
 ADDALT-RXN 
 

 

 

 
 D-PPENTOMUT-RXN 
 

 

 
        Evidence for , EC# 2.4.2.1   DEOXYGUANPHOSPHOR-RXN 
  

 

 
 Hit 
 

 
 Common 
 

 
 P 
 

 
 #Q 
 

 
 Best Qry 
 

 
 Best Eval 
 

 
 Avg Rank 
 

 
 Aln Len 
 

 
 Qry Len 
 

 
 IDOP? 
 

 
 adj? 
 

 

 

 
GKFG-1361-MONOMER
 

 
hypothetical protein; K06
 

 
1.5421621e-5
 

 
1.0
 

 
P00491
 

 
0.0990956
 

 
1.0
 

 
0.18685122
 

 
289
 

 
T
 

 
NIL
 

 

 
     Evidence for , EC# 2.4.2.1   DEOXYADENPHOSPHOR-RXN 
  
no BLAST hits found
 
     Evidence for , EC# 2.4.2.1   DEOXYINOPHOSPHOR-RXN 
  

 

 
 Hit 
 

 
 Common 
 

 
 P 
 

 
 #Q 
 

 
 Best Qry 
 

 
 Best Eval 
 

 
 Avg Rank 
 

 
 Aln Len 
 

 
 Qry Len 
 

 
 IDOP? 
 

 
 adj? 
 

 

 

 
GKFG-1361-MONOMER
 

 
hypothetical protein; K06
 

 
1.5421621e-5
 

 
1.0
 

 
P00491
 

 
0.0990956
 

 
1.0
 

 
0.18685122
 

 
289
 

 
T
 

 
NIL
 

 

 
     Evidence for    ADDALT-RXN 
  
no BLAST hits found
 
     Evidence for D-deoxyribose 1,5-phosphomutase, EC# 5.4.2.7   D-PPENTOMUT-RXN 
  

 

 
 Hit 
 

 
 Common 
 

 
 P 
 

 
 #Q 
 

 
 Best Qry 
 

 
 Best Eval 
 

 
 Avg Rank 
 

 
 Aln Len 
 

 
 Qry Len 
 

 
 IDOP? 
 

 
 adj? 
 

 

 

 
GKFG-591-MONOMER
 

 
type I phosphodiesterase/
 

 
1.3945618e-5
 

 
1.0
 

 
P0A6K6
 

 
0.0964581
 

 
1.0
 

 
0.1007371
 

 
407
 

 
T
 

 
NIL
 

 

 
     

  adenosine nucleotides degradation I     Total # of reactions in pathway = 8   Present reactions: 3
  

 

 
 Reaction 
 

 
 Protein(s) 
 

 

 

 
IMP-DEHYDROG-RXN
 

 
(# 
 # 
 # )
 

 

 

 
XMPXAN-RXN
 

 
(# )
 

 

 

 
RXN0-363
 

 
(# )
 

 

 
   Missing reactions: 5
  

 

 
 AMP-DEAMINASE-RXN 
 

 

 

 
 RXN-7607 
 

 

 

 
 INOSINE-NUCLEOSIDASE-RXN 
 

 

 

 
 RXN0-901 
 

 

 

 
 RXN-7682 
 

 

 
        Evidence for AMP deaminase, EC# 3.5.4.6   AMP-DEAMINASE-RXN 
  

 

 
 Hit 
 

 
 Common 
 

 
 P 
 

 
 #Q 
 

 
 Best Qry 
 

 
 Best Eval 
 

 
 Avg Rank 
 

 
 Aln Len 
 

 
 Qry Len 
 

 
 IDOP? 
 

 
 adj? 
 

 

 

 
GKFG-844-MONOMER
 

 
ahe:Arch_0366 ATP synthas
 

 
1.3945618e-5
 

 
2.0
 

 
Q01432
 

 
0.0552658
 

 
1.0
 

 
0.05623612
 

 
767
 

 
T
 

 
NIL
 

 

 
     Evidence for , EC# 3.1.3.5   RXN-7607 
  

 

 
 Hit 
 

 
 Common 
 

 
 P 
 

 
 #Q 
 

 
 Best Qry 
 

 
 Best Eval 
 

 
 Avg Rank 
 

 
 Aln Len 
 

 
 Qry Len 
 

 
 IDOP? 
 

 
 adj? 
 

 

 

 
GKFG-2023-MONOMER
 

 
mcu:HMPREF0573_11895 hypo
 

 
0.8538447
 

 
1.0
 

 
P0AF24
 

 
1.76344e-21
 

 
1.0
 

 
0.912
 

 
250
 

 
T
 

 
NIL
 

 

 

 
GKFG-1734-MONOMER
 

 
pad:TIIST44_04425 HAD hyd
 

 
0.46065697
 

 
1.0
 

 
P0AF24
 

 
3.39614e-18
 

 
2.0
 

 
0.956
 

 
250
 

 
T
 

 
NIL
 

 

 
     Evidence for inosine nucleosidase, EC# 3.2.2.2   INOSINE-NUCLEOSIDASE-RXN 
  

 

 
 Hit 
 

 
 Common 
 

 
 P 
 

 
 #Q 
 

 
 Best Qry 
 

 
 Best Eval 
 

 
 Avg Rank 
 

 
 Aln Len 
 

 
 Qry Len 
 

 
 IDOP? 
 

 
 adj? 
 

 

 

 
GKFG-890-MONOMER
 

 
aau:AAur_3646 inosine-uri
 

 
0.9980101
 

 
2.0
 

 
P22564
 

 
2.3909e-52
 

 
1.0
 

 
0.91400373
 

 
304
 

 
T
 

 
NIL
 

 

 

 
GKFG-842-MONOMER
 

 
F0F1-type ATP synthase su
 

 
2.795795e-6
 

 
1.0
 

 
Q9SJM7
 

 
0.0853741
 

 
2.0
 

 
0.15178572
 

 
336
 

 
T
 

 
NIL
 

 

 
     Evidence for xanthine dehydrogenase, EC# 1.17.1.4   RXN0-901 
  

 

 
 Hit 
 

 
 Common 
 

 
 P 
 

 
 #Q 
 

 
 Best Qry 
 

 
 Best Eval 
 

 
 Avg Rank 
 

 
 Aln Len 
 

 
 Qry Len 
 

 
 IDOP? 
 

 
 adj? 
 

 

 

 
GKFG-603-MONOMER
 

 
jde:Jden_1438 phosphofruc
 

 
0.0018147272
 

 
1.0
 

 
O32145
 

 
0.554235
 

 
1.0
 

 
0.49097472
 

 
277
 

 
T
 

 
NIL
 

 

 

 
GKFG-1883-MONOMER
 

 
transmembrane_regions (db
 

 
3.182795e-4
 

 
1.0
 

 
O32143
 

 
0.281918
 

 
1.0
 

 
0.40462428
 

 
173
 

 
T
 

 
NIL
 

 

 

 
GKFG-1803-MONOMER
 

 
cfi:Celf_2027 protein-exp
 

 
1.5421621e-5
 

 
1.0
 

 
O32147
 

 
0.0745426
 

 
1.0
 

 
0.15454546
 

 
330
 

 
T
 

 
NIL
 

 

 

 
GKFG-2024-MONOMER
 

 
pad:TIIST44_04395 glycero
 

 
1.3945618e-5
 

 
1.0
 

 
Q12553
 

 
0.222902
 

 
1.0
 

 
0.1056493
 

 
1363
 

 
T
 

 
NIL
 

 

 

 
GKFG-1956-MONOMER
 

 
bsd:BLASA_2671 pafA; pup-
 

 
1.3945618e-5
 

 
1.0
 

 
P77324
 

 
0.91284
 

 
1.0
 

 
0.10062893
 

 
318
 

 
T
 

 
NIL
 

 

 
     Evidence for , EC# 1.17.1.4   RXN-7682 
  
no BLAST hits found
 
     

  pyrimidine deoxyribonucleosides degradation     Total # of reactions in pathway = 6   Present reactions: 2
  

 

 
 Reaction 
 

 
 Protein(s) 
 

 

 

 
DEOXYRIBOSE-P-ALD-RXN
 

 
(# )
 

 

 

 
ACETALD-DEHYDROG-RXN
 

 
(# )
 

 

 
   Missing reactions: 4
  

 

 
 CYTIDEAM-RXN 
 

 

 

 
 URA-PHOSPH-RXN 
 

 

 

 
 THYM-PHOSPH-RXN 
 

 

 

 
 D-PPENTOMUT-RXN 
 

 

 
        Evidence for deoxycytidine deaminase, EC# 3.5.4.14   CYTIDEAM-RXN 
  
no BLAST hits found
 
     Evidence for    URA-PHOSPH-RXN 
  
no BLAST hits found
 
     Evidence for thymidine phosphorylase, EC# 2.4.2.4   THYM-PHOSPH-RXN 
  

 

 
 Hit 
 

 
 Common 
 

 
 P 
 

 
 #Q 
 

 
 Best Qry 
 

 
 Best Eval 
 

 
 Avg Rank 
 

 
 Aln Len 
 

 
 Qry Len 
 

 
 IDOP? 
 

 
 adj? 
 

 

 

 
GKFG-1012-MONOMER
 

 
krh:KRH_10210 ilvB; aceto
 

 
3.182795e-4
 

 
1.0
 

 
Q0KA59
 

 
0.298569
 

 
1.0
 

 
0.3673077
 

 
520
 

 
T
 

 
NIL
 

 

 

 
GKFG-1243-MONOMER
 

 
hypothetical protein (db=
 

 
1.5421621e-5
 

 
1.0
 

 
O28927
 

 
0.0181538
 

 
1.0
 

 
0.18019801
 

 
505
 

 
T
 

 
NIL
 

 

 

 
GKFG-1567-MONOMER
 

 
hypothetical protein (db=
 

 
1.5421621e-5
 

 
1.0
 

 
Q3IP82
 

 
0.94675
 

 
1.0
 

 
0.176
 

 
500
 

 
T
 

 
NIL
 

 

 

 
GKFG-1317-MONOMER
 

 
bfa:Bfae_13380 UDP-N-acet
 

 
1.5421621e-5
 

 
1.0
 

 
Q483R6
 

 
0.666039
 

 
1.0
 

 
0.14840183
 

 
438
 

 
T
 

 
NIL
 

 

 

 
GKFG-1121-MONOMER
 

 
UniRef90_H5UW39 Putative 
 

 
1.3945618e-5
 

 
1.0
 

 
A8IA58
 

 
0.137108
 

 
1.0
 

 
0.09090909
 

 
693
 

 
T
 

 
NIL
 

 

 

 
GKFG-1741-MONOMER
 

 
ABC transporter ATP-bindi
 

 
1.3945618e-5
 

 
1.0
 

 
Q16A32
 

 
0.151935
 

 
1.0
 

 
0.028064992
 

 
677
 

 
T
 

 
NIL
 

 

 

 
GKFG-340-MONOMER
 

 
UniRef90_C0VYI5 ABC super
 

 
6.867209e-6
 

 
2.0
 

 
A8IA58
 

 
0.155193
 

 
2.0
 

 
0.030210745
 

 
693
 

 
NIL
 

 
NIL
 

 

 

 
GKFG-102-MONOMER
 

 
mcu:HMPREF0573_11535 ABC 
 

 
2.9678517e-6
 

 
2.0
 

 
A8IA58
 

 
0.171672
 

 
3.0
 

 
0.0466876
 

 
693
 

 
NIL
 

 
NIL
 

 

 

 
GKFG-1832-MONOMER
 

 
cytidylate kinase (EC:2.7
 

 
1.4610267e-6
 

 
1.0
 

 
A8IA58
 

 
0.277365
 

 
4.0
 

 
0.043290045
 

 
693
 

 
T
 

 
NIL
 

 

 

 
GKFG-1615-MONOMER
 

 
ica:Intca_0241 exonucleas
 

 
1.0926308e-6
 

 
3.0
 

 
Q46UT0
 

 
0.143909
 

 
2.6666667
 

 
0.09002874
 

 
602
 

 
T
 

 
NIL
 

 

 
     Evidence for D-deoxyribose 1,5-phosphomutase, EC# 5.4.2.7   D-PPENTOMUT-RXN 
  

 

 
 Hit 
 

 
 Common 
 

 
 P 
 

 
 #Q 
 

 
 Best Qry 
 

 
 Best Eval 
 

 
 Avg Rank 
 

 
 Aln Len 
 

 
 Qry Len 
 

 
 IDOP? 
 

 
 adj? 
 

 

 

 
GKFG-591-MONOMER
 

 
type I phosphodiesterase/
 

 
1.3945618e-5
 

 
1.0
 

 
P0A6K6
 

 
0.0964581
 

 
1.0
 

 
0.1007371
 

 
407
 

 
T
 

 
NIL
 

 

 
     

  nitrate reduction VI (assimilatory)     Total # of reactions in pathway = 4   Present reactions: 2
  

 

 
 Reaction 
 

 
 Protein(s) 
 

 

 

 
GLUTAMINESYN-RXN
 

 
(# 
 # )
 

 

 

 
GLUTDEHYD-RXN
 

 
(# 
 # )
 

 

 
   Missing reactions: 2
  

 

 
 1.7.7.2-RXN 
 

 

 

 
 FERREDOXIN--NITRITE-REDUCTASE-RXN 
 

 

 
        Evidence for ferredoxin&mdash;nitrate reductase, EC# 1.7.7.2   1.7.7.2-RXN 
  

 

 
 Hit 
 

 
 Common 
 

 
 P 
 

 
 #Q 
 

 
 Best Qry 
 

 
 Best Eval 
 

 
 Avg Rank 
 

 
 Aln Len 
 

 
 Qry Len 
 

 
 IDOP? 
 

 
 adj? 
 

 

 

 
GKFG-167-MONOMER
 

 
jde:Jden_0545 NADH-quinon
 

 
0.36918753
 

 
1.0
 

 
P39458
 

 
6.31293e-15
 

 
1.0
 

 
0.6227709
 

 
729
 

 
T
 

 
NIL
 

 

 

 
GKFG-805-MONOMER
 

 
ske:Sked_35330 exodeoxyri
 

 
2.5282066e-6
 

 
1.0
 

 
P39458
 

 
0.331332
 

 
2.0
 

 
0.0521262
 

 
729
 

 
T
 

 
NIL
 

 

 
     Evidence for ferredoxin&mdash;nitrite reductase, EC# 1.7.7.1   FERREDOXIN--NITRITE-REDUCTASE-RXN 
  

 

 
 Hit 
 

 
 Common 
 

 
 P 
 

 
 #Q 
 

 
 Best Qry 
 

 
 Best Eval 
 

 
 Avg Rank 
 

 
 Aln Len 
 

 
 Qry Len 
 

 
 IDOP? 
 

 
 adj? 
 

 

 

 
GKFG-1558-MONOMER
 

 
seg (db=Seg db_id=seg fro
 

 
1.5421621e-5
 

 
1.0
 

 
P39661
 

 
0.0950127
 

 
1.0
 

 
0.15234375
 

 
512
 

 
T
 

 
NIL
 

 

 

 
GKFG-179-MONOMER
 

 
sugar phosphate isomerase
 

 
1.3945618e-5
 

 
1.0
 

 
Q42997
 

 
0.682099
 

 
1.0
 

 
0.10402685
 

 
596
 

 
T
 

 
NIL
 

 

 
     

  ( R )-cysteate degradation     Total # of reactions in pathway = 3   Present reactions: 1
  

 

 
 Reaction 
 

 
 Protein(s) 
 

 

 

 
RXN-11737
 

 
(# )
 

 

 
   Missing reactions: 2
  

 

 
 R230-RXN 
 

 

 

 
 RXN-11691 
 

 

 
        Evidence for , EC# 1.1.1.272   R230-RXN 
  
no BLAST hits found
 
     Evidence for (2 R )-sulfolactate sulfo-lyase, EC# 4.4.1.24   RXN-11691 
  

 

 
 Hit 
 

 
 Common 
 

 
 P 
 

 
 #Q 
 

 
 Best Qry 
 

 
 Best Eval 
 

 
 Avg Rank 
 

 
 Aln Len 
 

 
 Qry Len 
 

 
 IDOP? 
 

 
 adj? 
 

 

 

 
GKFG-1368-MONOMER
 

 
bfa:Bfae_10530 recombinat
 

 
3.7905585e-4
 

 
1.0
 

 
Q0RXU4
 

 
0.49588
 

 
1.0
 

 
0.3529412
 

 
102
 

 
NIL
 

 
NIL
 

 

 
     

  sulfolactate degradation III     Total # of reactions in pathway = 3   Present reactions: 1
  

 

 
 Reaction 
 

 
 Protein(s) 
 

 

 

 
RXN-11737
 

 
(# )
 

 

 
   Missing reactions: 2
  

 

 
 RXN-11738 
 

 

 

 
 4.4.1.25-RXN 
 

 

 
        Evidence for sulfolactate dehydrogenase, EC# 1.1.1.-   RXN-11738 
  
no BLAST hits found
 
     Evidence for  L -cysteate sulfo-lyase, EC# 4.4.1.25   4.4.1.25-RXN 
  

 

 
 Hit 
 

 
 Common 
 

 
 P 
 

 
 #Q 
 

 
 Best Qry 
 

 
 Best Eval 
 

 
 Avg Rank 
 

 
 Aln Len 
 

 
 Qry Len 
 

 
 IDOP? 
 

 
 adj? 
 

 

 

 
GKFG-1551-MONOMER
 

 
threonine dehydratase (EC
 

 
3.7905585e-4
 

 
1.0
 

 
A3SQG3
 

 
0.00752314
 

 
1.0
 

 
0.26331362
 

 
338
 

 
NIL
 

 
NIL
 

 

 

 
GKFG-339-MONOMER
 

 
ABC transporter permease;
 

 
2.5307965e-5
 

 
1.0
 

 
A3SQG3
 

 
0.571056
 

 
2.0
 

 
0.3106509
 

 
338
 

 
T
 

 
NIL
 

 

 
     

  androstenedione degradation     Total # of reactions in pathway = 14   Present reactions: 1
  

 

 
 Reaction 
 

 
 Protein(s) 
 

 

 

 
RXN-12746
 

 
(# 
 # 
 # 
 # )
 

 

 
   Missing reactions: 13
  

 

 
 RXN-12691 
 

 

 

 
 RXN-12714 
 

 

 

 
 RXN-12717 
 

 

 

 
 1.13.11.25-RXN 
 

 

 

 
 RXN-12718 
 

 

 

 
 RXN-12735 
 

 

 

 
 RXN-12734 
 

 

 

 
 RXN-12736 
 

 

 

 
 RXN-12747 
 

 

 

 
 RXN-12748 
 

 

 

 
 RXN-12749 
 

 

 

 
 RXN-12750 
 

 

 

 
 RXN-12751 
 

 

 
        Evidence for androst-dien-3,17-dione hydroxylase, EC# 1.14.13.-   RXN-12691 
  

 

 
 Hit 
 

 
 Common 
 

 
 P 
 

 
 #Q 
 

 
 Best Qry 
 

 
 Best Eval 
 

 
 Avg Rank 
 

 
 Aln Len 
 

 
 Qry Len 
 

 
 IDOP? 
 

 
 adj? 
 

 

 

 
GKFG-413-MONOMER
 

 
UniRef90_F3P5U3 Putative 
 

 
3.787886e-5
 

 
1.0
 

 
P71875
 

 
0.711966
 

 
1.0
 

 
0.08290155
 

 
386
 

 
NIL
 

 
NIL
 

 

 

 
GKFG-1887-MONOMER
 

 
nca:Noca_4168 phosphoenol
 

 
2.795795e-6
 

 
1.0
 

 
tr|B6V6V5|B6V6V5_RHORH
 

 
0.0709635
 

 
2.0
 

 
0.22486773
 

 
378
 

 
T
 

 
NIL
 

 

 

 
GKFG-2010-MONOMER
 

 
Rieske-type ferredoxin; K
 

 
2.5282066e-6
 

 
2.0
 

 
tr|B6V6V5|B6V6V5_RHORH
 

 
0.0511179
 

 
1.5
 

 
0.11508621
 

 
378
 

 
T
 

 
NIL
 

 

 
     Evidence for androst-4-en-3,17-dione 1-dehydrogenase, EC# 1.3.99.4   RXN-12714 
  

 

 
 Hit 
 

 
 Common 
 

 
 P 
 

 
 #Q 
 

 
 Best Qry 
 

 
 Best Eval 
 

 
 Avg Rank 
 

 
 Aln Len 
 

 
 Qry Len 
 

 
 IDOP? 
 

 
 adj? 
 

 

 

 
GKFG-845-MONOMER
 

 
ahe:Arch_0365 ATP synthas
 

 
1.3945618e-5
 

 
1.0
 

 
tr|Q9RA02|Q9RA02_RHOER
 

 
0.402075
 

 
1.0
 

 
0.11176471
 

 
510
 

 
T
 

 
NIL
 

 

 

 
GKFG-1193-MONOMER
 

 
pfr:PFREUD_12990 glpA; an
 

 
1.3945618e-5
 

 
1.0
 

 
P71864
 

 
0.035202
 

 
1.0
 

 
0.08703375
 

 
563
 

 
T
 

 
NIL
 

 

 

 
GKFG-549-MONOMER
 

 
iva:Isova_3010 thioredoxi
 

 
3.968502e-6
 

 
1.0
 

 
P71864
 

 
0.79482
 

 
4.0
 

 
0.06571936
 

 
563
 

 
NIL
 

 
NIL
 

 

 

 
GKFG-900-MONOMER
 

 
ahe:Arch_1354 electron-tr
 

 
2.5282066e-6
 

 
1.0
 

 
P71864
 

 
0.0441302
 

 
2.0
 

 
0.09413854
 

 
563
 

 
T
 

 
NIL
 

 

 

 
GKFG-795-MONOMER
 

 
bcv:Bcav_1171 UDP-galacto
 

 
1.0926308e-6
 

 
1.0
 

 
P71864
 

 
0.129409
 

 
3.0
 

 
0.06749556
 

 
563
 

 
T
 

 
NIL
 

 

 
     Evidence for 3-hydroxy-9,10-secoandrosta-1,3,5(10)-triene-9,17-dione monooxygenase, EC# 1.14.14.12   RXN-12717 
  

 

 
 Hit 
 

 
 Common 
 

 
 P 
 

 
 #Q 
 

 
 Best Qry 
 

 
 Best Eval 
 

 
 Avg Rank 
 

 
 Aln Len 
 

 
 Qry Len 
 

 
 IDOP? 
 

 
 adj? 
 

 

 

 
GKFG-907-MONOMER
 

 
pfr:PFREUD_02420 caiA; cr
 

 
0.0018147272
 

 
2.0
 

 
Q0S811
 

 
0.00675863
 

 
1.0
 

 
0.5456853
 

 
382
 

 
T
 

 
NIL
 

 

 
     Evidence for 3,4-dihydroxy-9,10-secoandrosta-1,3,5(10)-triene-9,17-dione 4,5-dioxygenase, EC# 1.13.11.25   1.13.11.25-RXN 
  

 

 
 Hit 
 

 
 Common 
 

 
 P 
 

 
 #Q 
 

 
 Best Qry 
 

 
 Best Eval 
 

 
 Avg Rank 
 

 
 Aln Len 
 

 
 Qry Len 
 

 
 IDOP? 
 

 
 adj? 
 

 

 

 
GKFG-316-MONOMER
 

 
putative secreted protein
 

 
3.7905585e-4
 

 
1.0
 

 
tr|Q9FAE3|Q9FAE3_COMTE
 

 
0.489193
 

 
1.0
 

 
0.28333333
 

 
300
 

 
NIL
 

 
NIL
 

 

 

 
GKFG-295-MONOMER
 

 
hypothetical protein (db=
 

 
7.594039e-6
 

 
1.0
 

 
tr|Q9FAE3|Q9FAE3_COMTE
 

 
0.914294
 

 
2.0
 

 
0.12666667
 

 
300
 

 
NIL
 

 
NIL
 

 

 
     Evidence for 4,5-9,10-diseco-3-hydroxy-5,9,17-trioxoandrosta-1(10),2-diene-4-oate hydrolase, EC# 3.7.1.-   RXN-12718 
  

 

 
 Hit 
 

 
 Common 
 

 
 P 
 

 
 #Q 
 

 
 Best Qry 
 

 
 Best Eval 
 

 
 Avg Rank 
 

 
 Aln Len 
 

 
 Qry Len 
 

 
 IDOP? 
 

 
 adj? 
 

 

 

 
GKFG-1169-MONOMER
 

 
UniRef90_E6M3C9 Acyl-CoA 
 

 
0.008898924
 

 
1.0
 

 
tr|Q83VZ6|Q83VZ6_COMTE
 

 
2.60392e-5
 

 
1.0
 

 
0.83154124
 

 
279
 

 
T
 

 
NIL
 

 

 

 
GKFG-1906-MONOMER
 

 
mcu:HMPREF0573_11357 puta
 

 
0.0016251113
 

 
2.0
 

 
Q9KWQ6
 

 
6.86048e-5
 

 
2.0
 

 
0.823624
 

 
292
 

 
T
 

 
NIL
 

 

 

 
GKFG-149-MONOMER
 

 
UniRef90_C0VYV3 Lysophosp
 

 
2.4681233e-4
 

 
1.0
 

 
tr|Q83VZ6|Q83VZ6_COMTE
 

 
0.0800367
 

 
6.0
 

 
0.31182796
 

 
279
 

 
NIL
 

 
NIL
 

 

 

 
GKFG-864-MONOMER
 

 
crd:CRES_0981 pip; prolin
 

 
1.9042875e-4
 

 
1.0
 

 
tr|Q83VZ6|Q83VZ6_COMTE
 

 
5.74264e-4
 

 
4.0
 

 
0.50537634
 

 
279
 

 
T
 

 
NIL
 

 

 

 
GKFG-1919-MONOMER
 

 
putative acyl-CoA thioest
 

 
1.4241917e-4
 

 
2.0
 

 
tr|Q83VZ6|Q83VZ6_COMTE
 

 
1.21373e-4
 

 
2.5
 

 
0.5572065
 

 
279
 

 
T
 

 
NIL
 

 

 

 
GKFG-836-MONOMER
 

 
alpha/beta hydrolase (db=
 

 
2.4944004e-5
 

 
3.0
 

 
Q9KWQ6
 

 
4.17486e-4
 

 
2.6666667
 

 
0.4621066
 

 
292
 

 
T
 

 
NIL
 

 

 
     Evidence for 4-hydroxy-2-oxohexanoate aldolase, EC# 4.1.3.-   RXN-12735 
  
no BLAST hits found
 
     Evidence for 2-hydroxyhexa-2,4-dienoate hydratase, EC# 4.2.1.-   RXN-12734 
  
no BLAST hits found
 
     Evidence for propanal dehydrogenase (acetylating), EC# 1.2.1.-   RXN-12736 
  

 

 
 Hit 
 

 
 Common 
 

 
 P 
 

 
 #Q 
 

 
 Best Qry 
 

 
 Best Eval 
 

 
 Avg Rank 
 

 
 Aln Len 
 

 
 Qry Len 
 

 
 IDOP? 
 

 
 adj? 
 

 

 

 
GKFG-960-MONOMER
 

 
isochorismate synthase; K
 

 
1.3945618e-5
 

 
1.0
 

 
Q0S816
 

 
0.657121
 

 
1.0
 

 
0.07333333
 

 
300
 

 
T
 

 
NIL
 

 

 
     Evidence for 5-hydroxy-3-[(3a S ,4 S ,5 R ,7a S )-7a-methyl-1,5-dioxo-octahydro-1 H -inden-4-yl]propanoyl-CoA 5-dehydrogenase, EC# 1.1.1.145   RXN-12747      Evidence for 5-hydroxy-3-[(3a S ,4 S ,5 R ,7a S )-7a-methyl-1,5-dioxo-octahydro-1 H -inden-4-yl]propanoyl-CoA dehydrogenase, EC# 1.3.99.-   RXN-12748 
  

 

 
 Hit 
 

 
 Common 
 

 
 P 
 

 
 #Q 
 

 
 Best Qry 
 

 
 Best Eval 
 

 
 Avg Rank 
 

 
 Aln Len 
 

 
 Qry Len 
 

 
 IDOP? 
 

 
 adj? 
 

 

 

 
GKFG-907-MONOMER
 

 
pfr:PFREUD_02420 caiA; cr
 

 
0.36918753
 

 
2.0
 

 
tr|E4WD16|E4WD16_RHOE1
 

 
2.01884e-11
 

 
1.0
 

 
0.688186
 

 
403
 

 
T
 

 
NIL
 

 

 
     Evidence for (2 E )-3-[(3a S ,4 S ,5 R ,7a S )-5-hydroxy-7a-methyl-1-oxo-octahydro-1 H -inden-4-yl]prop-2-enoate hydratase, EC# 4.2.1.-   RXN-12749      Evidence for 3-[(3a S ,4 S ,5 R ,7a S )-5-hydroxy-7a-methyl-1-oxo-octahydro-1 H -inden-4-yl]-3-hydroxypropanoate dehydrogenase, EC# 1.1.1.35   RXN-12750      Evidence for 3-[(3a S ,4 S ,5 R ,7a S )-5-hydroxy-7a-methyl-1-oxo-octahydro-1 H -inden-4-yl]-3-oxopropanoyl-CoA hydrolase, EC# 3.1.2.-   RXN-12751      

  sitosterol degradation to androstenedione     Total # of reactions in pathway = 18   Present reactions: 2
  

 

 
 Reaction 
 

 
 Protein(s) 
 

 

 

 
RXN-12706
 

 
(# )
 

 

 

 
RXN-12710
 

 
(# )
 

 

 
   Missing reactions: 16
  

 

 
 RXN-12789 
 

 

 

 
 RXN-12790 
 

 

 

 
 RXN-12788 
 

 

 

 
 RXN-12787 
 

 

 

 
 RXN-12784 
 

 

 

 
 RXN-12785 
 

 

 

 
 RXN-12782 
 

 

 

 
 RXN-12781 
 

 

 

 
 RXN-12780 
 

 

 

 
 RXN-12786 
 

 

 

 
 RXN-12707 
 

 

 

 
 RXN-12708 
 

 

 

 
 RXN-12709 
 

 

 

 
 RXN-12743 
 

 

 

 
 RXN-12744 
 

 

 

 
 RXN-12745 
 

 

 
        Evidence for sitosterol dehydrogenase, EC# 1.1.1.145   RXN-12789      Evidence for 3-oxo-24-ethyl-cholest-5-ene isomerase, EC# 5.3.3.1   RXN-12790      Evidence for 3-oxo-24-ethyl-cholest-4-ene C-26 monooxygenase, EC# 1.14.13.-   RXN-12788      Evidence for 3-oxo-24-ethyl-26-hydroxy-cholest-4-ene dehydrogenase, EC# 1.1.1.-   RXN-12787      Evidence for 3-oxo-24-ethyl-cholest-4-en-26-oyl-CoA dehydrogenase, EC# 1.3.99.-   RXN-12784      Evidence for 3-oxo-24-ethyl-cholest-4-en-26-oate:CoA ligase, EC# 6.2.1.-   RXN-12785      Evidence for 3-oxo-24-ethyl-cholest-4,24-dien-26-oyl-CoA carboxylase, EC# 6.4.1.-   RXN-12782      Evidence for 3-oxo-24-(isopropanoate)-cholest-4,24-dien-26-oyl-CoA hydrolase, EC# 3.1.2.-   RXN-12781      Evidence for 3,24-dioxo-cholest-4-en-26-oate:CoA ligase, EC# 6.2.1.-   RXN-12780      Evidence for 3-oxo-24-ethyl-26-al-cholest-4-ene dehydrogenase, EC# 1.2.1.-   RXN-12786      Evidence for , EC# 1.3.99.-   RXN-12707      Evidence for , EC# 4.2.1.-   RXN-12708      Evidence for , EC# 1.1.1.35   RXN-12709      Evidence for 3-oxo-23,24-bisnorchol-4-en-22-oyl-CoA 17(20)-dehydrogenase, EC# 1.3.99.-   RXN-12743 
  

 

 
 Hit 
 

 
 Common 
 

 
 P 
 

 
 #Q 
 

 
 Best Qry 
 

 
 Best Eval 
 

 
 Avg Rank 
 

 
 Aln Len 
 

 
 Qry Len 
 

 
 IDOP? 
 

 
 adj? 
 

 

 

 
GKFG-907-MONOMER
 

 
pfr:PFREUD_02420 caiA; cr
 

 
0.5061234
 

 
4.0
 

 
tr|E4WCW5|E4WCW5_RHOE1
 

 
7.94942e-13
 

 
1.0
 

 
0.75395125
 

 
357
 

 
T
 

 
NIL
 

 

 

 
GKFG-130-MONOMER
 

 
sortase family protein; K
 

 
6.867209e-6
 

 
1.0
 

 
tr|E4WCW5|E4WCW5_RHOE1
 

 
0.311287
 

 
2.0
 

 
0.1092437
 

 
357
 

 
NIL
 

 
NIL
 

 

 

 
GKFG-1489-MONOMER
 

 
lacto-N-biosidase (EC:3.2
 

 
2.795795e-6
 

 
1.0
 

 
tr|E4WCW6|E4WCW6_RHOE1
 

 
0.199153
 

 
2.0
 

 
0.22774869
 

 
382
 

 
T
 

 
NIL
 

 

 
     Evidence for 3-oxo-23,24-bisnorchol-4,17(20)-dien-22-oyl-CoA hydratase, EC# 4.2.1.-   RXN-12744 
  

 

 
 Hit 
 

 
 Common 
 

 
 P 
 

 
 #Q 
 

 
 Best Qry 
 

 
 Best Eval 
 

 
 Avg Rank 
 

 
 Aln Len 
 

 
 Qry Len 
 

 
 IDOP? 
 

 
 adj? 
 

 

 

 
GKFG-1456-MONOMER
 

 
ahe:Arch_1237 peptidase M
 

 
1.3958463e-4
 

 
1.0
 

 
tr|E4WCW8|E4WCW8_RHOE1
 

 
0.0482813
 

 
1.0
 

 
0.31428573
 

 
140
 

 
T
 

 
NIL
 

 

 

 
GKFG-1621-MONOMER
 

 
tcu:Tcur_3714 3-phosphosh
 

 
1.3958463e-4
 

 
1.0
 

 
tr|E4WCW7|E4WCW7_RHOE1
 

 
0.25286
 

 
1.0
 

 
0.25671643
 

 
335
 

 
T
 

 
NIL
 

 

 
     Evidence for 3-oxo-23,24-bisnorchol-4-en-17-ol-22-oyl-CoA lyase, EC# 2.3.1.-   RXN-12745 
  

 

 
 Hit 
 

 
 Common 
 

 
 P 
 

 
 #Q 
 

 
 Best Qry 
 

 
 Best Eval 
 

 
 Avg Rank 
 

 
 Aln Len 
 

 
 Qry Len 
 

 
 IDOP? 
 

 
 adj? 
 

 

 

 
GKFG-1625-MONOMER
 

 
cga:Celgi_2334 2-oxogluta
 

 
1.3958463e-4
 

 
1.0
 

 
tr|P71861|P71861_MYCTU
 

 
0.690409
 

 
1.0
 

 
0.26165804
 

 
386
 

 
T
 

 
NIL
 

 

 
     

  cholesterol degradation to androstenedione I (cholesterol oxidase)     Total # of reactions in pathway = 17   Present reactions: 3
  

 

 
 Reaction 
 

 
 Protein(s) 
 

 

 

 
RXN-12702
 

 
(# 
 # 
 # 
 # )
 

 

 

 
RXN-12706
 

 
(# )
 

 

 

 
RXN-12710
 

 
(# )
 

 

 
   Missing reactions: 14
  

 

 
 RXN-12703 
 

 

 

 
 RXN-12704 
 

 

 

 
 RXN-12705 
 

 

 

 
 RXN-12707 
 

 

 

 
 RXN-12708 
 

 

 

 
 RXN-12709 
 

 

 

 
 RXN-12694 
 

 

 

 
 RXN-12778 
 

 

 

 
 RXN-12743 
 

 

 

 
 RXN-12744 
 

 

 

 
 RXN-12745 
 

 

 

 
 RXN-12848 
 

 

 

 
 RXN-12850 
 

 

 

 
 RXN-12849 
 

 

 
        Evidence for cholest-4-en-3,27-dione-26-CoA dehydrogenase, EC# 1.3.99.-   RXN-12703      Evidence for cholest-4,24-dien-3,27-dione-26-CoA hydratase, EC# 4.2.1.-   RXN-12704      Evidence for 3-hydroxyacyl-CoA dehydrogenase, EC# 1.1.1.35   RXN-12705      Evidence for , EC# 1.3.99.-   RXN-12707      Evidence for , EC# 4.2.1.-   RXN-12708      Evidence for , EC# 1.1.1.35   RXN-12709      Evidence for cholest-5-en-3-one isomerase, EC# 5.3.3.1   RXN-12694 
  
no BLAST hits found
 
     Evidence for , EC# 1.1.3.6   RXN-12778 
  
no BLAST hits found
 
     Evidence for 3-oxo-23,24-bisnorchol-4-en-22-oyl-CoA 17(20)-dehydrogenase, EC# 1.3.99.-   RXN-12743 
  

 

 
 Hit 
 

 
 Common 
 

 
 P 
 

 
 #Q 
 

 
 Best Qry 
 

 
 Best Eval 
 

 
 Avg Rank 
 

 
 Aln Len 
 

 
 Qry Len 
 

 
 IDOP? 
 

 
 adj? 
 

 

 

 
GKFG-907-MONOMER
 

 
pfr:PFREUD_02420 caiA; cr
 

 
0.5061234
 

 
4.0
 

 
tr|E4WCW5|E4WCW5_RHOE1
 

 
7.94942e-13
 

 
1.0
 

 
0.75395125
 

 
357
 

 
T
 

 
NIL
 

 

 

 
GKFG-130-MONOMER
 

 
sortase family protein; K
 

 
6.867209e-6
 

 
1.0
 

 
tr|E4WCW5|E4WCW5_RHOE1
 

 
0.311287
 

 
2.0
 

 
0.1092437
 

 
357
 

 
NIL
 

 
NIL
 

 

 

 
GKFG-1489-MONOMER
 

 
lacto-N-biosidase (EC:3.2
 

 
2.795795e-6
 

 
1.0
 

 
tr|E4WCW6|E4WCW6_RHOE1
 

 
0.199153
 

 
2.0
 

 
0.22774869
 

 
382
 

 
T
 

 
NIL
 

 

 
     Evidence for 3-oxo-23,24-bisnorchol-4,17(20)-dien-22-oyl-CoA hydratase, EC# 4.2.1.-   RXN-12744 
  

 

 
 Hit 
 

 
 Common 
 

 
 P 
 

 
 #Q 
 

 
 Best Qry 
 

 
 Best Eval 
 

 
 Avg Rank 
 

 
 Aln Len 
 

 
 Qry Len 
 

 
 IDOP? 
 

 
 adj? 
 

 

 

 
GKFG-1456-MONOMER
 

 
ahe:Arch_1237 peptidase M
 

 
1.3958463e-4
 

 
1.0
 

 
tr|E4WCW8|E4WCW8_RHOE1
 

 
0.0482813
 

 
1.0
 

 
0.31428573
 

 
140
 

 
T
 

 
NIL
 

 

 

 
GKFG-1621-MONOMER
 

 
tcu:Tcur_3714 3-phosphosh
 

 
1.3958463e-4
 

 
1.0
 

 
tr|E4WCW7|E4WCW7_RHOE1
 

 
0.25286
 

 
1.0
 

 
0.25671643
 

 
335
 

 
T
 

 
NIL
 

 

 
     Evidence for 3-oxo-23,24-bisnorchol-4-en-17-ol-22-oyl-CoA lyase, EC# 2.3.1.-   RXN-12745 
  

 

 
 Hit 
 

 
 Common 
 

 
 P 
 

 
 #Q 
 

 
 Best Qry 
 

 
 Best Eval 
 

 
 Avg Rank 
 

 
 Aln Len 
 

 
 Qry Len 
 

 
 IDOP? 
 

 
 adj? 
 

 

 

 
GKFG-1625-MONOMER
 

 
cga:Celgi_2334 2-oxogluta
 

 
1.3958463e-4
 

 
1.0
 

 
tr|P71861|P71861_MYCTU
 

 
0.690409
 

 
1.0
 

 
0.26165804
 

 
386
 

 
T
 

 
NIL
 

 

 
     Evidence for cholest-4-en-3-one C26-hydroxylase, EC# 1.14.13.-   RXN-12848 
  

 

 
 Hit 
 

 
 Common 
 

 
 P 
 

 
 #Q 
 

 
 Best Qry 
 

 
 Best Eval 
 

 
 Avg Rank 
 

 
 Aln Len 
 

 
 Qry Len 
 

 
 IDOP? 
 

 
 adj? 
 

 

 

 
GKFG-1062-MONOMER
 

 
mcu:HMPREF0573_10372 rho;
 

 
1.5421621e-5
 

 
1.0
 

 
Q0S7M1
 

 
0.0598865
 

 
1.0
 

 
0.1910828
 

 
471
 

 
T
 

 
NIL
 

 

 

 
GKFG-1464-MONOMER
 

 
mcu:HMPREF0573_11568 serA
 

 
2.5282066e-6
 

 
1.0
 

 
Q0S7M1
 

 
0.292173
 

 
2.0
 

 
0.07855626
 

 
471
 

 
T
 

 
NIL
 

 

 
     Evidence for 3-oxocholest-4-en-26-ol 26-dehydrogenase, EC# 1.1.1.-   RXN-12850 
  

 

 
 Hit 
 

 
 Common 
 

 
 P 
 

 
 #Q 
 

 
 Best Qry 
 

 
 Best Eval 
 

 
 Avg Rank 
 

 
 Aln Len 
 

 
 Qry Len 
 

 
 IDOP? 
 

 
 adj? 
 

 

 

 
GKFG-1062-MONOMER
 

 
mcu:HMPREF0573_10372 rho;
 

 
1.5421621e-5
 

 
1.0
 

 
Q0S7M1
 

 
0.0598865
 

 
1.0
 

 
0.1910828
 

 
471
 

 
T
 

 
NIL
 

 

 

 
GKFG-1464-MONOMER
 

 
mcu:HMPREF0573_11568 serA
 

 
2.5282066e-6
 

 
1.0
 

 
Q0S7M1
 

 
0.292173
 

 
2.0
 

 
0.07855626
 

 
471
 

 
T
 

 
NIL
 

 

 
     Evidence for 3-oxocholest-4-en-26-al 26-oxidoreductase, EC# 1.2.1.-   RXN-12849 
  

 

 
 Hit 
 

 
 Common 
 

 
 P 
 

 
 #Q 
 

 
 Best Qry 
 

 
 Best Eval 
 

 
 Avg Rank 
 

 
 Aln Len 
 

 
 Qry Len 
 

 
 IDOP? 
 

 
 adj? 
 

 

 

 
GKFG-1062-MONOMER
 

 
mcu:HMPREF0573_10372 rho;
 

 
1.5421621e-5
 

 
1.0
 

 
Q0S7M1
 

 
0.0598865
 

 
1.0
 

 
0.1910828
 

 
471
 

 
T
 

 
NIL
 

 

 

 
GKFG-1464-MONOMER
 

 
mcu:HMPREF0573_11568 serA
 

 
2.5282066e-6
 

 
1.0
 

 
Q0S7M1
 

 
0.292173
 

 
2.0
 

 
0.07855626
 

 
471
 

 
T
 

 
NIL
 

 

 
     

  cholesterol degradation to androstenedione II (cholesterol dehydrogenase)     Total # of reactions in pathway = 17   Present reactions: 3
  

 

 
 Reaction 
 

 
 Protein(s) 
 

 

 

 
RXN-12702
 

 
(# 
 # 
 # 
 # )
 

 

 

 
RXN-12706
 

 
(# )
 

 

 

 
RXN-12710
 

 
(# )
 

 

 
   Missing reactions: 14
  

 

 
 RXN-12693 
 

 

 

 
 RXN-12694 
 

 

 

 
 RXN-12703 
 

 

 

 
 RXN-12704 
 

 

 

 
 RXN-12705 
 

 

 

 
 RXN-12707 
 

 

 

 
 RXN-12708 
 

 

 

 
 RXN-12709 
 

 

 

 
 RXN-12743 
 

 

 

 
 RXN-12744 
 

 

 

 
 RXN-12745 
 

 

 

 
 RXN-12850 
 

 

 

 
 RXN-12849 
 

 

 

 
 RXN-12848 
 

 

 
        Evidence for cholesterol dehydrogenase, EC# 1.1.1.145   RXN-12693      Evidence for cholest-5-en-3-one isomerase, EC# 5.3.3.1   RXN-12694 
  
no BLAST hits found
 
     Evidence for cholest-4-en-3,27-dione-26-CoA dehydrogenase, EC# 1.3.99.-   RXN-12703      Evidence for cholest-4,24-dien-3,27-dione-26-CoA hydratase, EC# 4.2.1.-   RXN-12704      Evidence for 3-hydroxyacyl-CoA dehydrogenase, EC# 1.1.1.35   RXN-12705      Evidence for , EC# 1.3.99.-   RXN-12707      Evidence for , EC# 4.2.1.-   RXN-12708      Evidence for , EC# 1.1.1.35   RXN-12709      Evidence for 3-oxo-23,24-bisnorchol-4-en-22-oyl-CoA 17(20)-dehydrogenase, EC# 1.3.99.-   RXN-12743 
  

 

 
 Hit 
 

 
 Common 
 

 
 P 
 

 
 #Q 
 

 
 Best Qry 
 

 
 Best Eval 
 

 
 Avg Rank 
 

 
 Aln Len 
 

 
 Qry Len 
 

 
 IDOP? 
 

 
 adj? 
 

 

 

 
GKFG-907-MONOMER
 

 
pfr:PFREUD_02420 caiA; cr
 

 
0.5061234
 

 
4.0
 

 
tr|E4WCW5|E4WCW5_RHOE1
 

 
7.94942e-13
 

 
1.0
 

 
0.75395125
 

 
357
 

 
T
 

 
NIL
 

 

 

 
GKFG-130-MONOMER
 

 
sortase family protein; K
 

 
6.867209e-6
 

 
1.0
 

 
tr|E4WCW5|E4WCW5_RHOE1
 

 
0.311287
 

 
2.0
 

 
0.1092437
 

 
357
 

 
NIL
 

 
NIL
 

 

 

 
GKFG-1489-MONOMER
 

 
lacto-N-biosidase (EC:3.2
 

 
2.795795e-6
 

 
1.0
 

 
tr|E4WCW6|E4WCW6_RHOE1
 

 
0.199153
 

 
2.0
 

 
0.22774869
 

 
382
 

 
T
 

 
NIL
 

 

 
     Evidence for 3-oxo-23,24-bisnorchol-4,17(20)-dien-22-oyl-CoA hydratase, EC# 4.2.1.-   RXN-12744 
  

 

 
 Hit 
 

 
 Common 
 

 
 P 
 

 
 #Q 
 

 
 Best Qry 
 

 
 Best Eval 
 

 
 Avg Rank 
 

 
 Aln Len 
 

 
 Qry Len 
 

 
 IDOP? 
 

 
 adj? 
 

 

 

 
GKFG-1456-MONOMER
 

 
ahe:Arch_1237 peptidase M
 

 
1.3958463e-4
 

 
1.0
 

 
tr|E4WCW8|E4WCW8_RHOE1
 

 
0.0482813
 

 
1.0
 

 
0.31428573
 

 
140
 

 
T
 

 
NIL
 

 

 

 
GKFG-1621-MONOMER
 

 
tcu:Tcur_3714 3-phosphosh
 

 
1.3958463e-4
 

 
1.0
 

 
tr|E4WCW7|E4WCW7_RHOE1
 

 
0.25286
 

 
1.0
 

 
0.25671643
 

 
335
 

 
T
 

 
NIL
 

 

 
     Evidence for 3-oxo-23,24-bisnorchol-4-en-17-ol-22-oyl-CoA lyase, EC# 2.3.1.-   RXN-12745 
  

 

 
 Hit 
 

 
 Common 
 

 
 P 
 

 
 #Q 
 

 
 Best Qry 
 

 
 Best Eval 
 

 
 Avg Rank 
 

 
 Aln Len 
 

 
 Qry Len 
 

 
 IDOP? 
 

 
 adj? 
 

 

 

 
GKFG-1625-MONOMER
 

 
cga:Celgi_2334 2-oxogluta
 

 
1.3958463e-4
 

 
1.0
 

 
tr|P71861|P71861_MYCTU
 

 
0.690409
 

 
1.0
 

 
0.26165804
 

 
386
 

 
T
 

 
NIL
 

 

 
     Evidence for 3-oxocholest-4-en-26-ol 26-dehydrogenase, EC# 1.1.1.-   RXN-12850 
  

 

 
 Hit 
 

 
 Common 
 

 
 P 
 

 
 #Q 
 

 
 Best Qry 
 

 
 Best Eval 
 

 
 Avg Rank 
 

 
 Aln Len 
 

 
 Qry Len 
 

 
 IDOP? 
 

 
 adj? 
 

 

 

 
GKFG-1062-MONOMER
 

 
mcu:HMPREF0573_10372 rho;
 

 
1.5421621e-5
 

 
1.0
 

 
Q0S7M1
 

 
0.0598865
 

 
1.0
 

 
0.1910828
 

 
471
 

 
T
 

 
NIL
 

 

 

 
GKFG-1464-MONOMER
 

 
mcu:HMPREF0573_11568 serA
 

 
2.5282066e-6
 

 
1.0
 

 
Q0S7M1
 

 
0.292173
 

 
2.0
 

 
0.07855626
 

 
471
 

 
T
 

 
NIL
 

 

 
     Evidence for 3-oxocholest-4-en-26-al 26-oxidoreductase, EC# 1.2.1.-   RXN-12849 
  

 

 
 Hit 
 

 
 Common 
 

 
 P 
 

 
 #Q 
 

 
 Best Qry 
 

 
 Best Eval 
 

 
 Avg Rank 
 

 
 Aln Len 
 

 
 Qry Len 
 

 
 IDOP? 
 

 
 adj? 
 

 

 

 
GKFG-1062-MONOMER
 

 
mcu:HMPREF0573_10372 rho;
 

 
1.5421621e-5
 

 
1.0
 

 
Q0S7M1
 

 
0.0598865
 

 
1.0
 

 
0.1910828
 

 
471
 

 
T
 

 
NIL
 

 

 

 
GKFG-1464-MONOMER
 

 
mcu:HMPREF0573_11568 serA
 

 
2.5282066e-6
 

 
1.0
 

 
Q0S7M1
 

 
0.292173
 

 
2.0
 

 
0.07855626
 

 
471
 

 
T
 

 
NIL
 

 

 
     Evidence for cholest-4-en-3-one C26-hydroxylase, EC# 1.14.13.-   RXN-12848 
  

 

 
 Hit 
 

 
 Common 
 

 
 P 
 

 
 #Q 
 

 
 Best Qry 
 

 
 Best Eval 
 

 
 Avg Rank 
 

 
 Aln Len 
 

 
 Qry Len 
 

 
 IDOP? 
 

 
 adj? 
 

 

 

 
GKFG-1062-MONOMER
 

 
mcu:HMPREF0573_10372 rho;
 

 
1.5421621e-5
 

 
1.0
 

 
Q0S7M1
 

 
0.0598865
 

 
1.0
 

 
0.1910828
 

 
471
 

 
T
 

 
NIL
 

 

 

 
GKFG-1464-MONOMER
 

 
mcu:HMPREF0573_11568 serA
 

 
2.5282066e-6
 

 
1.0
 

 
Q0S7M1
 

 
0.292173
 

 
2.0
 

 
0.07855626
 

 
471
 

 
T
 

 
NIL
 

 

 
     

  glutathione-mediated detoxification II     Total # of reactions in pathway = 8   Present reactions: 1
  

 

 
 Reaction 
 

 
 Protein(s) 
 

 

 

 
RXN-6642
 

 
(# )
 

 

 
   Missing reactions: 7
  

 

 
 GSHTRAN-RXN 
 

 

 

 
 RXN-12532 
 

 

 

 
 RXN-12533 
 

 

 

 
 RXN-12534 
 

 

 

 
 RXN-6641 
 

 

 

 
 RXN-6763 
 

 

 

 
 THIOL-S-METHYLTRANSFERASE-RXN 
 

 

 
        Evidence for glutathione transferase, EC# 2.5.1.18   GSHTRAN-RXN 
  

 

 
 Hit 
 

 
 Common 
 

 
 P 
 

 
 #Q 
 

 
 Best Qry 
 

 
 Best Eval 
 

 
 Avg Rank 
 

 
 Aln Len 
 

 
 Qry Len 
 

 
 IDOP? 
 

 
 adj? 
 

 

 

 
GKFG-1401-MONOMER
 

 
Gst; glutathione S-transf
 

 
0.02949142
 

 
8.0
 

 
P36156
 

 
9.54268e-6
 

 
1.0
 

 
0.4969112
 

 
370
 

 
T
 

 
NIL
 

 

 

 
GKFG-661-MONOMER
 

 
mcu:HMPREF0573_11338 tsf;
 

 
0.008898924
 

 
1.0
 

 
P40582
 

 
0.107583
 

 
1.0
 

 
0.8076923
 

 
234
 

 
T
 

 
NIL
 

 

 

 
GKFG-1875-MONOMER
 

 
ahe:Arch_1100 glutamyl-tR
 

 
0.0018147272
 

 
1.0
 

 
P81942
 

 
0.0638463
 

 
1.0
 

 
0.5952381
 

 
210
 

 
T
 

 
NIL
 

 

 

 
GKFG-526-MONOMER
 

 
YidC/Oxa1 family membrane
 

 
3.7905585e-4
 

 
1.0
 

 
O18598
 

 
0.224644
 

 
1.0
 

 
0.27450982
 

 
204
 

 
NIL
 

 
NIL
 

 

 

 
GKFG-83-MONOMER
 

 
transmembrane_regions (db
 

 
3.7905585e-4
 

 
1.0
 

 
P46088
 

 
0.290067
 

 
1.0
 

 
0.2857143
 

 
203
 

 
NIL
 

 
NIL
 

 

 

 
GKFG-1465-MONOMER
 

 
iva:Isova_1268 UvrD/REP h
 

 
3.182795e-4
 

 
1.0
 

 
O74830
 

 
0.345391
 

 
1.0
 

 
0.37727273
 

 
220
 

 
T
 

 
NIL
 

 

 

 
GKFG-1857-MONOMER
 

 
aai:AARI_29350 hsdM; type
 

 
3.182795e-4
 

 
1.0
 

 
P30102
 

 
0.370486
 

 
1.0
 

 
0.40837696
 

 
191
 

 
T
 

 
NIL
 

 

 

 
GKFG-651-MONOMER
 

 
mcu:HMPREF0573_11422 proS
 

 
1.3958463e-4
 

 
1.0
 

 
P15214
 

 
0.311195
 

 
1.0
 

 
0.32019705
 

 
203
 

 
T
 

 
NIL
 

 

 

 
GKFG-1678-MONOMER
 

 
mcu:HMPREF0573_11450 valS
 

 
1.3958463e-4
 

 
1.0
 

 
Q9JHF7
 

 
0.313367
 

 
1.0
 

 
0.2964824
 

 
199
 

 
T
 

 
NIL
 

 

 

 
GKFG-1527-MONOMER
 

 
ADP-ribose pyrophosphatas
 

 
1.3958463e-4
 

 
1.0
 

 
P42769
 

 
0.476593
 

 
1.0
 

 
0.27522936
 

 
218
 

 
T
 

 
NIL
 

 

 

 
GKFG-1755-MONOMER
 

 
fal:FRAAL2180 dnaE2; erro
 

 
1.3958463e-4
 

 
1.0
 

 
Q18973
 

 
0.701554
 

 
1.0
 

 
0.31555554
 

 
225
 

 
T
 

 
NIL
 

 

 

 
GKFG-1819-MONOMER
 

 
ahe:Arch_0813 threonyl-tR
 

 
1.3958463e-4
 

 
1.0
 

 
P81065
 

 
0.18941
 

 
1.0
 

 
0.27860695
 

 
201
 

 
T
 

 
NIL
 

 

 

 
GKFG-1196-MONOMER
 

 
rha:RHA1_ro05116 sugar AB
 

 
1.3958463e-4
 

 
1.0
 

 
O16116
 

 
0.269826
 

 
1.0
 

 
0.28985506
 

 
207
 

 
T
 

 
NIL
 

 

 

 
GKFG-1770-MONOMER
 

 
surA; cell surface protei
 

 
1.3958463e-4
 

 
1.0
 

 
D2YW48
 

 
0.0214451
 

 
1.0
 

 
0.3116883
 

 
231
 

 
T
 

 
NIL
 

 

 

 
GKFG-701-MONOMER
 

 
ach:Achl_1330 fibronectin
 

 
5.771539e-5
 

 
1.0
 

 
O18598
 

 
0.635871
 

 
2.0
 

 
0.3970588
 

 
204
 

 
T
 

 
NIL
 

 

 

 
GKFG-1089-MONOMER
 

 
147998..148516 - ( gc_con
 

 
2.5307965e-5
 

 
1.0
 

 
P40582
 

 
0.593922
 

 
2.0
 

 
0.2735043
 

 
234
 

 
T
 

 
NIL
 

 

 

 
GKFG-1416-MONOMER
 

 
Putative uncharacterized 
 

 
2.5307965e-5
 

 
1.0
 

 
P81065
 

 
0.385693
 

 
2.0
 

 
0.2885572
 

 
201
 

 
T
 

 
NIL
 

 

 

 
GKFG-1533-MONOMER
 

 
mcu:HMPREF0573_10095 pncB
 

 
1.5421621e-5
 

 
1.0
 

 
Q12390
 

 
0.188508
 

 
1.0
 

 
0.16309012
 

 
233
 

 
T
 

 
NIL
 

 

 

 
GKFG-962-MONOMER
 

 
menC; O-succinylbenzoate-
 

 
1.5421621e-5
 

 
1.0
 

 
Q9P6M1
 

 
0.49557
 

 
1.0
 

 
0.14666666
 

 
225
 

 
T
 

 
NIL
 

 

 

 
GKFG-1248-MONOMER
 

 
cfi:Celf_2759 glycogen de
 

 
1.5421621e-5
 

 
1.0
 

 
Q8ILQ7
 

 
0.916971
 

 
1.0
 

 
0.21327014
 

 
211
 

 
T
 

 
NIL
 

 

 

 
GKFG-1041-MONOMER
 

 
coiled-coil (db=Coil db_i
 

 
1.5421621e-5
 

 
1.0
 

 
P26697
 

 
0.157892
 

 
1.0
 

 
0.13537118
 

 
229
 

 
T
 

 
NIL
 

 

 

 
GKFG-446-MONOMER
 

 
DNA-damage-inducible prot
 

 
7.594039e-6
 

 
1.0
 

 
Q94999
 

 
0.512908
 

 
2.0
 

 
0.1770335
 

 
209
 

 
NIL
 

 
NIL
 

 

 

 
GKFG-364-MONOMER
 

 
mcu:HMPREF0573_11145 aspC
 

 
7.594039e-6
 

 
1.0
 

 
Q61133
 

 
0.407706
 

 
2.0
 

 
0.12704918
 

 
244
 

 
NIL
 

 
NIL
 

 

 

 
GKFG-319-MONOMER
 

 
hypothetical protein (db=
 

 
7.594039e-6
 

 
1.0
 

 
P46419
 

 
0.129785
 

 
2.0
 

 
0.23287672
 

 
219
 

 
NIL
 

 
NIL
 

 

 

 
GKFG-353-MONOMER
 

 
ahe:Arch_0262 glycerol ki
 

 
2.9678517e-6
 

 
1.0
 

 
P81065
 

 
0.499172
 

 
3.0
 

 
0.09950249
 

 
201
 

 
NIL
 

 
NIL
 

 

 
     Evidence for , EC# 3.4.17.-   RXN-12532 
  
no BLAST hits found
 
     Evidence for , EC# 3.4.13.-   RXN-12533      Evidence for , EC# 2.3.1.-   RXN-12534      Evidence for , EC# 3.4.19.9   RXN-6641 
  
no BLAST hits found
 
     Evidence for , EC# 4.4.1.6   RXN-6763 
  

 

 
 Hit 
 

 
 Common 
 

 
 P 
 

 
 #Q 
 

 
 Best Qry 
 

 
 Best Eval 
 

 
 Avg Rank 
 

 
 Aln Len 
 

 
 Qry Len 
 

 
 IDOP? 
 

 
 adj? 
 

 

 

 
GKFG-364-MONOMER
 

 
mcu:HMPREF0573_11145 aspC
 

 
0.9275211
 

 
1.0
 

 
Q8L0M8
 

 
1.17822e-13
 

 
1.0
 

 
0.86340207
 

 
388
 

 
NIL
 

 
NIL
 

 

 

 
GKFG-510-MONOMER
 

 
bcv:Bcav_3969 histidinol-
 

 
1.5675386e-4
 

 
1.0
 

 
Q8L0M8
 

 
1.4031e-5
 

 
2.0
 

 
0.46134022
 

 
388
 

 
NIL
 

 
NIL
 

 

 

 
GKFG-999-MONOMER
 

 
mcu:HMPREF0573_10302 hemL
 

 
1.09376415e-5
 

 
1.0
 

 
Q8L0M8
 

 
0.0237725
 

 
3.0
 

 
0.33505154
 

 
388
 

 
T
 

 
NIL
 

 

 
     Evidence for thiol  S -methyltransferase, EC# 2.1.1.9   THIOL-S-METHYLTRANSFERASE-RXN 
  

 

 
 Hit 
 

 
 Common 
 

 
 P 
 

 
 #Q 
 

 
 Best Qry 
 

 
 Best Eval 
 

 
 Avg Rank 
 

 
 Aln Len 
 

 
 Qry Len 
 

 
 IDOP? 
 

 
 adj? 
 

 

 

 
GKFG-577-MONOMER
 

 
putative toxin-antitoxin 
 

 
1.5421621e-5
 

 
1.0
 

 
O80562
 

 
0.676782
 

 
1.0
 

 
0.2246696
 

 
227
 

 
T
 

 
NIL
 

 

 
     

  superoxide radicals degradation     Total # of reactions in pathway = 2   Present reactions: 1
  

 

 
 Reaction 
 

 
 Protein(s) 
 

 

 

 
SUPEROX-DISMUT-RXN
 

 
(# )
 

 

 
   Missing reactions: 1
  

 

 
 CATAL-RXN 
 

 

 
        Evidence for catalase, EC# 1.11.1.6   CATAL-RXN 
  

 

 
 Hit 
 

 
 Common 
 

 
 P 
 

 
 #Q 
 

 
 Best Qry 
 

 
 Best Eval 
 

 
 Avg Rank 
 

 
 Aln Len 
 

 
 Qry Len 
 

 
 IDOP? 
 

 
 adj? 
 

 

 

 
GKFG-1136-MONOMER
 

 
UniRef90_C2KNZ3 Putative 
 

 
3.182795e-4
 

 
1.0
 

 
Q97FE0
 

 
0.868761
 

 
1.0
 

 
0.37777779
 

 
225
 

 
T
 

 
NIL
 

 

 

 
GKFG-558-MONOMER
 

 
histidine kinase; K07768 
 

 
4.188785e-5
 

 
1.0
 

 
P24168
 

 
0.571046
 

 
1.0
 

 
0.13934426
 

 
488
 

 
NIL
 

 
NIL
 

 

 

 
GKFG-536-MONOMER
 

 
mcu:HMPREF0573_10685 gyrB
 

 
3.787886e-5
 

 
1.0
 

 
O28050
 

 
0.981077
 

 
1.0
 

 
0.11605938
 

 
741
 

 
NIL
 

 
NIL
 

 

 

 
GKFG-1239-MONOMER
 

 
(db=HMMPfam db_id=PF05738
 

 
1.5421621e-5
 

 
1.0
 

 
P37743
 

 
0.29568
 

 
1.0
 

 
0.2204861
 

 
576
 

 
T
 

 
NIL
 

 

 

 
GKFG-821-MONOMER
 

 
bad:BAD_0776 icd1; isocit
 

 
1.5421621e-5
 

 
1.0
 

 
P80878
 

 
0.530308
 

 
1.0
 

 
0.22710623
 

 
273
 

 
T
 

 
NIL
 

 

 

 
GKFG-838-MONOMER
 

 
bcv:Bcav_1314 N-acetylglu
 

 
1.5421621e-5
 

 
1.0
 

 
B1M869
 

 
0.0296895
 

 
1.0
 

 
0.1515544
 

 
772
 

 
T
 

 
NIL
 

 

 

 
GKFG-790-MONOMER
 

 
bcv:Bcav_2038 carbamoyl-p
 

 
1.3945618e-5
 

 
1.0
 

 
P50979
 

 
0.983561
 

 
1.0
 

 
0.0509915
 

 
706
 

 
T
 

 
NIL
 

 

 

 
GKFG-817-MONOMER
 

 
mph:MLP_05080 pgi; glucos
 

 
1.3945618e-5
 

 
1.0
 

 
P15202
 

 
0.198986
 

 
1.0
 

 
0.06407767
 

 
515
 

 
T
 

 
NIL
 

 

 

 
GKFG-1568-MONOMER
 

 
phage terminase protein (
 

 
1.3945618e-5
 

 
1.0
 

 
A4QUT2
 

 
0.134697
 

 
1.0
 

 
0.0826972
 

 
786
 

 
T
 

 
NIL
 

 

 
     

  pyruvate fermentation to butanol I     Total # of reactions in pathway = 8   Present reactions: 3
  

 

 
 Reaction 
 

 
 Protein(s) 
 

 

 

 
ACETYL-COA-ACETYLTRANSFER-RXN
 

 
(# )
 

 

 

 
PYRUFLAVREDUCT-RXN
 

 
(# 
 # 
 # )
 

 

 

 
BUTYRYL-COA-DEHYDROGENASE-RXN
 

 
(# )
 

 

 
   Missing reactions: 5
  

 

 
 BUTANAL-DEHYDROGENASE-RXN 
 

 

 

 
 ENZRXN-161-RXN 
 

 

 

 
 ENZRXN-201-RXN 
 

 

 

 
 RXN-11662 
 

 

 

 
 RXN-11667 
 

 

 
        Evidence for butanal dehydrogenase, EC# 1.2.1.57   BUTANAL-DEHYDROGENASE-RXN 
  

 

 
 Hit 
 

 
 Common 
 

 
 P 
 

 
 #Q 
 

 
 Best Qry 
 

 
 Best Eval 
 

 
 Avg Rank 
 

 
 Aln Len 
 

 
 Qry Len 
 

 
 IDOP? 
 

 
 adj? 
 

 

 

 
GKFG-1235-MONOMER
 

 
msm:MSMEG_0582 succinate-
 

 
0.0018147272
 

 
2.0
 

 
Q9ANR5
 

 
8.09898e-10
 

 
1.0
 

 
0.5075893
 

 
858
 

 
T
 

 
NIL
 

 

 

 
GKFG-977-MONOMER
 

 
paz:TIA2EST2_02200 methyl
 

 
5.771539e-5
 

 
2.0
 

 
Q9ANR5
 

 
4.12243e-7
 

 
2.0
 

 
0.4073562
 

 
858
 

 
T
 

 
NIL
 

 

 

 
GKFG-997-MONOMER
 

 
ssm:Spirs_0789 aldehyde d
 

 
1.4625372e-5
 

 
1.0
 

 
Q9ANR5
 

 
3.19042e-5
 

 
4.0
 

 
0.27272728
 

 
858
 

 
T
 

 
NIL
 

 

 

 
GKFG-1221-MONOMER
 

 
thioredoxin domain-contai
 

 
3.972156e-6
 

 
1.0
 

 
Q9ANR5
 

 
0.681581
 

 
5.0
 

 
0.066433564
 

 
858
 

 
T
 

 
NIL
 

 

 

 
GKFG-328-MONOMER
 

 
mcu:HMPREF0573_11678 L-pr
 

 
1.2082764e-6
 

 
1.0
 

 
Q9ANR5
 

 
9.88359e-6
 

 
3.0
 

 
0.1981352
 

 
858
 

 
T
 

 
NIL
 

 

 
     Evidence for , EC# 1.1.1.-   ENZRXN-161-RXN 
  

 

 
 Hit 
 

 
 Common 
 

 
 P 
 

 
 #Q 
 

 
 Best Qry 
 

 
 Best Eval 
 

 
 Avg Rank 
 

 
 Aln Len 
 

 
 Qry Len 
 

 
 IDOP? 
 

 
 adj? 
 

 

 

 
GKFG-935-MONOMER
 

 
ahe:Arch_0038 alcohol deh
 

 
0.95706683
 

 
1.0
 

 
P39451
 

 
3.08215e-44
 

 
1.0
 

 
1.0
 

 
336
 

 
T
 

 
NIL
 

 

 

 
GKFG-1960-MONOMER
 

 
mcu:HMPREF0573_10477 adhC
 

 
0.805337
 

 
1.0
 

 
P39451
 

 
4.91702e-33
 

 
2.0
 

 
0.95238096
 

 
336
 

 
T
 

 
NIL
 

 

 

 
GKFG-1882-MONOMER
 

 
pcn:TIB1ST10_03215 oxidor
 

 
0.37966776
 

 
1.0
 

 
P39451
 

 
2.50021e-23
 

 
4.0
 

 
0.95535713
 

 
336
 

 
T
 

 
NIL
 

 

 

 
GKFG-860-MONOMER
 

 
msm:MSMEG_3094 oxidoreduc
 

 
0.18173979
 

 
1.0
 

 
P39451
 

 
4.56615e-32
 

 
3.0
 

 
0.7083333
 

 
336
 

 
T
 

 
NIL
 

 

 

 
GKFG-1235-MONOMER
 

 
msm:MSMEG_0582 succinate-
 

 
0.0018147272
 

 
1.0
 

 
P33744
 

 
1.01994e-6
 

 
1.0
 

 
0.49419954
 

 
862
 

 
T
 

 
NIL
 

 

 

 
GKFG-977-MONOMER
 

 
paz:TIA2EST2_02200 methyl
 

 
3.294785e-4
 

 
1.0
 

 
P33744
 

 
1.67919e-4
 

 
2.0
 

 
0.49419954
 

 
862
 

 
T
 

 
NIL
 

 

 
     Evidence for , EC# 1.1.1.-   ENZRXN-201-RXN 
  

 

 
 Hit 
 

 
 Common 
 

 
 P 
 

 
 #Q 
 

 
 Best Qry 
 

 
 Best Eval 
 

 
 Avg Rank 
 

 
 Aln Len 
 

 
 Qry Len 
 

 
 IDOP? 
 

 
 adj? 
 

 

 

 
GKFG-720-MONOMER
 

 
fabG; 3-ketoacyl-ACP redu
 

 
0.8538447
 

 
2.0
 

 
Q9FZ42
 

 
3.26905e-29
 

 
1.0
 

 
0.96221405
 

 
288
 

 
T
 

 
NIL
 

 

 

 
GKFG-803-MONOMER
 

 
short-chain dehydrogenase
 

 
0.09592287
 

 
2.0
 

 
Q9SQR2
 

 
4.50261e-11
 

 
2.0
 

 
0.704759
 

 
272
 

 
T
 

 
NIL
 

 

 

 
GKFG-300-MONOMER
 

 
UniRef90_F9EI64 ABC super
 

 
4.0159098e-4
 

 
1.0
 

 
Q9FZ42
 

 
0.176778
 

 
3.0
 

 
0.6597222
 

 
288
 

 
T
 

 
NIL
 

 

 

 
GKFG-1089-MONOMER
 

 
147998..148516 - ( gc_con
 

 
3.182795e-4
 

 
1.0
 

 
Q0PGJ6
 

 
0.952555
 

 
1.0
 

 
0.43174604
 

 
315
 

 
T
 

 
NIL
 

 

 

 
GKFG-632-MONOMER
 

 
rdn:HMPREF0733_11588 dinG
 

 
1.4241917e-4
 

 
1.0
 

 
Q9SQR2
 

 
0.555285
 

 
3.0
 

 
0.48161766
 

 
272
 

 
T
 

 
NIL
 

 

 

 
GKFG-691-MONOMER
 

 
phosphotransferase system
 

 
1.4625372e-5
 

 
1.0
 

 
Q9SQR2
 

 
0.610582
 

 
4.0
 

 
0.2757353
 

 
272
 

 
T
 

 
NIL
 

 

 
     Evidence for , EC# 1.1.1.35   RXN-11662 
  
no BLAST hits found
 
     Evidence for (S)-3-hydroxybutanoyl-CoA dehydrogenase, EC# 4.2.1.-   RXN-11667 
  

 

 
 Hit 
 

 
 Common 
 

 
 P 
 

 
 #Q 
 

 
 Best Qry 
 

 
 Best Eval 
 

 
 Avg Rank 
 

 
 Aln Len 
 

 
 Qry Len 
 

 
 IDOP? 
 

 
 adj? 
 

 

 

 
GKFG-964-MONOMER
 

 
pak:HMPREF0675_3964 menB;
 

 
0.8538447
 

 
1.0
 

 
A5N5C7
 

 
5.44063e-21
 

 
1.0
 

 
1.0
 

 
259
 

 
T
 

 
NIL
 

 

 

 
GKFG-1235-MONOMER
 

 
msm:MSMEG_0582 succinate-
 

 
0.035064183
 

 
1.0
 

 
P77455
 

 
3.97539e-14
 

 
1.0
 

 
0.41556534
 

 
681
 

 
T
 

 
NIL
 

 

 

 
GKFG-997-MONOMER
 

 
ssm:Spirs_0789 aldehyde d
 

 
1.4241917e-4
 

 
1.0
 

 
P77455
 

 
7.06938e-8
 

 
3.0
 

 
0.56828195
 

 
681
 

 
T
 

 
NIL
 

 

 

 
GKFG-977-MONOMER
 

 
paz:TIA2EST2_02200 methyl
 

 
5.771539e-5
 

 
1.0
 

 
P77455
 

 
6.07981e-10
 

 
2.0
 

 
0.46108663
 

 
681
 

 
T
 

 
NIL
 

 

 

 
GKFG-328-MONOMER
 

 
mcu:HMPREF0573_11678 L-pr
 

 
3.3353957e-5
 

 
1.0
 

 
P77455
 

 
2.09464e-4
 

 
4.0
 

 
0.42143905
 

 
681
 

 
T
 

 
NIL
 

 

 
     

  mixed acid fermentation     Total # of reactions in pathway = 16   Present reactions: 13
  

 

 
 Reaction 
 

 
 Protein(s) 
 

 

 

 
PHOSACETYLTRANS-RXN
 

 
(# )
 

 

 

 
ACETATEKIN-RXN
 

 
(# )
 

 

 

 
ACETALD-DEHYDROG-RXN
 

 
(# )
 

 

 

 
ALCOHOL-DEHYDROG-RXN
 

 
(# 
 # )
 

 

 

 
PEPDEPHOS-RXN
 

 
(# )
 

 

 

 
DLACTDEHYDROGNAD-RXN
 

 
(# )
 

 

 

 
FUMHYDR-RXN
 

 
(# )
 

 

 

 
MALATE-DEH-RXN
 

 
(# )
 

 

 

 
R601-RXN
 

 
(# )
 

 

 

 
CITSYN-RXN
 

 
(# )
 

 

 

 
ACONITATEDEHYDR-RXN
 

 
(# )
 

 

 

 
ACONITATEHYDR-RXN
 

 
(# )
 

 

 

 
ISOCITDEH-RXN
 

 
(# )
 

 

 
   Missing reactions: 3
  

 

 
 PYRUVFORMLY-RXN 
 

 

 

 
 PEPCARBOX-RXN 
 

 

 

 
 FHLMULTI-RXN 
 

 

 
        Evidence for formate  C -acetyltransferase, EC# 2.3.1.54   PYRUVFORMLY-RXN 
  

 

 
 Hit 
 

 
 Common 
 

 
 P 
 

 
 #Q 
 

 
 Best Qry 
 

 
 Best Eval 
 

 
 Avg Rank 
 

 
 Aln Len 
 

 
 Qry Len 
 

 
 IDOP? 
 

 
 adj? 
 

 

 

 
GKFG-1796-MONOMER
 

 
pac:PPA0489 UTP-glucose-1
 

 
3.787886e-5
 

 
1.0
 

 
P43753
 

 
0.742193
 

 
1.0
 

 
0.10519481
 

 
770
 

 
NIL
 

 
NIL
 

 

 

 
GKFG-347-MONOMER
 

 
ahe:Arch_0333 N-acetylglu
 

 
1.3945618e-5
 

 
1.0
 

 
P42632
 

 
0.619868
 

 
1.0
 

 
0.107329845
 

 
764
 

 
T
 

 
NIL
 

 

 
     Evidence for phospho enol pyruvate carboxylase, EC# 4.1.1.31   PEPCARBOX-RXN 
  

 

 
 Hit 
 

 
 Common 
 

 
 P 
 

 
 #Q 
 

 
 Best Qry 
 

 
 Best Eval 
 

 
 Avg Rank 
 

 
 Aln Len 
 

 
 Qry Len 
 

 
 IDOP? 
 

 
 adj? 
 

 

 

 
GKFG-1046-MONOMER
 

 
xce:Xcel_0723 ATP-depende
 

 
1.3945618e-5
 

 
1.0
 

 
O32483
 

 
0.139123
 

 
1.0
 

 
0.053418804
 

 
936
 

 
T
 

 
NIL
 

 

 

 
GKFG-289-MONOMER
 

 
mcu:HMPREF0573_10751 puta
 

 
1.5421621e-5
 

 
1.0
 

 
A8AW99
 

 
0.703679
 

 
1.0
 

 
0.21518987
 

 
948
 

 
T
 

 
NIL
 

 

 

 
GKFG-1465-MONOMER
 

 
iva:Isova_1268 UvrD/REP h
 

 
1.5421621e-5
 

 
1.0
 

 
A4VN44
 

 
0.215022
 

 
1.0
 

 
0.1592719
 

 
879
 

 
T
 

 
NIL
 

 

 

 
GKFG-1767-MONOMER
 

 
hypothetical protein (db=
 

 
1.5421621e-5
 

 
1.0
 

 
Q0STS8
 

 
0.046729
 

 
1.0
 

 
0.1359404
 

 
537
 

 
T
 

 
NIL
 

 

 

 
GKFG-594-MONOMER
 

 
hypothetical protein (db=
 

 
1.5421621e-5
 

 
1.0
 

 
B0R7F9
 

 
0.884405
 

 
1.0
 

 
0.14634146
 

 
492
 

 
T
 

 
NIL
 

 

 

 
GKFG-765-MONOMER
 

 
cfl:Cfla_2325 Preprotein 
 

 
1.5421621e-5
 

 
1.0
 

 
Q8TZL5
 

 
0.14614
 

 
1.0
 

 
0.17372881
 

 
472
 

 
T
 

 
NIL
 

 

 

 
GKFG-142-MONOMER
 

 
dsy:DSY3812 hypothetical 
 

 
1.3945618e-5
 

 
1.0
 

 
Q8PS70
 

 
0.667179
 

 
1.0
 

 
0.10076046
 

 
526
 

 
T
 

 
NIL
 

 

 

 
GKFG-1621-MONOMER
 

 
tcu:Tcur_3714 3-phosphosh
 

 
1.3945618e-5
 

 
1.0
 

 
O28786
 

 
0.102436
 

 
1.0
 

 
0.10403397
 

 
471
 

 
T
 

 
NIL
 

 

 

 
GKFG-739-MONOMER
 

 
bcv:Bcav_1600 DEAD/DEAH b
 

 
1.3945618e-5
 

 
1.0
 

 
A1R2V3
 

 
0.792382
 

 
1.0
 

 
0.04828326
 

 
932
 

 
T
 

 
NIL
 

 

 

 
GKFG-1304-MONOMER
 

 
UniRef90_UPI000051013A hy
 

 
1.3945618e-5
 

 
1.0
 

 
Q5SKL7
 

 
0.671567
 

 
1.0
 

 
0.09440559
 

 
858
 

 
T
 

 
NIL
 

 

 

 
GKFG-732-MONOMER
 

 
ahe:Arch_1078 LuxR family
 

 
1.3945618e-5
 

 
1.0
 

 
Q2SL23
 

 
0.350988
 

 
1.0
 

 
0.08399546
 

 
881
 

 
T
 

 
NIL
 

 

 

 
GKFG-1347-MONOMER
 

 
aur:HMPREF9243_0883 hypot
 

 
1.3945618e-5
 

 
1.0
 

 
A4JGV5
 

 
0.0294267
 

 
1.0
 

 
0.055165496
 

 
997
 

 
T
 

 
NIL
 

 

 

 
GKFG-1068-MONOMER
 

 
ckp:ckrop_1874 hypothetic
 

 
1.3945618e-5
 

 
1.0
 

 
Q93MH3
 

 
0.252621
 

 
1.0
 

 
0.054406963
 

 
919
 

 
T
 

 
NIL
 

 

 

 
GKFG-86-MONOMER
 

 
Putative uncharacterized 
 

 
2.5282066e-6
 

 
2.0
 

 
O32483
 

 
0.238824
 

 
1.5
 

 
0.09476648
 

 
936
 

 
T
 

 
NIL
 

 

 

 
GKFG-1383-MONOMER
 

 
jde:Jden_1695 DEAD/DEAH b
 

 
2.5282066e-6
 

 
2.0
 

 
A4JGV5
 

 
0.0496707
 

 
1.5
 

 
0.070196435
 

 
997
 

 
T
 

 
NIL
 

 

 

 
GKFG-844-MONOMER
 

 
ahe:Arch_0366 ATP synthas
 

 
2.5282066e-6
 

 
1.0
 

 
A8AW99
 

 
0.828791
 

 
2.0
 

 
0.07489452
 

 
948
 

 
T
 

 
NIL
 

 

 

 
GKFG-177-MONOMER
 

 
ubiE; methylase; K03183 u
 

 
1.0926308e-6
 

 
1.0
 

 
O32483
 

 
0.310147
 

 
3.0
 

 
0.086538464
 

 
936
 

 
T
 

 
NIL
 

 

 
     Evidence for    FHLMULTI-RXN 
  

 

 
 Hit 
 

 
 Common 
 

 
 P 
 

 
 #Q 
 

 
 Best Qry 
 

 
 Best Eval 
 

 
 Avg Rank 
 

 
 Aln Len 
 

 
 Qry Len 
 

 
 IDOP? 
 

 
 adj? 
 

 

 

 
GKFG-171-MONOMER
 

 
ahe:Arch_0296 NADH dehydr
 

 
0.7392329
 

 
1.0
 

 
P16431
 

 
1.99058e-33
 

 
1.0
 

 
0.71704745
 

 
569
 

 
T
 

 
NIL
 

 

 

 
GKFG-173-MONOMER
 

 
NADH dehydrogenase subuni
 

 
0.42053136
 

 
1.0
 

 
P16433
 

 
3.31047e-30
 

 
1.0
 

 
0.6745098
 

 
255
 

 
T
 

 
NIL
 

 

 

 
GKFG-165-MONOMER
 

 
bcv:Bcav_3221 NADH-quinon
 

 
0.17184281
 

 
2.0
 

 
P16432
 

 
1.26543e-15
 

 
1.0
 

 
0.58943623
 

 
180
 

 
T
 

 
NIL
 

 

 

 
GKFG-162-MONOMER
 

 
mcu:HMPREF0573_11003 nuoL
 

 
0.036253624
 

 
1.0
 

 
P16429
 

 
7.22414e-16
 

 
2.0
 

 
0.5378289
 

 
608
 

 
T
 

 
NIL
 

 

 

 
GKFG-160-MONOMER
 

 
mcu:HMPREF0573_11005 nuoN
 

 
0.01599724
 

 
1.0
 

 
P16429
 

 
9.37633e-13
 

 
3.0
 

 
0.57236844
 

 
608
 

 
T
 

 
NIL
 

 

 

 
GKFG-161-MONOMER
 

 
bcv:Bcav_3217 proton-tran
 

 
0.015683802
 

 
1.0
 

 
P16429
 

 
2.47501e-18
 

 
1.0
 

 
0.32894737
 

 
608
 

 
T
 

 
NIL
 

 

 

 
GKFG-166-MONOMER
 

 
bcv:Bcav_3222 NADH dehydr
 

 
0.0051016076
 

 
1.0
 

 
P16430
 

 
5.93133e-9
 

 
1.0
 

 
0.6872964
 

 
307
 

 
T
 

 
NIL
 

 

 

 
GKFG-1191-MONOMER
 

 
pfr:PFREUD_12970 glpC; an
 

 
9.2874106e-4
 

 
1.0
 

 
P16432
 

 
0.00678462
 

 
2.0
 

 
0.6333333
 

 
180
 

 
T
 

 
NIL
 

 

 

 
GKFG-433-MONOMER
 

 
mcu:HMPREF0573_10281 sdhB
 

 
1.4241917e-4
 

 
1.0
 

 
P16432
 

 
0.106695
 

 
3.0
 

 
0.5555556
 

 
180
 

 
T
 

 
NIL
 

 

 

 
GKFG-260-MONOMER
 

 
twh:TWT601 ABC transporte
 

 
2.5307965e-5
 

 
1.0
 

 
P16433
 

 
0.0821585
 

 
2.0
 

 
0.33333334
 

 
255
 

 
T
 

 
NIL
 

 

 

 
GKFG-172-MONOMER
 

 
mcu:HMPREF0573_10994 nuoC
 

 
2.5307965e-5
 

 
1.0
 

 
P16431
 

 
3.66705e-9
 

 
2.0
 

 
0.27768013
 

 
569
 

 
T
 

 
NIL
 

 

 

 
GKFG-167-MONOMER
 

 
jde:Jden_0545 NADH-quinon
 

 
1.5421621e-5
 

 
1.0
 

 
P07658
 

 
1.01689e-9
 

 
1.0
 

 
0.23776224
 

 
715
 

 
T
 

 
NIL
 

 

 
     

  acetyl-CoA fermentation to butyrate II     Total # of reactions in pathway = 6   Present reactions: 4
  

 

 
 Reaction 
 

 
 Protein(s) 
 

 

 

 
ACETYL-COA-ACETYLTRANSFER-RXN
 

 
(# )
 

 

 

 
PWY0-1312
 

 
NIL
 

 

 

 
RXN-5901
 

 
(# )
 

 

 

 
BUTYRYL-COA-DEHYDROGENASE-RXN
 

 
(# )
 

 

 
   Missing reactions: 2
  

 

 
 3-HYDROXBUTYRYL-COA-DEHYDRATASE-RXN 
 

 

 

 
 R11-RXN 
 

 

 
        Evidence for 3-hydroxybutyryl-CoA dehydratase, EC# 4.2.1.55   3-HYDROXBUTYRYL-COA-DEHYDRATASE-RXN 
  

 

 
 Hit 
 

 
 Common 
 

 
 P 
 

 
 #Q 
 

 
 Best Qry 
 

 
 Best Eval 
 

 
 Avg Rank 
 

 
 Aln Len 
 

 
 Qry Len 
 

 
 IDOP? 
 

 
 adj? 
 

 

 

 
GKFG-964-MONOMER
 

 
pak:HMPREF0675_3964 menB;
 

 
0.8538447
 

 
1.0
 

 
P52046
 

 
1.09798e-24
 

 
1.0
 

 
1.0
 

 
261
 

 
T
 

 
NIL
 

 

 
     Evidence for butyryl coenzyme A transferase, EC# 2.8.3.8   R11-RXN 
  

 

 
 Hit 
 

 
 Common 
 

 
 P 
 

 
 #Q 
 

 
 Best Qry 
 

 
 Best Eval 
 

 
 Avg Rank 
 

 
 Aln Len 
 

 
 Qry Len 
 

 
 IDOP? 
 

 
 adj? 
 

 

 

 
GKFG-718-MONOMER
 

 
mta:Moth_1259 propionate 
 

 
0.8249092
 

 
2.0
 

 
P76458
 

 
1.65522e-13
 

 
1.0
 

 
0.9964225
 

 
220
 

 
T
 

 
NIL
 

 

 

 
GKFG-410-MONOMER
 

 
hydrolase of the HAD supe
 

 
7.594039e-6
 

 
1.0
 

 
P76459
 

 
0.0929638
 

 
2.0
 

 
0.2361111
 

 
216
 

 
NIL
 

 
NIL
 

 

 

 
GKFG-101-MONOMER
 

 
hydrolase of the HAD supe
 

 
3.2819723e-6
 

 
1.0
 

 
P76459
 

 
0.0929638
 

 
3.0
 

 
0.2361111
 

 
216
 

 
NIL
 

 
NIL
 

 

 
     

  pyruvate fermentation to acetate IV     Total # of reactions in pathway = 4   Present reactions: 3
  

 

 
 Reaction 
 

 
 Protein(s) 
 

 

 

 
PHOSACETYLTRANS-RXN
 

 
(# )
 

 

 

 
ACETATEKIN-RXN
 

 
(# )
 

 

 

 
PWY0-1312
 

 
NIL
 

 

 
   Missing reactions: 1
  

 

 
 PYRUVFORMLY-RXN 
 

 

 
        Evidence for formate  C -acetyltransferase, EC# 2.3.1.54   PYRUVFORMLY-RXN 
  

 

 
 Hit 
 

 
 Common 
 

 
 P 
 

 
 #Q 
 

 
 Best Qry 
 

 
 Best Eval 
 

 
 Avg Rank 
 

 
 Aln Len 
 

 
 Qry Len 
 

 
 IDOP? 
 

 
 adj? 
 

 

 

 
GKFG-347-MONOMER
 

 
ahe:Arch_0333 N-acetylglu
 

 
1.3945618e-5
 

 
1.0
 

 
P42632
 

 
0.619868
 

 
1.0
 

 
0.107329845
 

 
764
 

 
T
 

 
NIL
 

 

 

 
GKFG-1796-MONOMER
 

 
pac:PPA0489 UTP-glucose-1
 

 
3.787886e-5
 

 
1.0
 

 
P43753
 

 
0.742193
 

 
1.0
 

 
0.10519481
 

 
770
 

 
NIL
 

 
NIL
 

 

 
     

  glycolysis I     Total # of reactions in pathway = 11   Present reactions: 10
  

 

 
 Reaction 
 

 
 Protein(s) 
 

 

 

 
PEPDEPHOS-RXN
 

 
(# )
 

 

 

 
2PGADEHYDRAT-RXN
 

 
(# )
 

 

 

 
3PGAREARR-RXN
 

 
(# 
 # 
 # 
 # )
 

 

 

 
PHOSGLYPHOS-RXN
 

 
(# )
 

 

 

 
GAPOXNPHOSPHN-RXN
 

 
(# )
 

 

 

 
TRIOSEPISOMERIZATION-RXN
 

 
(# )
 

 

 

 
F16ALDOLASE-RXN
 

 
(# )
 

 

 

 
6PFRUCTPHOS-RXN
 

 
(# )
 

 

 

 
PGLUCISOM-RXN
 

 
(# )
 

 

 

 
PEPSYNTH-RXN
 

 
(# )
 

 

 
   Missing reactions: 1
  

 

 
 F16BDEPHOS-RXN 
 

 

 
        Evidence for fructose-bisphosphatase, EC# 3.1.3.11   F16BDEPHOS-RXN 
  

 

 
 Hit 
 

 
 Common 
 

 
 P 
 

 
 #Q 
 

 
 Best Qry 
 

 
 Best Eval 
 

 
 Avg Rank 
 

 
 Aln Len 
 

 
 Qry Len 
 

 
 IDOP? 
 

 
 adj? 
 

 

 

 
GKFG-153-MONOMER
 

 
mcu:HMPREF0573_10425 ppdK
 

 
1.5421621e-5
 

 
1.0
 

 
A3D0P6
 

 
0.413008
 

 
1.0
 

 
0.20175439
 

 
342
 

 
T
 

 
NIL
 

 

 

 
GKFG-1229-MONOMER
 

 
hypothetical protein; K09
 

 
1.3958463e-4
 

 
1.0
 

 
C6BTU7
 

 
0.458626
 

 
1.0
 

 
0.295858
 

 
338
 

 
T
 

 
NIL
 

 

 

 
GKFG-1027-MONOMER
 

 
cfi:Celf_2525 mannose-6-p
 

 
1.3958463e-4
 

 
1.0
 

 
A1KHN9
 

 
0.810805
 

 
1.0
 

 
0.32320443
 

 
362
 

 
T
 

 
NIL
 

 

 

 
GKFG-933-MONOMER
 

 
HAD-superfamily hydrolase
 

 
3.3353957e-5
 

 
1.0
 

 
P21829
 

 
0.00268893
 

 
4.0
 

 
0.38235295
 

 
272
 

 
T
 

 
NIL
 

 

 

 
GKFG-1543-MONOMER
 

 
UBA/ThiF-type NAD/FAD bin
 

 
2.5307965e-5
 

 
1.0
 

 
P46726
 

 
0.899178
 

 
2.0
 

 
0.34317344
 

 
271
 

 
T
 

 
NIL
 

 

 

 
GKFG-681-MONOMER
 

 
archaeal fructose-1,6-bis
 

 
1.5421621e-5
 

 
3.0
 

 
P46726
 

 
0.0526868
 

 
1.0
 

 
0.23745935
 

 
271
 

 
T
 

 
NIL
 

 

 

 
GKFG-1563-MONOMER
 

 
hypothetical protein (db=
 

 
1.5421621e-5
 

 
1.0
 

 
O25936
 

 
0.252277
 

 
1.0
 

 
0.15172414
 

 
290
 

 
T
 

 
NIL
 

 

 

 
GKFG-304-MONOMER
 

 
hypothetical protein (db=
 

 
1.5421621e-5
 

 
1.0
 

 
Q5V3Z1
 

 
0.494724
 

 
1.0
 

 
0.23367697
 

 
291
 

 
T
 

 
NIL
 

 

 

 
GKFG-86-MONOMER
 

 
Putative uncharacterized 
 

 
1.5421621e-5
 

 
1.0
 

 
B0CAD9
 

 
0.0244708
 

 
1.0
 

 
0.19327731
 

 
357
 

 
T
 

 
NIL
 

 

 

 
GKFG-1275-MONOMER
 

 
metal ion ABC transporter
 

 
1.5421621e-5
 

 
1.0
 

 
Q2FM20
 

 
0.272969
 

 
1.0
 

 
0.15755627
 

 
311
 

 
T
 

 
NIL
 

 

 

 
GKFG-1632-MONOMER
 

 
ahe:Arch_1316 acetate kin
 

 
1.5421621e-5
 

 
1.0
 

 
B5EFV5
 

 
0.370326
 

 
1.0
 

 
0.17791411
 

 
326
 

 
T
 

 
NIL
 

 

 

 
GKFG-739-MONOMER
 

 
bcv:Bcav_1600 DEAD/DEAH b
 

 
1.5421621e-5
 

 
1.0
 

 
B2V6E2
 

 
0.348491
 

 
1.0
 

 
0.21671827
 

 
323
 

 
T
 

 
NIL
 

 

 

 
GKFG-935-MONOMER
 

 
ahe:Arch_0038 alcohol deh
 

 
1.5421621e-5
 

 
1.0
 

 
A5E987
 

 
0.14728
 

 
1.0
 

 
0.15680474
 

 
338
 

 
T
 

 
NIL
 

 

 

 
GKFG-410-MONOMER
 

 
hydrolase of the HAD supe
 

 
1.5421621e-5
 

 
1.0
 

 
P21829
 

 
7.54305e-6
 

 
1.0
 

 
0.23529412
 

 
272
 

 
T
 

 
NIL
 

 

 

 
GKFG-701-MONOMER
 

 
ach:Achl_1330 fibronectin
 

 
1.5421621e-5
 

 
1.0
 

 
P19112
 

 
0.0755288
 

 
1.0
 

 
0.21763085
 

 
363
 

 
T
 

 
NIL
 

 

 

 
GKFG-598-MONOMER
 

 
krh:KRH_14360 sigA; RNA p
 

 
1.3945618e-5
 

 
1.0
 

 
B0C7G7
 

 
0.366936
 

 
1.0
 

 
0.11782477
 

 
331
 

 
T
 

 
NIL
 

 

 

 
GKFG-66-MONOMER
 

 
kfl:Kfla_6989 hypothetica
 

 
1.3945618e-5
 

 
1.0
 

 
A9FZA0
 

 
0.460166
 

 
1.0
 

 
0.0873635
 

 
641
 

 
T
 

 
NIL
 

 

 

 
GKFG-1270-MONOMER
 

 
jde:Jden_1096 bifunctiona
 

 
1.3945618e-5
 

 
1.0
 

 
A7IL64
 

 
0.882531
 

 
1.0
 

 
0.10030395
 

 
329
 

 
T
 

 
NIL
 

 

 

 
GKFG-1882-MONOMER
 

 
pcn:TIB1ST10_03215 oxidor
 

 
1.3945618e-5
 

 
1.0
 

 
A7IGL7
 

 
0.937412
 

 
1.0
 

 
0.08625337
 

 
371
 

 
T
 

 
NIL
 

 

 

 
GKFG-176-MONOMER
 

 
bcv:Bcav_3230 geranylgera
 

 
1.3945618e-5
 

 
1.0
 

 
Q8D275
 

 
0.908721
 

 
1.0
 

 
0.10060976
 

 
328
 

 
T
 

 
NIL
 

 

 

 
GKFG-1699-MONOMER
 

 
mcu:HMPREF0573_11727 alaS
 

 
1.3945618e-5
 

 
1.0
 

 
A6X307
 

 
0.0588743
 

 
1.0
 

 
0.09117647
 

 
340
 

 
T
 

 
NIL
 

 

 

 
GKFG-1231-MONOMER
 

 
fma:FMG_0239 antibiotic A
 

 
1.3945618e-5
 

 
1.0
 

 
B1XVF7
 

 
0.0506714
 

 
1.0
 

 
0.06784661
 

 
339
 

 
T
 

 
NIL
 

 

 

 
GKFG-281-MONOMER
 

 
bfa:Bfae_19800 copper/sil
 

 
4.3925725e-6
 

 
1.0
 

 
P21829
 

 
0.55393
 

 
5.0
 

 
0.14338236
 

 
272
 

 
T
 

 
NIL
 

 

 

 
GKFG-1239-MONOMER
 

 
(db=HMMPfam db_id=PF05738
 

 
2.795795e-6
 

 
2.0
 

 
A6X307
 

 
0.50127
 

 
1.5
 

 
0.22415079
 

 
340
 

 
T
 

 
NIL
 

 

 

 
GKFG-101-MONOMER
 

 
hydrolase of the HAD supe
 

 
2.795795e-6
 

 
1.0
 

 
P21829
 

 
7.54305e-6
 

 
2.0
 

 
0.23529412
 

 
272
 

 
T
 

 
NIL
 

 

 

 
GKFG-75-MONOMER
 

 
Bacterial adhesins (db=su
 

 
2.5282066e-6
 

 
1.0
 

 
B5EFV5
 

 
0.521669
 

 
2.0
 

 
0.1196319
 

 
326
 

 
T
 

 
NIL
 

 

 

 
GKFG-1943-MONOMER
 

 
bcv:Bcav_1945 P-type HAD 
 

 
1.2082764e-6
 

 
1.0
 

 
P21829
 

 
0.00187333
 

 
3.0
 

 
0.1985294
 

 
272
 

 
T
 

 
NIL
 

 

 
     

  glycerol-3-phosphate shuttle     Total # of reactions in pathway = 2   Present reactions: 1
  

 

 
 Reaction 
 

 
 Protein(s) 
 

 

 

 
RXN0-5260
 

 
(# 
 # 
 # 
 # )
 

 

 
   Missing reactions: 1
  

 

 
 1.1.1.8-RXN 
 

 

 
        Evidence for glycerol-3-phosphate dehydrogenase (NAD  +  ), EC# 1.1.1.8   1.1.1.8-RXN 
  

 

 
 Hit 
 

 
 Common 
 

 
 P 
 

 
 #Q 
 

 
 Best Qry 
 

 
 Best Eval 
 

 
 Avg Rank 
 

 
 Aln Len 
 

 
 Qry Len 
 

 
 IDOP? 
 

 
 adj? 
 

 

 

 
GKFG-2024-MONOMER
 

 
pad:TIIST44_04395 glycero
 

 
0.99750745
 

 
9.0
 

 
Q4QHG4
 

 
1.05556e-19
 

 
1.0
 

 
0.6340903
 

 
367
 

 
T
 

 
T
 

 

 

 
GKFG-1480-MONOMER
 

 
UDP-N-acetylmuramate--L-a
 

 
2.5307965e-5
 

 
1.0
 

 
Q9UVF4
 

 
0.750801
 

 
2.0
 

 
0.298995
 

 
398
 

 
T
 

 
NIL
 

 

 

 
GKFG-442-MONOMER
 

 
mcu:HMPREF0573_10293 glmS
 

 
7.594039e-6
 

 
1.0
 

 
Q7ZA45
 

 
0.711052
 

 
2.0
 

 
0.18734793
 

 
411
 

 
NIL
 

 
NIL
 

 

 

 
GKFG-1544-MONOMER
 

 
bcv:Bcav_2890 hypothetica
 

 
2.795795e-6
 

 
1.0
 

 
Q4QHG4
 

 
0.0588331
 

 
2.0
 

 
0.1880109
 

 
367
 

 
T
 

 
NIL
 

 

 

 
GKFG-1689-MONOMER
 

 
AMP-dependent synthetase 
 

 
2.795795e-6
 

 
1.0
 

 
P41911
 

 
0.819287
 

 
2.0
 

 
0.23181818
 

 
440
 

 
T
 

 
NIL
 

 

 

 
GKFG-853-MONOMER
 

 
Sua5/YciO/YrdC/YwlC famil
 

 
2.5282066e-6
 

 
1.0
 

 
Q5G5B9
 

 
0.198164
 

 
2.0
 

 
0.09237875
 

 
433
 

 
T
 

 
NIL
 

 

 

 
GKFG-887-MONOMER
 

 
UniRef90_C2KTG6 Possible 
 

 
1.0926308e-6
 

 
1.0
 

 
Q7ZA45
 

 
0.727866
 

 
3.0
 

 
0.08272506
 

 
411
 

 
T
 

 
NIL
 

 

 
     

  photosynthesis light reactions     Total # of reactions in pathway = 4   Present reactions: 3
  

 

 
 Reaction 
 

 
 Protein(s) 
 

 

 

 
RXN490-3650
 

 
NIL
 

 

 

 
1.18.1.2-RXN
 

 
(# )
 

 

 

 
PLASTOQUINOL--PLASTOCYANIN-REDUCTASE-RXN
 

 
NIL
 

 

 
   Missing reactions: 1
  

 

 
 PSII-RXN 
 

 

 
        Evidence for photosystem II, EC# 1.10.3.9   PSII-RXN 
  

 

 
 Hit 
 

 
 Common 
 

 
 P 
 

 
 #Q 
 

 
 Best Qry 
 

 
 Best Eval 
 

 
 Avg Rank 
 

 
 Aln Len 
 

 
 Qry Len 
 

 
 IDOP? 
 

 
 adj? 
 

 

 

 
GKFG-526-MONOMER
 

 
YidC/Oxa1 family membrane
 

 
0.41138098
 

 
1.0
 

 
Q8LBP4
 

 
7.89712e-21
 

 
1.0
 

 
0.5800866
 

 
462
 

 
NIL
 

 
NIL
 

 

 

 
GKFG-894-MONOMER
 

 
pad:TIIST44_03795 toxin s
 

 
0.013736963
 

 
2.0
 

 
P14835
 

 
0.385393
 

 
1.0
 

 
0.66245997
 

 
64
 

 
NIL
 

 
NIL
 

 

 

 
GKFG-1136-MONOMER
 

 
UniRef90_C2KNZ3 Putative 
 

 
0.004913946
 

 
1.0
 

 
Q8KPP0
 

 
0.995529
 

 
1.0
 

 
0.51282054
 

 
39
 

 
NIL
 

 
NIL
 

 

 

 
GKFG-1533-MONOMER
 

 
mcu:HMPREF0573_10095 pncB
 

 
8.6405425e-4
 

 
1.0
 

 
P11472
 

 
0.0321933
 

 
1.0
 

 
0.4296029
 

 
277
 

 
NIL
 

 
NIL
 

 

 

 
GKFG-528-MONOMER
 

 
Ribonuclease P protein co
 

 
8.6405425e-4
 

 
1.0
 

 
Q8KPP3
 

 
0.141563
 

 
1.0
 

 
0.44578314
 

 
83
 

 
NIL
 

 
NIL
 

 

 

 
GKFG-2014-MONOMER
 

 
bcv:Bcav_2351 virulence f
 

 
3.7905585e-4
 

 
1.0
 

 
tr|P74797|P74797_SYNY3
 

 
0.140977
 

 
1.0
 

 
0.31428573
 

 
70
 

 
NIL
 

 
NIL
 

 

 

 
GKFG-911-MONOMER
 

 
nfa:nfa37970 hrpA; ATP-de
 

 
3.7905585e-4
 

 
1.0
 

 
tr|A8HXG5|A8HXG5_CHLRE
 

 
0.453704
 

 
1.0
 

 
0.29752067
 

 
121
 

 
NIL
 

 
NIL
 

 

 

 
GKFG-991-MONOMER
 

 
mcu:HMPREF0573_11504 hypo
 

 
3.7905585e-4
 

 
1.0
 

 
Q9LXX5
 

 
0.542513
 

 
1.0
 

 
0.2519084
 

 
262
 

 
NIL
 

 
NIL
 

 

 

 
GKFG-807-MONOMER
 

 
TetR family transcription
 

 
1.5675386e-4
 

 
1.0
 

 
tr|A8HXG5|A8HXG5_CHLRE
 

 
0.584618
 

 
2.0
 

 
0.42975205
 

 
121
 

 
NIL
 

 
NIL
 

 

 

 
GKFG-825-MONOMER
 

 
seg (db=Seg db_id=seg fro
 

 
6.873975e-5
 

 
1.0
 

 
P73070
 

 
0.759345
 

 
2.0
 

 
0.35897437
 

 
39
 

 
NIL
 

 
NIL
 

 

 

 
GKFG-329-MONOMER
 

 
mcu:HMPREF0573_11679 opuE
 

 
5.771539e-5
 

 
2.0
 

 
Q31RR2
 

 
0.508854
 

 
1.5
 

 
0.44882733
 

 
67
 

 
T
 

 
NIL
 

 

 

 
GKFG-766-MONOMER
 

 
sro:Sros_2818 methionine 
 

 
4.188785e-5
 

 
1.0
 

 
P04996
 

 
0.23961
 

 
1.0
 

 
0.15
 

 
360
 

 
NIL
 

 
NIL
 

 

 

 
GKFG-874-MONOMER
 

 
rer:RER_18960 phage porta
 

 
4.188785e-5
 

 
1.0
 

 
P31094
 

 
0.0726464
 

 
1.0
 

 
0.12795275
 

 
508
 

 
NIL
 

 
NIL
 

 

 

 
GKFG-1185-MONOMER
 

 
ele:Elen_1490 ABC transpo
 

 
4.188785e-5
 

 
1.0
 

 
tr|A8IYH9|A8IYH9_CHLRE
 

 
0.225778
 

 
1.0
 

 
0.17021276
 

 
282
 

 
NIL
 

 
NIL
 

 

 

 
GKFG-433-MONOMER
 

 
mcu:HMPREF0573_10281 sdhB
 

 
2.5307965e-5
 

 
1.0
 

 
Q8KPP3
 

 
0.567855
 

 
2.0
 

 
0.26506025
 

 
83
 

 
T
 

 
NIL
 

 

 

 
GKFG-432-MONOMER
 

 
ahe:Arch_1329 glycoprotea
 

 
1.5421621e-5
 

 
2.0
 

 
P11004
 

 
0.103985
 

 
1.0
 

 
0.19428672
 

 
461
 

 
T
 

 
NIL
 

 

 

 
GKFG-368-MONOMER
 

 
UniRef90_F2UVM4 PE-PGRS f
 

 
1.5421621e-5
 

 
1.0
 

 
P72575
 

 
0.487484
 

 
1.0
 

 
0.20512821
 

 
39
 

 
T
 

 
NIL
 

 

 

 
GKFG-214-MONOMER
 

 
304174..304446 - ( gc_con
 

 
1.5421621e-5
 

 
1.0
 

 
Q55332
 

 
0.241697
 

 
1.0
 

 
0.16793893
 

 
131
 

 
T
 

 
NIL
 

 

 
     

  respiration (anaerobic)     Total # of reactions in pathway = 13   Present reactions: 9
  

 

 
 Reaction 
 

 
 Protein(s) 
 

 

 

 
PEPDEPHOS-RXN
 

 
(# )
 

 

 

 
CITSYN-RXN
 

 
(# )
 

 

 

 
ACONITATEDEHYDR-RXN
 

 
(# )
 

 

 

 
ACONITATEHYDR-RXN
 

 
(# )
 

 

 

 
FUMHYDR-RXN
 

 
(# )
 

 

 

 
2PGADEHYDRAT-RXN
 

 
(# )
 

 

 

 
R601-RXN
 

 
(# )
 

 

 

 
MALATE-DEH-RXN
 

 
(# )
 

 

 

 
ISOCITDEH-RXN
 

 
(# )
 

 

 
   Missing reactions: 4
  

 

 
 PYRUVDEH-RXN 
 

 

 

 
 PEPCARBOX-RXN 
 

 

 

 
 PYRUVFORMLY-RXN 
 

 

 

 
 FHLMULTI-RXN 
 

 

 
        Evidence for , EC# 1.2.1.-   PYRUVDEH-RXN 
  

 

 
 Hit 
 

 
 Common 
 

 
 P 
 

 
 #Q 
 

 
 Best Qry 
 

 
 Best Eval 
 

 
 Avg Rank 
 

 
 Aln Len 
 

 
 Qry Len 
 

 
 IDOP? 
 

 
 adj? 
 

 

 

 
GKFG-1625-MONOMER
 

 
cga:Celgi_2334 2-oxogluta
 

 
0.015683802
 

 
1.0
 

 
P06959
 

 
1.05612e-12
 

 
1.0
 

 
0.2920635
 

 
630
 

 
T
 

 
NIL
 

 

 

 
GKFG-1950-MONOMER
 

 
bcv:Bcav_2215 pyruvate ki
 

 
0.002462277
 

 
1.0
 

 
P0A9P0
 

 
0.768297
 

 
5.0
 

 
0.1392405
 

 
474
 

 
T
 

 
T
 

 

 

 
GKFG-669-MONOMER
 

 
ahe:Arch_1322 alkyl hydro
 

 
3.182795e-4
 

 
1.0
 

 
P0A9P0
 

 
3.51896e-10
 

 
1.0
 

 
0.36919832
 

 
474
 

 
T
 

 
NIL
 

 

 

 
GKFG-900-MONOMER
 

 
ahe:Arch_1354 electron-tr
 

 
2.4944004e-5
 

 
1.0
 

 
P0A9P0
 

 
0.310782
 

 
3.0
 

 
0.37130803
 

 
474
 

 
T
 

 
NIL
 

 

 

 
GKFG-1737-MONOMER
 

 
cmi:CMM_1734 tktA; transk
 

 
1.5421621e-5
 

 
1.0
 

 
P0AFG8
 

 
0.00753695
 

 
1.0
 

 
0.19503947
 

 
887
 

 
T
 

 
NIL
 

 

 

 
GKFG-549-MONOMER
 

 
iva:Isova_3010 thioredoxi
 

 
6.867209e-6
 

 
1.0
 

 
P0A9P0
 

 
0.0052998
 

 
2.0
 

 
0.08438819
 

 
474
 

 
NIL
 

 
NIL
 

 

 

 
GKFG-1661-MONOMER
 

 
aau:AAur_3325 gdhA; gluta
 

 
1.6156636e-6
 

 
1.0
 

 
P0A9P0
 

 
0.366188
 

 
4.0
 

 
0.12236287
 

 
474
 

 
T
 

 
NIL
 

 

 
     Evidence for phospho enol pyruvate carboxylase, EC# 4.1.1.31   PEPCARBOX-RXN 
  

 

 
 Hit 
 

 
 Common 
 

 
 P 
 

 
 #Q 
 

 
 Best Qry 
 

 
 Best Eval 
 

 
 Avg Rank 
 

 
 Aln Len 
 

 
 Qry Len 
 

 
 IDOP? 
 

 
 adj? 
 

 

 

 
GKFG-1046-MONOMER
 

 
xce:Xcel_0723 ATP-depende
 

 
1.3945618e-5
 

 
1.0
 

 
O32483
 

 
0.139123
 

 
1.0
 

 
0.053418804
 

 
936
 

 
T
 

 
NIL
 

 

 

 
GKFG-289-MONOMER
 

 
mcu:HMPREF0573_10751 puta
 

 
1.5421621e-5
 

 
1.0
 

 
A8AW99
 

 
0.703679
 

 
1.0
 

 
0.21518987
 

 
948
 

 
T
 

 
NIL
 

 

 

 
GKFG-1465-MONOMER
 

 
iva:Isova_1268 UvrD/REP h
 

 
1.5421621e-5
 

 
1.0
 

 
A4VN44
 

 
0.215022
 

 
1.0
 

 
0.1592719
 

 
879
 

 
T
 

 
NIL
 

 

 

 
GKFG-1767-MONOMER
 

 
hypothetical protein (db=
 

 
1.5421621e-5
 

 
1.0
 

 
Q0STS8
 

 
0.046729
 

 
1.0
 

 
0.1359404
 

 
537
 

 
T
 

 
NIL
 

 

 

 
GKFG-594-MONOMER
 

 
hypothetical protein (db=
 

 
1.5421621e-5
 

 
1.0
 

 
B0R7F9
 

 
0.884405
 

 
1.0
 

 
0.14634146
 

 
492
 

 
T
 

 
NIL
 

 

 

 
GKFG-765-MONOMER
 

 
cfl:Cfla_2325 Preprotein 
 

 
1.5421621e-5
 

 
1.0
 

 
Q8TZL5
 

 
0.14614
 

 
1.0
 

 
0.17372881
 

 
472
 

 
T
 

 
NIL
 

 

 

 
GKFG-142-MONOMER
 

 
dsy:DSY3812 hypothetical 
 

 
1.3945618e-5
 

 
1.0
 

 
Q8PS70
 

 
0.667179
 

 
1.0
 

 
0.10076046
 

 
526
 

 
T
 

 
NIL
 

 

 

 
GKFG-1621-MONOMER
 

 
tcu:Tcur_3714 3-phosphosh
 

 
1.3945618e-5
 

 
1.0
 

 
O28786
 

 
0.102436
 

 
1.0
 

 
0.10403397
 

 
471
 

 
T
 

 
NIL
 

 

 

 
GKFG-739-MONOMER
 

 
bcv:Bcav_1600 DEAD/DEAH b
 

 
1.3945618e-5
 

 
1.0
 

 
A1R2V3
 

 
0.792382
 

 
1.0
 

 
0.04828326
 

 
932
 

 
T
 

 
NIL
 

 

 

 
GKFG-1304-MONOMER
 

 
UniRef90_UPI000051013A hy
 

 
1.3945618e-5
 

 
1.0
 

 
Q5SKL7
 

 
0.671567
 

 
1.0
 

 
0.09440559
 

 
858
 

 
T
 

 
NIL
 

 

 

 
GKFG-732-MONOMER
 

 
ahe:Arch_1078 LuxR family
 

 
1.3945618e-5
 

 
1.0
 

 
Q2SL23
 

 
0.350988
 

 
1.0
 

 
0.08399546
 

 
881
 

 
T
 

 
NIL
 

 

 

 
GKFG-1347-MONOMER
 

 
aur:HMPREF9243_0883 hypot
 

 
1.3945618e-5
 

 
1.0
 

 
A4JGV5
 

 
0.0294267
 

 
1.0
 

 
0.055165496
 

 
997
 

 
T
 

 
NIL
 

 

 

 
GKFG-1068-MONOMER
 

 
ckp:ckrop_1874 hypothetic
 

 
1.3945618e-5
 

 
1.0
 

 
Q93MH3
 

 
0.252621
 

 
1.0
 

 
0.054406963
 

 
919
 

 
T
 

 
NIL
 

 

 

 
GKFG-86-MONOMER
 

 
Putative uncharacterized 
 

 
2.5282066e-6
 

 
2.0
 

 
O32483
 

 
0.238824
 

 
1.5
 

 
0.09476648
 

 
936
 

 
T
 

 
NIL
 

 

 

 
GKFG-1383-MONOMER
 

 
jde:Jden_1695 DEAD/DEAH b
 

 
2.5282066e-6
 

 
2.0
 

 
A4JGV5
 

 
0.0496707
 

 
1.5
 

 
0.070196435
 

 
997
 

 
T
 

 
NIL
 

 

 

 
GKFG-844-MONOMER
 

 
ahe:Arch_0366 ATP synthas
 

 
2.5282066e-6
 

 
1.0
 

 
A8AW99
 

 
0.828791
 

 
2.0
 

 
0.07489452
 

 
948
 

 
T
 

 
NIL
 

 

 

 
GKFG-177-MONOMER
 

 
ubiE; methylase; K03183 u
 

 
1.0926308e-6
 

 
1.0
 

 
O32483
 

 
0.310147
 

 
3.0
 

 
0.086538464
 

 
936
 

 
T
 

 
NIL
 

 

 
     Evidence for formate  C -acetyltransferase, EC# 2.3.1.54   PYRUVFORMLY-RXN 
  

 

 
 Hit 
 

 
 Common 
 

 
 P 
 

 
 #Q 
 

 
 Best Qry 
 

 
 Best Eval 
 

 
 Avg Rank 
 

 
 Aln Len 
 

 
 Qry Len 
 

 
 IDOP? 
 

 
 adj? 
 

 

 

 
GKFG-1796-MONOMER
 

 
pac:PPA0489 UTP-glucose-1
 

 
3.787886e-5
 

 
1.0
 

 
P43753
 

 
0.742193
 

 
1.0
 

 
0.10519481
 

 
770
 

 
NIL
 

 
NIL
 

 

 

 
GKFG-347-MONOMER
 

 
ahe:Arch_0333 N-acetylglu
 

 
1.3945618e-5
 

 
1.0
 

 
P42632
 

 
0.619868
 

 
1.0
 

 
0.107329845
 

 
764
 

 
T
 

 
NIL
 

 

 
     Evidence for    FHLMULTI-RXN 
  

 

 
 Hit 
 

 
 Common 
 

 
 P 
 

 
 #Q 
 

 
 Best Qry 
 

 
 Best Eval 
 

 
 Avg Rank 
 

 
 Aln Len 
 

 
 Qry Len 
 

 
 IDOP? 
 

 
 adj? 
 

 

 

 
GKFG-171-MONOMER
 

 
ahe:Arch_0296 NADH dehydr
 

 
0.7392329
 

 
1.0
 

 
P16431
 

 
1.99058e-33
 

 
1.0
 

 
0.71704745
 

 
569
 

 
T
 

 
NIL
 

 

 

 
GKFG-173-MONOMER
 

 
NADH dehydrogenase subuni
 

 
0.42053136
 

 
1.0
 

 
P16433
 

 
3.31047e-30
 

 
1.0
 

 
0.6745098
 

 
255
 

 
T
 

 
NIL
 

 

 

 
GKFG-165-MONOMER
 

 
bcv:Bcav_3221 NADH-quinon
 

 
0.17184281
 

 
2.0
 

 
P16432
 

 
1.26543e-15
 

 
1.0
 

 
0.58943623
 

 
180
 

 
T
 

 
NIL
 

 

 

 
GKFG-162-MONOMER
 

 
mcu:HMPREF0573_11003 nuoL
 

 
0.036253624
 

 
1.0
 

 
P16429
 

 
7.22414e-16
 

 
2.0
 

 
0.5378289
 

 
608
 

 
T
 

 
NIL
 

 

 

 
GKFG-160-MONOMER
 

 
mcu:HMPREF0573_11005 nuoN
 

 
0.01599724
 

 
1.0
 

 
P16429
 

 
9.37633e-13
 

 
3.0
 

 
0.57236844
 

 
608
 

 
T
 

 
NIL
 

 

 

 
GKFG-161-MONOMER
 

 
bcv:Bcav_3217 proton-tran
 

 
0.015683802
 

 
1.0
 

 
P16429
 

 
2.47501e-18
 

 
1.0
 

 
0.32894737
 

 
608
 

 
T
 

 
NIL
 

 

 

 
GKFG-166-MONOMER
 

 
bcv:Bcav_3222 NADH dehydr
 

 
0.0051016076
 

 
1.0
 

 
P16430
 

 
5.93133e-9
 

 
1.0
 

 
0.6872964
 

 
307
 

 
T
 

 
NIL
 

 

 

 
GKFG-1191-MONOMER
 

 
pfr:PFREUD_12970 glpC; an
 

 
9.2874106e-4
 

 
1.0
 

 
P16432
 

 
0.00678462
 

 
2.0
 

 
0.6333333
 

 
180
 

 
T
 

 
NIL
 

 

 

 
GKFG-433-MONOMER
 

 
mcu:HMPREF0573_10281 sdhB
 

 
1.4241917e-4
 

 
1.0
 

 
P16432
 

 
0.106695
 

 
3.0
 

 
0.5555556
 

 
180
 

 
T
 

 
NIL
 

 

 

 
GKFG-260-MONOMER
 

 
twh:TWT601 ABC transporte
 

 
2.5307965e-5
 

 
1.0
 

 
P16433
 

 
0.0821585
 

 
2.0
 

 
0.33333334
 

 
255
 

 
T
 

 
NIL
 

 

 

 
GKFG-172-MONOMER
 

 
mcu:HMPREF0573_10994 nuoC
 

 
2.5307965e-5
 

 
1.0
 

 
P16431
 

 
3.66705e-9
 

 
2.0
 

 
0.27768013
 

 
569
 

 
T
 

 
NIL
 

 

 

 
GKFG-167-MONOMER
 

 
jde:Jden_0545 NADH-quinon
 

 
1.5421621e-5
 

 
1.0
 

 
P07658
 

 
1.01689e-9
 

 
1.0
 

 
0.23776224
 

 
715
 

 
T
 

 
NIL
 

 

 
     

  TCA cycle V (2-oxoglutarate:ferredoxin oxidoreductase)     Total # of reactions in pathway = 12   Present reactions: 8
  

 

 
 Reaction 
 

 
 Protein(s) 
 

 

 

 
CITSYN-RXN
 

 
(# )
 

 

 

 
ACONITATEDEHYDR-RXN
 

 
(# )
 

 

 

 
ACONITATEHYDR-RXN
 

 
(# )
 

 

 

 
SUCC-FUM-OXRED-RXN
 

 
(# 
 # 
 # )
 

 

 

 
FUMHYDR-RXN
 

 
(# )
 

 

 

 
MALATE-DEH-RXN
 

 
(# )
 

 

 

 
ISOCITDEH-RXN
 

 
(# )
 

 

 

 
2-OXOGLUTARATE-SYNTHASE-RXN
 

 
NIL
 

 

 
   Missing reactions: 4
  

 

 
 ISOCIT-CLEAV-RXN 
 

 

 

 
 MALSYN-RXN 
 

 

 

 
 RXNI-2 
 

 

 

 
 RXN-12912 
 

 

 
        Evidence for isocitrate lyase, EC# 4.1.3.1   ISOCIT-CLEAV-RXN 
  

 

 
 Hit 
 

 
 Common 
 

 
 P 
 

 
 #Q 
 

 
 Best Qry 
 

 
 Best Eval 
 

 
 Avg Rank 
 

 
 Aln Len 
 

 
 Qry Len 
 

 
 IDOP? 
 

 
 adj? 
 

 

 

 
GKFG-1815-MONOMER
 

 
lipid A biosynthesis acyl
 

 
1.5421621e-5
 

 
2.0
 

 
Q43097
 

 
1.50841e-4
 

 
1.0
 

 
0.21169952
 

 
580
 

 
T
 

 
NIL
 

 

 

 
GKFG-1133-MONOMER
 

 
hypothetical protein (db=
 

 
1.3945618e-5
 

 
1.0
 

 
Q10663
 

 
0.327332
 

 
1.0
 

 
0.05268595
 

 
968
 

 
T
 

 
NIL
 

 

 
     Evidence for malate synthase, EC# 2.3.3.9   MALSYN-RXN 
  

 

 
 Hit 
 

 
 Common 
 

 
 P 
 

 
 #Q 
 

 
 Best Qry 
 

 
 Best Eval 
 

 
 Avg Rank 
 

 
 Aln Len 
 

 
 Qry Len 
 

 
 IDOP? 
 

 
 adj? 
 

 

 

 
GKFG-1133-MONOMER
 

 
hypothetical protein (db=
 

 
1.3945618e-5
 

 
1.0
 

 
Q10663
 

 
0.327332
 

 
1.0
 

 
0.05268595
 

 
968
 

 
T
 

 
NIL
 

 

 

 
GKFG-1988-MONOMER
 

 
cga:Celgi_1384 (Glutamate
 

 
1.3945618e-5
 

 
1.0
 

 
Q8T2K9
 

 
0.249717
 

 
1.0
 

 
0.08103131
 

 
543
 

 
T
 

 
NIL
 

 

 

 
GKFG-838-MONOMER
 

 
bcv:Bcav_1314 N-acetylglu
 

 
1.3945618e-5
 

 
1.0
 

 
P37330
 

 
0.73556
 

 
1.0
 

 
0.045643155
 

 
723
 

 
T
 

 
NIL
 

 

 
     Evidence for acetoacetate CoA-transferase, EC# 2.8.3.5   RXNI-2 
  

 

 
 Hit 
 

 
 Common 
 

 
 P 
 

 
 #Q 
 

 
 Best Qry 
 

 
 Best Eval 
 

 
 Avg Rank 
 

 
 Aln Len 
 

 
 Qry Len 
 

 
 IDOP? 
 

 
 adj? 
 

 

 

 
GKFG-718-MONOMER
 

 
mta:Moth_1259 propionate 
 

 
0.5061234
 

 
1.0
 

 
P55809
 

 
8.26278e-19
 

 
1.0
 

 
0.8096154
 

 
520
 

 
T
 

 
NIL
 

 

 
     Evidence for , EC# 2.6.1.-   RXN-12912      

  TCA cycle I (prokaryotic)     Total # of reactions in pathway = 10   Present reactions: 9
  

 

 
 Reaction 
 

 
 Protein(s) 
 

 

 

 
FUMHYDR-RXN
 

 
(# )
 

 

 

 
SUCCCOASYN-RXN
 

 
(# 
 # )
 

 

 

 
2OXOGLUTARATEDEH-RXN
 

 
(# )
 

 

 

 
ACONITATEHYDR-RXN
 

 
(# )
 

 

 

 
CITSYN-RXN
 

 
(# )
 

 

 

 
ACONITATEDEHYDR-RXN
 

 
(# )
 

 

 

 
SUCCINATE-DEHYDROGENASE-UBIQUINONE-RXN
 

 
(# )
 

 

 

 
MALATE-DEH-RXN
 

 
(# )
 

 

 

 
ISOCITDEH-RXN
 

 
(# )
 

 

 
   Missing reactions: 1
  

 

 
 MALATE-DEHYDROGENASE-ACCEPTOR-RXN 
 

 

 
        Evidence for malate dehydrogenase (quinone), EC# 1.1.5.4   MALATE-DEHYDROGENASE-ACCEPTOR-RXN 
  

 

 
 Hit 
 

 
 Common 
 

 
 P 
 

 
 #Q 
 

 
 Best Qry 
 

 
 Best Eval 
 

 
 Avg Rank 
 

 
 Aln Len 
 

 
 Qry Len 
 

 
 IDOP? 
 

 
 adj? 
 

 

 

 
GKFG-1594-MONOMER
 

 
hypothetical protein (db=
 

 
1.5421621e-5
 

 
1.0
 

 
Q7V8S6
 

 
0.557301
 

 
1.0
 

 
0.13508065
 

 
496
 

 
T
 

 
NIL
 

 

 

 
GKFG-801-MONOMER
 

 
ahe:Arch_0192 fructose-bi
 

 
1.5421621e-5
 

 
1.0
 

 
B2FSQ8
 

 
0.587058
 

 
1.0
 

 
0.16192171
 

 
562
 

 
T
 

 
NIL
 

 

 

 
GKFG-900-MONOMER
 

 
ahe:Arch_1354 electron-tr
 

 
1.3945618e-5
 

 
2.0
 

 
Q9ZMY5
 

 
0.141854
 

 
1.0
 

 
0.0991121
 

 
450
 

 
T
 

 
NIL
 

 

 

 
GKFG-1031-MONOMER
 

 
pantoate/beta-alanine lig
 

 
1.3945618e-5
 

 
1.0
 

 
Q7VRS0
 

 
0.418221
 

 
1.0
 

 
0.08015267
 

 
524
 

 
T
 

 
NIL
 

 

 

 
GKFG-1504-MONOMER
 

 
hypothetical protein (db=
 

 
2.795795e-6
 

 
1.0
 

 
Q7V8S6
 

 
0.557301
 

 
2.0
 

 
0.13508065
 

 
496
 

 
T
 

 
NIL
 

 

 
   
